# Generation and Use of Bicyclo[1.1.0]butyllithium under Continuous Flow Conditions

**DOI:** 10.1021/acs.orglett.5c00705

**Published:** 2025-03-20

**Authors:** Elena Graziano, Marco Colella, Marcus Baumann, Renzo Luisi

**Affiliations:** † FLAME-Lab, Flow Chemistry and Microreactor Technology Laboratory, Department of Pharmacy-Drug Sciences, University of Bari “A. Moro”, Via Edoardo Orabona 4, 70125 Bari, Italy; ‡ School of Chemistry, University College Dublin, O’Brien Centre for Science, Belfield, Dublin 4, Ireland

## Abstract

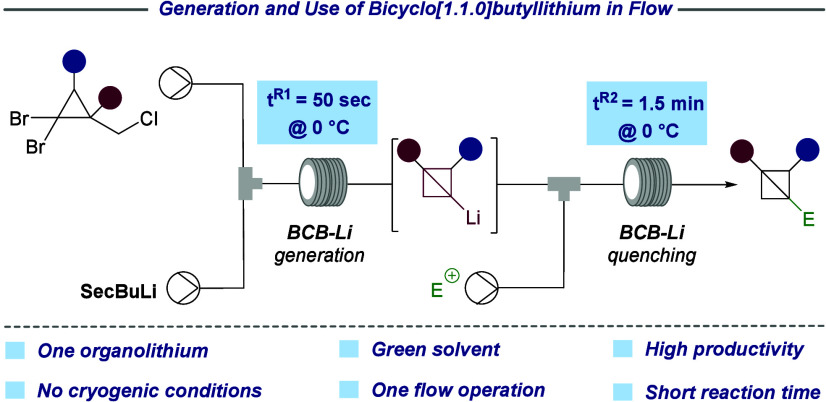

The bicyclo[1.1.0]butyl scaffold has emerged as a valuable bioisostere in drug discovery programs. Here, we present a streamlined approach for the generation of bicyclo[1.1.0]butyllithium and its functionalization with various classes of electrophiles in a one-flow process, eliminating the need for intermediate isolation. In comparison to traditional batch processes, the flow method allows the use of a single organolithium reagent instead of two and operates at significantly higher temperatures (0 °C versus −78 °C), enhancing both practicality and scalability.

Bicyclo[1.1.0]butanes (BCBs) represent a unique class of highly strained organic molecules characterized by exceptional reactivity.^1,2^ The significant ring strain of this four-membered carbocycle, defined by its bridging C(1)–C(3) bond, imparts BCBs with a remarkable propensity for diverse ring-opening reactions.^3,4^ This distinctive reactivity has made them indispensable building blocks in organic synthesis and has driven considerable interest in developing efficient and versatile methods for their preparation. Their utility spans multiple domains, including medicinal chemistry, materials science, and catalysis. Derivatives of BCBs have recently been used as key building blocks in radical reactions, facilitating the formation of complex and often unprecedented structural motifs.^5−8^ Furthermore, their inclusion in biologically active compounds and natural products has underscored their importance in drug discovery and chemical biology. Several bioactive structures featuring the BCB motif have been identified, further highlighting their synthetic and biological relevance (Figure 1).^9−12^ The bicyclo[1.1.0]butyl scaffold was first synthesized in 1959 by Wiberg and Cuila,^13^ while the parent bicyclo[1.1.0]butane was reported in 1963.^14^ Over the years, several strategies have been developed for the preparation and functionalization of the bicyclo[1.1.0]butane framework. These include the 3-exo-tet cyclization of cyclopropane or cyclobutane precursors, the cyclopropanation of unsaturated functional groups, and the functionalization of bicyclo[1.1.0]butyl lithium with various electrophiles.^3,15^ One of the most versatile methods for the synthesis of BCBs was developed by Brinker and co-workers in 1999.^16^ This approach employs 1,1-dibromocyclopropanes as starting materials, which are conveniently synthesized via dibromocarbene cyclopropanation of allyl chlorides.

**Figure 1:**
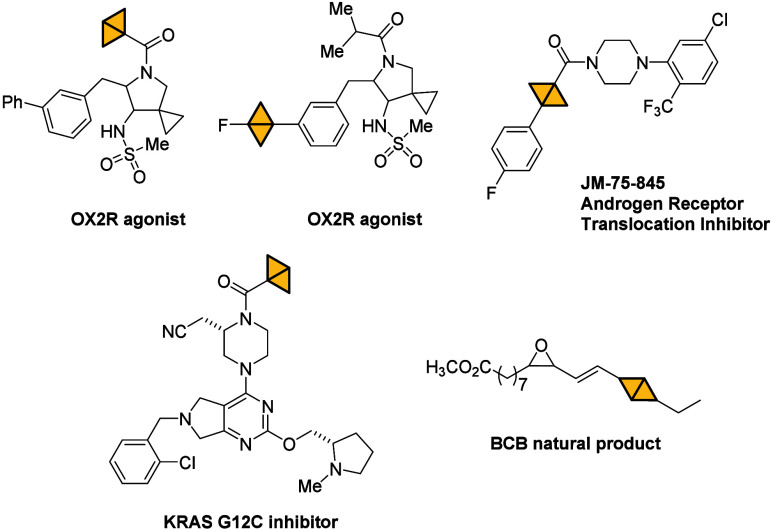
Biorelevant molecules containing the BCB motif in their structure.

The treatment of these trihalides with 2 equiv of an alkylithium reagent produces bicyclobutyllithium species that can be trapped subsequently with a variety of electrophiles to yield functionalized BCBs. In another significant contribution, Gassman et al. demonstrated that lithiated BCBs could be obtained directly through the deprotonation of the bridgehead C–H bond using an organolithium reagent.^17^ Moreover, the current need to introduce new bioisosteres and increase the C(sp^3^) fraction in pharmaceutically relevant molecules has reignited interest in the chemistry of BCBs.^18^ Following the pioneering work of Wipf and co-workers,^19,20^ several research groups, including those led by Malins,^21^ Anderson,^22^ and Glorius,^23^ among others, rediscovered and further explored the unique chemistry and reactivity of BCBs. A particularly notable contribution came from Aggarwal and co-workers, who developed innovative synthetic strategies centered around bicyclobutyllithium (BCB-Li).^24−27^ For the generation of highly versatile BCB-Li from cyclopropane **1a** (Scheme 1A), traditional batch protocols involve the use of two different organolithium reagents (i.e., MeLi and *t*-BuLi) under cryogenic conditions (temperatures at or below −78 °C). Malins and co-workers proposed a method to prepare a stock solution of BCB bromide **2** after removal of MeBr under a high vacuum (Scheme 1A). This solution was then used to generate BCB-Li, although through reaction with *t*-BuLi at low temperatures. However, this procedure poses challenges for process intensification. Aggarwal and co-workers first introduced BCB sulfoxide **4** as a stable, isolable precursor for BCB-Li, also derived from compound **1a** (Scheme 1A). While this advancement provides a stable precursor, the procedures to generate BCB-Li still require cryogenic conditions (−78 or −95 °C), rapid (1 min) *ex situ* or *in situ* quenching protocols, and the use of pyrophoric *t*-BuLi to achieve lithiation and subsequent electrophilic functionalization with boronates or carbonyl compounds.^24^

**Scheme 1:**
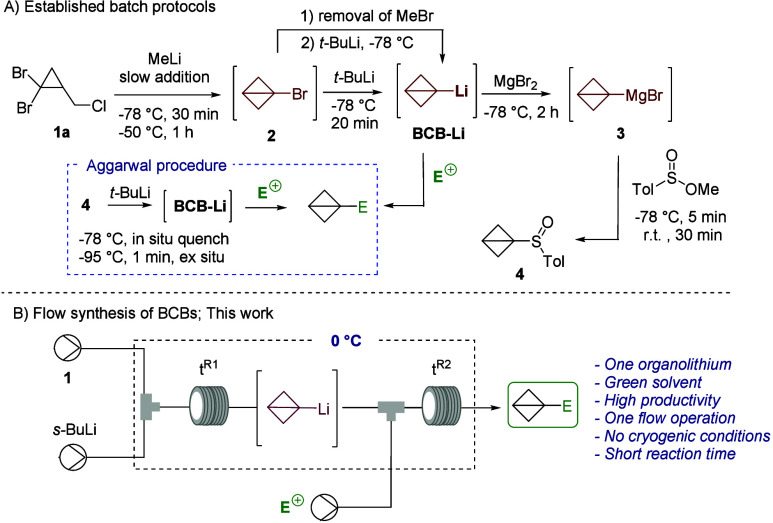
Generation of BCB-Li in Batch and Flow

However, the exceptional efficacy of the described elegant protocols for the synthesis and functionalization of BCBs cannot overlook the increasingly stringent requirements for sustainability and reduced environmental impact demanded by the chemical and pharmaceutical industries. In this context, the adoption of modern technologies, such as flow technology, aimed at making synthetic processes less environmentally impactful, simpler, and safer is of paramount importance.^28^ Building upon our expertise in flow chemistry,^29−32^ we present herein a simple and streamlined approach for the generation of BCB-Li and its subsequent functionalization with electrophiles in an one-flow fashion starting from readily available compound **1a** without isolation of any intermediates. This flow process utilizes a single organolithium reagent, namely, *s*-BuLi, which is less hazardous and more practical compared to MeLi and highly pyrophoric *t*-BuLi. The entire process operates at significantly higher temperatures than traditional batch methods (0 °C versus −78 °C), enhancing its practicality and scalability. Furthermore, 2-methyltetrahydrofuran (2-MeTHF) was chosen as a greener alternative to tetrahydrofuran and diethyl ether, which is conventionally used for BCB-Li generation. Our investigation began using the flow setup described in Table 1, consisting of two T-mixers (M1 and M2), each followed by a coil (V1 and V2) with volumes of 1.12 and 2.77 mL, respectively. Starting with compound **1a** and using *s*-BuLi, putative BCB-Li was generated in M1 and R1 and then transferred to M2, where it reacted with *p*-chlorobenzaldehyde, selected as a model electrophile, to yield BCB **5a**. The optimization study primarily focused on the residence times (*t*^R1^ and *t*^R2^). By increasing *t*^R1^ from 15 to 50 s while keeping *t*^R2^ constant at 2.5 min, we observed a significant increase in yield, from 16 to 92% (entries 1–5 in Table 1). However, further extension of *t*^R1^ resulted in a sharp decline in the yield, likely due to the rapid decomposition of BCB-Li (entries 6–8 in Table 1). Further evaluation of the quenching section of the flow reactor (M2 and V2) revealed that reducing *t*^R2^ from 2.5 to 1.5 min led to an additional improvement in the yield, achieving 95% for product **5a** (entries 9 and 10 in Table 1). Interestingly, when the flow process was conducted at room temperature, only traces of product **5a** were observed likely due to the thermal instability of BCB-Li (entry 11 in Table 1). The use of *n*-BuLi resulted in a 23% yield of product **5a** (entry 12 in Table 1).

**Table 1:**
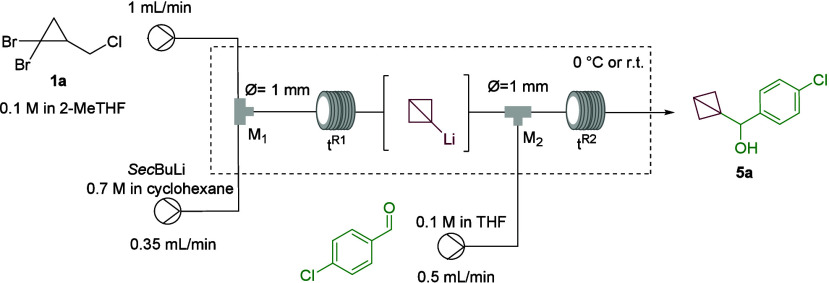
Optimization Study

entry	*t*^R1^	*t*^R2^	yield (%)^a^
1	15 s	2.5 min	16
2	25 s	2.5 min	48
3	30 s	2.5 min	81
4	40 s	2.5 min	89
5	50 s	2.5 min	92
6	1 min	2.5 min	55
7	2 min	2.5 min	4
8	5 min	2.5 min	3
9	50 s	1 min	93
10	50 s	1.5 min	95
11	50 s	1.5 min	traces at rt
12	50 s	1.5 min	23

a Reported yields are gas chromatography (GC) yields of compound **5a** using dodecane as the internal standard.

Conditions in entry 10 in Table 1 were selected as optimal conditions for exploring the reaction scope under continuous flow (Scheme 2). The use of aromatic and α,β-unsaturated aldehydes provided the corresponding BCB adducts **5a**–**5d** in excellent yields (>91%). Similarly, aromatic, heteroaromatic, and α,β-unsaturated aliphatic ketones smoothly reacted with BCB-Li providing the corresponding tertiary alcohols **5e**–**5o** in good to excellent yields. Imines were competent electrophiles, furnishing the BCB-substituted amine **5p** in 63% yield. The use of Weinreb amides as acylating reactants provided BCB ketones **5q**–**5t** in good yields in the range of 48–72% (Scheme 2). For comparison, when ethyl benzoate was employed as the electrophile, clean conversion into the corresponding double addition product **5u** was observed in 54% yield. The addition of BCB-Li to *N*-sulfinyltritylamine (TrNSO) provides desired sulfinamide **5v** in a satisfactory yield, demonstrating that this methodology is not limited to carbonyl electrophiles. Remarkably, the use of bioactive fenofibrate as a competent electrophilic partner led to the corresponding BCB derivative **5w** in an acceptable 25% yield.

**Scheme 2:**
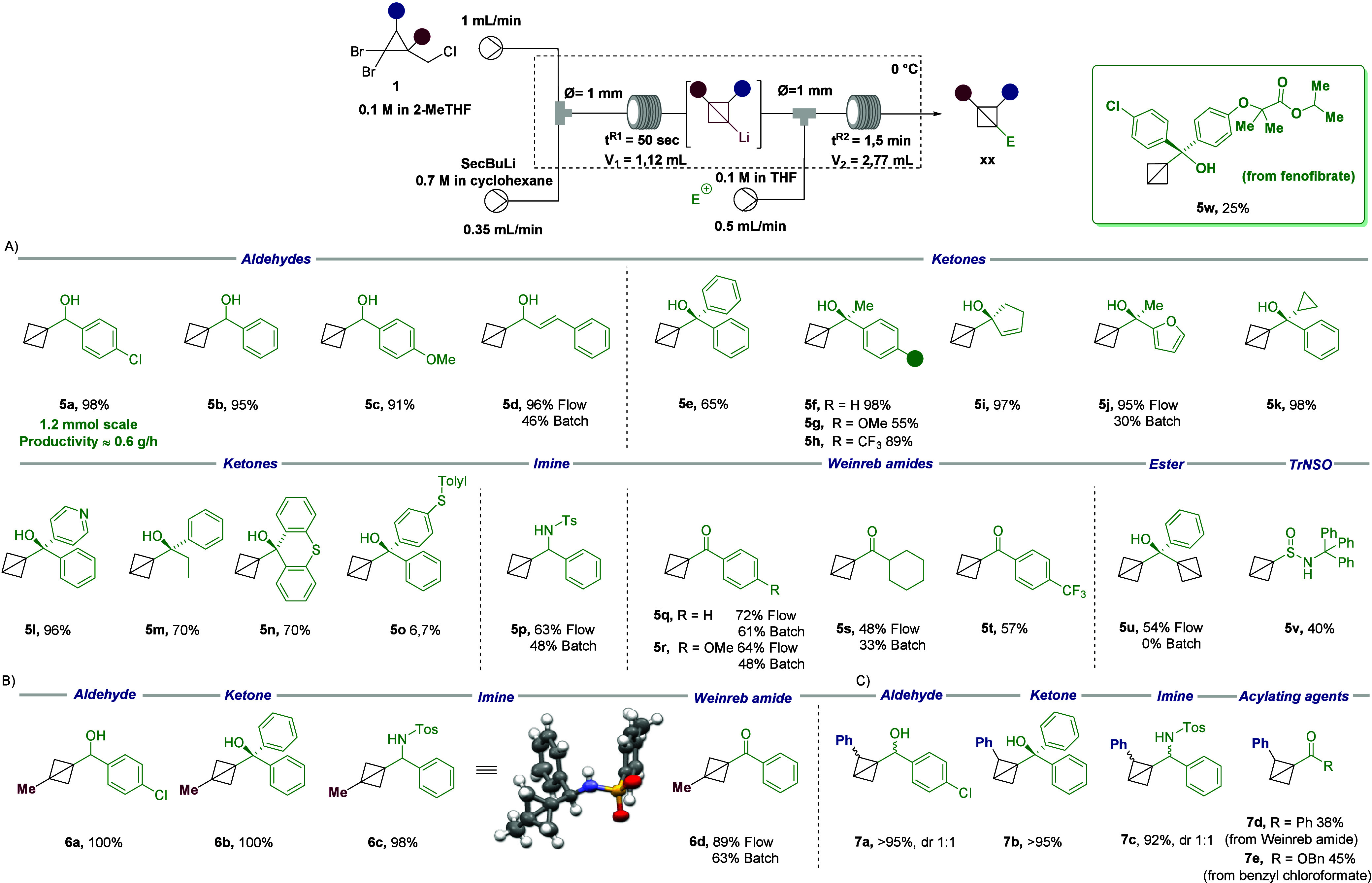
Scope of the Continuous Flow Generation and Electrophilic Trapping of BCB-Li Yields were determined by ^1^H nuclear magnetic resonance (NMR) spectroscopy using dibromomethane as an internal standard.

Interestingly, the use of substituted dibromocyclopropane **1b** provided C3 methyl-substituted BCB under the optimized flow conditions (Scheme 2B). In fact, BCB derivatives **6a**–**6d** were obtained with excellent yields (from 89 up to 100%) regardless of the nature of the electrophile, aldehyde, ketone, Weinreb amide, or imine. Similarly, when subjecting dibromo cyclopropane **1c** (derived from cinnamyl chloride) to the lithiation/electrophilic trapping process using the developed flow protocol, a clean synthesis of C2-substituted BCBs **7a**–**7c** in yields up to >95% was achieved (Scheme 2C). The use of Weinreb amide and benzyl chloroformate as acylating agents yielded the corresponding BCBs **7d** and **7e** in 38 and 45% yields, respectively. It is worth pointing out that these results showcase the wide applicability of this flow setup to a large number of electrophiles and different BCB precursors. To enable a direct comparison, several reactions with representative electrophiles were executed under batch conditions at 0 °C (see the Supporting Information for details), observing consistently lower yields compared to flow (see compounds **5d**, **5j**, **5p**, **5q**–**5s**, **5u**, and **6d** in Scheme 2). Additionally, the robustness of the flow protocol was tested in a longer experiment using *p*-chlorobenzaldehyde as the electrophile (1.2 mmol scale; Scheme 2A), realizing a productivity of 0.6 g/h. The developed flow process was further benchmarked in the Aggarwal borylation–migration sequence of BCB-Li (Scheme 3). Starting from compound **1a** and using arylboronates **8a** and **8b**, corresponding BCB-BPin was generated under flow conditions in less than 3 min. A subsequent reaction under fed-batch conditions with benzaldehyde at −78 °C in the presence of tetrafluoroethylene (TFE) furnished 1,4-disubstituted cyclobutanes **9a** and **9b** with overall yields of 50 and 57%, respectively, over three formal synthetic steps in a one-flow operation. On the other hand, product **9a** could be obtained, under batch conditions, in a formal seven-step sequence (synthetic operations) with several temperature adjustments, resulting in an overall yield of 30% from compound **1a** (Scheme 3B). Remarkably, the flow process requires a significantly reduced reaction time (i.e., 7 h in batch versus 2 h in flow) while maintaining the same scale. To conclude our study and further emphasize the superior performance and sustainability of the flow process over batch processing, we conducted a systematic comparison to previously reported procedures for the synthesis of selected compounds (see the Supporting Information).

**Scheme 3:**
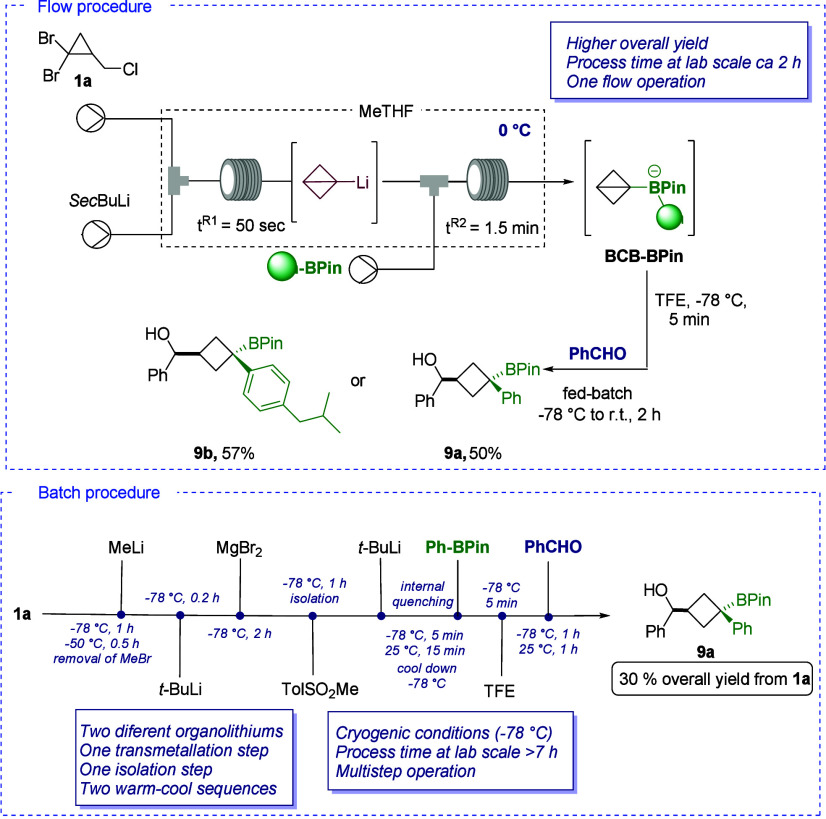
One-Flow Lithiation/Borylation/Electrophilic Trapping and Comparison between Batch and Flow Approaches

As shown in Figure 2 and based on literature data, we compared productivity and overall reaction time for the preparation of BCBs **5m**, **5p**, **5q**, **5s**, **5t**, and **9a** at the same scale in both batch and flow processes. Notably, the flow process consistently outperformed batch processing, resulting in significantly higher productivity and a substantial reduction in the reaction time.

**Figure 2:**
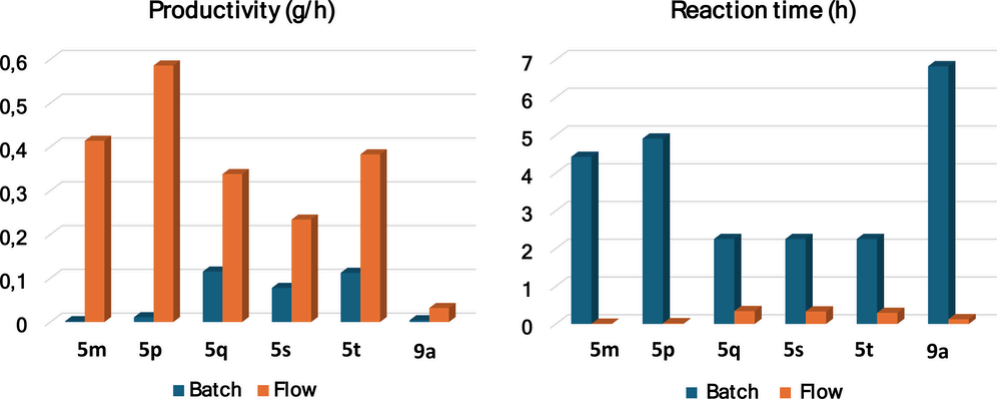
Batch/flow comparison of productivity (g/h) and reaction time (h) for the preparation of six BCB derivatives.

In conclusion, we developed the first flow-based preparation of BCB-Li, followed by electrophilic functionalization. The flow process demonstrates the safe and facile use of *sec*-BuLi at elevated temperatures (0 °C) as the sole organolithium species, which indicates clear advantages over reported batch procedures, offering higher yields, improved productivity, and shorter reaction times. Furthermore, we demonstrated that 2-methyltetrahydrofuran can be used as a greener alternative to tetrahydrofuran and diethyl ether, which are traditionally employed for BCB-Li generation. These advancements enable the streamlined synthesis of a variety of bicyclo[1.1.0]butyl derivatives and align well with the urgent need for new bioisosteric structural motifs in drug discovery and more sustainable synthetic technologies.

## Data Availability

The data underlying this study are available in the published article and its Supporting Information.

## Supporting Information Available

General procedures, characterization of compounds, crystallographic data, and NMR spectra (PDF)
FAIR data, including the primary NMR FID files, for compounds **3a**, **5a**–**5w**, **6a**–**6d**, **7a**–**7e**, **9a**, and **9b** (ZIP)
