# Supplementary material for: Generation and Use of Bicyclo[1.1.0]butyllithium under Continuous Flow Conditions
Source: Org Lett. 2025 Mar 20;27(13):3344–8. doi: 10.1021/acs.orglett.5c00705 (PMC11976866; doi:10.1021/acs.orglett.5c00705)
Supplement: Supplementary file 1 — ol5c00705_si_001.pdf [file ol5c00705_si_001.pdf]

Supplementary Information

Generation and Use of Bicyclo[1.1.0]butyllithium Under Continuous Flow Conditions

Elena Graziano,[a,b] Marco Colella,[a] Marcus Baumann,[b,*] Renzo Luisi[a,*]

[a] FLAME-Lab, Flow Chemistry and Microreactor Technology Laboratory, Department of Pharmacy-Drug Sciences, University of Bari "A. Moro", Via E. Orabona 4, 70125, Bari, Italy

[b] School of Chemistry, University College Dublin, O'Brien Centre for Science, Belfield, Dublin 4, Ireland

Email: renzo.luisi@uniba.it

Email: marcus.baumann@ucd.ie

Table of contents

1. General Information S3

2. General procedure S4

2.1General procedure 1 (GP1): Synthesis of 1,1-dibromo cyclopropanes S4

2.2 General procedure 2 (GP2): Synthesis of Weinreb amides S4

2.3 General procedure 3 (GP3): Synthesis of functionalized bicyclo [1.1.0] butanes using a batch reactor S5

2.4 General procedure 4 (GP4): Synthesis of functionalized bicyclo [1.1.0] butanes under flow conditions S5

2.5 General procedure 5 (GP5) for the preparation of cyclobutane boronic esters in flow S6

Long run experiment S7

3. Optimization study under flow conditions S8

4. Electrophile collection S9

6. Comparison Batch-Flow S10

7. Characterisation of compounds S12

8. Copies of NMR spectra S23

9. X-ray structrure of compound 6c S57

10. References S618

1. General Information

Unless otherwise stated, all solvents were purchased from Fisher Scientific and used without further purification. Also, unless otherwise stated, all substrates and reagents were purchased from Fluorochem, Sigma-Aldrich or TCI and used as received. ^{1}H NMR spectra were recorded on 400, 500 and 600 MHz instruments and are reported relative to the residual solvent: $CHCl_3$ (δ 7.26 ppm). ^{13}C{1H} NMR spectra were recorded on the same instruments (101, 125 and 150 MHz) and are reported relative to $CHCl_3$ (δ 77.0 ppm). ^{19}F-NMR spectra were recorded on a 400 MHz (376 MHz) spectrometer. Data for ^{1}H NMR are reported as follows: chemical shift (δ/ ppm) (integration, multiplicity, coupling constant (Hz)). Multiplicities are reported as follows: s = singlet, d = doublet, t = triplet, q = quartet, p = pentet, m = multiplet, br s = broad singlet, app = apparent. Data for ^{13}C{1H} NMR are reported in terms of chemical shift (δ/ppm) and multiplicity (C, CH, CH_2, or CH_3). Spin-spin coupling constants (J) are given in Hz. As far as possible, the assignment of all unambiguous resonances was performed by combined application of 1D and 2D NMR techniques, i.e. HSQC, COSY and HMBC experiments. ^{1}H NMR on the reaction crude was used to establish the diastereomeric ratio.

IR spectra were obtained by use of a Platinum spectrometer (near, ATR sampling, Bruker, Billercia, MA, USA) with intensities of the characteristic signals as reported as weak (w, <20% of tallest signal), medium (m, 21-70% of tallest signal) or strong (s, >71% of tallest signal). Thermoscientific Nicolet Summit PRO FTIR Spectrometer was employed to obtain the infrared spectra and samples were loaded neat, and absorption frequencies are recorded in cm-1.

High-resolution mass spectrometry was performed using the indicated techniques on a micromass LCT orthogonal time-of-flight mass spectrometer with leucine-enkephalin (Tyr-GlyPhe-Leu) as an internal lock mass. Agilent 6530 accurate mass Q-TOF instrument and Excalibur data system were used to record the high-resolution mass spectrometry (HRMS) spectra.

TLC was performed on Merck pre-coated Silica gel 60 F254 aluminium plates with realisation by UV irradiation at 254 nm or by oxidation with $KMnO_4$ stain. Column chromatography was performed with the "flash" methodology using Macherey-Nagel silica gel 60 M, with a particle range of 0.04 - 0.063 mm. Solvents employed as eluents and for all other routine operations, as well as all reagents used were purchased from commercial suppliers and employed without any further purification.

Flow set-up: PTFE tubes (0.8 mmID) (Bola) and T-connectors (PEEK, 1.0 mm through hole) (Idex) were utilized for the synthesis, lithiation and quenching steps. The flow microreactor system was immersed in a thermostat-bath to control the temperature. Solutions of the reaction components were introduced to the flow microreactor system using three Syringe Pump (Chemyx).

2. General procedure

2.1 General procedure 1 (GP1): Synthesis of 1,1-dibromo cyclopropanes

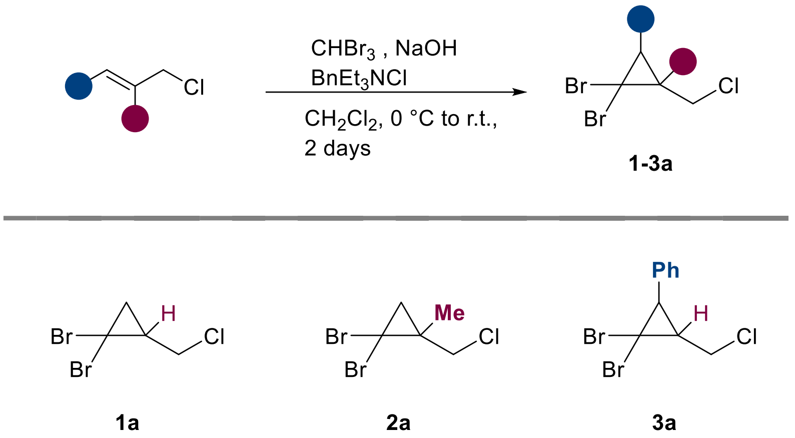

Note: Prepared according to a previously reported procedure.[1] Compounds **1a** and **2a** are known compounds. The obtained spectroscopic data matched with those previously reported.[1]

A 500 mL round bottom flask was fitted with a reflux condenser and an elliptical shaped stirrer bar under a nitrogen atmosphere. The flask was charged with sodium hydroxide powder (56.0 g, 1.40 mol, 8.75 equiv.) The reaction flask was cooled in an ice/water bath and CH_2Cl_2 (1.23 M, 130 mL) was added. The corresponding alkene (0.24 mmol, 1.5 equiv.), bromoform (14.0 mL, 0.16 mmol, 1.0 equiv.) and benzyltriethylammonium chloride (66 mg, catalytic) were added sequentially. The reaction was allowed to warm slowly to 40°C and stirring was maintained for 2 days. The reaction mixture was diluted with dicloromethane and was passed through a silica plug and the filtrate collected (!! Extra attention should be paid to avoid pouring the black precipitate over the filter !!). The filtrate was subsequently concentrated under reduced pressure to obtain the desired compounds, which were sufficiently pure for further synthetic steps (!! The compounds were kept under a nitrogen atmosphere in the freezer at – 20 °C and in the dark !!).

2.2 General procedure 2 (GP2): Synthesis of Weinreb amides

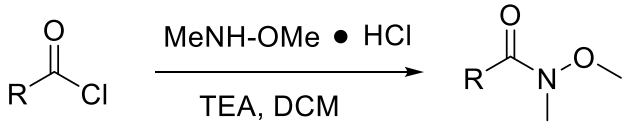

Notes: Following a modified literature procedure.[2]

To a solution of acyl chloride (5.86 mmol, 1.0 equiv.) in CH_2Cl_2 (17.7 mL, 0.33 M), N,O-dimethylhydroxylamine hydrochloride (571 mg, 5.86 mmol, 1.0 equiv.) was added at room temperature. Triethylamine (3.27 mL, 23.4 mmol, 4.0 equiv.) was added dropwise at 0 °C. The reaction mixture was stirred at room temperature overnight. It was diluted with CH_2Cl_2 (20 mL) and was washed with HCl 1 M (2 x 60 mL), sat. aq. $NaHCO_3$. (2 x 60 mL) and brine (2 x 60 mL). The combined organic phases were dried

over Na_2SO_4. The solvent was removed under reduced pressure to afford the desired Weinreb amide. Weinreb amides were synthesized according to this general procedure starting from the corresponding acyl chlorides.

2.3 General procedure 3 (GP3): Synthesis of functionalized bicyclo [1.1.0] butanes using a batch reactor

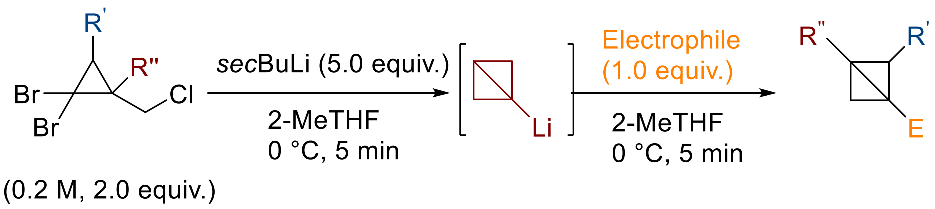

To a stirred solution of 1,1-dibromo cyclopropane (1.0 mmol, 2.0 equiv.) in dry 2-MeTHF (5 mL), under a nitrogen atmosphere, a solution of sec-buthyllithium (1.4 M in cyclohexane) (2.5 mmol, 5.0 equiv.) was added dropwise at 0 °C under stirring. After 5 minutes, the electrophile (0.5 mmol, 1.0 equiv) in dry 2-MeTHF (1 mL) was added and the resulting solution was stirred for 5 minutes at the same temperature. The reaction mixture was quenched with water (2 mL) and extracted with CH_2Cl_2 (3 x 6 mL). The combined organic phases were dried over Na_2SO_4 and evaporated under reduced pressure. When possible, the residue was purified using flash column chromatography yielding the desired product.

2.4 General procedure 4 (GP4): Synthesis of functionalized bicyclo [1.1.0] butanes under flow conditions

Note: Preparation of 0.7 M sec-buthyllithium solution (in cyclohexane)

To dry cyclohexane (3.0 mL) cooled to 0 °C was added dropwise commercial *sec*-buthyllithium solution (3.0 mL, 1.4 M in cyclohexane). The resulting sec-buthyllithium solution was kept at low temperature until use in the flow procedure.

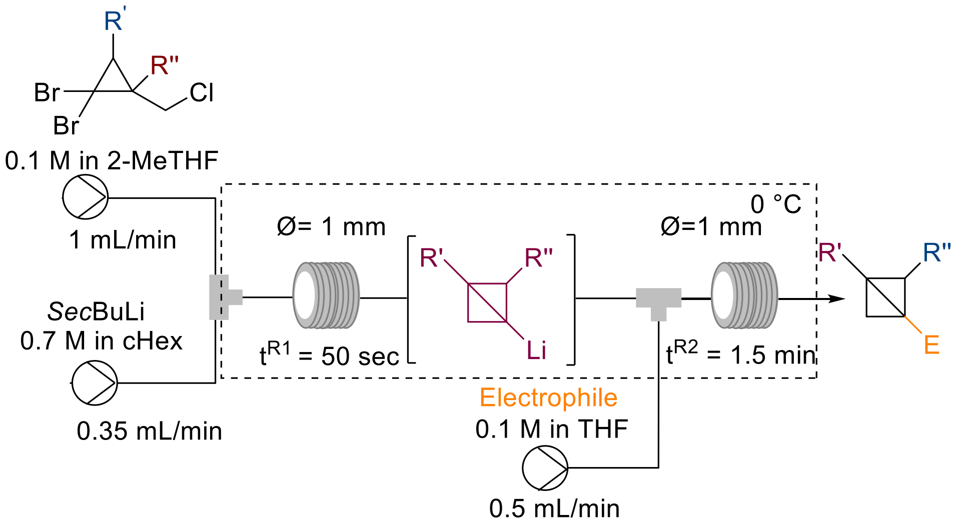

The microreactor system was fully immersed in a cooling bath (0 °C). A solution of 1,1-dibromo cyclopropane (0.1 M in 2-MeTHF) (flow rate: 1.0 mL/min) and a solution of sec-butyllithium (0.7 M in

cyclohexane) (flow rate: 0.35 mL/min), were introduced to M_1 (inner diameter = 1.0 mm) by syringe pumps. The resulting solution was passed through R_1 [ϕ1 = 0.8 mm, L1 = 224 cm (t^{R1} = 50 s)] and mixed in M_2 (inner diameter = 1.0 mm) with a solution of electrophile (0.1 M in 2-MeTHF) (flow rate: 0.5 mL/min). The resulting solution was passed through R_2 (ϕ2 = 0.8 mm, L2 = 554 cm (t^{R2} = 1 min and 30 sec)). After reaching the steady state, the solution was collected for 7 min and 40 sec in a separate vial containing an excess of water as the quench. The reaction mixture was extracted with diethyl ether (3 x 20 mL). The combined organic phases were dried over Na_2SO_4 and the solvent evaporated under reduced pressure. The residue was purified as described per example to yield the desired products.

2.5 General procedure 5 (GP5) for the preparation of cyclobutane boronic esters in flow

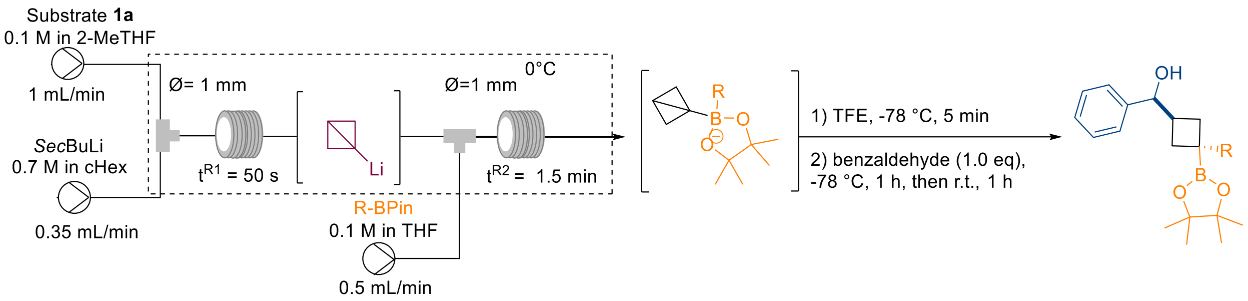

Bicyclo[1.1.0]butyllithium was quenched with a boronic ester according to **GP4**. After reaching steady state conditions, the solution obtained from the reactor output was collected in a round bottom flask before being cooled to –78 °C (dry ice/acetone) under a nitrogen atmosphere. Then 2,2,2-trifluoroethanol (0.1 mL) was added dropwise before stirring for a further 5 min. The aldehyde (0.38 mmol, 1.0 equiv) was then added and the mixture was left to stir for 1 h. The cooling bath was then removed, and the reaction was warmed to ambient temperature for 1 h. It was diluted with diethylether (10 mL) and was washed with sat.aq. NH_4Cl (2 x 10 mL). The combined organic phases were dried over Na_2SO_4, filtered and concentrated under reduced pressure. The crude residue was analyzed by 1H NMR to confirm the formation of the product and determine the diastereomeric ratio. The crude residue was then directly purified by flash column chromatography (SiO_2) to yield the desired cyclobutane.

Long run experiment

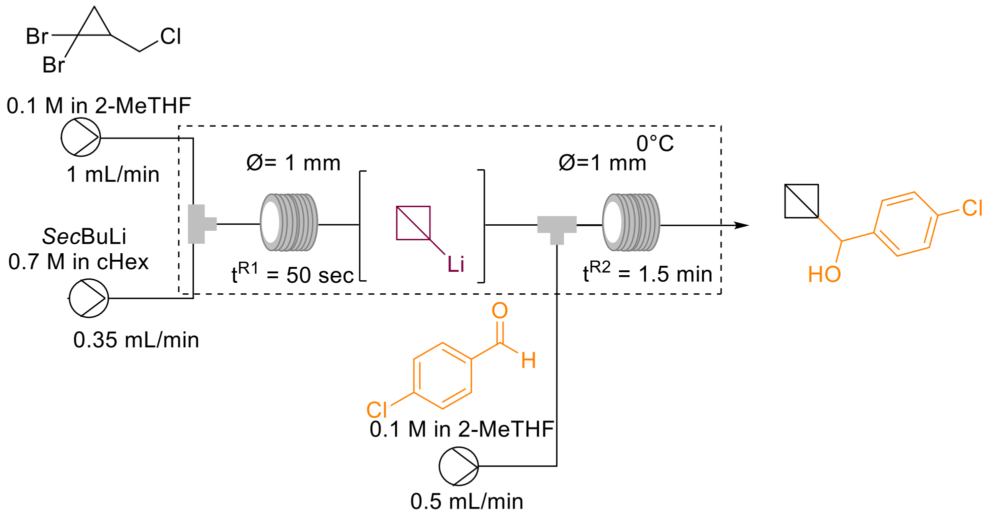

The long run experiment was performed utilizing the classic flow set-up (**GP4**). The microreactor system was fully immersed in a cooling bath (0 °C). A solution of 1,1-dibromo cyclopropane (0.1 M in 2-MeTHF) (flow rate: 1.0 mL/min) and a solution of sec-butyllithium (0.7 M in cyclohexane) (flow rate: 0.35 mL/min), were introduced to M_1 (inner diameter = 1.0 mm) by syringe pumps. The resulting solution was passed through R_1 [$\phi1$ = 0.8 mm, L1 = 224 cm (t_{R1} = 50 s)] and mixed in M_2 (inner diameter = 1.0 mm) with a solution of electrophile (0.1 M in 2-MeTHF) (flow rate: 0.5 mL/min). The resulting solution was passed through R_2 ($\phi2$ = 0.8 mm, L2 = 554 cm (t_{R2} = 1 min and 30 sec)). After reaching the steady state, the solution was collected for over 21 minutes and 40 sec in a separate vial containing an excess of water as the quench. The reaction mixture was extracted with diethyl ether (3 x 20 mL). The combined organic phases were dried over Na_2SO_4 and the solvent evaporated under reduced pressure. The crude residue was analysed by ^{1}H NMR to determine NMR yield of the desired product (**5a**, >95%).

3. Optimization study under flow conditions

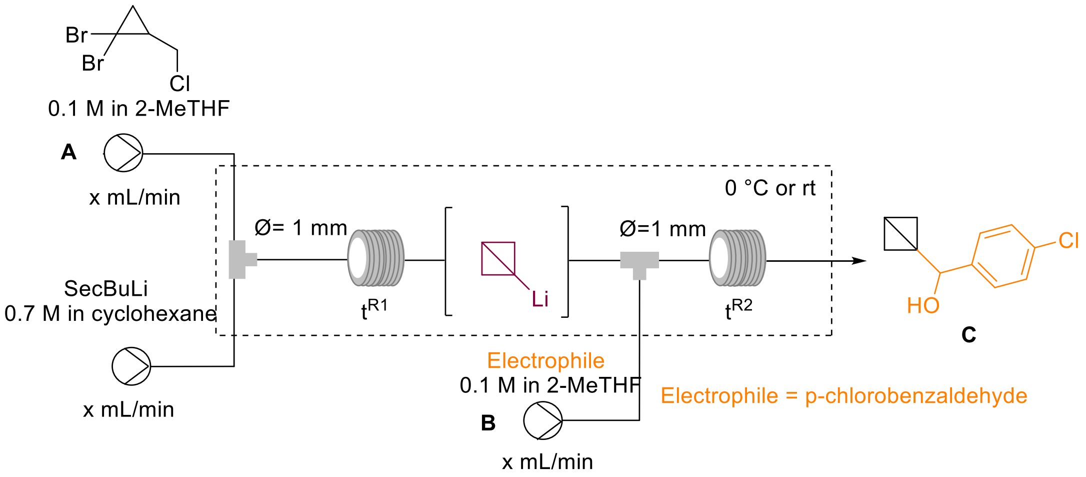

Entry	Substrate	Base	Electrophile	t^{R1}	t^{R2}	Conversion %	GC yield %
1	1 mL/min (2.0 equiv.)	0.35 mL/min (5.0 equiv.)	0.5 mL/min (1.0 equiv.)	15 sec	2.5 min	55 % A<br>24 % B<br>21 % C	16 %
2	1 mL/min (2.0 equiv.)	0.35 mL/min (5.0 equiv.)	0.5 mL/min (1.0 equiv.)	25 sec	2.5 min	35 % A<br>19 % B<br>46 % C	48 %
3	1 mL/min (2.0 equiv.)	0.35 mL/min (5.0 equiv.)	0.5 mL/min (1.0 equiv.)	30 sec	2.5 min	15 % C<br>85 % C	81 %
4	1 mL/min (2.0 equiv.)	0.35 mL/min (5.0 equiv.)	0.5 mL/min (1.0 equiv.)	40 sec	2.5 min	7 % B<br>93% C	89 %
5	1 mL/min (2.0 equiv.)	0.35 mL/min (5.0 equiv.)	0.5 mL/min (1.0 equiv.)	50 sec	2.5 min	5 % B<br>95 % C	92 % [A]
6	1 mL/min (2.0 equiv.)	0.35 mL/min (5.0 equiv.)	0.5 mL/min (1.0 equiv.)	1 min	2.5 min	39 % B<br>61 % C	55 %
7	1 mL/min (2.0 equiv.)	0.35 mL/min (5.0 equiv.)	0.5 mL/min (1.0 equiv.)	2 min	2.5 min	100 % B	4 %
8	1 mL/min (2.0 equiv.)	0.35 mL/min (5.0 equiv.)	0.5 mL/min (1.0 equiv.)	5 min	2.5 min	100 % B	3 %
9	1 mL/min (2.0 equiv.)	0.35 mL/min (5.0 equiv.)	0.5 mL/min (1.0 equiv.)	50 sec	1 min	2 % B<br>98 % C	93 %
10	1 mL/min (2.0 equiv.)	0.35 mL/min (5.0 equiv.)	0.5 mL/min (1.0 equiv.)	50 sec	1.5 min	>98 % C	95 % [B] [C]

[A] This reaction was repeated with different concentrations (0.2 M, 1.4 M, 0.2 M of substrate, sec-BuLi, and electrophile, respectively), but a clogging issue occurred. [B] This reaction was repeated at room temperature, resulting in degradation of BCB-Li. [C] This reaction was repeated using n-BuLi instead of sec-BuLi, resulting in product C with a 23% yield.

Notes: To evaluate the yield of the process, the output stream from the reactor was monitored by GC analysis. A calibration curve was built using dodecane as internal standard.

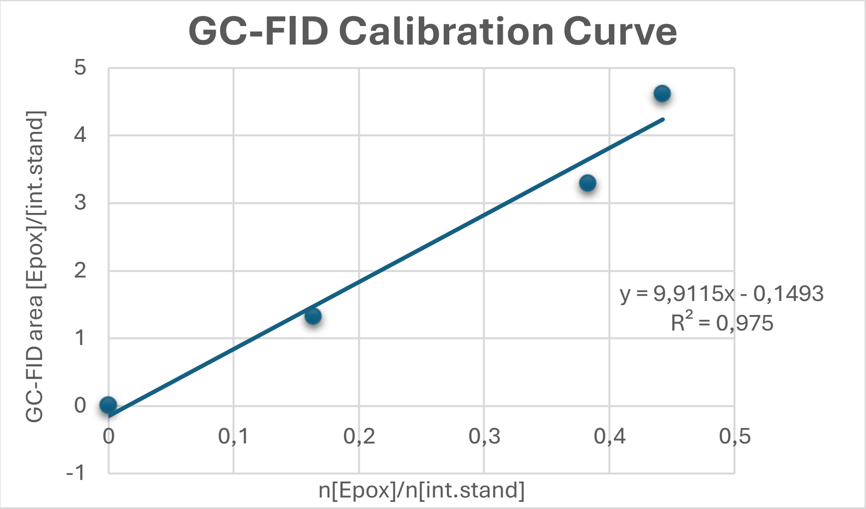

4. Electrophile collection

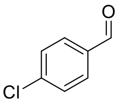

A

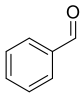

B

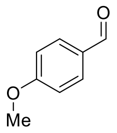

C

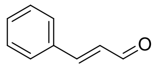

D

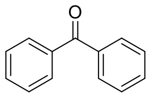

E

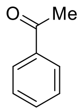

F

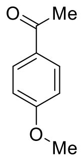

G

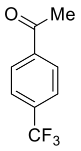

H

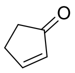

I

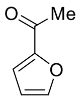

J

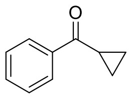

K

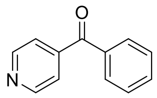

L

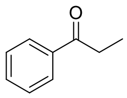

M

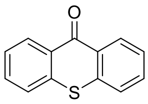

N

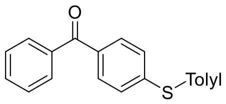

O

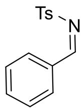

P

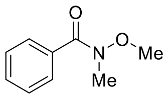

Q

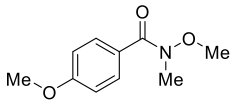

R

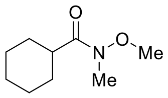

S

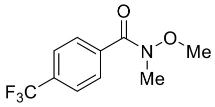

T

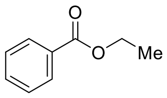

U

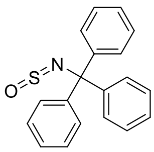

V

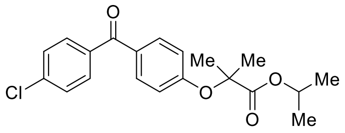

W

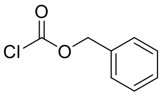

Z

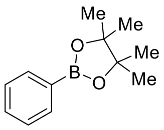

AA

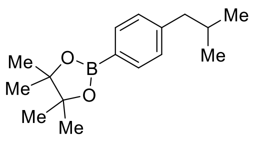

BB

Electrophiles **A-K**, **M-P**, **U-Z** are commercially available. Electrophile **L** is prepared according to a reported procedure.[3] Electrophiles **Q-T** were synthesized by adapting an established method.[2] The obtained spectroscopic data matched those previously reported **L**[3], **Q**[2], **R-T**[4].

6. Comparison Batch-Flow

Comparison of productivity and overall reaction time at the same scale between batch and flow protocol for selected six compounds (**5m**,[5] **5p**,[6] **5q**,[1] **5s**,[1] **5t**[1] and **9a**[6]). The mathematical formulas used are given and the numerical values obtained are expressed in a bar graph.

$$\textbf{Productivity} = \frac{Mass\ of\ desired\ product\ (g)}{Reaction\ time\ (h)}$$

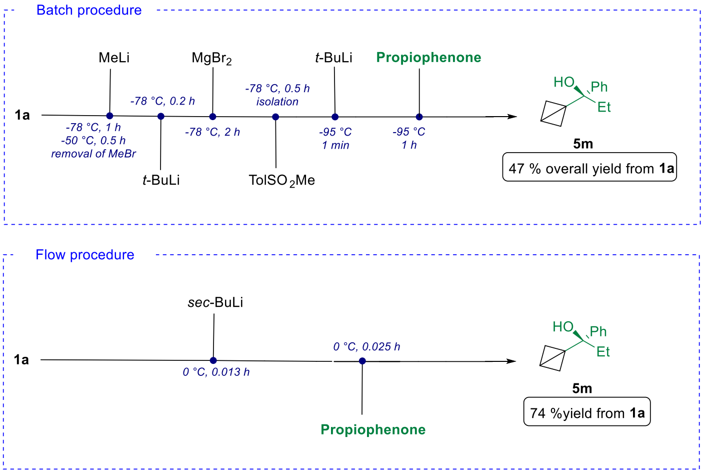

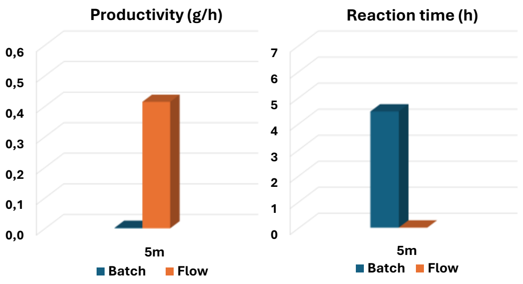

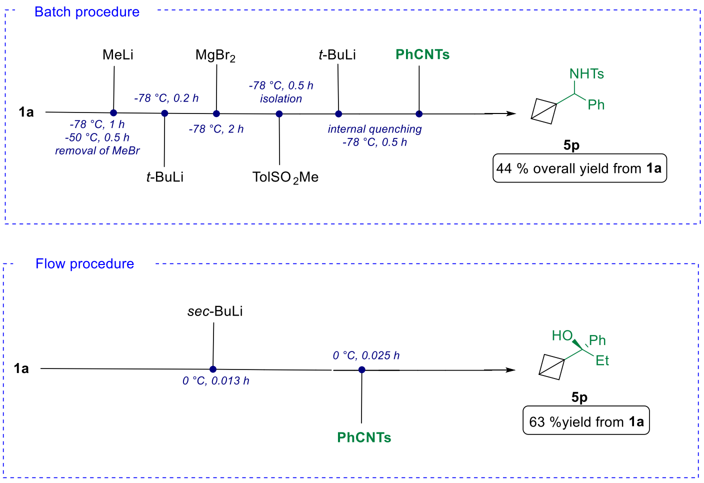

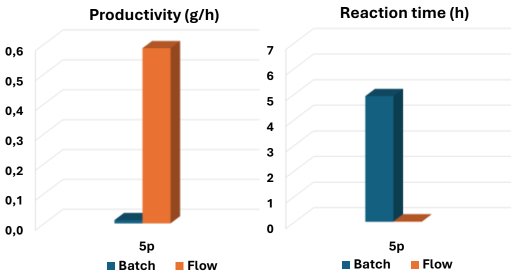

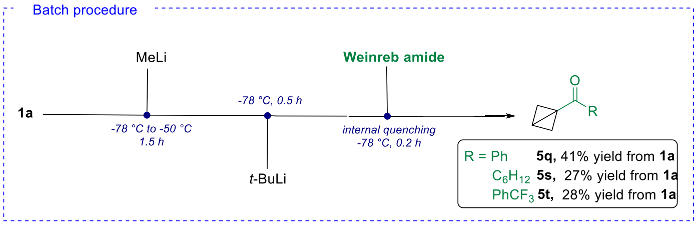
Batch procedure
MeLi
Weinreb amide
1a
-78 °C, 0.5 h
-78 °C to -50 °C
1.5 h
internal quenching
-78 °C, 0.2 h
t-BuLi
O
R
R = Ph 5q, 41% yield from 1a
C6H12 5s, 27% yield from 1a
PhCF3 5t, 28% yield from 1a

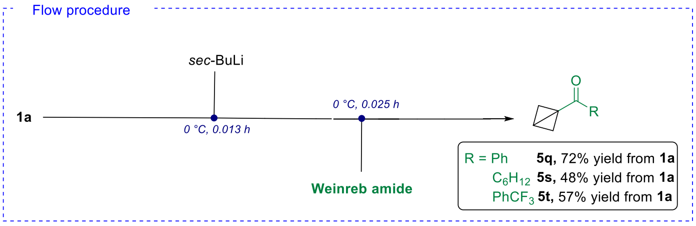
Flow procedure
sec-BuLi
1a
0 °C, 0.025 h
0 °C, 0.013 h
Weinreb amide
O
R
R = Ph 5q, 72% yield from 1a
C6H12 5s, 48% yield from 1a
PhCF3 5t, 57% yield from 1a

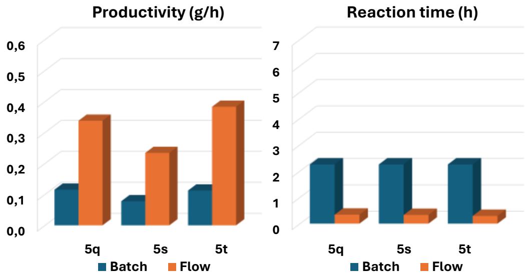
Productivity (g/h)
0,6
0,5
0,4
0,3
0,2
0,1
0,0
5q
5s
5t
Batch
Flow
Reaction time (h)
7
6
5
4
3
2
1
0
5q
5s
5t
Batch
Flow

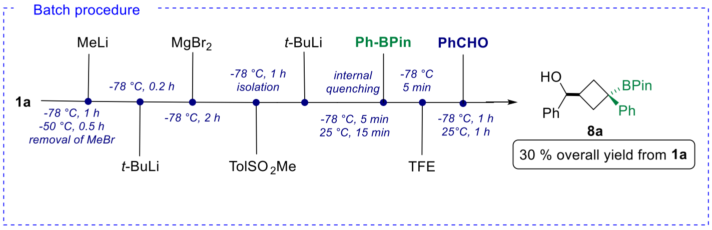
Batch procedure
MeLi
MgBr2
t-BuLi
Ph-BPin
PhCHO
1a
-78 °C, 0.2 h
-78 °C, 1 h
isolation
internal
quenching
-78 °C
5 min
-78 °C, 1 h
-50 °C, 0.5 h
removal of MeBr
-78 °C, 2 h
-78 °C, 5 min
25 °C, 15 min
-78 °C, 1 h
25°C, 1 h
t-BuLi
TolSO2Me
TFE
HO
Ph
BPin
Ph
8a
30 % overall yield from 1a

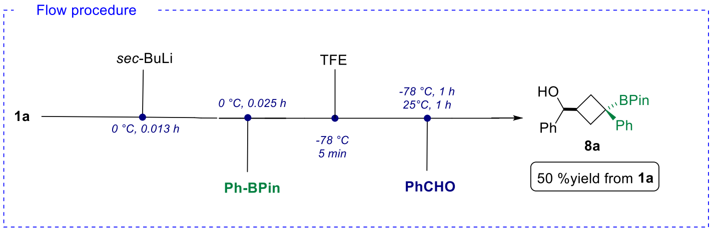
Flow procedure
sec-BuLi
TFE
1a
0 °C, 0.025 h
-78 °C, 1 h
25°C, 1 h
0 °C, 0.013 h
-78 °C
5 min
Ph-BPin
PhCHO
HO
Ph
BPin
Ph
8a
50 %yield from 1a

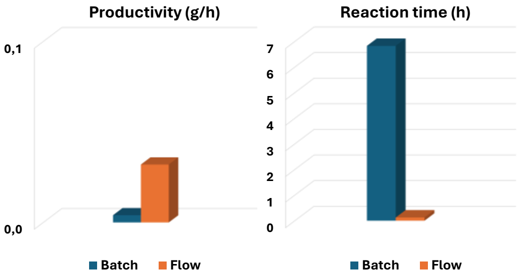
Productivity (g/h)
0,1
0,0
Batch
Flow
Reaction time (h)
7
6
5
4
3
2
1
0
Batch
Flow

7. Characterisation of compounds

(2,2-Dibromo-3-(chloromethyl)cyclopropyl)benzene 3a

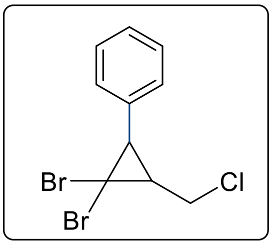

Prepared following **GP1**, a mixture of **3a** (89%) and alkene starting material was obtained as a brown oil after distillation. **^{1}H NMR** (400 MHz, $CDCl_3$) δ 7.40 – 7.29 (m, 5H, Ar-H), 3.90 – 3.81 (m, 2H, CH_2), 2.75 (t, *J* = 8.4 Hz, 1H, CH), 2.33 (td, *J* = 8.0, 7.2 Hz, 1H, CH). **^{13}C{^{1}H} NMR** (101 MHz, $CDCl_3$) δ 135.1 (Ar-C_q), 128.9 (2 x Ar-C), 128.6 (Ar-C), 128.1 (2 x Ar-C), 46.2 (CH_2), 42.1 (CH), 36.7 (CH), 34.2 (C_q). **HRMS** (ESI+) *m/z:* Calcd for $[M+Na]^+$ $C_{10}H_9Br_2Cl+Na^+$: 344.8657 Not found in HRMS analysis in either positive or negative ion mode. **IR (film, cm^{-1})**: vmax = 3028.2098, 1496, 1447, 1260, 1184, 1081, 1056, 1001, 963.

Bicyclo[1.1.0]butan-1-yl(4-chlorophenyl)methanol 5a

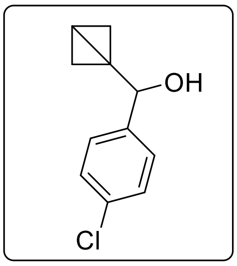

Prepared following **GP4** using *4-chlorobenzaldehyde* ***A***. Compound **5a** was obtained as a white powder (43 mg, 0.22 mmol, 56%) after flash column chromatography on silica gel R_f = 0.45 (7:3 cyclohexane/AcOEt*)*. **^{1}H NMR** (400 MHz, $CDCl_3$) δ 7.30 (d, *J = 1.2 Hz,* 4H, Ar-H), 5.05 (d, *J = 3.5 Hz,* 1H, OHC*H*), 2.27 (d, *J = 3.5Hz,* 1H, OH), 1.67 (dd, *J = 6.0, 3.2 Hz,* 1H, C*H*H), 1.37 – 1.33 (m, 2H, CH overlapping C*H*H), 0.78 (s, 1H, CH*H*), 0.66 (s, 1H, CH*H*). **^{13}C{^{1}H} NMR** (101 MHz, $CDCl_3$) δ 141.0 (Ar-C_q), 133.4 (Ar-C_q), 128.5 (2 x Ar-C), 127.7 (2 x Ar-C), 73.2 (OHCH), 32.6 (CH_2), 31.7 (CH_2), 15.2 (C_q), 1.3 (CH). **HRMS** (ESI+) *m/z:* $[M+Na]^+$ Calcd for $C_{11}H_{11}ClO+Na^+$: 217.0396; Found 217.0391. **IR (film, cm^{-1})**: vmax = 3354, 3053, 2959, 1723, 1590, 1264, 1115, 1091, 1013, 733, 702, 526.

Bicyclo[1.1.0]butan-1-yl(phenyl)methanol 5b

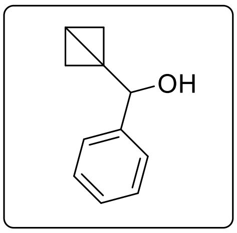

Prepared following **GP4** using *benzaldehyde* ***B***. A brown oil was obtained as crude product, which could not be purified via flash column chromatography due to instability of the product on SiO_2 (57 mg, 0.36 mmol, 95% NMR yield). **^{1}H NMR** (400 MHz, $CDCl_3$) δ 7.39 – 7.33 (m, 5H, Ar-H), 5.10 (d, *J = 3.6 Hz,* 1H, OHC*H*), 2.00 (d, *J = 3.6 Hz,* 1H, OH), 171 (dd, *J = 6.1, 3.1 Hz,* 1H, C*H*H), 1.39 – 1.35 (m, 2H, CH overlapping C*H*H), 0.80 (s, 1H, CH*H*), 0.68 (s, 1H, CH*H*). **^{13}C{^{1}H} NMR** (101 MHz, $CDCl_3$) δ 142.6 (Ar-C_q), 128.5 (2 x Ar-C), 127.8 (Ar-C), 126.4 (2 x Ar-C), 74.0 (OHCH), 32.7 (CH_2), 31.8 (CH_2), 15.2 (C_q), 1.2 (CH). **HRMS** (ESI+) *m/z:* $[M+Na]^+$ Calcd for $C_{11}H_{12}O+Na^+$: 183.0786; Found 183.0791.

Spectroscopic data are in agreement with those reported in literature.[7]

Bicyclo[1.1.0]butan-1-yl(4-methoxyphenyl)methanol 5c

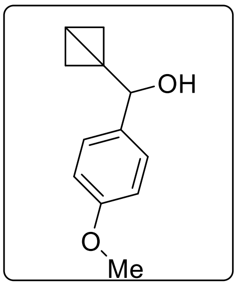

Prepared following **GP4** using *4-methoxybenzaldehyde* ***C***. Compound **5c** was obtained as a white powder (33 mg, 0.17 mmol, 44%) after flash column chromatography on silica gel R_f = 0.3 (8:2 cyclohexane/AcOEt*)*. **^{1}H NMR** (400 MHz, $CDCl_3$) δ 7.30 (d, *J = 8.7 Hz,* 2H, 2 x Ar-H), 6.88 (d, *J = 8.7 Hz,* 2H, 2 x Ar-H), 5.05 (d, *J = 3.5 Hz,* 1H, OHC*H*), 3.81 (s, 3H, CH_3), 1.92 (d, , *J = 3.5 Hz,* 1H, OH), 1.71 (dd, *J = 6.2, 2.9 Hz,* 1H, C*H*H), 1.40 (dd, *J = 6.2, 2.9 Hz,* 1H, C*H*H), 1.33 (t, *J = 3.1 Hz,* 1H, CH), 0.79 (s, 1H, CH*H*), 0.68 (s, 1H, CH*H*). **^{13}C{^{1}H} NMR** (101 MHz, $CDCl_3$) δ 159.2 (Ar-C_q), 134.9 (Ar-C_q), 127.6 (2 x Ar-C), 113.8 (2 x Ar-C), 73.6 (OHCH), 55.4 (OCH_3), 32.5 (CH_2), 31.8 (CH_2),

15.2 (C_q), 1.0 (CH). **HRMS** (ESI+) *m/z:* $[M+Na]^+$ Calcd for $C_{12}H_{14}O_2+Na^+$: 213.0891; Found 213.0886. **IR (film, cm^{-1})**: vmax = 3286, 3053, 1723, 1512, 1264, 895, 732, 701.

(E)-1-(Bicyclo[1.1.0]butan-1-yl)-3-phenylprop-2-en-1-ol 5d

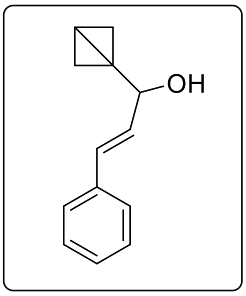

Prepared following **GP4** using *(E)-3-phenylprop-2-en-1-ol* ***D***. A brown oil was obtained as crude product, which could not be purified via flash column chromatography due to instability of the product on SiO_2 (68 mg, 0.36 mmol, 96% NMR yield). **^{1}H NMR** (400 MHz, $CDCl_3$) δ 7.39 – 7.30 (m, 5H, Ar-H), 6.63 (d, *J = 15.9 Hz,* 1H, CHC*H*=CH), 6.22 (dd, *J = 15.9, 6.4 Hz,* 1H, Ar-CH), 4.69 (bs, 1H, C*H*OH), 4.54 (s, 1H, OH), 1.73 – 1.66 (m, 2H, 2 x C*H*H), 1.44 – 1.41 (m, 1H, CH), 0.80 (s, 1H, CH*H*), 0.72 (s, 1H, CH*H*). **^{13}C{^{1}H} NMR** (101 MHz, $CDCl_3$) δ 136.6 (Ar-C_q), 131.0 (*C*H=CH), 129.7 (CH=*C*H), 128.7 (2 x Ar-C), 127.9 (Ar-C), 126.6 (2 x Ar-C), 72.4 (OHCH), 32.8 (CH_2), 31.5 (CH_2), 13.8 (C_q), 1.0 (CH). **HRMS** (ESI+) *m/z:* $[M+Na]^+$ Calcd for $C_{13}H_{14}O+Na^+$: 209.0942; Found 209.0942.

Bicyclo[1.1.0]butan-1-yldiphenylmethanol 5e

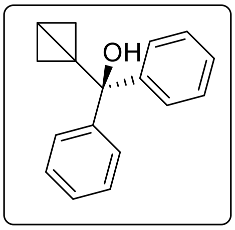

Prepared following **GP4** using *benzophenone* ***E***. Compound **5e** was obtained as a white solid (55 mg, 0.23 mmol, 57%) after flash column chromatography on silica gel R_f = 0.3 (9:1 cyclohexane/AcOEt*)*. **^{1}H NMR** (400 MHz, $CDCl_3$) δ 7.40 – 7.37 (m, 3H, Ar-H), 7.28 – 7.20 (m, 7H, Ar-H), 2.17 (s, 1H, OH), 1.52 – 1.50 (m, 1H, CH_2C*H*), 1.38 (d, *J = 2.8 Hz,* 2H, CH_2), 0.73 (d, *J = 2.8 Hz,* 2H, CH_2). **^{13}C{^{1}H} NMR** (101 MHz, $CDCl_3$) δ 146.3 (2 x Ar_q), 128.0 (4 x Ar-C), 127.4 (2 x Ar-C), 127.4 (4 x Ar-C), 77.5 (C_q), 32.3 (2 x CH_2), 19.1 (C_q), 1.1 (CH). **HRMS** (ESI+) *m/z:* $[M+Na]^+$ Calcd for $C_{17}H_{16}O+Na^+$: 259.1099; Found 259.1093. **IR (film, cm^{-1})**: vmax = 3450, 2959, 2925, 2872, 1447, 1264, 1012, 757, 736, 699.

1-(bicyclo[1.1.0]butan-1-yl)-1-phenylethan-1-ol 5f

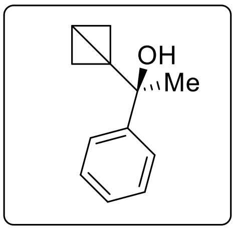

Prepared following **GP4** using *acetophenone* ***F***. A brown oil was obtained as crude product, which could not be purified via flash column chromatography due to instability of the product on SiO_2 (64 mg, 0.37 mmol, 98% NMR yield). **^{1}H NMR** (400 MHz, $CDCl_3$) δ 7.55 – 7.53 (m, 2H, Ar-H), 7.37 – 7.33 (m, 2H, Ar-H), 7.28 – 7.24 (m, 1H, Ar-H overlapping residual solvent), 1.91 (s, 1H, OH), 1.64 – 1.62 (m, 1H, CH_2C*H*), 1.59 (s, 3H, CH_3), 1.47 (dd, *J = 6.4, 2.9 Hz,* 1H, C*H*H), 1.43 (s,1H, C*H*H), 0.66 (d, *J = 1.3 Hz,* 1H, CH*H*), 0.60 (d, *J = 1.3 Hz,* 1H, CH*H*). **^{13}C{^{1}H} NMR** (101 MHz, $CDCl_3$) δ 147.2 (Ar_q), 128.2 (2 x Ar-C), 127.1 (Ar-C), 125.3 (2 x Ar-C), 73.1 (C_q), 31.4 (CH_2), 30.3 (CH_2), 28.7 (CH_3), 19.9 (C_q), 2.2 (CH). **HRMS** (ESI+) *m/z:* $[M+Na]^+$ Calcd for $C_{12}H_{14}O+Na^+$: 197.0942; Found 197.0929.

1-(Bicyclo[1.1.0]butan-1-yl)-1-(4-methoxyphenyl)ethan-1-ol 5g

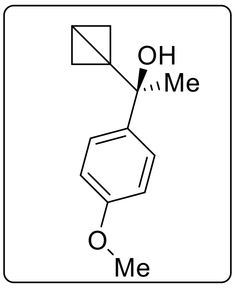

Prepared following **GP4** using 1-(4-methoxyphenyl)ethan-1-one ***G***. A brown oil was obtained as crude product, which could not be purified via flash column chromatography due to instability of the product on SiO_2 (42 mg, 0.21 mmol, 55% NMR yield). **^{1}H NMR** (400 MHz, $CDCl_3$) δ 7.45 (d, *J = 8.8 Hz,* 2H, Ar-H), 6.87 (d, *J = 8.9 Hz,* 2H, Ar-H), 3.80 (s, 3H, OCH_3), 1.89 (bs, 1H, OH), 1.61 (dd, *J* = *6.5, 2.9* Hz, 1H, C*H*H), 1.57 – 1.55 (m, 4H, CH_3 overlapping CH), 1.47 (dd, *J* = 6.5, 2.9 Hz, 1H, C*H*H), 0.64 (s, 1H, CH*H*), 0.59 (s, 1H, CH*H*). **^{13}C{^{1}H} NMR** (101 MHz, $CDCl_3$) δ 158.6

(Ar-C_q), 139.6 (Ar-C_q), 126.6 (2 x Ar-C), 113.5 (2 x Ar-C), 72.7 (C_q), 55.4 (OCH_3), 31.4 (CH_2), 30.3 (CH_2), 28.7 (CH_3), 19.4 (C_q), 2.1 (CH **HRMS** (ESI+) *m/z:* $[M+H]^+$ Calcd for $C_{13}H_{16}O_2+H^+$: 205.1229 Found 205.1223.

1-(Bicyclo[1.1.0]butan-1-yl)-1-(4-(trifluoromethyl)phenyl)ethan-1-ol 5h

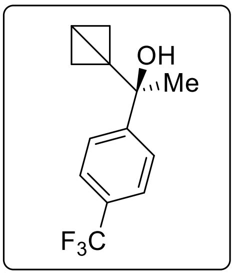

Prepared following **GP4** using *1-(4-(trifluoromethyl)phenyl)ethan-1-one* ***H***. A brown oil was obtained as crude product, which could not be purified via flash column chromatography due to instability of the product on SiO_2 (81 mg, 0.33 mmol, 89% NMR yield). **^{1}H NMR** (400 MHz, $CDCl_3$) δ 7.65 (d, *J = 8.2 Hz,* 2H, Ar-H), 7.59 (d, *J = 8.2 Hz,* 2H, Ar-H), 2.01 (bs, 1H, OH), 1.63 – 1.60 (m, 2H, CH overlapping C*H*H), 1.59 (s, 3H, CH_3), 1.47 (dd, *J = 6.4, 3.0 Hz,* 1H, C*H*H), 0.67 (s, 1H, CH*H*), 0.60 (s, 1H, CH*H*). **^{13}C{^{1}H} NMR** (101 MHz, $CDCl_3$) δ 151.2 (Ar_q), 129.2 (q, $^2J_{C-F}$ = 32.8 Hz, Ar_q), 125.8 (s, 2 x Ar-C), 125.1 (q, $^3J_{C-F}$ = 3.8 Hz, 2 x Ar-C), 121.6 (q, , $^1J_{C-F}$ = 271.9 Hz, Ar_q), 73.0 (C_q), 31.4 (CH_2), 30.2 (CH_2), 28.8 (CH_3), 19.4 (C_q), 2.6 (CH). **^{19}F NMR** (376.5MHz, $CDCl_3$) δ -62.32. **HRMS** (ESI+) *m/z:* $[M+Na]^+$ Calcd for $C_{13}H_{13}F_3O+Na^+$: 265.0816; Found 265.0817.

1-(Bicyclo[1.1.0]butan-1-yl)cyclopent-2-en-1-ol 5i

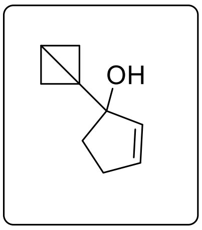

Prepared following **GP4** using *cyclopent-2-en-1-one* ***I***. A brown oil was obtained as crude product, which could not be purified via flash column chromatography due to instability of the product on SiO_2 (50 mg, 0.36 mmol, 97% NMR yield). **^{1}H NMR** (400 MHz, $CDCl_3$) δ 5.90 (dt, *J = 5.2, 2.4 Hz,* 1H, CC*H*=CH), 5.67 (dd, *J = 5.0, 2.8 Hz,* 1H, CCH=C*H*), 2.53 – 2.47 (m, 1H, CC*H*H-cyclopentyl), 2.32 – 2.27 (m, 1H, CCH*H*-cyclopentyl), 2.05 – 2.01 (m, 1H, CC*H*H-cyclopentyl), 1.98 – 1.95 (m, 1H, CCH*H*-cyclopentyl), 1.65 (s, 1H, C*H*H), 1.64 (s, 1H, OH), 1.43 (s, 1H, C*H*H), 1.35 (t, *J = 3.0 Hz,* 1H, CH), 0.61 (s, 2H, 2 x CH*H*). **^{13}C{^{1}H} NMR** (101 MHz, $CDCl_3$) δ 134.7 (CCH=CH), 134.1 (CCH=CH), 84.5 (Ar-C_q-cyclopentyl), 37.2 (CH_2-cyclopentyl), 31.5 (CH_2-cyclopentyl), 30.7 (CH_2), 30.4 (CH_2), 29.8 (Ar-C_q), 1.5 (CH). **HRMS** (ESI+) *m/z:* $[M+Na]^+$ Calcd for $C_{19}H_{12}O+Na^+$: 159.0786; Found 159.0783.

1-(Bicyclo[1.1.0]butan-1-yl)-1-(furan-2-yl)ethan-1-ol 5j

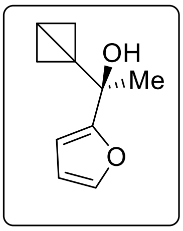

Prepared following **GP4** using *1-(furan-2-yl)ethan-1-one* ***J***. A brown oil was obtained as crude product, which could not be purified via flash column chromatography due to instability of the product on SiO_2 (59 mg, 0.36 mmol, 95% NMR yield). **^{1}H NMR** (400 MHz, $CDCl_3$) δ 7.34 – 7.33 (m, 1H, OCH), 6.31 (dd, *J = 3.3, 1.8 Hz,* 1H, C=C*H*), 6.26 (dd, *J = 3.3, 0.9 Hz,* 1H, C=CHC*H*), 2.18 (s, 1H, OH), 1.61 (s, 3H, CH_3), 1.60 (s, 1H, CH), 1.43 (s, 2H, 2 x C*H*H), 0.63 (s, 1H, CH*H*), 0.61 (s, 1H, CH*H*). **^{13}C{^{1}H} NMR** (101 MHz, $CDCl_3$) δ 158.9 (C_q-furanyl), 141.7 (CH-furanyl), 110.1 (CH-furanyl), 105.2 (CH-furanyl), 70.2 (C_q), 31.0 (CH_2), 30.8 (CH_2), 25.8 (CH_3), 17.8 (C_q), 2.2 (CH). **HRMS** (ESI+) *m/z:* $[M+Na]^+$ Calcd for $C_{10}H_{12}O_2+Na^+$: 187.0735; Not found in HRMS analysis in either positive or negative ion mode.

Bicyclo[1.1.0]butan-1-yl(cyclopropyl)(phenyl)methanol 5k

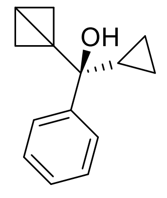

Prepared following **GP4** using *cyclopropyl(phenyl)methanone **K***. Compound **5k** was obtained as a white powder (64 mg, 0.32 mmol, 81%) after flash column chromatography on silica gel R_f = 0.35 (9:1 cyclohexane/AcOEt). **^{1}H NMR** (400 MHz, $CDCl_3$) δ 7.59 – 7.56 (m, 2H, Ar-H), 7.34 – 7.30 (m, 2H, Ar-H), 7.26 – 7.22 (m, 1H, Ar-H), 1.74 (dd, *J = 6.4, 2.9 Hz,* 1H, C*H*H), 1.66 (s, 1H, OH), 1.48 (t, *J = 2.9 Hz,* 1H, CH), 1.35 – 1.28 (m, 2H, C*H*H overlapping CC*H*CH_2), 0.71 – 0.65 (m, 1H, CHC*H*H), 0.64 (s, 1H, CH*H*), 0.63 – 0.52 (m, 2H, CHC*H*H overlapping CHCH*H*), 0,51 (s,1H, CH*H*), 0.45 – 0.38 (m, 1H, CHCH*H*). **^{13}C{^{1}H} NMR** (101 MHz, $CDCl_3$) δ 145.8 (Ar_q), 127.9 (2 x Ar-C), 127.1 (Ar-C), 126.1 (2 x Ar-C), 73.8 (C_q), 31.7 (CH_2), 31.0 (CH_2), 21.4 (C*C*HCH_2), 17.6 (Ar_q), 2.5 (CH_2-cyclopropyl), 0.3 (CH_2-cyclopropyl), 0.1 (CH). **HRMS** (ESI+) *m/z:* $[M+Na]^+$ Calcd for $C_{14}H_{16}O+Na^+$: 223.1099; Found 223.109 . **IR (film, cm^{-1})**: vmax = 3051, 3482, 3084, 3030, 3006, 2924, 1323, 1168, 1066, 993, 757, 700.

Bicyclo[1.1.0]butan-1-yl(phenyl)(pyridin-4-yl)methanol 5l

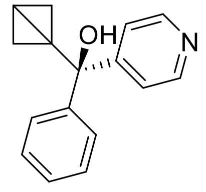

Prepared following **GP4** using phenyl(pyridin-4-yl)methanone ***L***. Compound **5l** was obtained as a yellow power (85 mg, 0.35 mmol, 91%) after flash column chromatography on silica gel R_f = 0.15 (5:5 cyclohexane/AcOEt*)*.**^{1}H NMR** (400 MHz, $CDCl_3$) δ 8.46 (d, *J* = 6.3 Hz, 2H, Ar-H), 7.48 – 7.45 (m, 2H, Ar-H), 7.36 – 7.29 (m, 5H, Ar-H), 2.97 (bs, 1H, OH), 1.60 (t, *J* = 2.9 Hz, 1H, CH), 1.56 (dd, *J* = 6.5, 2.9 Hz, 1H, C*H*H), 1.32 (dd, *J* = 6.5, 2.9 Hz, 1H, C*H*H), 0.81 (d, *J* = 5.1 Hz, 2H, 2 x CH*H*). **^{13}C{^{1}H} NMR** (101 MHz, $CDCl_3$) δ 154.6 (Ar-C_q), 149.4 (2 x Ar-C), 145.4 (Ar-C_q), 128.3 (2 x Ar-C), 128.0 (Ar-C), 127.3 (2 x Ar-C), 122.3 (2 x Ar-C), 76.8 (C_q), 32.3 (CH_2), 32.0 (CH_2), 18.6 (C_q), 1.4 (CH). **HRMS** (ESI-) *m/z:* $[M-H]^-$ Calcd for $C_{16}H_{15}NO-H^-$: 236.1075; Found 236.1082. **IR (film, cm^{-1})**: vmax = 3163, 3043, 2924, 1597, 1490, 1411, 1176, 1101, 1047, 975, 807, 761, 700.

(*R*)-1-(Bicyclo[1.1.0]butan-1-yl)-1-phenylpropan-1-ol 5m

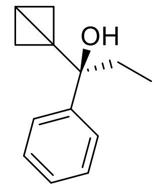

Prepared following **GP4** using propiophenone ***M***. A brown oil was obtained as crude product, which could not be purified via flash column chromatography due to instability of the product on SiO_2 (50 mg, 0.26 mmol, 70% NMR yield). **^{1}H NMR** (400 MHz, $CDCl_3$) δ 7.45 – 7.42 (m, 2H, Ar-H), 7.34 – 7.30 (m, 2H, Ar-H), 7.24 – 7.20 (m, 1H, Ar-H), 1.93 (qd, J = 7.4, 4.7 Hz, 2H), 1.60 – 1.58 (m, 1H, CH), 1.50 (dd, J = 2.8, 0.9 Hz, 2H), 0.81 (t, J = 7.4 Hz, 3H, CH_2C*H$_3$*), 0.60 (dd, J = 1.5, 0.7 Hz, 1H, C*H*H), 0.52 (dd, J = 1.5, 0.7 Hz, 1H, C*H*H). **HRMS** (ESI+) *m/z:* $[M+Na]^+$ Calcd for $C_{13}H_{16}O+Na^+$: 211.1099; Found 211.1030.

Spectroscopic data are in agreement with those reported in literature.[5]

9-(bicyclo[1.1.0]butan-1-yl)-9H-thioxanthen-9-ol 5n

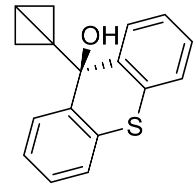

Prepared following **GP4** using *9H-thioxanthen-9-one* ***N*** Compound **5n** was obtained as a yellow powder (79 mg, 0.3 mmol, 60%) washing the crude with hexane.**^{1}H NMR** (400 MHz, $CDCl_3$) δ 7.84 (dd, *J* = 7.8, 1.7 Hz, 2H, Ar-H), 7.25 – 7.12 (m, 6H, Ar-H), 2.07 (s, 1H, OH), 1.50 – 1.48 (bt, 1H, CH), 0.99 (d, *J* = 2.9 Hz, 2H, CH_2), 0.38 (s, 2H, CH_2).**^{13}C{^{1}H} NMR** (101 MHz, $CDCl_3$) δ 138.1 (C_q), 130.0 (C_q), 127.4 (2 x Ar-C), 126.5 (2 x Ar-C), 126.3 (2 x Ar-C), 125.9 (2 x Ar-C), 73.7 (C_q), 31.3

(2 x CH_2), 19.3 (C_q), 3.2 (CH) . **HRMS** (ESI+) *m/z:* $[M+H]^+$ Calcd for $C_{17}H_{14}OS+H^+$: 267.0844; Found 267.0829. **IR (film, cm^{-1})**: vmax = 3381, 2957, 2923, 2871, 1459, 1376, 1039, 734, 631.

Note: the crude prodcut could not be purified via flash column chromatography due its instability on SiO_2.

Bicyclo[1.1.0]butan-1-yl(phenyl)(4-(p-tolylthio)phenyl)methanol 5o

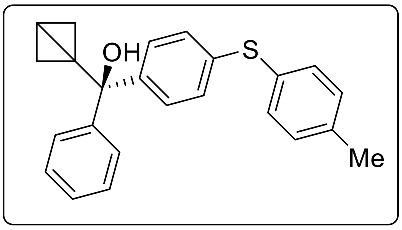

Prepared following **GP4** using phenyl(4-(p tolylthio)phenyl)methanone ***O***. Compound **5o** was obtained as a white powder (71 mg, 0.20 mmol, 52%) after flash column chromatography on silica gel R_f = 0.5 (9:1 cyclohexane/AcOEt*).* **^{1}H NMR** (500 MHz, $CDCl_3$) δ 7.37 – 7.35 (m, 2H, Ar-H), 7.25 – 7.19 (m, 7H, Ar-H), 7.13 – 7.06 (m, 4H, Ar-H), 2.28 (s, 3H, CH_3), 2.14 (bs, 1H, OH), 1.52 (s, 1H, CH_2CH), 1.35 (d, *J* = 2.4 Hz, 2H, CH_2), 0.70 (d, *J* = 2.8 Hz, 1H, CH_2). **$^{13}C\{^1H\}$ NMR** (125.75 MHz, $CDCl_3$) δ 146.0 (Ar_q), 144.4 (Ar_q), 138.0 (Ar_q), 136.5 (Ar_q), 134.7 (Ar_q), 132.8 (2 x Ar-C), 130.2 (2 x Ar-C), 128.8 (2 x Ar-C), 128.1 (2 x Ar-C), 128.0 (2 x Ar-C), 127.5 (Ar-C), 127.3 (2 x Ar-C), 77.3 (C_q), 32.3 (2 x CH_2), 21.3 (CH_3), 19.1 (C_q), 1.1 (CH). **HRMS** (ESI-) *m/z:* $[M-H]^-$ Calcd for $C_{24}H_{22}OS-H^-$: 357.1313; Found 357.1288. **IR (film, cm^{-1})**: vmax = 3474, 3024, 2919, 2850, 1671, 1586, 1489, 1397, 1014, 970, 808, 698, 659.

***N*-(Bicyclo[1.1.0]butan-1-yl(phenyl)methyl)-4-methylbenzenesulfonamide 5p**

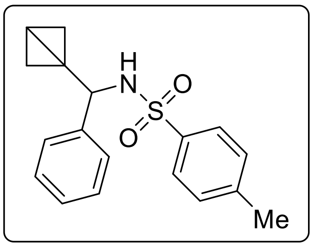

Prepared following **GP4** using *(Z)-N-benzylidene-4-methylbenzenesulfonamide* ***P***. Compound **5p** was obtained as a white powder (74 mg, 0.22 mmol, 59%) after flash column chromatography on silica gel R_f = 0.25 (8:2 cyclohexane/AcOEt*).* **^{1}H NMR** (400 MHz, $CDCl_3$) δ 7.62 (d, *J = 8.3 Hz,* 2H, Ar-H), 7.20 – 7.17 (m, 5H, Ar-H), 7.09 (dd, *J = 6.9, 2.8 Hz,* 2H, Ar-H), 5.08 (d, *J = 6.9 Hz,* 1H, NH), 4.76 (d, *J = 6.9 Hz,* 1H, NHC*H*), 2.39 (s, 3H, CH_3), 1.50 (dd, *J = 5.4, 3.7 Hz,* 1H, C*H*H), 1.27 – 1.26 (m, 2H, CH overlapping CH*H*), 0.62 (s, 1H, CH*H*), 0.54 (s, 1H, CH*H*). **$^{13}C\{^1H\}$ NMR** (101 MHz, $CDCl_3$) δ 143.4 (Ar-C_q), 139.6 (Ar-C_q), 138.0 (Ar-C_q), 129.5 (2 x Ar-C), 128.5 (2 x Ar-C), 127.7 (Ar-C), 127.3 (2 x Ar-C), 127.0 (2 x Ar-C), 58.1 (NHCH), 32.6 (CH_2), 31.7 (CH_2), 21.6 (CH_3), 14.3 (C_q), 1.8 (CH).

Spectroscopic data are in agreement with those reported in literature.[6]

Bicyclo[1.1.0]butan-1-yl(phenyl)methanone 5q

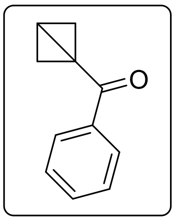

Prepared following **GP4** using *N-methoxy-N-methylbenzamide* ***Q***. Compound **5q** was obtained as a white powder (39 mg, 0.24 mmol, 62%) after flash column chromatography on silica gel R_f = 0.68 (5:1 pentane/ether*).* **^{1}H NMR** (600 MHz, $CDCl_3$) δ 7.87 – 7.85 (m, 2H, Ar-H), 7.54 – 7.51 (m, 1H, Ar-H), 7.44 – 7.42 (m, 2H, Ar-H), 2.62 (d, *J* = 3.5 Hz, 2H, CH_2), 2.21 (p, *J* = 3.4 Hz, 1H, CH), 1.48 (d, *J* = 3.2 Hz, 2H, CH_2). **$^{13}C\{^1H\}$ NMR** (150 MHz, $CDCl_3$) δ 200.1 (C=O), 137.9 (Ar-C_q), 132.3 (Ar-C), 128.9 (2 x Ar-C), 128.3 (2 x Ar-C), 38.0 (2 x CH_2), 21.6 (CH), 17.2 (C_q).

Spectroscopic data are in agreement with those reported in literature.[1]

Bicyclo[1.1.0]butan-1-yl(4-methoxyphenyl)methanone 5r

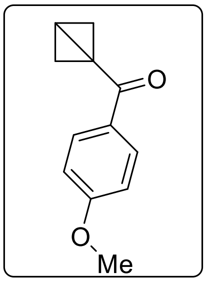

Prepared following **GP4** using *N,4-dimethoxy-N-methylbenzamide* ***R***. Compound **5r** was obtained as a courless oil (22 mg, 0.14 mmol, 35%) after flash column chromatography on silica gel R_f = 0.35 (1:1 PS 40-60/ether*)*. **^{1}H NMR** (500 MHz, $CDCl_3$) δ 7.89 (d, *J* = 8.7 Hz, 2H, Ar-H), 6.91 (d, *J* = 8.7 Hz, 2H, Ar-H), 3.85 (s, 3H, CH_3), 2.61 (d, *J* = 4.2 Hz, 2H, CH_2), 2.14 (p, *J* = 3.3 Hz, 1H, CH), 1.43 (d, *J* = 3.2 Hz, 2H, CH_2). **^{13}C{^{1}H} NMR** (125.75 MHz, $CDCl_3$) δ 198.3 (C=O), 163.0 (Ar-C_q), 131.1 (2 x Ar-C), 130.8 (Ar-C_q), 113.5 (2 x Ar-C), 55.5 (CH_3), 38.0 (2 x CH_2), 20.7 (CH).

Spectroscopic data are in agreement with those reported in literature.[8]

Bicyclo[1.1.0]butan-1-yl(cyclohexyl)methanone 5s

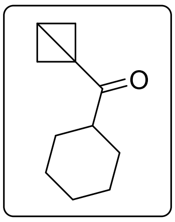

Prepared following **GP4** using *N-methoxy-N-methylcyclohexanecarboxamide* ***S***. Compound **5s** was obtained as a courless oil (22 mg, 0.14 mmol, 35%) after flash column chromatography on silica gel R_f = 0.65 (9:1 pentane/ether*)*. **^{1}H NMR** (400 MHz, $CDCl_3$) δ 2.48 (tt, *J* = 11.8, 3.4 Hz, 1H, COCH), 2.43 (d, *J* = 3.4 Hz, 2H, CH_2), 2.16 (p, *J* = 3.1 Hz, 1H, CH), 1.82 – 1.75 (m, 4H, cyclohexyl CH), 1.69 – 1.65 (m, 1H, cyclohexyl CH), 1.48 – 1.38 (m, 2H, cyclohexyl CH), 1.32 – 1.21 (m, 3H, cyclohexyl CH), 2.43 (d, *J* = 3.4 Hz, 2H, CH_2). **^{13}C{^{1}H} NMR** (101 MHz, $CDCl_3$) δ 210.1 (C=O), 46.8 (CO*C*H), 35.5 (2 x CH_2), 29.4 (cyclohexyl CH_2), 26.0 (cyclohexyl CH_2), 25.9 (cyclohexyl CH_2), 20.1 (CH), 17.6 (C_q).

Spectroscopic data are in agreement with those reported in literature.[1]

Bicyclo[1.1.0]butan-1-yl(4-(trifluoromethyl)phenyl)methanone 5t

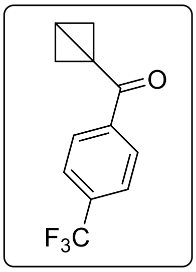

Prepared following **GP4** using *N-methoxy-N-methyl-4-(trifluoromethyl) benzamide* ***T***. Compound **5t** was obtained as a courless oil (38 mg, 0.16 mmol, 42%) after flash column chromatography on silica gel R_f = 0.5 (9:1 cHex/AcOEt*)*. **^{1}H NMR** (400 MHz, $CDCl_3$) δ 7.95 (d, *J* = 8.1 Hz, 2H, Ar-H), 7.70 (d, *J* = 8.2 Hz, 2H, Ar-H), 2.61 (dt, *J* = 3.7, 1.2 Hz, 2H, CH_2), 2.30 (p, *J* = 3.4 Hz, 1H, CH), 1.53 (dt, *J* = 3.2, 1.2 Hz, 2H, CH_2). **^{13}C{^{1}H} NMR** (101 MHz, $CDCl_3$) δ 199.0 (C=O), 140.8 (Ar-C_q), 133.6 (q, $^2J_{C-F}$ = 32.8 Hz, Ar_q), 129.1 (2 x Ar-C), 125.3 (q, $^3J_{C-F}$ = 3.8 Hz, 2 x Ar-C), 121.6 (q, $^1J_{C-F}$ = 271.9 Hz, CF_3), 38.2 (2 x CH_2), 22.7 (CH), 17.6 (C_q). **^{19}F NMR** (376.14 MHz, $CDCl_3$) δ -62.32 – -63.4 (m, 3F).

Spectroscopic data are in agreement with those reported in literature.[1]

Di(bicyclo[1.1.0]butan-1-yl)(phenyl)methanol 5u

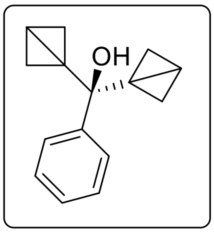

Prepared following **GP4** using *ethyl benzoate* ***U***. Compound **5u** was obtained as a white powder (34 mg, 0.16 mmol, 42%) after flash column chromatography on silica gel R_f = 0.5 (9:1 cyclohexane/AcOEt*)*. **^{1}H NMR** (600 MHz, $CDCl_3$) δ 7.58 – 7.56 (m, 2H, Ar-H), 7.35 – 7.32 (m, 3H, Ar-H), 1.85 (dd, *J* = 6.4, 2.8 Hz, 1H, 2 x C*H*H), 1.71 (s, H, OH), 1.61 (t, *J* = 2.9 Hz, 2H, 2 x CH), 1.33 (dd, *J* = 6.4, 2.8 Hz, 2H, 2 x C*H*H), 0.72 (s, 2H, CH*H*), 0.59 (s, 2H, CH*H*). **^{13}C{^{1}H} NMR** (150 MHz, $CDCl_3$) δ 144.6 (Ar-C_q), 128.0 (2 x Ar-C), 127.4 (Ar-C), 126.3 (2 x Ar-C), 73.3 (C_q), 31.7 (2 x CH_2), 31.5 (2 x CH_2), 18.9 (2 x C_q), 0.6 (2 x

CH). **HRMS** (ESI+) *m/z:* $[M+H]^+$ Calcd for $C_{15}H_{16}O+H^+$: 213.1279; Found 213.1274. **IR (film, cm^{-1})**: vmax = 3523, 3432, 3030, 2962,, 2925, 2874, 1638, 1323, 1163m 1130, 1065, 699.

N-tritylbicyclo[1.1.0]butane-1-sulfinamide 5v

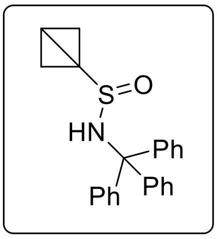

Prepared following **GP4** using *(tritylimino)-l4-sulfanone* **V**. Compound **5v** was obtained as a white powder (45 mg, 0.12 mmol, 33%) after flash column chromatography on silica gel R_f = 0.3 (7:3 hexane/AcOEt*).*[1]**H NMR** (400 MHz, $CDCl_3$) δ 7.36 – 7.33 (m, 5H, Ar-H), 7.30 – 7.23 (m, 10H, Ar-H), 5.23 (s, 1H, NH), 2.49 (ddd, *J* = 5.9, 3.6, 1.5 Hz, 1H, C*H*H), 1.97 (ddd, *J* = 5.9, 3.4, 1.7 Hz, 1H, C*H*H), 1.72 – 1.70 (m, 1H, CH), 1.18 (s, 1H, CH*H*), 1.12 (dd, *J* = 2.5, 1.3 Hz, 1H, CH*H*).**$^{13}C\{^1H\}$ NMR** (101 MHz, $CDCl_3$) δ 145.1 (3 x Ar-C_q), 129.3 (6 x Ar-C), 128.2 (6 x Ar-C), 127.5 (3 x Ar-C), 72.9 (NHC_q), 36.1 (CH_2), 32.5 (CH_2), 25.0 (C_q), 7.7 (CH). **HRMS** (ESI+) *m/z:* $[M+Na]^+$ Calcd for $C_{23}H_{21}NOS+Na^+$: 382.1242; Found 382.1238. **IR (film, cm^{-1})**: vmax = 3186, 3058, 2959, 1492, 1445, 1056, 1020, 910, 731, 699, 644.

Isopropyl2-(4-(bicyclo[1.1.0]butan-1-yl(4-chlorophenyl)(hydroxy)methyl)phenoxy)-2-methylpropanoate 5w

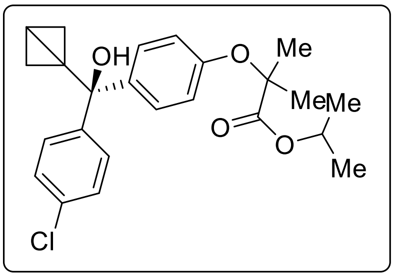

Prepared following **GP4** using *fenofibrate* **W**. Compound **5w** was obtained as a white powder (27 mg, 0.07 mmol, 15%) after flash column chromatography on silica gel R_f = 0.2 (9:1 cyclohexane/AcOEt*).*[1]**H NMR** (400 MHz, $CDCl_3$) δ 7.26 – 7.17 (m, 6H,Ar-H), 6.71 (d, *J* = 8.8 Hz, 2H, Ar-H), 5.00 (hept, *J* = 6.3 Hz, 1H, OC*H*(CH_3)$_2$), 2.07 (s,1H, OH), 1.51 (s, 6H, C*(CH_3)$_2$*), 1.45 (t, *J* = 2.9 Hz, 1H, CH), 1.39 (dd, *J* = 6.5, 2.9 Hz, 1H, C*H*H), 1.25 (dd, *J* = 6.5, 2.9 Hz, 1H, C*H*H), 1.13 (d, *J* = 6.3 Hz, 6H, CH*(CH_3)$_2$*), 0.68 (d, *J* = 6.0 Hz, 2H, 2 x CH*H*).**$^{13}C\{^1H\}$ NMR** (101 MHz, $CDCl_3$) δ 173.8 (C=O), 155.1 (Ar-C_q), 144.7 (Ar-C_q), 139.5 (Ar-C_q), 133.2 (Ar-C_q), 128.8 (2 x Ar-C), 128.2 (2 x Ar-C), 128.0 (2 x Ar-C), 118.3 (2 x Ar-C), 79.3 (*C*(CH_3)$_2$), 77.4 (C_q),69.1 (*C*H(CH_3)$_3$), 32.3 (CH_2), 32.2 (CH_2), 25.5 (2 x CH_3), 21.7 (2 x CH_3), 19.0 (C_q), 1.1 (CH). **HRMS** (ESI+) *m/z:* $[M+Na]^+$ Calcd for $C_{24}H_{27}ClO_4+Na^+$: 437.1496; Found 437.1500. **IR (film, cm^{-1})**: vmax = 3506, 2981, 2933, 1728, 1505, 1154, 1102, 830, 641.

(4-Chlorophenyl)(3-methylbicyclo[1.1.0]butan-1-yl)methanol 6a

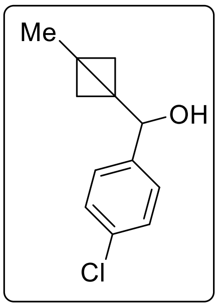

Prepared following **GP4** using *1,1-dibromo-2-(chloromethyl)-2-methylcyclopropane* ***2a*** *and 4-chlorobenzaldehyde* ***A***. Compound **6a** was obtained as a courless oil (81 mg, 0.39 mmol, 98%) without further purification.[1]**H NMR** (400 MHz, $CDCl_3$) δ 7.31 (s, 4H, Ar-H), 5.00 (d, *J* = 3.6 Hz, 1H, CH), 2.13 (d, *J* = 4.0 Hz, 1H, OH), 1.44 – 1.43 (m, 4H, CH_3 overlapping C*H*H), 1.09 (d, *J* = 6.6 Hz, 1H, C*H*H), 0.69 (s, 1H, CH*H*), 0.61(s, 1H, CH*H*). **$^{13}C\{^1H\}$ NMR** (101 MHz, $CDCl_3$) δ 141.7 (Ar-C_q), 133.4 (Ar-C_q), 128.5 (2 x Ar-C), 128.0 (2 x Ar-C), 72.9 (CH), 32.9 (CH_2), 32.7 (CH_2), 16.4 (C_q), 11.3 (CH_3), 11.1 (C_q). **HRMS** (ESI+) *m/z:* $[M+Na]^+$ Calcd for $C_{12}H_{13}ClO+Na^+$: 231.0553; Found 231.0563. **IR (film, cm^{-1})**: vmax = 3404, 2924, 1489, 1376, 1264, 1090, 1013, 703.

(3-Methylbicyclo[1.1.0]butan-1-yl)diphenylmethanol 6b

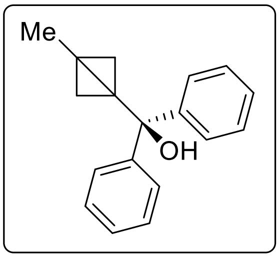

Prepared following **GP4** using *1,1-dibromo-2-(chloromethyl)-2-methylcyclopropane **2a** and benzophenone **E***. Compound **6b** was obtained as a courless oil (98 mg, 0.39 mmol, 98%) without further purification.**^{1}H NMR** (400 MHz, $CDCl_3$) δ 7.48 – 7.45 (m, 4H, Ar-H), 7.34 – 7.24 (m, 6H, Ar-H), 2.24 (s, 1H, OH), 1.49 (s, 3H, CH_3), 1.24 (s, 2H, CH_2), 0.69 (s, 2H, CH_2). **^{13}C{^{1}H} NMR** (101 MHz, $CDCl_3$) δ 146.9 (Ar-C_q), 127.9 (2 x Ar-C), 127.4 (Ar-C), 127.2 (2 x Ar-C), 78.5 (C_q), 32.5 (2 x CH_2), 20.1 (C_q), 12.4 (C_q), 10.9 (CH_3 **HRMS** (ESI+) *m/z:* $[M+Na]^+$ Calcd for $C_{18}H_{18}O+Na^+$: 273.1255; Found 273.1215. **IR (film, cm^{-1})**: vmax = 3588, 3389, 2923, 1447, 1264, 982, 699, 634.

4-Methyl-*N*-((3-methylbicyclo[1.1.0]butan-1-yl)(phenyl)methyl)benzenesulfonamide 6c

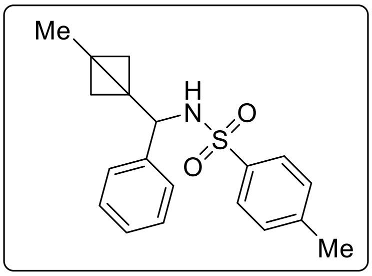

Prepared following **GP4** using *1,1-dibromo-2-(chloromethyl)-2-methylcyclopropane **2a** and (Z)-N-benzylidene-4-methylbenzenesulfonamide **P***. Compound **6c** was obtained as a yellow oil (100 mg, 0.32 mmol, 80%) washing the crude with a mixture of cHex/ether (8:2).**^{1}H NMR** (400 MHz, $CDCl_3$) δ 7.57 – 7.54 (m, 2H, Ar-H), 7.15 – 7.10 (m, 5H, Ar-H), 7.04 – 7.02 (m, 2H, Ar-H), 5.25 (d, *J* = 6.6 Hz, 1H, NH), 4.75 (d, *J* = 6.6 Hz, 1H, CH), 2.35 (s, 3H, Ar-CH_3) , 1.28 (s, 3H, CH_3), 1.19 (d, *J* = 6.6 Hz, 1H, C*H*H), 0.93 (d, *J* = 6.6 Hz, 1H, C*H*H), 0.52 (s, 1H, CH*H*), 0.42 (s, 1H, CH*H*). **^{13}C{^{1}H} NMR** (101 MHz, $CDCl_3$) δ 143.0 (Ar-C_q), 139.5 (Ar-C_q), 138.1 (Ar-C_q), 129.3 (2 x Ar-C), 128.3 (2 x Ar-C), 127.5 (Ar-C), 127.2 (4 x Ar-C), 57.9 (CH), 33.5 (CH_2), 32.2 (CH_2), 21.6 (CH_3), 16.0 (C_q), 11.9 (C_q), 10.8 (CH_3). **HRMS** (ESI+) *m/z:* $[M+Na]^+$ Calcd for $C_{19}H_{21}NO_2S+Na^+$: 350.1191; Found 350.1186. **IR (film, cm^{-1})**: vmax = 3280, 3058, 2957, 2925, 2856, 1599, 1454, 1325, 1264, 1158, 1093, 813, 700, 666.

(3-Methylbicyclo[1.1.0]butan-1-yl)(phenyl)methanone 6d

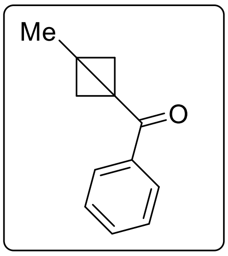

Prepared following **GP4** using *1,1-dibromo-2-(chloromethyl)-2-methylcyclopropane **2a** and N-methoxy-N-methylbenzamide* **Q**. Compound **6d** was obtained as a white powder (49 mg, 0.30 mmol, 76%) after flash column chromatography on silica gel R_f = 0.25 (9:1 pentane/ether*)*.**^{1}H NMR** (400 MHz, $CDCl_3$) δ 7.84 – 7.82 (m, 2H, Ar-H), 7.53 – 7.49 (m, 1H, Ar-H), 7.44 – 7.40 (m, 2H, Ar-H), 2.46 (s, 2H, CH_2), 1.57 (s, 2H, CH_2), 1.46 (s, 3H, CH_3). **^{13}C{^{1}H} NMR** (101 MHz, $CDCl_3$) δ 199.0 (C=O), 138.7 (Ar-C_q), 132.1 (Ar-C), 128.7 (2 Ar-C), 128.3 (2 x Ar-C), 41.1 (2 x CH_2), 33.9 (C_q), 21.5 (C_q), 12.8 (CH_3). **HRMS** (ESI+) *m/z:* $[M+Na]^+$ Calcd for $C_{12}H_{12}O+Na^+$: 195.0786; Found 195.0783. **IR (film, cm^{-1})**: vmax = 3485, 3056, 2956, 2924, 2855, 1633, 1576, 1447, 1373, 1102, 985, 776, 734, 570.

(4-Chlorophenyl)(2-phenylbicyclo[1.1.0]butan-1-yl)methanol 7a

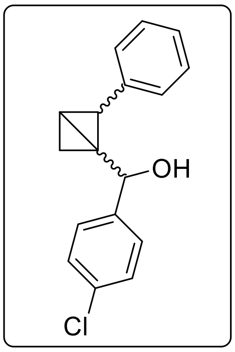

Prepared following **GP4** using *(2,2-Dibromo-3-(chloromethyl)cyclopropyl)benzene **3a** and 4-chlorobenzaldehyde **A***. Compound **7a** was obtained as a yellow powder (obtained as mixture of diastereomers, 88 mg, 0.32 mmol, 82%, *dr* ca 1:1) after flash column chromatography on silica gel R_f = 0.45 (8:2 cyclohexane /AcOEt*)*. **^{1}H NMR** (400 MHz, $CDCl_3$) δ 7.39 – 7.35 (m, 3H, Ar-H), 7.32 – 7.28 (m, 6H, Ar-H), 7.25 – 7.21 (m, 7H, Ar-H), 7.16 – 7.13 (m, 2H, Ar-H), 5.02 (d, *J = 3.5 Hz,* 1H, C*H*OH), 4.85 (d, *J = 4.4 Hz,* 1H, C*H*OH), 2.28 (d, *J = 2.9 Hz,* 1H, CH_2C*H*), 2.24 (d, *J = 1.0 Hz,* 1H, ArC*H*), 2.16 (d, *J = 1.3 Hz,* 1H, ArC*H*), 2.06 (d, *J = 3.7 Hz,* 1H, OH), 1.85 (d, *J = 3.0 Hz,* 1H, CH_2C*H*), 1.73 (d*, J = 2.9 Hz,* 1H, C*H*H), 1.71 (d, *J = 4.5 Hz,* 1H, OH), 1.04 (d, *J = 3.0 Hz,* 1H, C*H*H), 0.63 (d, *J = 4.5 Hz,* 2H, 2 x CH*H*). **^{13}C{^{1}H} NMR** (101 MHz, $CDCl_3$) δ 142.8 (Ar-C_q), 140.0 (Ar-C_q), 136.0 (Ar-C_q), 135.8 (Ar-C_q), 133.3 (Ar-C_q), 133.2 (Ar-C_q), 128.7 (2 x Ar-C), 128.6 (2 x Ar-C), 128.5 (2 x Ar-C), 128.5 (2 x Ar-C), 127.7 (2 x Ar-C), 127.6 (2 x Ar-C), 127.5 (2 x Ar-C), 127.2 (2 x Ar-C), 127.1 (Ar-C), 127.0 (Ar-C), 69.0 (CHOH), 67.3 (CHOH), 49.4 (Ar-CH), 48.7 (Ar-CH), 25.0 (CH_2), 24.3 (CH_2), 23.3 (C_q), 22.7 (C_q), 7.3 (CH), 6.7 (CH). **HRMS** (ESI+) *m/z:* [M+Na]$^+$ Calcd for $C_{17}H_{15}ClO+Na^+$: 293.0709; Not found in HRMS analysis in either positive or negative ion mode. **IR (film, cm^{-1})**: vmax = 3591, 3409, 3052, 3033, 1491, 1264, 1012, 732, 700.

Diphenyl(2-phenylbicyclo[1.1.0]butan-1-yl)methanol 7b

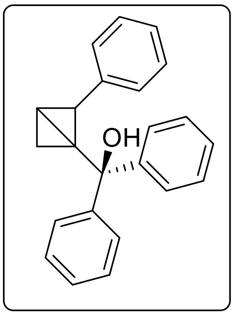

Prepared following **GP4** using *(2,2-Dibromo-3-(chloromethyl)cyclopropyl)benzene **3a** and benzophenone **E***. Compound **7b** was obtained as a yellow oil (109 mg, 0.348 mmol, 91%) after flash column chromatography on silica gel R_f = 0.65 (95:5 cyclohexane /AcOEt*)*. **^{1}H NMR** (400 MHz, $CDCl_3$) δ 7.64 – 7.62 (m, 2H, Ar-H), 7.39 – 7.31 (m, 3H, Ar-H), 7.24 – 7.14 (m, 10H, Ar-H), 2.55 (d, *J = 2.9 Hz*, 1H, CH_2C*H*), 2.23 (s, 1H, ArC*H*), 1.71 (s, 1H, OH), 0.93 (d, *J = 3.0 Hz*, 1H, C*H*H), 0.76 (d, *J = 1.3 Hz,* 1H, CH*H*). **^{13}C{^{1}H} NMR** (101 MHz, $CDCl_3$) δ 148.0 (Ar-C_q), 145.3 (Ar-C_q), 136.5 (Ar-C_q), 128.6 (2 x Ar-C), 128.5 (2 x Ar-C),128.0 (2 x Ar-C), 127.8 (2 x Ar-C), 127.7 (2 x Ar-C), 127.5 (Ar-C), 127.0 (2 x Ar-C), 126.8 (2 x Ar-C), 78.5 (OHC_q), 47.5 (Ar-CH),30.0 (CH_2), 26.7 (C_q), 5.0 (CH). **HRMS** (ESI+) *m/z:* [M+Na]$^+$ Calcd for $C_{23}H_{20}O+Na^+$: 335.1412; Found 335.1404. **IR (film, cm^{-1})**: vmax = 3564, 3059, 3029, 2961, 1638, 1600, 1492, 1447, 1163, 1065, 755, 699.

4-Methyl-*N*-(phenyl(2-phenylbicyclo[1.1.0]butan-1-yl)methyl)benzenesulfonamide 7c

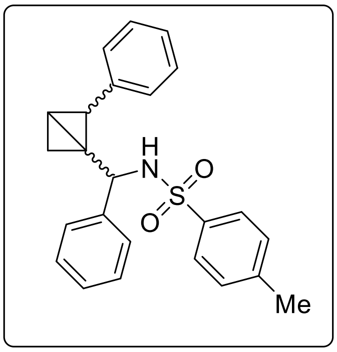

Prepared following **GP4** using *(2,2-Dibromo-3-(chloromethyl)cyclopropyl)benzene **3a*** and *(Z)-N-benzylidene-4-methylbenzenesulfonamide **P***. Compound **7c** was obtained as mixture of diastereomers (*dr* ca 1:1). The diastereomers were separated as a yellow oil (major: 54 mg, 0.14 mmol; minor: 54 mg, 0.14 mmol, 73%) after flash column chromatography on silica gel R_f = 0.5 (8:2 cyclohexane /AcOEt*)*. **First eluted diastereoisomer: ^{1}H NMR** (400 MHz, $CDCl_3$) δ 7.36 (d, J = 8.2 Hz, 2H, Ar-H), 7.26 – 7.16 (m, 8H, Ar-H), 7.02 – 6.99 (m, 4H, Ar-H), 5.08 (d, J = 4.9 Hz, 1H, NH), 4.61 (d, J = 5.0 Hz, 1H, NHC*H*), 2.36 (s, 3H, CH_3), 2.01 (s, 1H, Ar-C*H*), 1.74 (d, J = 3.0 Hz, 1H, CH), 1.05 (d, J = 3.0 Hz, 1H, C*H*H), 0.48 (s, 1H, CH*H*). **^{13}C{^{1}H} NMR** (101 MHz, $CDCl_3$) δ 142.9 (Ar-C_q), 138.8 (Ar-C_q), 137.2 (Ar-C_q), 135.4 (Ar-C_q), 129.4 (2 x Ar-C), 128.5 (2 x

Ar-C), 128.4 (2 x Ar-C), 127.6 (Ar-C), 127.4 (2 x Ar-C), 127.0 (2 x Ar-C),126.9 (Ar-C), 126.9 (2 x Ar-C), 53.8 (NHCH), 49.0 (Ar-*C*H), 24.9 (CH_2), 22.5 (C_q), 21.6 (CH_3), 7.4 (CH). **HRMS** (ESI+) *m/z:* $[M+Na]^+$ Calcd for $C_{24}H_{23}NO_2+Na^+$: 412.1347; Found 412.1338. **IR (film, cm^{-1})**: vmax = 3457, 3272, 3060, 3030, 2922, 1599, 1492, 1404, 1305, 1157, 1086, 1025, 731, 697. **Second eluted diastereoisomer: ^{1}H NMR** (400 MHz, $CDCl_3$) δ 7.65 (d, J = 8.3 Hz, 2H, Ar-H), 7.28 – 7.27 (m, 2H, Ar-H), 7.25 – 7.22 (m, 3H, Ar-H), 7.18 – 7.16 (m, 3H, Ar-H), 7.12 – 7.09 (m, 2H, Ar-H), 6.95 – 6.92 (m, 2H, Ar-H), 4.70 (d, J = 7.6 Hz, 1H, NH), 4.57 (d, *J = 7.6 Hz,* 1H, NHC*H*), 2.42 (s, 3H, CH_3), 2.20 (d, *J = 2.9 Hz*, 1H, CH), 2.05 (s, 1H, Ar-C*H*), 1.76 (d, *J = 3.0 Hz,* 1H, C*H*H), 0.68 (s, 1H, CH*H*). **^{13}C{^{1}H} NMR** (101 MHz, $CDCl_3$) δ 143.7 (Ar-C_q), 141.5 (Ar-C_q), 137.6 (Ar-C_q), 135.6 (Ar-C_q), 129.8 (2 x Ar-C), 128.7 (2 x Ar-C), 128.4 (2 x Ar-C), 127.8 (Ar-C), 127.5 (2 x Ar-C), 127.4 (2 x Ar-C),126.9 (Ar-C), 126.8 (2 x Ar-C), 52.0 (NHCH), 49.2 (Ar-*C*H), 24.3 (CH_2), 21.7 (CH_3), 21.5 (C_q), 7.1 (CH). **HRMS** (ESI+) *m/z:* $[M+Na]^+$ Calcd for $C_{24}H_{23}NO_2+Na^+$: 412.1347; Found 412.1338. **IR (film, cm^{-1})**: vmax = 3517, 3273, 3061, 3030, 2921, 1599, 1493, 1452, 1325, 1157, 1088, 910, 813, 697, 665.

Phenyl(2-phenylbicyclo[1.1.0]butan-1-yl)methanone 7d

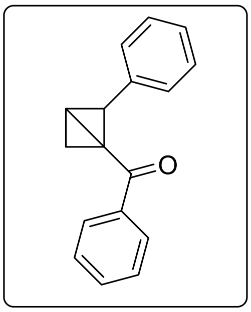

Prepared following **GP4** using *(2,2-Dibromo-3-(chloromethyl)cyclopropyl)benzene **3a** and N-methoxy-N-methylbenzamide* **Q**. Compound **7d** was obtained as a yellow oil (27 mg, 0.11 mmol, 30%) after flash column chromatography on silica gel R_f = 0.45 (9:1 hexane /AcOEt*).* **^{1}H NMR** (400 MHz, $CDCl_3$) δ 7.70 – 7.67 (m, 2H, Ar-H), 7.55 – 7.52 (m, 1H, Ar-H), 7.30 – 7.28 (m, 1H, Ar-H), 7.14 (t, *J* = 7.7 Hz, 2H), 7.06 – 6.99 (m, 2H, Ar-H), 6.97 – 6.94 (m, 2H, Ar-H), 2.78 – 2.76 (m, 1H, CH_2C*H*), 2.70 (d, *J* = 1.9 Hz, 1H, ArC*H*), 2.65 (d, *J* = 3.7 Hz, 1H, C*H*H), 1.40 (d, *J* = 2.8 Hz, 1H, CH*H*). **^{13}C{^{1}H} NMR** (101 MHz, $CDCl_3$) δ 198.8 (CO), 138.4 (Ar-C_q), 135.3 (Ar-C_q), 132.3 (Ar-C), 129.0 (2 x Ar-C), 128.1 (2 x Ar-C), 127.8 (2 x Ar-C), 127.2 (Ar-C), 127.0 (2 x Ar-C), 52.2 (Ar-CH), 34.6 (CH_2), 29.8 (C_q), 23.7 (CH). **HRMS** (ESI+) *m/z:* $[M+Na]^+$ Calcd for $C_{17}H_{14}O+Na^+$: 257.0942; Found 257.0943. **IR (film, cm^{-1})**: vmax =2965, 2878, 1722, 1642, 1449, 1384, 1224, 976, 765, 698.

Benzyl 2-phenylbicyclo[1.1.0]butane-1-carboxylate 7e

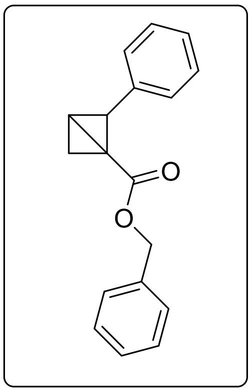

Prepared following **GP4** using *(2,2-Dibromo-3-(chloromethyl)cyclopropyl)benzene **3a** and benzyl carbonochloridate* **Z**. Compound **7e** was obtained as a white powder (38 mg, 0.14 mmol, 38%) after flash column chromatography on silica gel R_f = 0.2 (95:5 hexane /AcOEt*).* **^{1}H NMR** (400 MHz, $CDCl_3$) δ 7.27 – 7.16 (m, 6H, Ar-H), 7.17 (dd, *J* = 6.7, 2.9 Hz, 2H, Ar-H), 7.00 (dd, *J* = 6.5, 2.9 Hz, 2H, Ar-H), 5.08 (d, *J* = 12.6 Hz, 1H, OC*H*H), 4.98 (d, *J* = 12.6 Hz, 1H, OCH*H*), 2.80 (dt, *J* = 4.0, 2.1 Hz, 1H, CH_2C*H*), 2.44 (d, *J* = 1.9 Hz, 1H, ArC*H*), 2.34 (dd, *J* = 3.6, 1.1 Hz, 1H, C*H*H), 1.18 (s, 1H, CH*H*). **^{13}C{^{1}H} NMR** (101 MHz, $CDCl_3$) δ 171.5 (CO), 135.9 (Ar-C_q), 134.8 (Ar-C_q), 128.4 (2 x Ar-C), 128.2 (2 x Ar-C), 128.0 (3 x Ar-C), 127.5 (2 x Ar-C), 127.3 (Ar-C), 66.3 (OCH_2), 50.8 (Ar-CH), 31.4 (CH_2), 19.9 (CH), 15.8 (C_q). **HRMS** (ESI+) *m/z:* $[M+Na]^+$ Calcd for $C_{18}H_{16}O_2+Na^+$: 287.1048; Found 287.1057. **IR (film, cm^{-1})**: vmax =2959, 2926, 1708, 1498, 1404, 1190, 1140, 743, 695, 634.

(*R*)-Phenyl((1*r*,3*R*)-3-phenyl-3-(4,4,5,5-tetramethyl-1,3,2-dioxaborolan-2-yl)cyclobutyl)methanol 9a

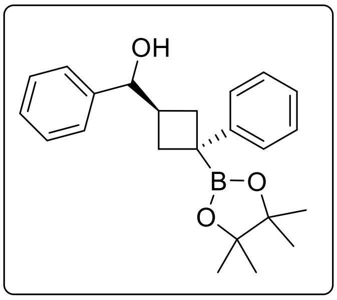

Prepared following **GP4** and **GP5** consecutively from bicyclo[1.1.0]butyl boronate complex of *phenylpinacol boronic ester* **AA** (0.38 mmol, 1.0 equiv.) (Electrophile for **GP5**: *benzaldehyde* (0.38 mmol, 1.0 equiv)). Compound **9a** was obtained as a colorless oil (62 mg, 0.17 mmol, 45%, d.r. > 95:5) after flash column chromatography on silica gel R_f = 0.16 (8:2 cyclohexane /diethyl ether*)*. **^{1}H NMR** (400 MHz, $CDCl_3$) δ 7.35 – 7.24 (m, 7H, Ar-H), 7.12 – 7.09 (m, 3H, Ar-H), 4.48 (d, J = 7.8 Hz, 1H, C*H*OH), 2.77 (ddd, J = 9.9, 7.5, 4.2 Hz, 1H, C*H*H), 2.66 – 2.57 (m, 1H, C*H*CHOH), 2.43 (ddd, J = 10.2, 7.7, 4.1 Hz, 1H, C*H*H), 2.26 (t, J = 9.8 Hz, 1H, CH*H*), 2.06 (t, J = 10.1 Hz, 1H, CH*H*), 1.80 (bs, 1H, OH), 1.15 (s, 12H, 4 x CH_3). **$^{13}C\{^1H\}$ NMR** (101 MHz, $CDCl_3$) δ 148.9 (Ar-C_q), 143.1 (Ar-C_q), 128.5 (2 x Ar-C), 128.2 (2 x Ar-C), 127.7 (Ar-C), 126.2 (2 x Ar-C), 125.7 (2 x Ar-C), 124.7 (Ar-C), 83.7 (2 x O*C*(CH_3)$_2$), 79.0 (CHOH), 39.0 (*C*HCHOH), 35.0 (CH_2), 34.4 (CH_2), 29.8 (C_q), 24.6 (2 x CH_3), 24.6 (2 x CH_3).

Spectroscopic data are in agreement with those reported in literature.[6]

(R)-((1r,3R)-3-(4-isobutylphenyl)-3-(4,4,5,5-tetramethyl-1,3,2-dioxaborolan-2-yl)cyclobutyl)(phenyl)methanol 9b

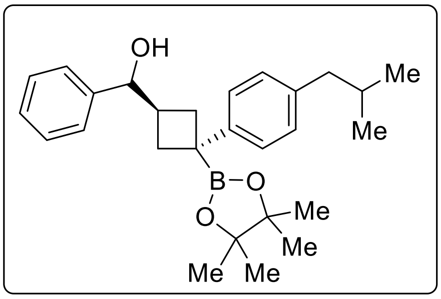

Prepared following **GP4** and **GP5** consecutively from bicyclo[1.1.0]butyl boronate complex of *2-(4-isobutylphenyl)-4,4,5,5-tetramethyl-1,3,2-dioxaborolane* **BB** (0.38 mmol, 1.0 equiv.) (Electrophile for **GP5**: *benzaldehyde* (0.38 mmol, 1.0 equiv)). Compound **9b** was obtained as a colorless oil (79 mg, 0.19 mmol, 50%, d.r. > 95:5) after flash column chromatography on silica gel R_f = 0.16 (6:4 cyclohexane /diethyl ether*)*. **^{1}H NMR** (500 MHz, $CDCl_3$) δ 7.28 – 7.18 (m, 5H, Ar-H), 6.97 – 6.92 (m, 4H, Ar-H), 4.42 (d, *J* = 7.9 Hz, 1H, C*H*OH), 2.68 (ddd, *J* = 10.0, 7.5, 4.2 Hz, 1H, C*H*H), 2.56 – 2.46 (m, 1H, C*H*CHOH), 2.36 – 2.30 (m, 3H, 2 x Ar-CH_2 overlapping 1 x C*H*H), 2.16 (t, *J* = 9.9 Hz, 1H, CH*H*), 1.96 (t, *J* = 10.1 Hz, 1H, CH*H*), 1.76 (dq, *J* = 13.6, 6.8 Hz, 1H, Ar-CH_2C*H*), 1.07 (s, 12H, 4 x CH_3), 0.82 (d, *J* = 6.6 Hz, 6H, 2 x CHC*H$_3$*). **$^{13}C\{^1H\}$ NMR** (125.75 MHz, $CDCl_3$) δ 145.8 (Ar-C_q), 143.1 (Ar-C_q), 137.9 (Ar-C_q), 128.9 (2 x Ar-C), 128.5 (2 x Ar-C), 127.6 (Ar-C), 126.2 (2 x Ar-C), 125.3 (2 x Ar-C), 83.6 (2 x O*C*(CH_3)$_2$), 79.1 (CHOH), 45.2 (Ar-CH_2), 39.0 (*C*HCHOH), 35.0 (CH_2), 34.4 (CH_2), 30.4 (Ar-CH_2CH), 29.8 (C_q), 24.6 (2 x CH_3), 24.5 (2 x CH_3), 22.6 (2 x CH_3). **HRMS** (ESI+) *m/z:* $[M+Na]^+$ Calcd for $C_{27}H_{37}BO_3+Na^+$: 443.2733; Found 443.2727. **IR (film, cm^{-1})**: vmax = 3393, 2954, 2928, 2867, 1682, 1604, 1454, 1308, 1260, 1020, 964, 798, 731, 699.

8. Copies of NMR spectra

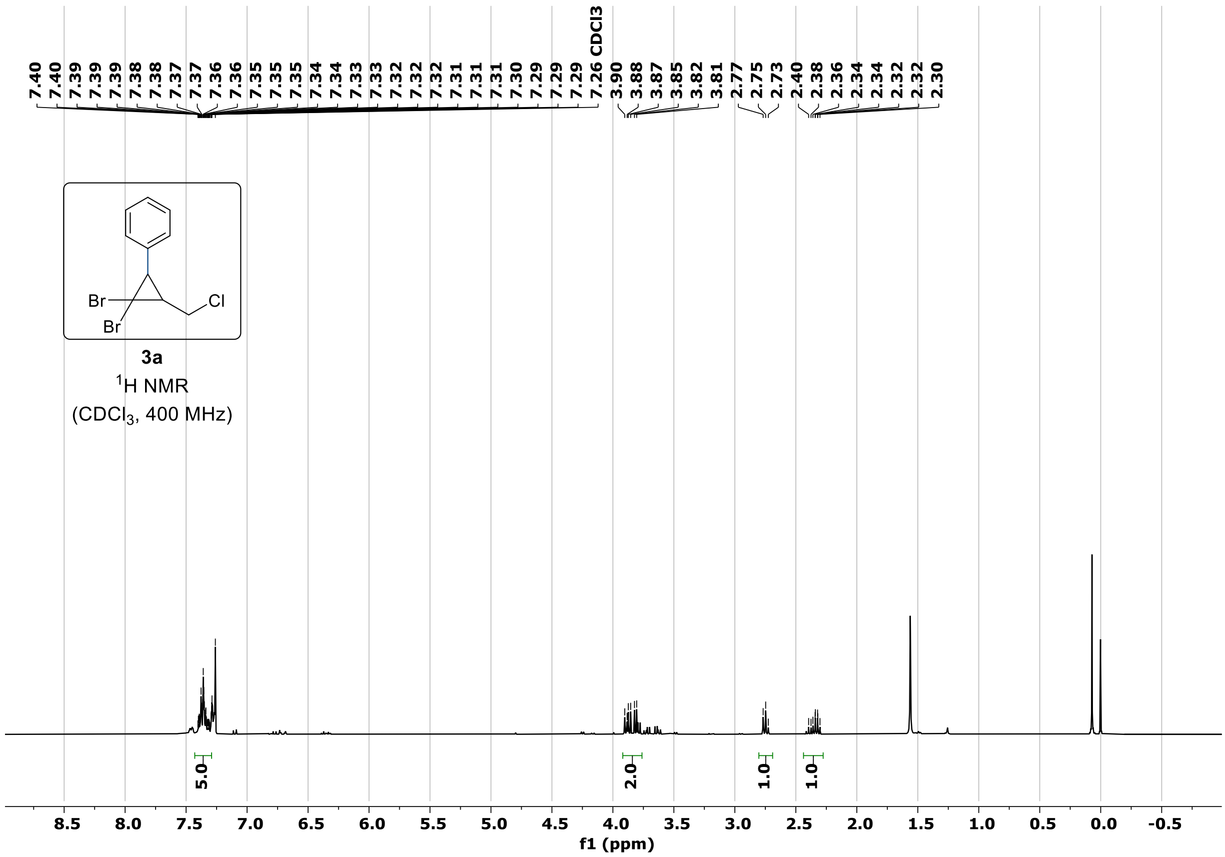

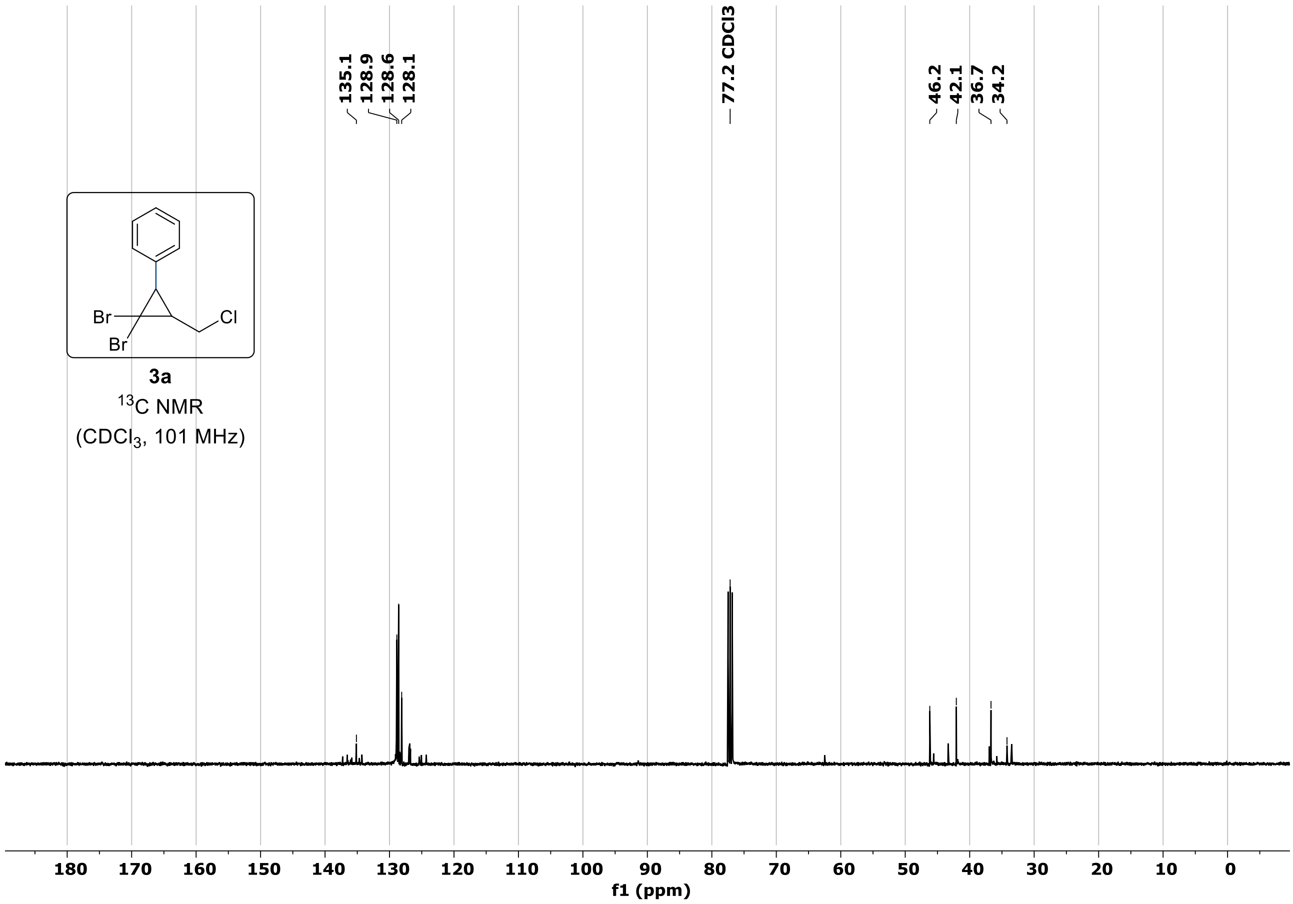

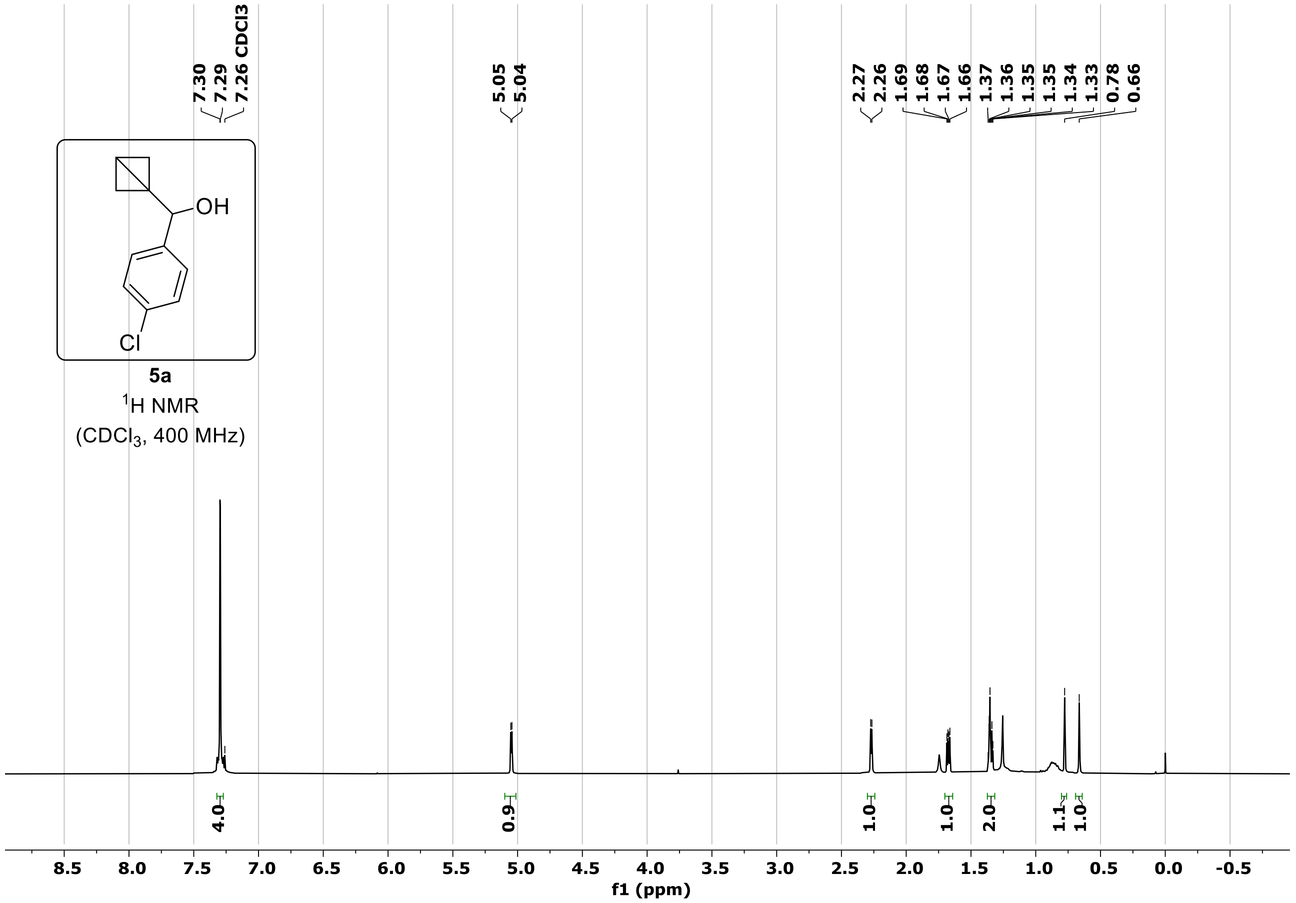
7.30
7.29
7.26 CDCl3
5.05
5.04
2.27
2.26
1.69
1.68
1.67
1.66
1.37
1.36
1.35
1.35
1.34
1.33
0.78
0.66
OH
Cl
5a
1H NMR
(CDCl3, 400 MHz)
4.0
0.9
1.0
1.0
2.0
1.1
1.0
f1 (ppm)

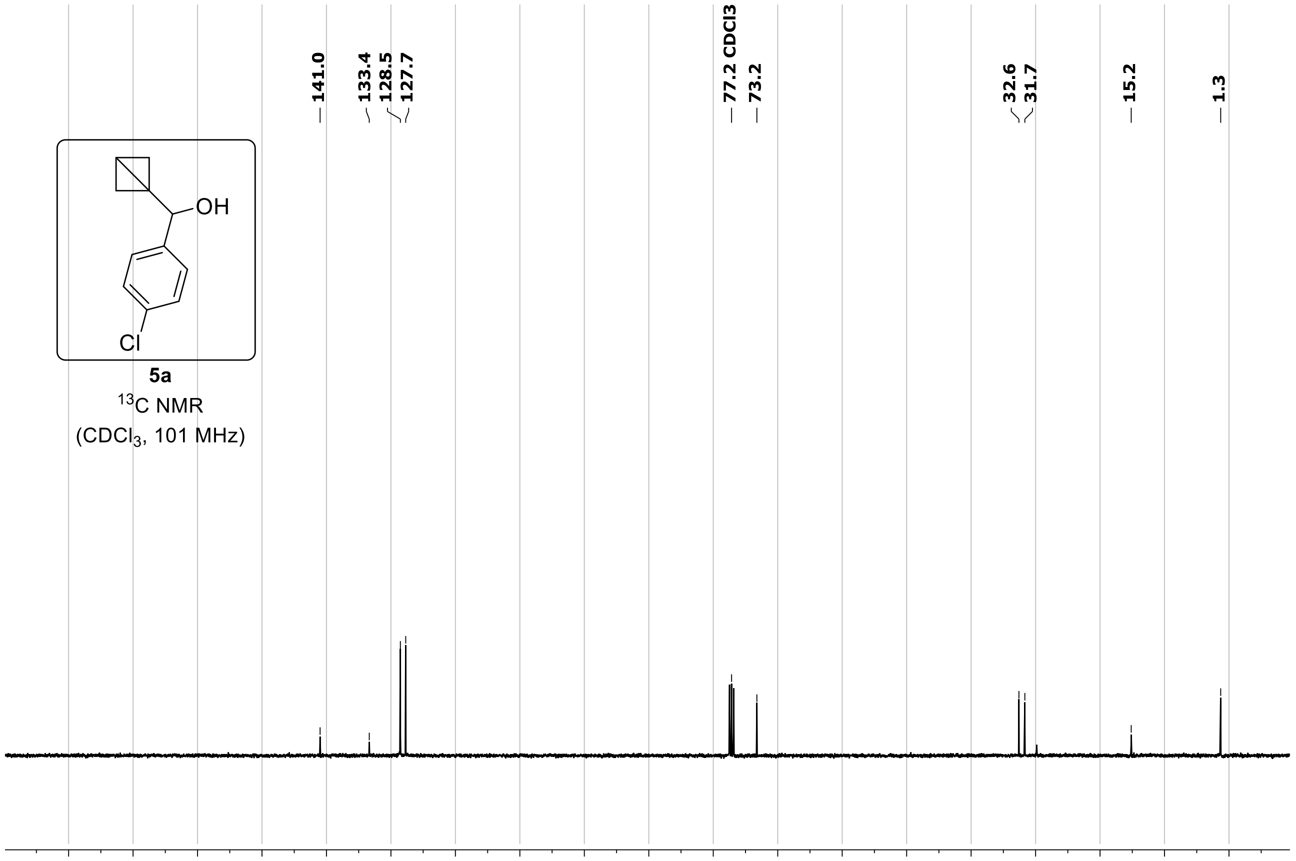
141.0
133.4
128.5
127.7
77.2 CDCl3
73.2
32.6
31.7
15.2
1.3
OH
Cl
5a
13C NMR
(CDCl3, 101 MHz)
f1 (ppm)

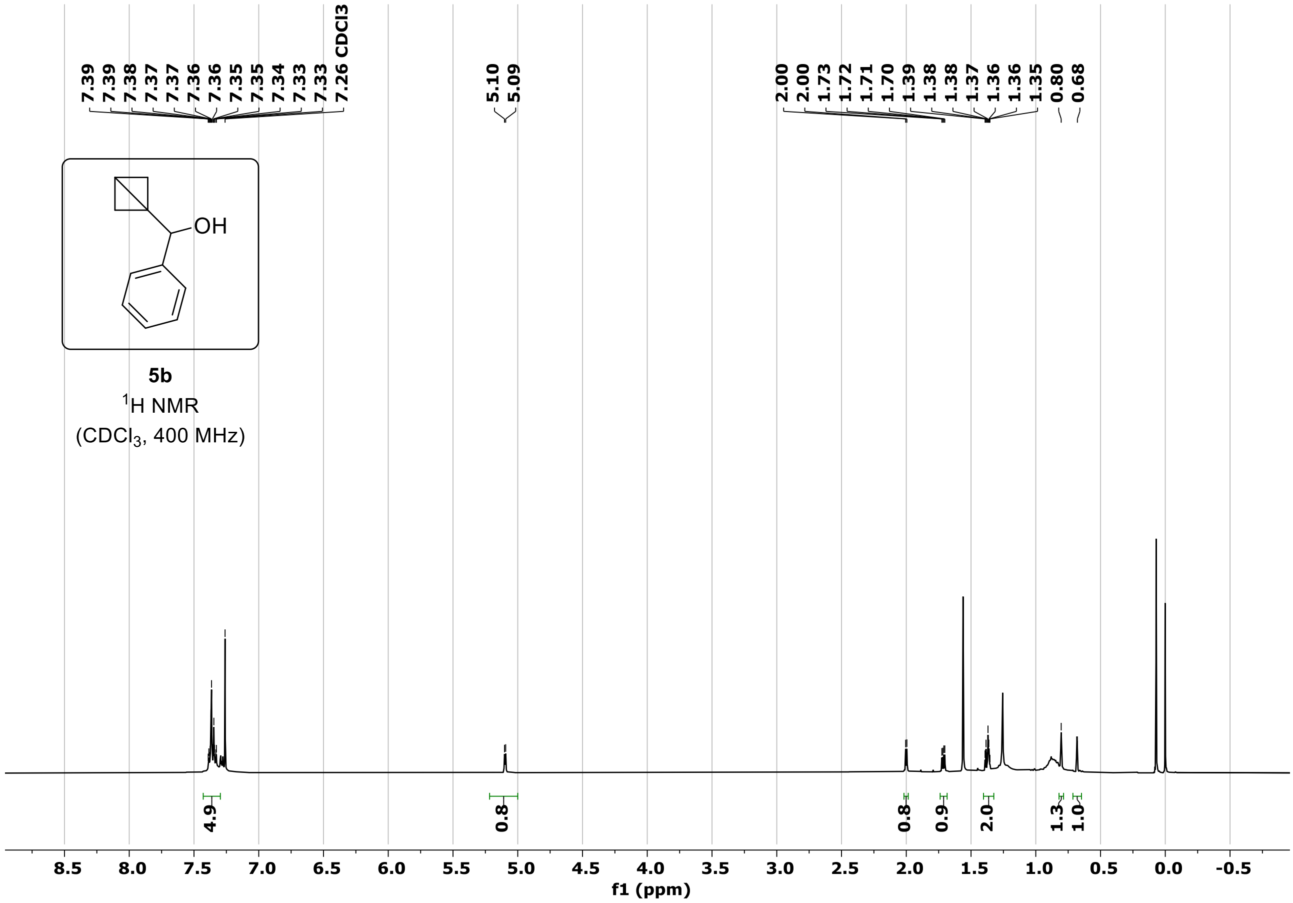
7.39
7.39
7.38
7.37
7.37
7.36
7.36
7.35
7.35
7.34
7.33
7.33
7.26 CDCl3
5.10
5.09
2.00
2.00
1.73
1.72
1.71
1.70
1.39
1.38
1.38
1.37
1.36
1.36
1.35
0.80
0.68
OH
5b
^{1}H NMR
(CDCl$_3$, 400 MHz)
4.9
0.8
0.8
0.9
2.0
1.3
1.0
f1 (ppm)

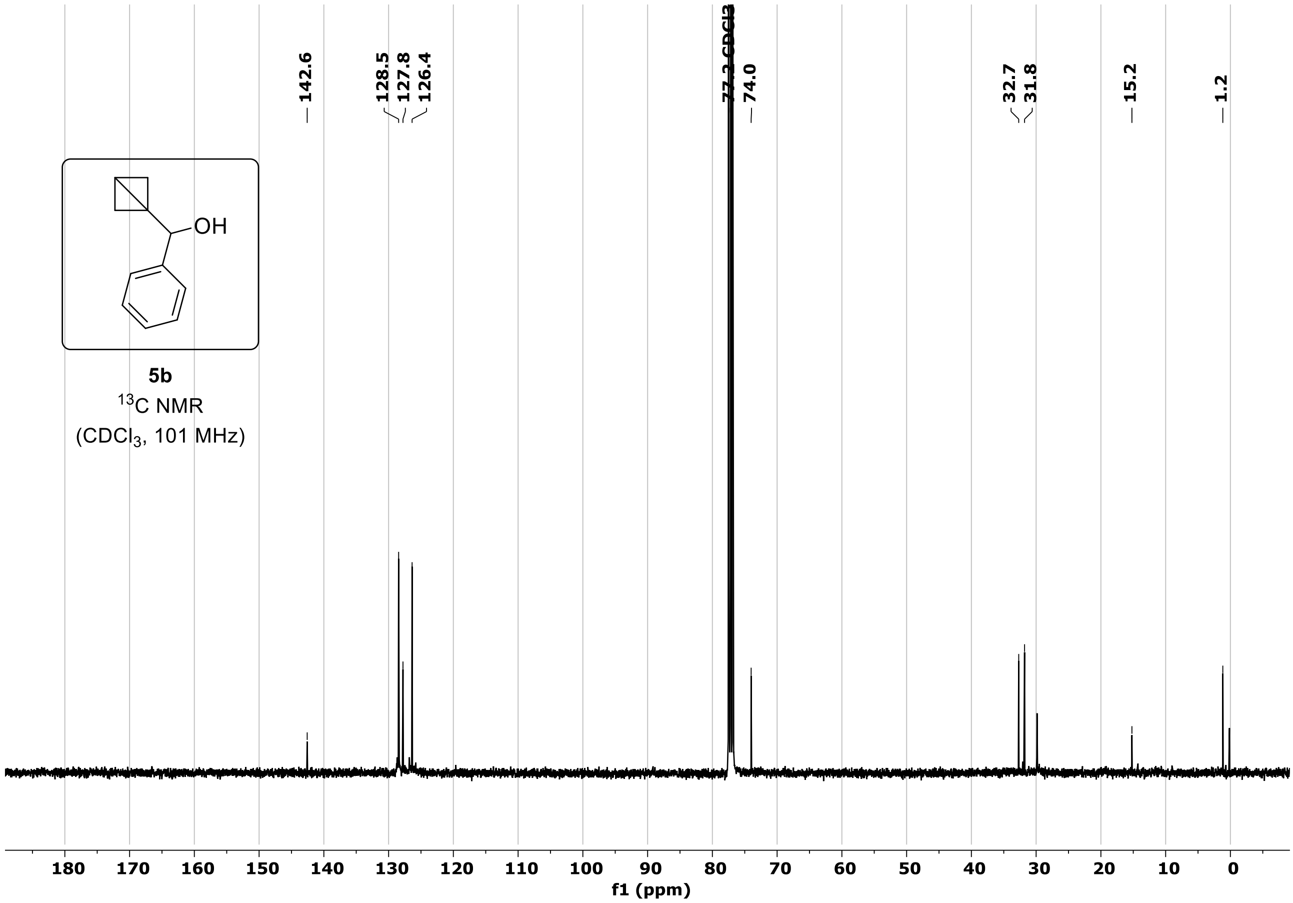
142.6
128.5
127.8
126.4
77.2 CDCl3
74.0
32.7
31.8
15.2
1.2
OH
5b
^{13}C NMR
(CDCl$_3$, 101 MHz)
f1 (ppm)

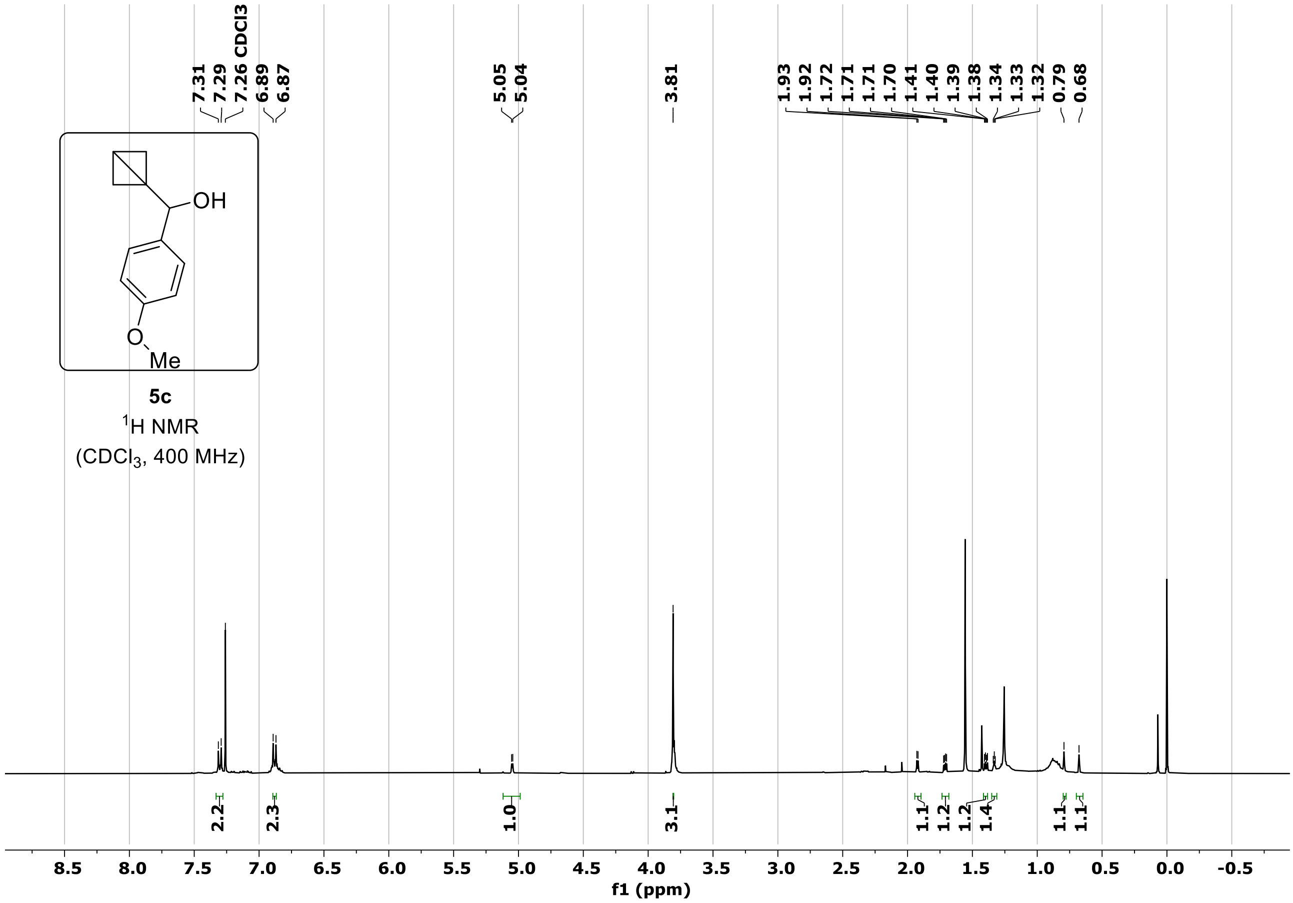
7.31
7.29
7.26 CDCl3
6.89
6.87
5.05
5.04
3.81
1.93
1.92
1.72
1.71
1.71
1.70
1.41
1.40
1.39
1.38
1.34
1.33
1.32
0.79
0.68
OH
O
Me
5c
¹H NMR
(CDCl3, 400 MHz)
2.2
2.3
1.0
3.1
1.1
1.2
1.2
1.4
1.1
1.1
8.5 8.0 7.5 7.0 6.5 6.0 5.5 5.0 4.5 4.0 3.5 3.0 2.5 2.0 1.5 1.0 0.5 0.0 -0.5
f1 (ppm)

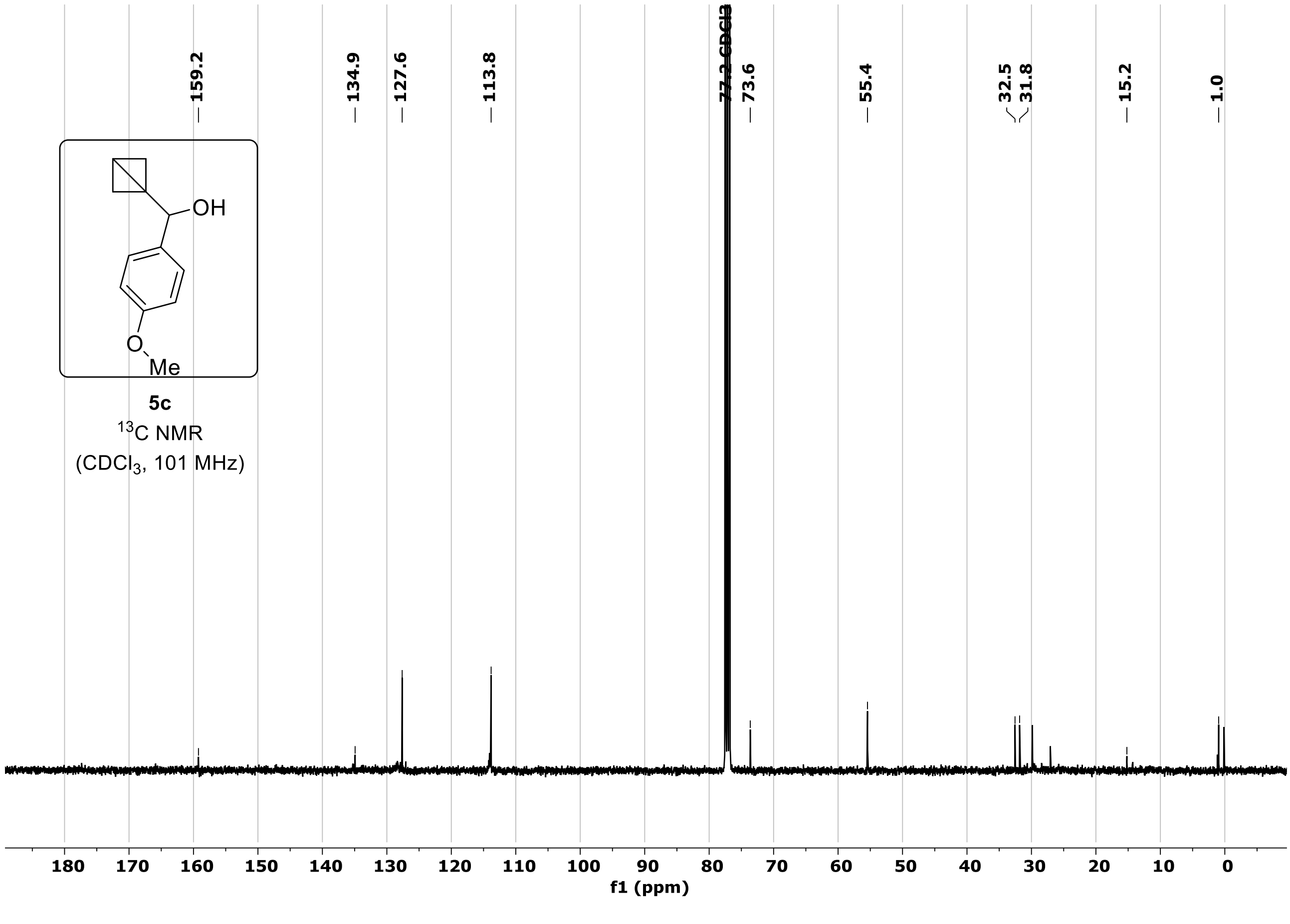
159.2
134.9
127.6
113.8
77.2 CDCl3
73.6
55.4
32.5
31.8
15.2
1.0
OH
O
Me
5c
¹³C NMR
(CDCl3, 101 MHz)
180 170 160 150 140 130 120 110 100 90 80 70 60 50 40 30 20 10 0
f1 (ppm)

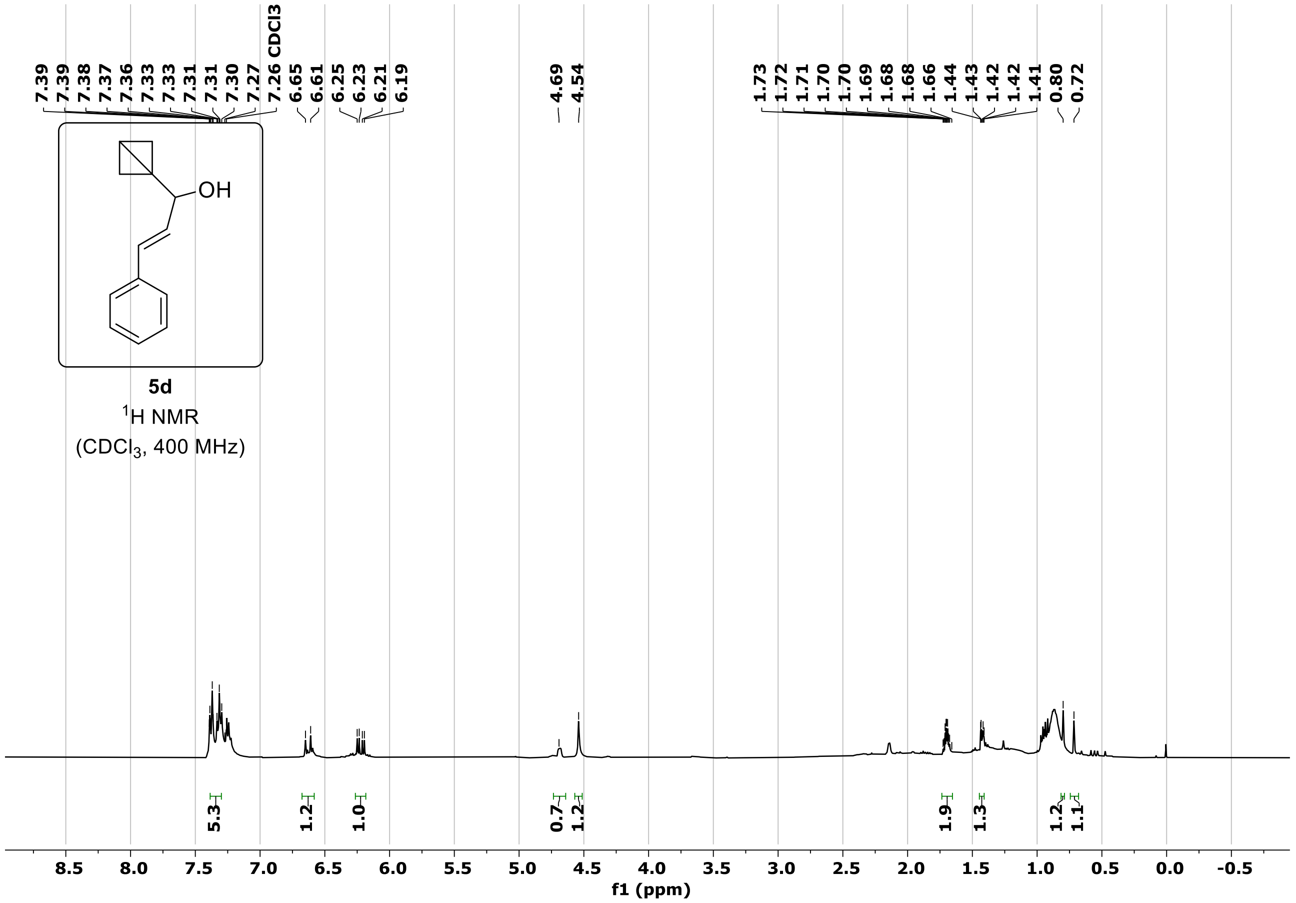
7.39 7.39 7.38 7.37 7.36 7.33 7.33 7.31 7.31 7.30 7.27 7.26 CDCl3 6.65 6.61 6.25 6.23 6.21 6.19
4.69 4.54
1.73 1.72 1.71 1.70 1.70 1.69 1.68 1.68 1.66 1.44 1.43 1.42 1.42 1.41 0.80 0.72
OH
5d
1H NMR
(CDCl3, 400 MHz)
5.3 1.2 1.0 0.7 1.2 1.9 1.3 1.2 1.1
8.5 8.0 7.5 7.0 6.5 6.0 5.5 5.0 4.5 4.0 3.5 3.0 2.5 2.0 1.5 1.0 0.5 0.0 -0.5
f1 (ppm)

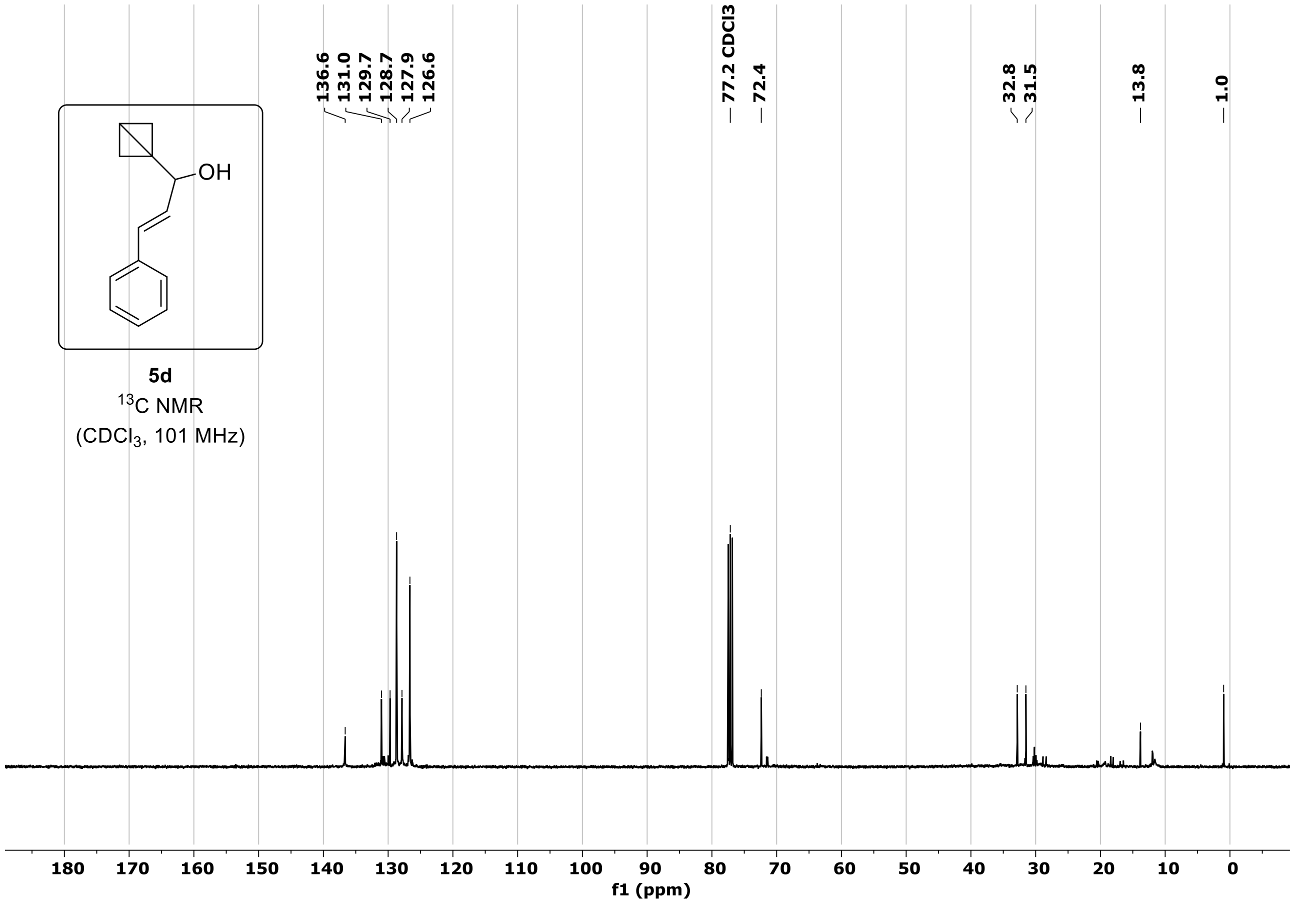
136.6 131.0 129.7 128.7 127.9 126.6
77.2 CDCl3 72.4
32.8 31.5
13.8
1.0
OH
5d
13C NMR
(CDCl3, 101 MHz)
180 170 160 150 140 130 120 110 100 90 80 70 60 50 40 30 20 10 0
f1 (ppm)

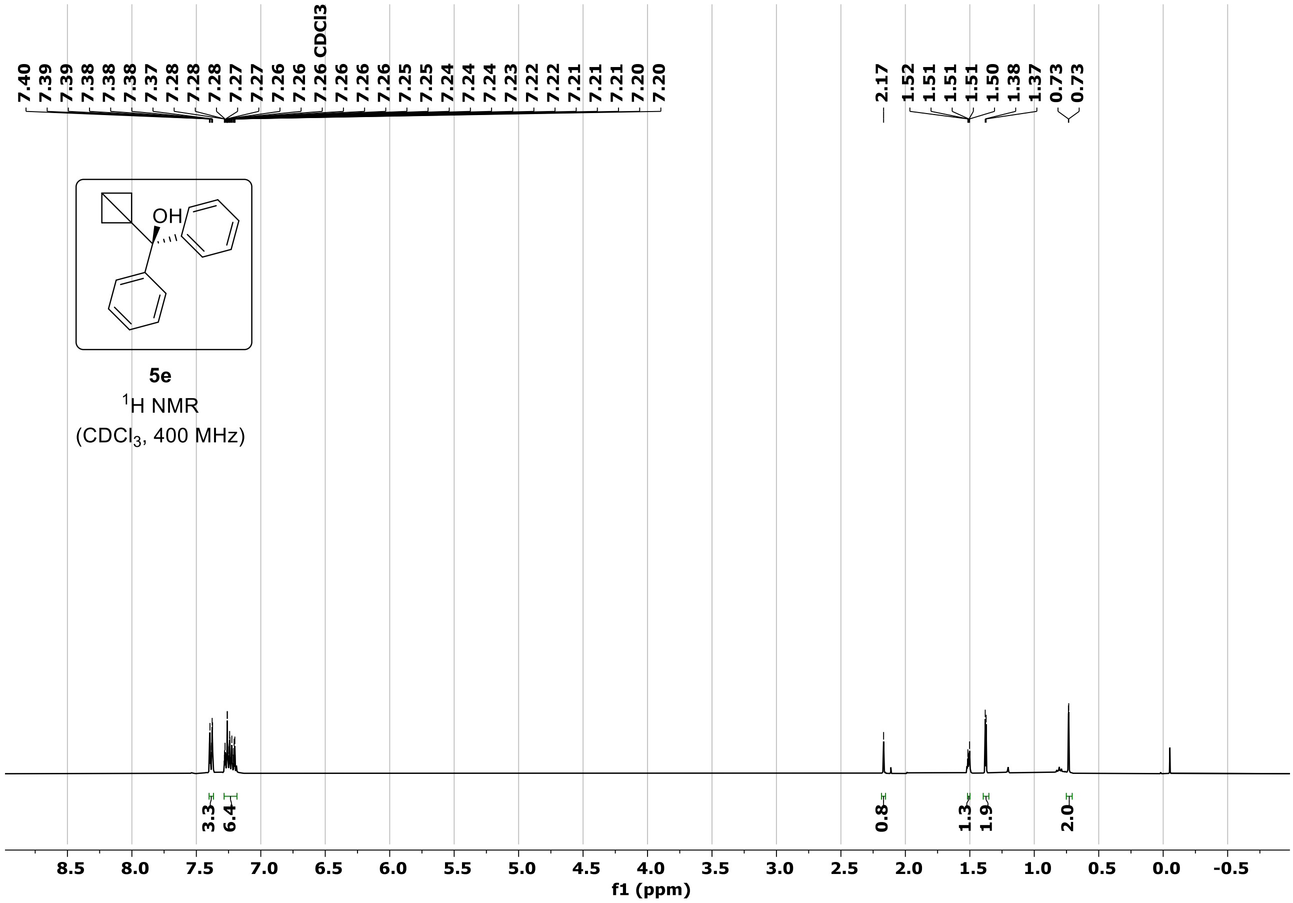
7.40 7.39 7.39 7.38 7.38 7.38 7.37 7.28 7.28 7.28 7.27 7.27 7.26 7.26 CDCl3 7.26 7.26 7.26 7.25 7.25 7.24 7.24 7.24 7.23 7.22 7.22 7.21 7.21 7.21 7.20 7.20
2.17 1.52 1.51 1.51 1.51 1.50 1.38 1.37 0.73 0.73
OH
5e
^{1}H NMR
(CDCl$_3$, 400 MHz)
3.3 6.4 0.8 1.3 1.9 2.0
8.5 8.0 7.5 7.0 6.5 6.0 5.5 5.0 4.5 4.0 3.5 3.0 2.5 2.0 1.5 1.0 0.5 0.0 -0.5
f1 (ppm)

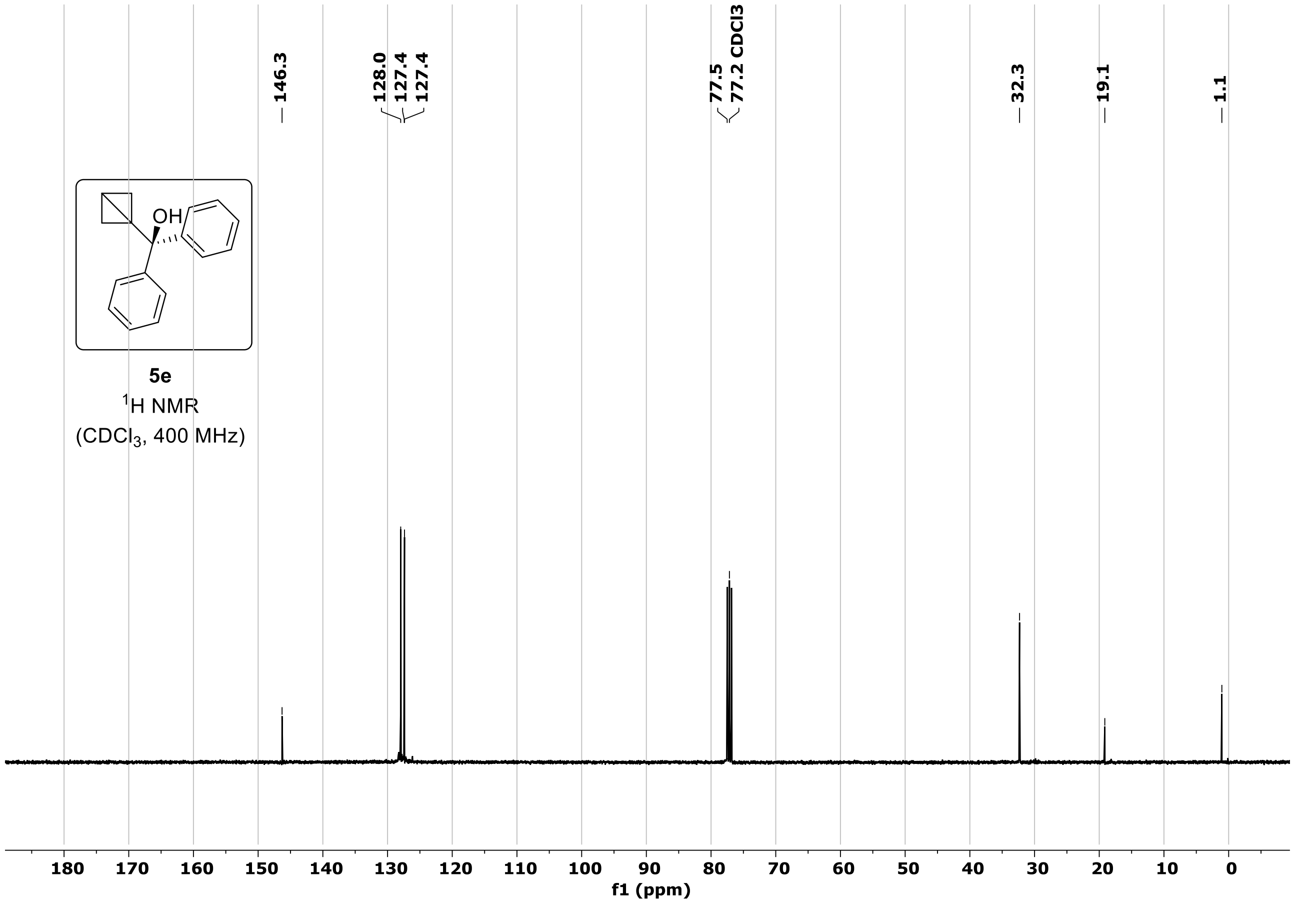
146.3
128.0 127.4 127.4
77.5 77.2 CDCl3
32.3
19.1
1.1
OH
5e
^{1}H NMR
(CDCl$_3$, 400 MHz)
180 170 160 150 140 130 120 110 100 90 80 70 60 50 40 30 20 10 0
f1 (ppm)

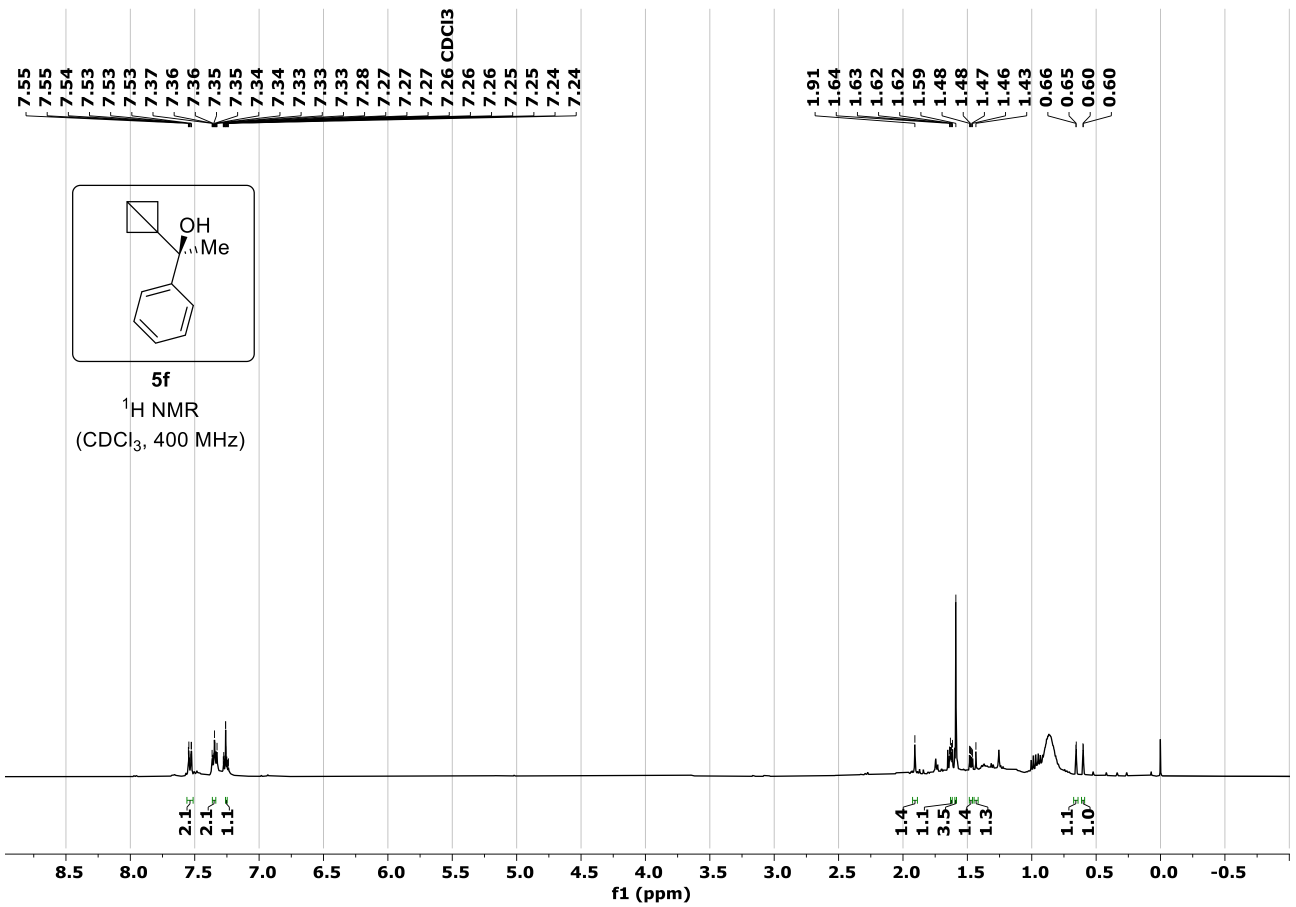
7.55 7.55 7.54 7.53 7.53 7.53 7.37 7.36 7.36 7.35 7.35 7.34 7.34 7.33 7.33 7.33 7.28 7.27 7.27 7.27 7.26 CDCl3 7.26 7.26 7.25 7.25 7.24 7.24
1.91 1.64 1.63 1.62 1.62 1.59 1.48 1.48 1.47 1.46 1.43 0.66 0.65 0.60 0.60
OH
Me
5f
¹H NMR
(CDCl₃, 400 MHz)
2.1 2.1 1.1 1.4 1.1 3.5 1.4 1.3 1.1 1.0
f1 (ppm)

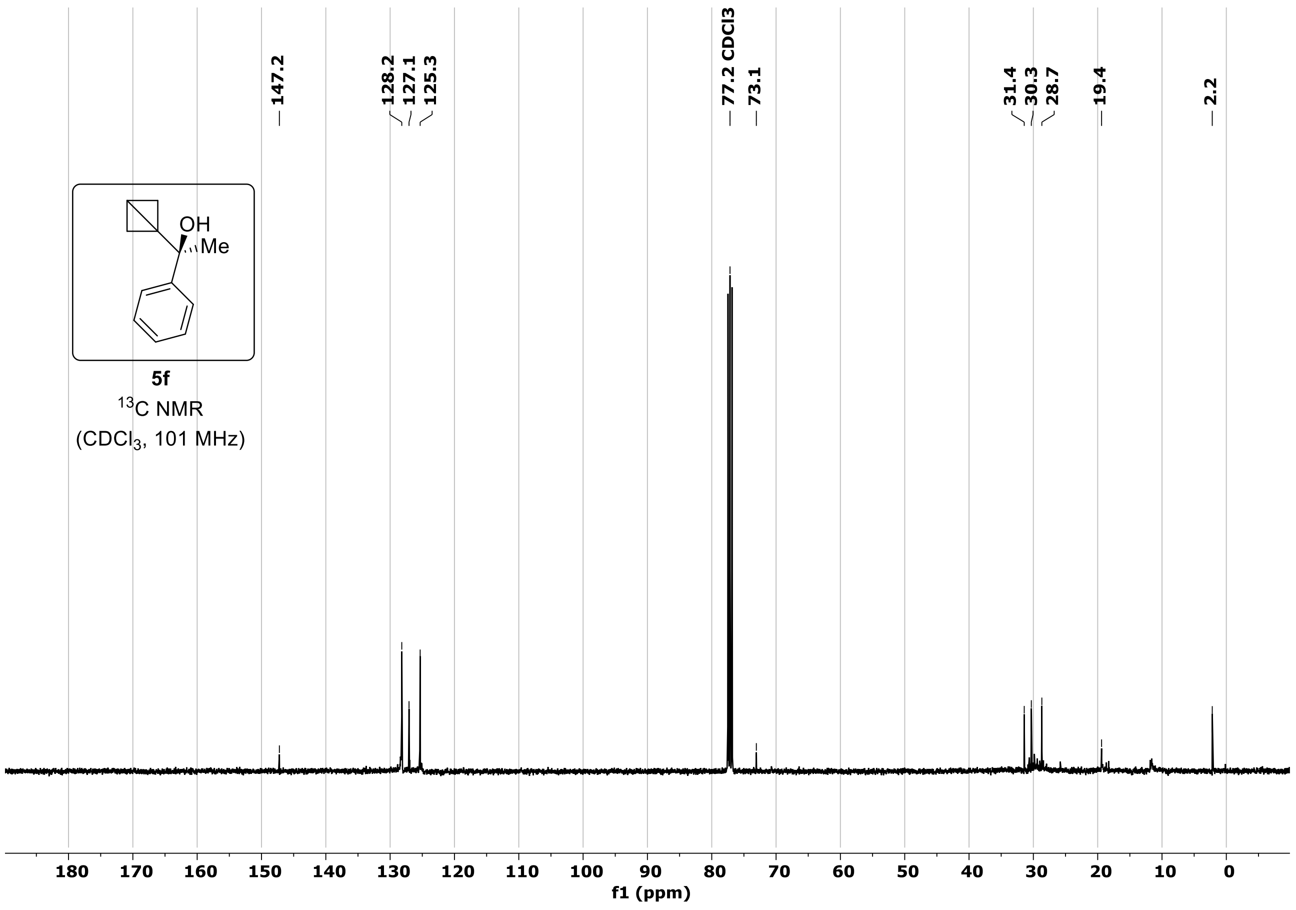
147.2
128.2 127.1 125.3
77.2 CDCl3
73.1
31.4 30.3 28.7
19.4
2.2
OH
Me
5f
¹³C NMR
(CDCl₃, 101 MHz)
f1 (ppm)

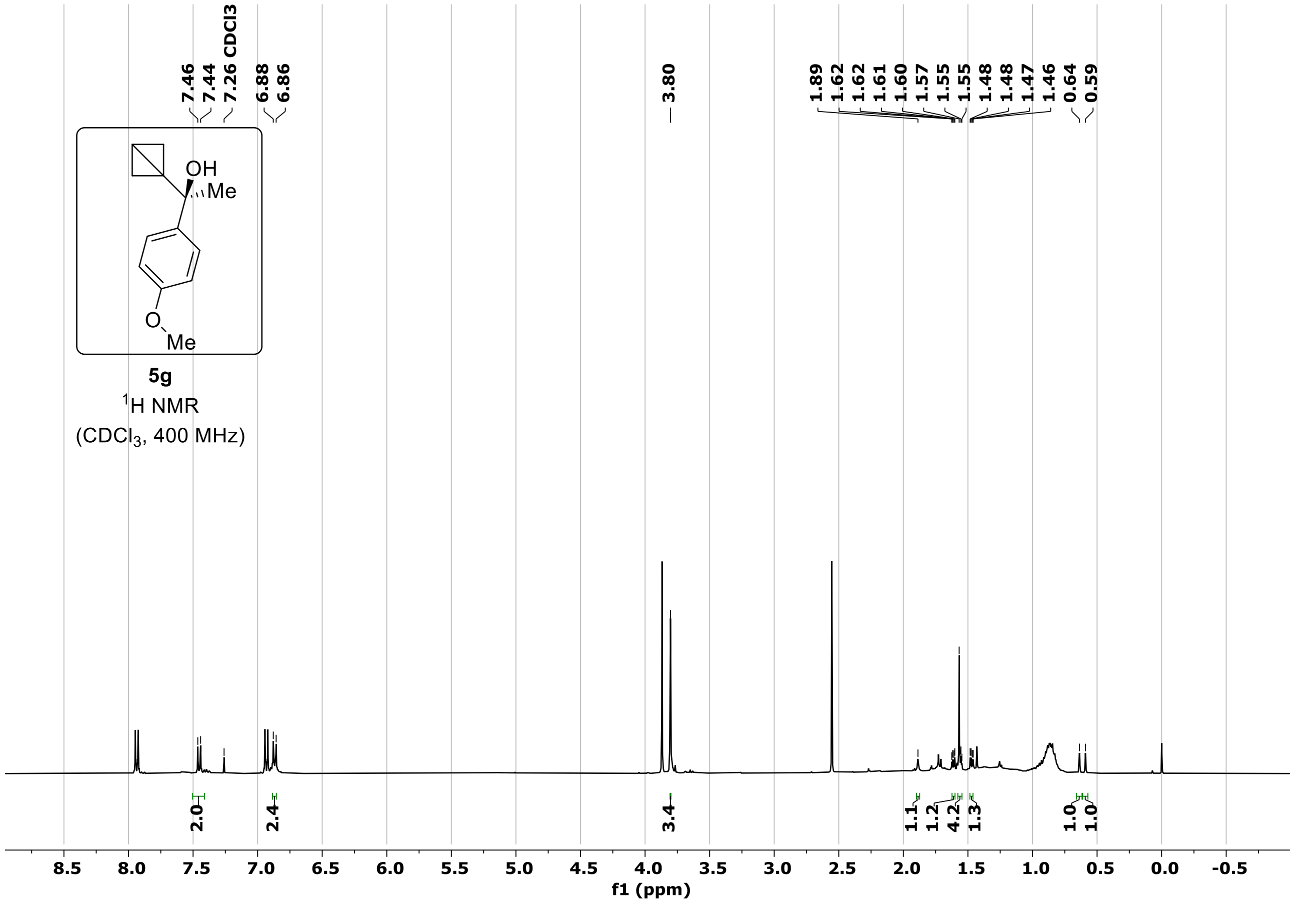

5g
^{1}H NMR
($CDCl_3$, 400 MHz)

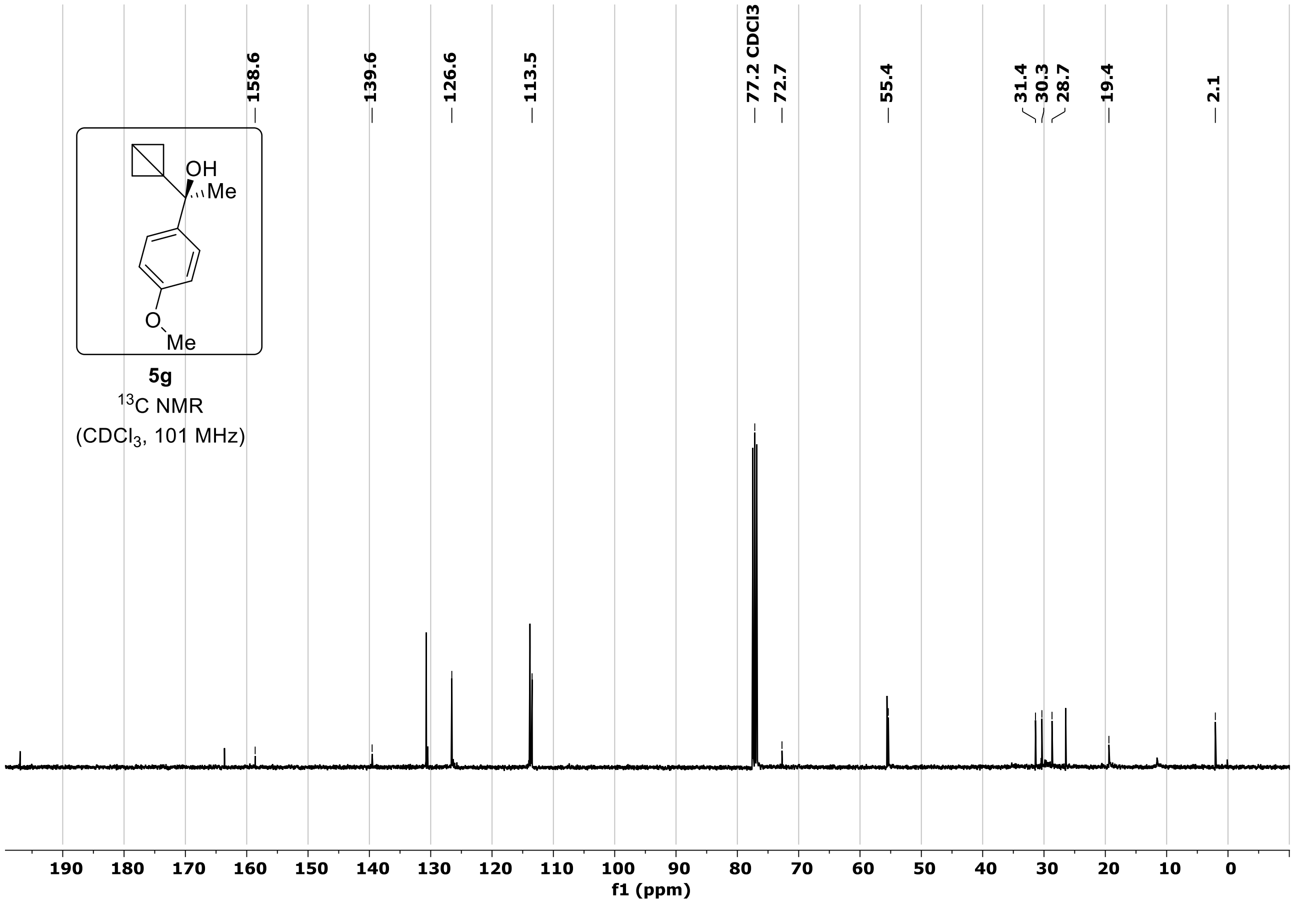

5g
^{13}C NMR
($CDCl_3$, 101 MHz)

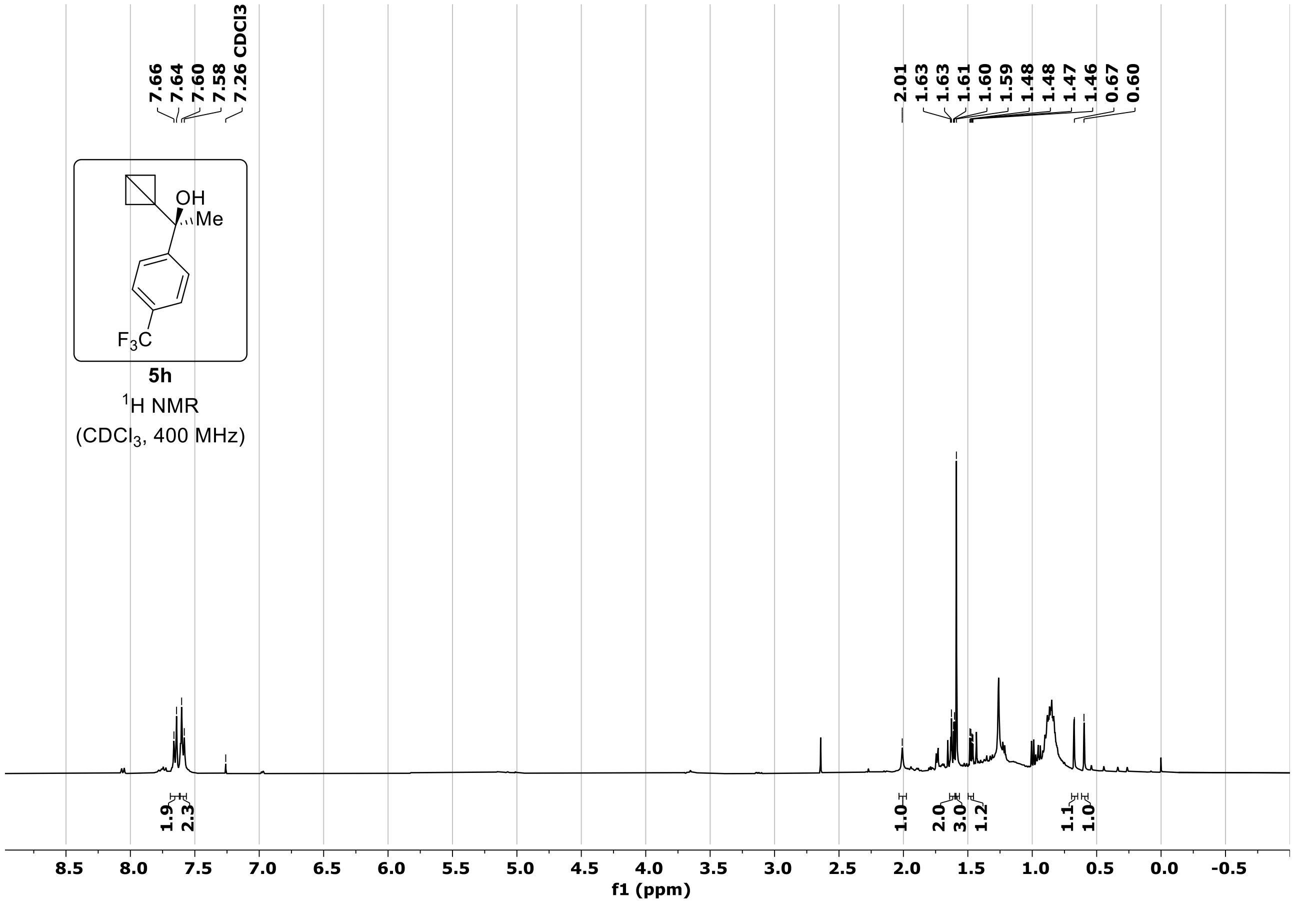
7.66
7.64
7.60
7.58
7.26 CDCl3
2.01
1.63
1.63
1.61
1.60
1.59
1.48
1.48
1.47
1.46
0.67
0.60
OH
Me
F3C
5h
1H NMR
(CDCl3, 400 MHz)
1.9
2.3
1.0
2.0
3.0
1.2
1.1
1.0
8.5 8.0 7.5 7.0 6.5 6.0 5.5 5.0 4.5 4.0 3.5 3.0 2.5 2.0 1.5 1.0 0.5 0.0 -0.5
f1 (ppm)

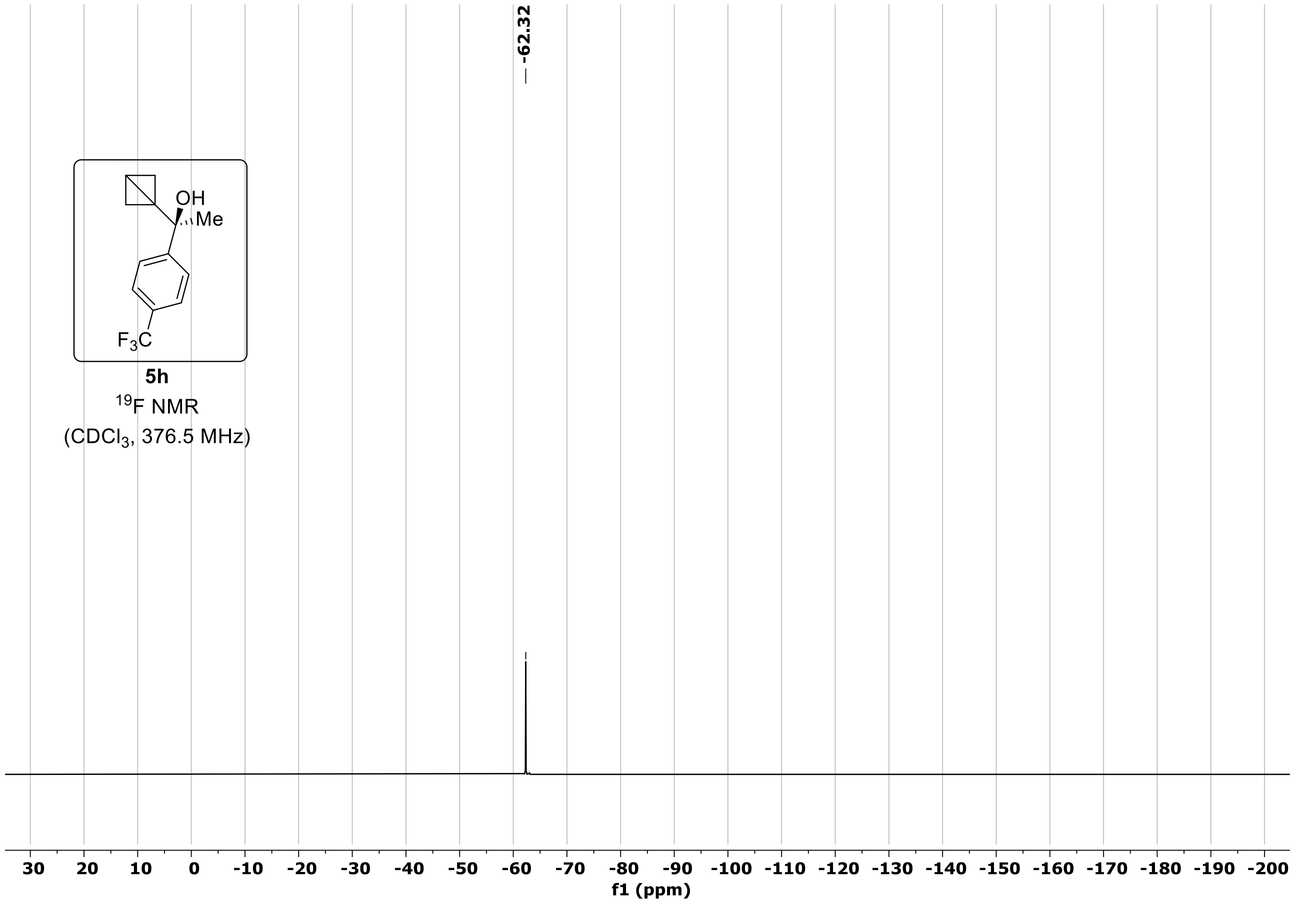
-62.32
OH
Me
F3C
5h
19F NMR
(CDCl3, 376.5 MHz)
30 20 10 0 -10 -20 -30 -40 -50 -60 -70 -80 -90 -100 -110 -120 -130 -140 -150 -160 -170 -180 -190 -200
f1 (ppm)

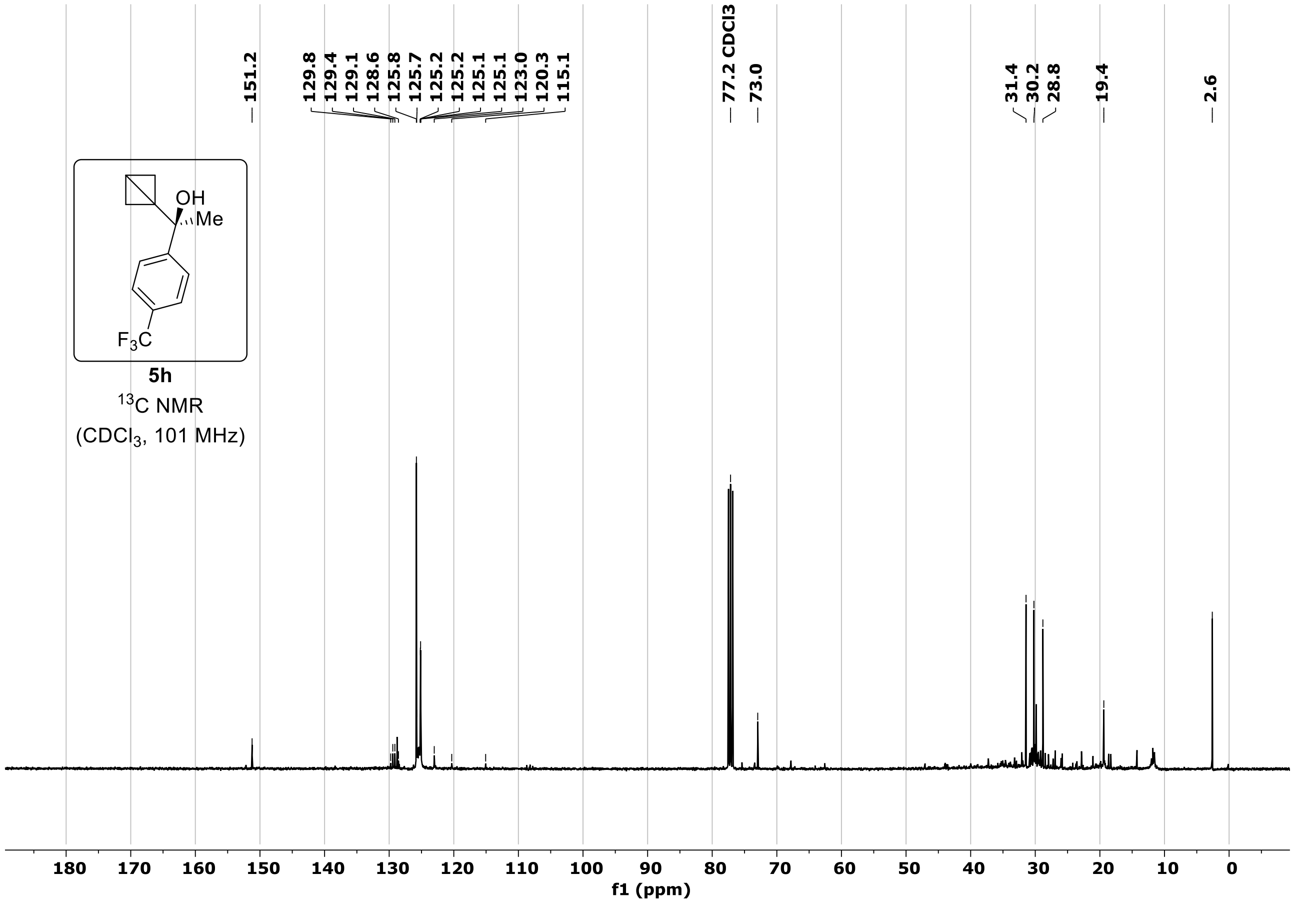
151.2
129.8
129.4
129.1
128.6
125.8
125.7
125.2
125.2
125.1
125.1
123.0
120.3
115.1
77.2 CDCl3
73.0
31.4
30.2
28.8
19.4
2.6
OH
Me
F3C
5h
13C NMR
(CDCl3, 101 MHz)
f1 (ppm)

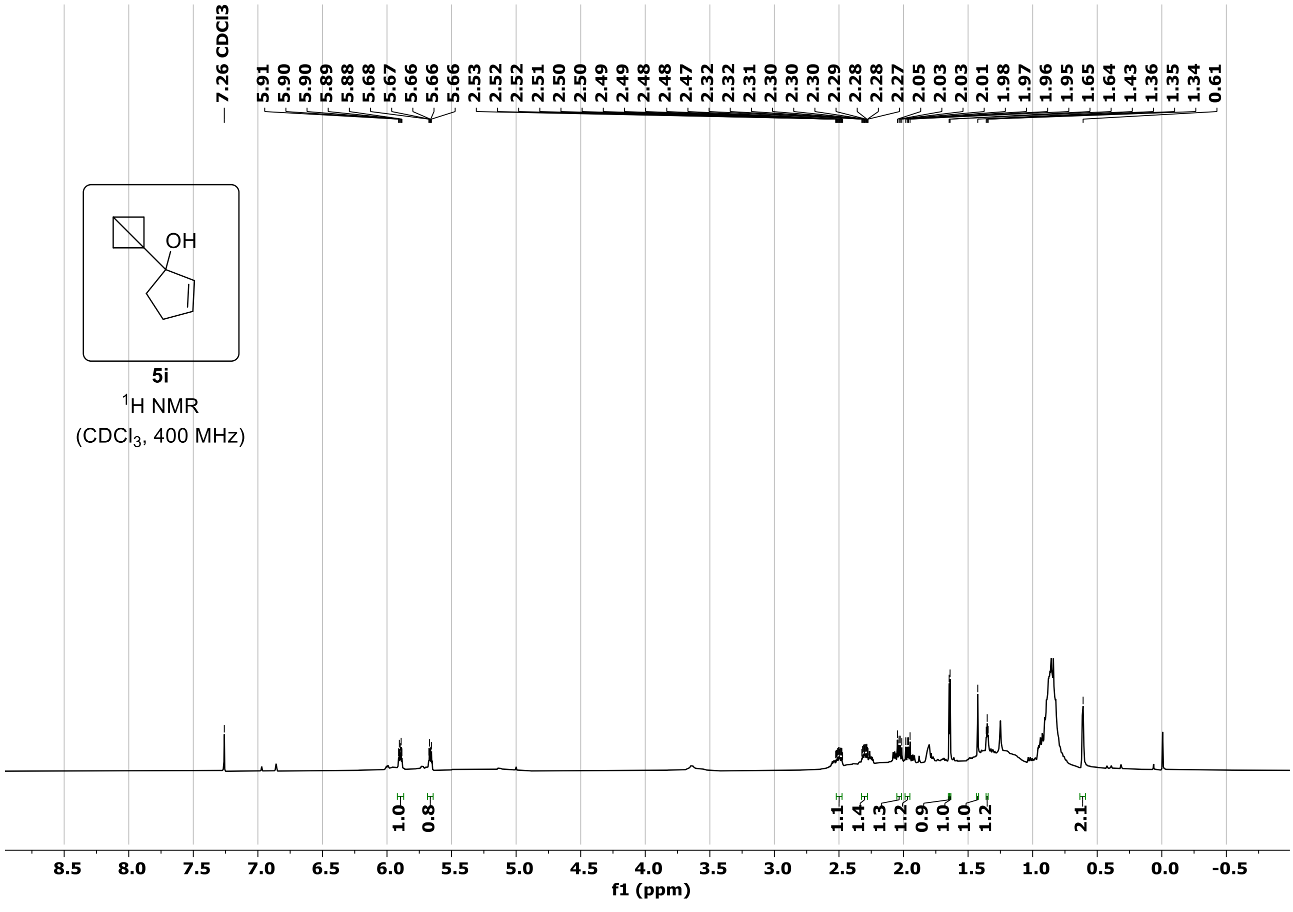
7.26 CDCl3
5.91
5.90
5.90
5.89
5.88
5.68
5.67
5.66
5.66
5.66
2.53
2.52
2.52
2.51
2.50
2.49
2.49
2.48
2.48
2.47
2.32
2.32
2.31
2.30
2.30
2.30
2.29
2.28
2.28
2.27
2.05
2.03
2.03
2.01
1.98
1.97
1.96
1.95
1.65
1.64
1.43
1.36
1.35
1.34
0.61
OH
5i
1H NMR
(CDCl3, 400 MHz)
1.0
0.8
1.1
1.4
1.3
1.2
0.9
1.0
1.0
1.2
2.1
f1 (ppm)

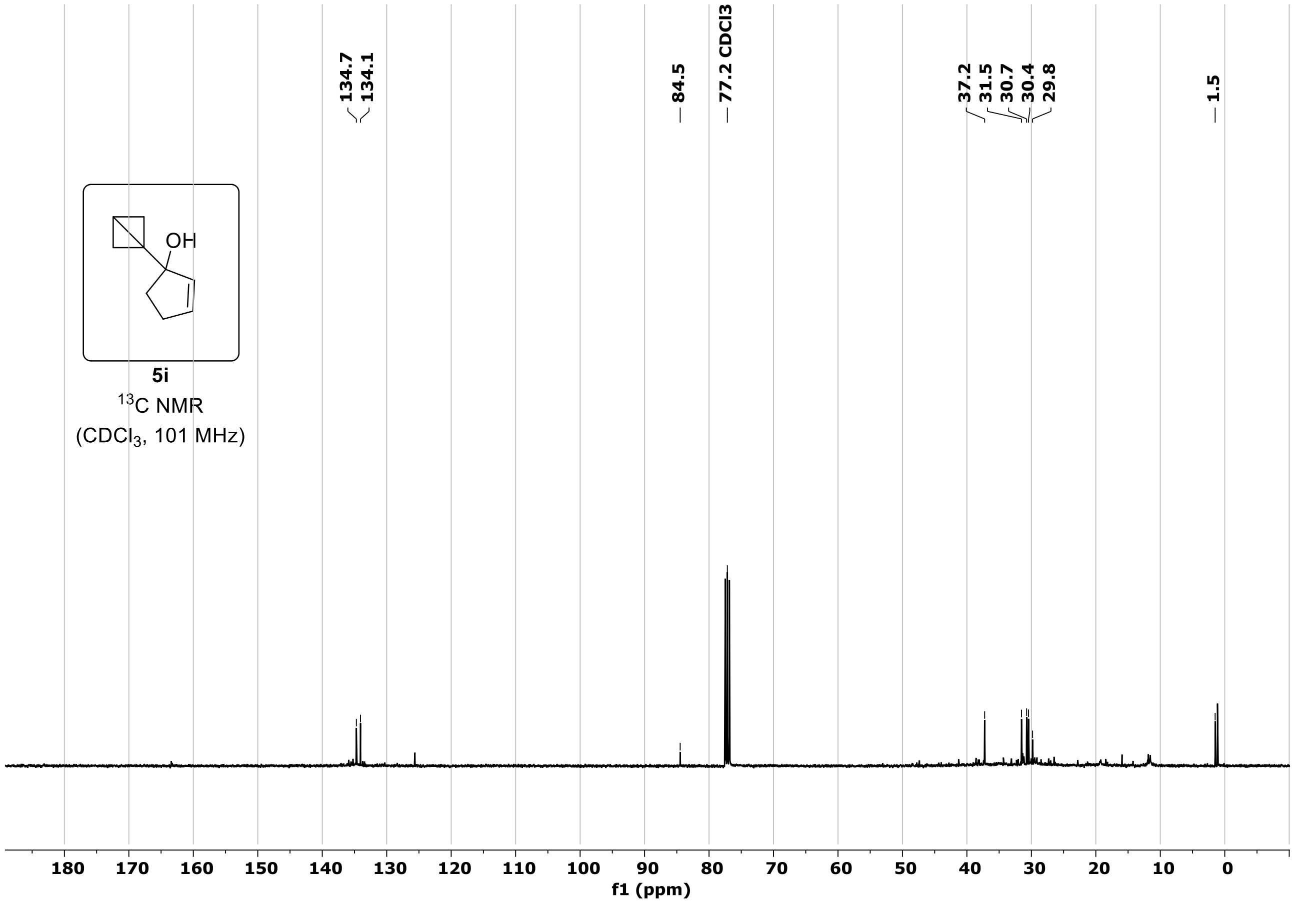
134.7
134.1
84.5
77.2 CDCl3
37.2
31.5
30.7
30.4
29.8
1.5
OH
5i
13C NMR
(CDCl3, 101 MHz)
180 170 160 150 140 130 120 110 100 90 80 70 60 50 40 30 20 10 0
f1 (ppm)

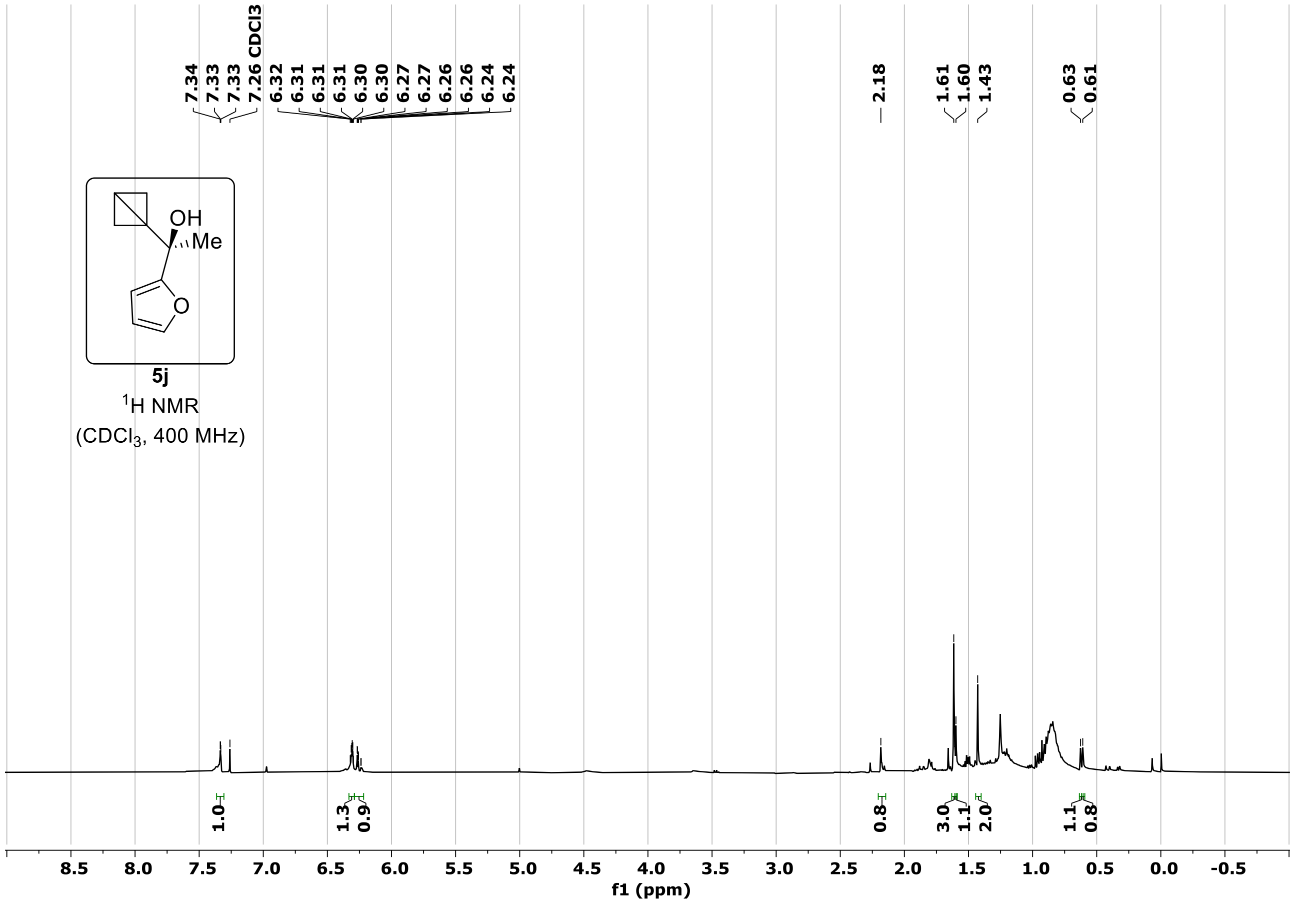
7.34
7.33
7.33
7.26 CDCl3
6.32
6.31
6.31
6.30
6.30
6.27
6.26
6.26
6.24
6.24
2.18
1.61
1.60
1.43
0.63
0.61
OH
Me
O
5j
1H NMR
(CDCl3, 400 MHz)
1.0
1.3
0.9
0.8
3.0
1.1
2.0
1.1
0.8
8.5 8.0 7.5 7.0 6.5 6.0 5.5 5.0 4.5 4.0 3.5 3.0 2.5 2.0 1.5 1.0 0.5 0.0 -0.5
f1 (ppm)

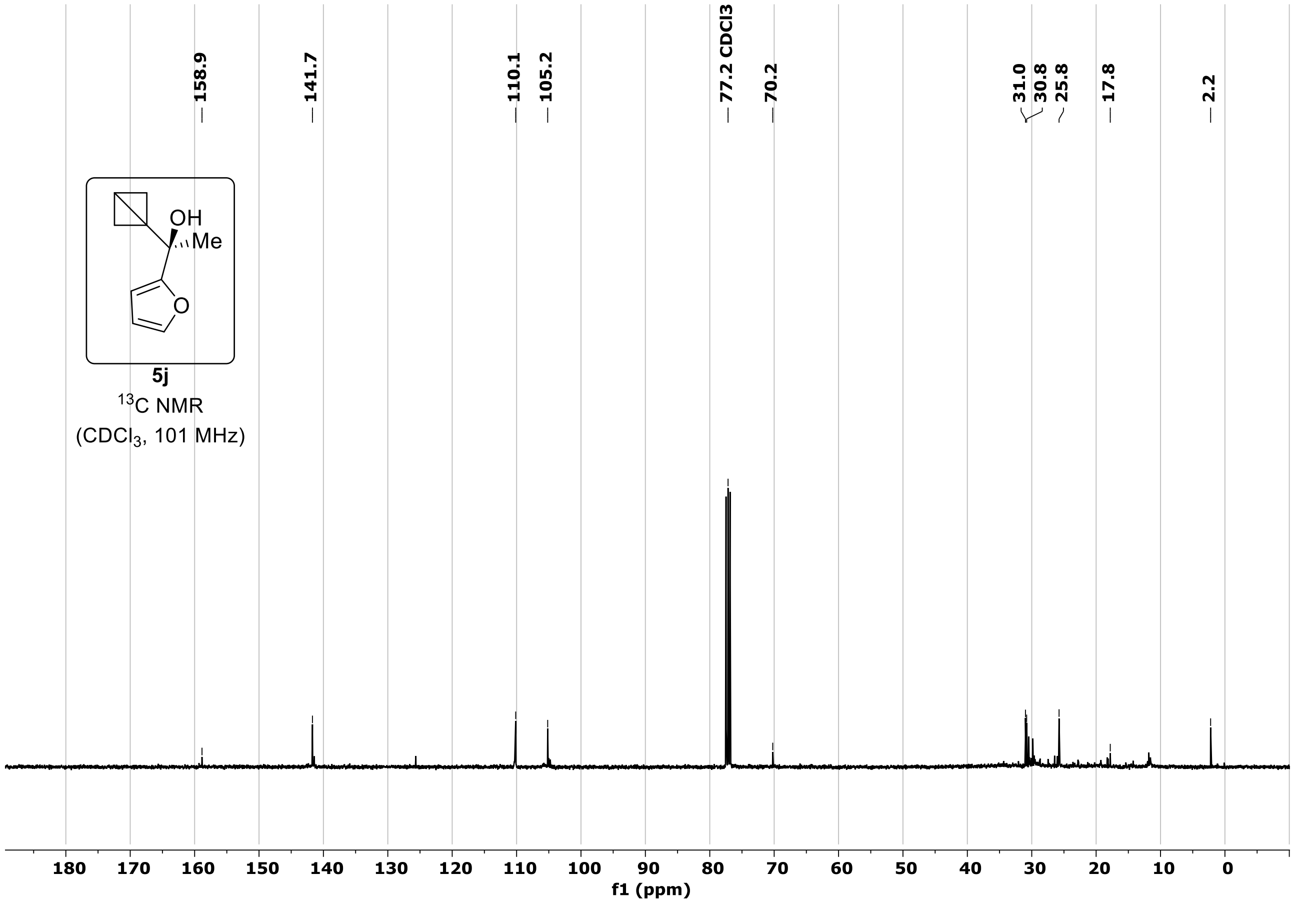
158.9
141.7
110.1
105.2
77.2 CDCl3
70.2
31.0
30.8
25.8
17.8
2.2
OH
Me
O
5j
13C NMR
(CDCl3, 101 MHz)
180 170 160 150 140 130 120 110 100 90 80 70 60 50 40 30 20 10 0
f1 (ppm)

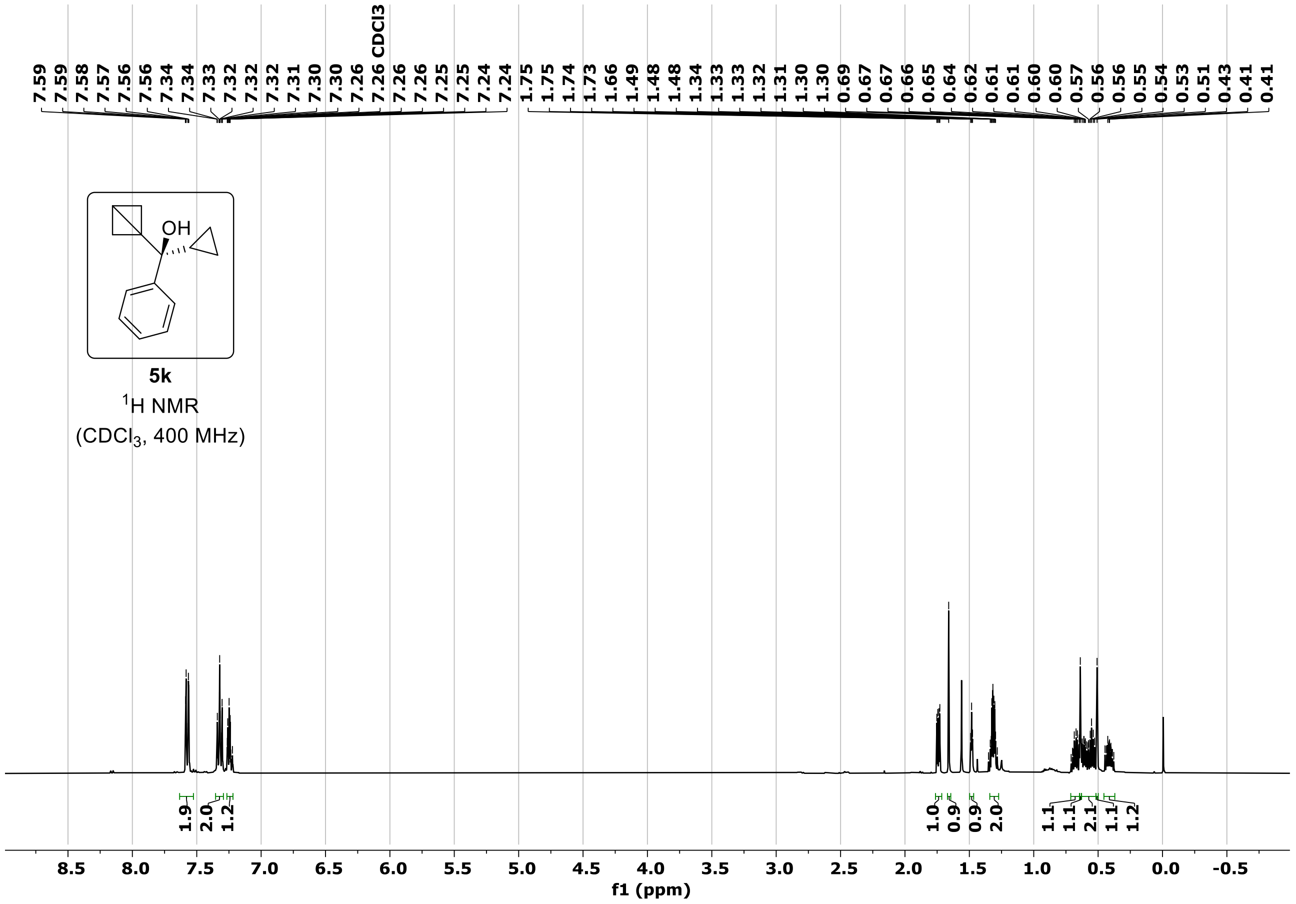
7.59 7.59 7.58 7.57 7.56 7.34 7.34 7.33 7.32 7.32 7.32 7.31 7.30 7.30 7.26 7.26 CDCl3 7.26 7.26 7.25 7.25 7.24 7.24
1.75 1.75 1.74 1.73 1.66 1.49 1.48 1.48 1.34 1.33 1.33 1.32 1.31 1.30 1.30 0.69 0.67 0.66 0.65 0.64 0.62 0.61 0.61 0.60 0.60 0.57 0.56 0.56 0.55 0.54 0.53 0.51 0.43 0.41 0.41
OH
5k
1H NMR
(CDCl3, 400 MHz)
1.9 2.0 1.2
1.0 0.9 0.9 2.0
1.1 1.1 2.1 1.1 1.2
8.5 8.0 7.5 7.0 6.5 6.0 5.5 5.0 4.5 4.0 3.5 3.0 2.5 2.0 1.5 1.0 0.5 0.0 -0.5
f1 (ppm)

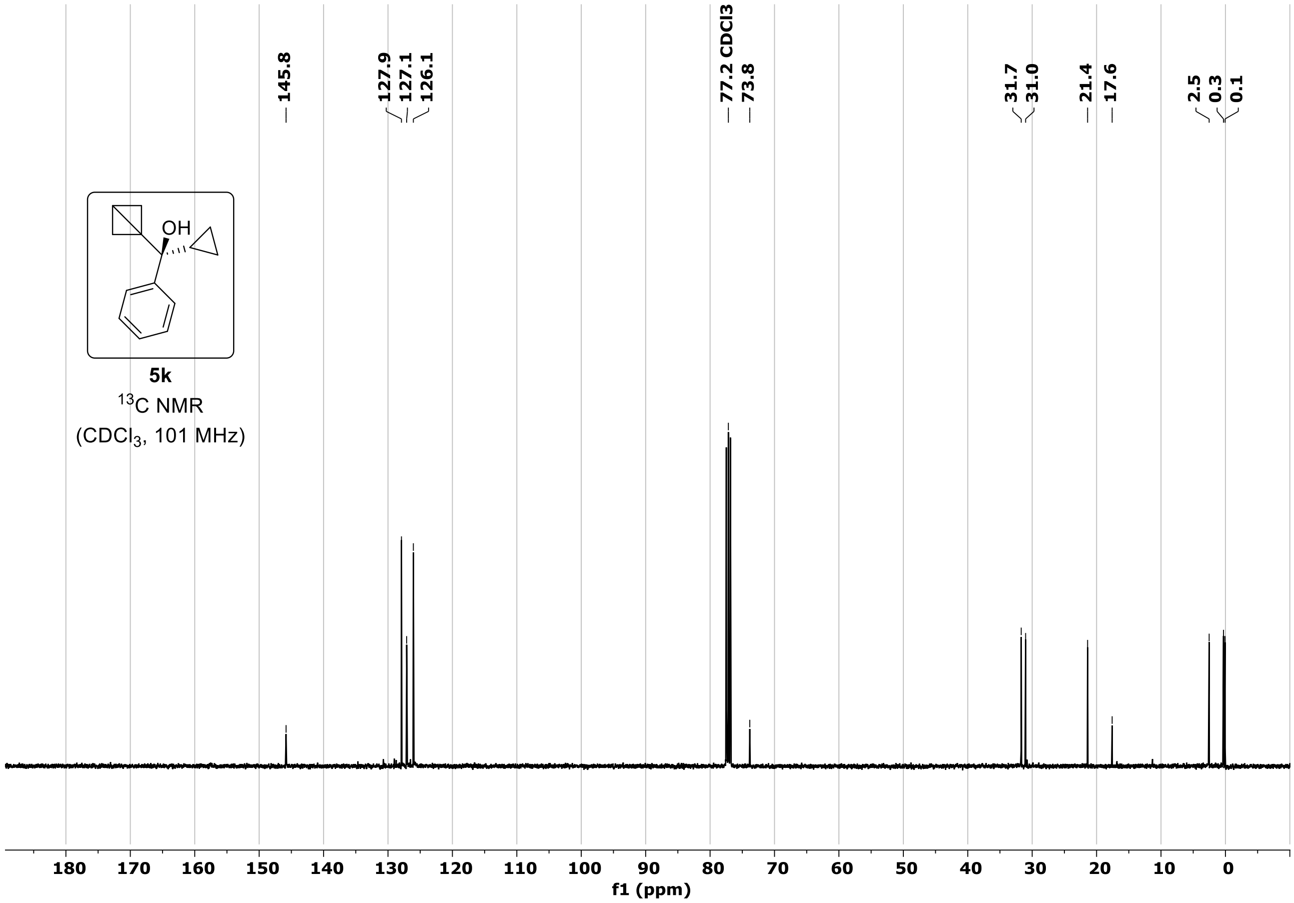
145.8
127.9
127.1
126.1
77.2 CDCl3
73.8
31.7
31.0
21.4
17.6
2.5
0.3
0.1
OH
5k
13C NMR
(CDCl3, 101 MHz)
180 170 160 150 140 130 120 110 100 90 80 70 60 50 40 30 20 10 0
f1 (ppm)

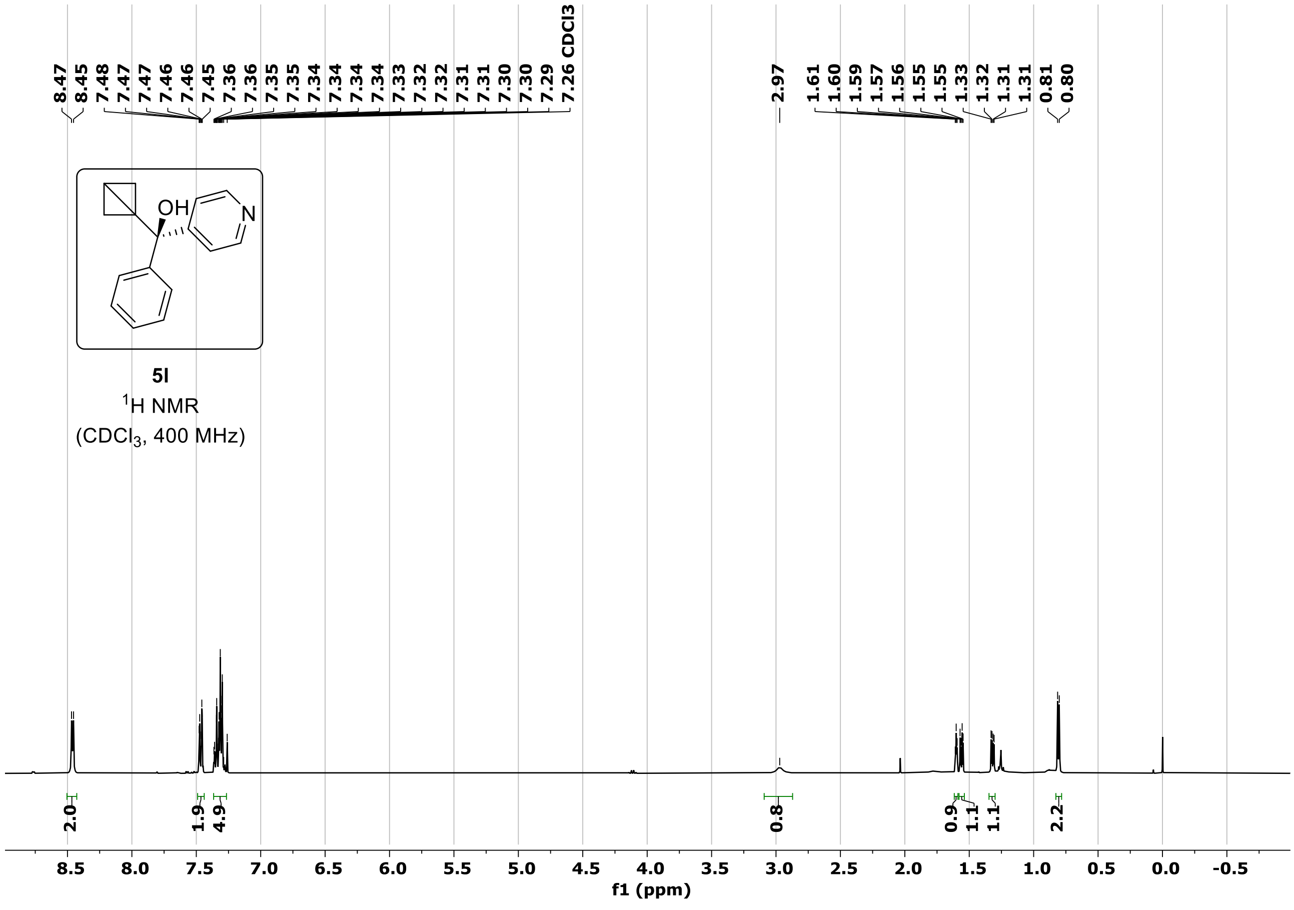
8.47
8.45
7.48
7.47
7.46
7.46
7.45
7.36
7.36
7.35
7.35
7.34
7.34
7.34
7.34
7.33
7.32
7.31
7.31
7.30
7.29
7.26 CDCl3
2.97
1.61
1.60
1.59
1.57
1.56
1.55
1.55
1.33
1.32
1.31
1.31
0.81
0.80
OH
N
5l
1H NMR
(CDCl3, 400 MHz)
2.0
1.9
4.9
0.8
0.9
1.1
1.1
2.2
8.5 8.0 7.5 7.0 6.5 6.0 5.5 5.0 4.5 4.0 3.5 3.0 2.5 2.0 1.5 1.0 0.5 0.0 -0.5
f1 (ppm)

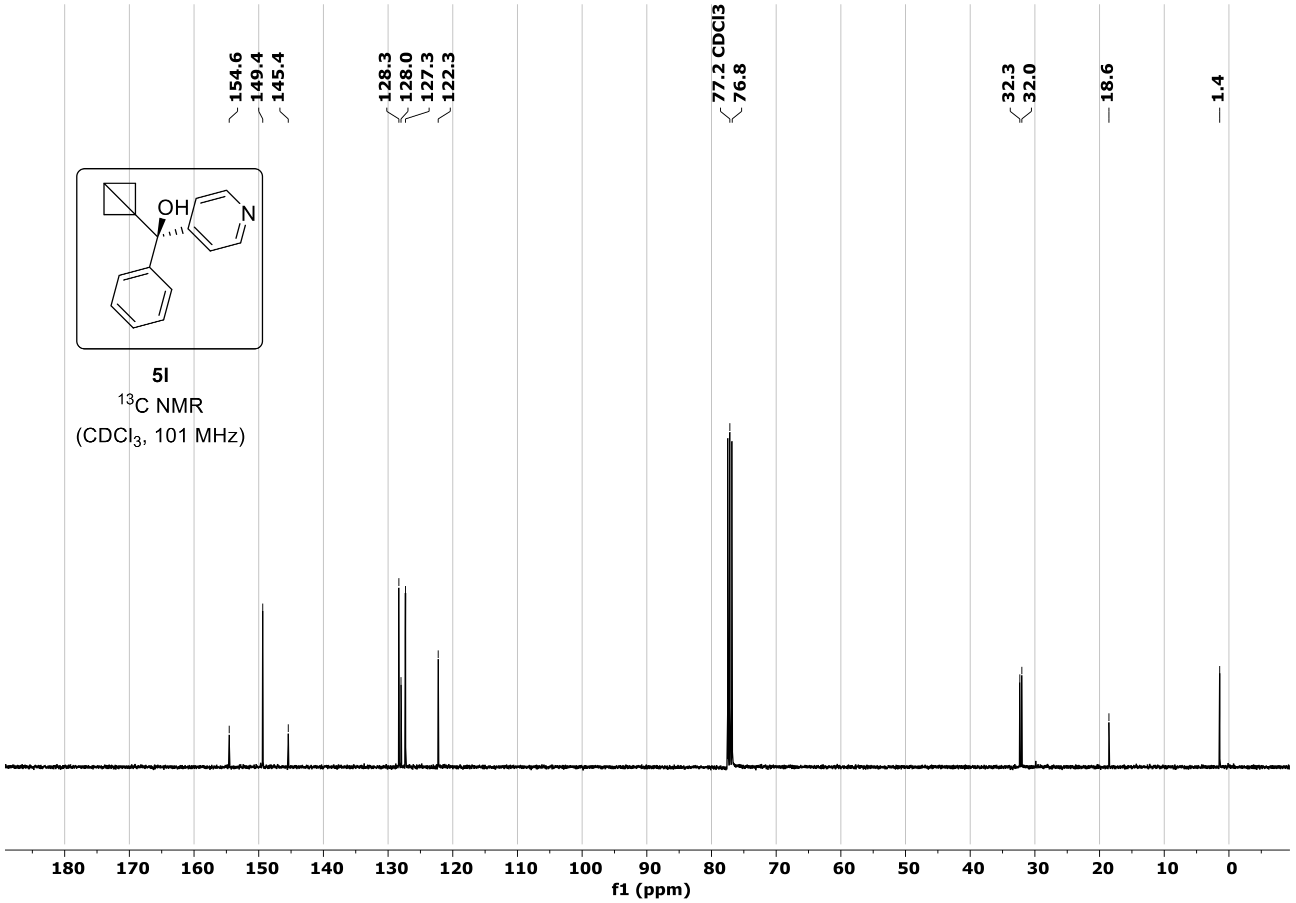
154.6
149.4
145.4
128.3
128.0
127.3
122.3
77.2 CDCl3
76.8
32.3
32.0
18.6
1.4
OH
N
5l
13C NMR
(CDCl3, 101 MHz)
180 170 160 150 140 130 120 110 100 90 80 70 60 50 40 30 20 10 0
f1 (ppm)

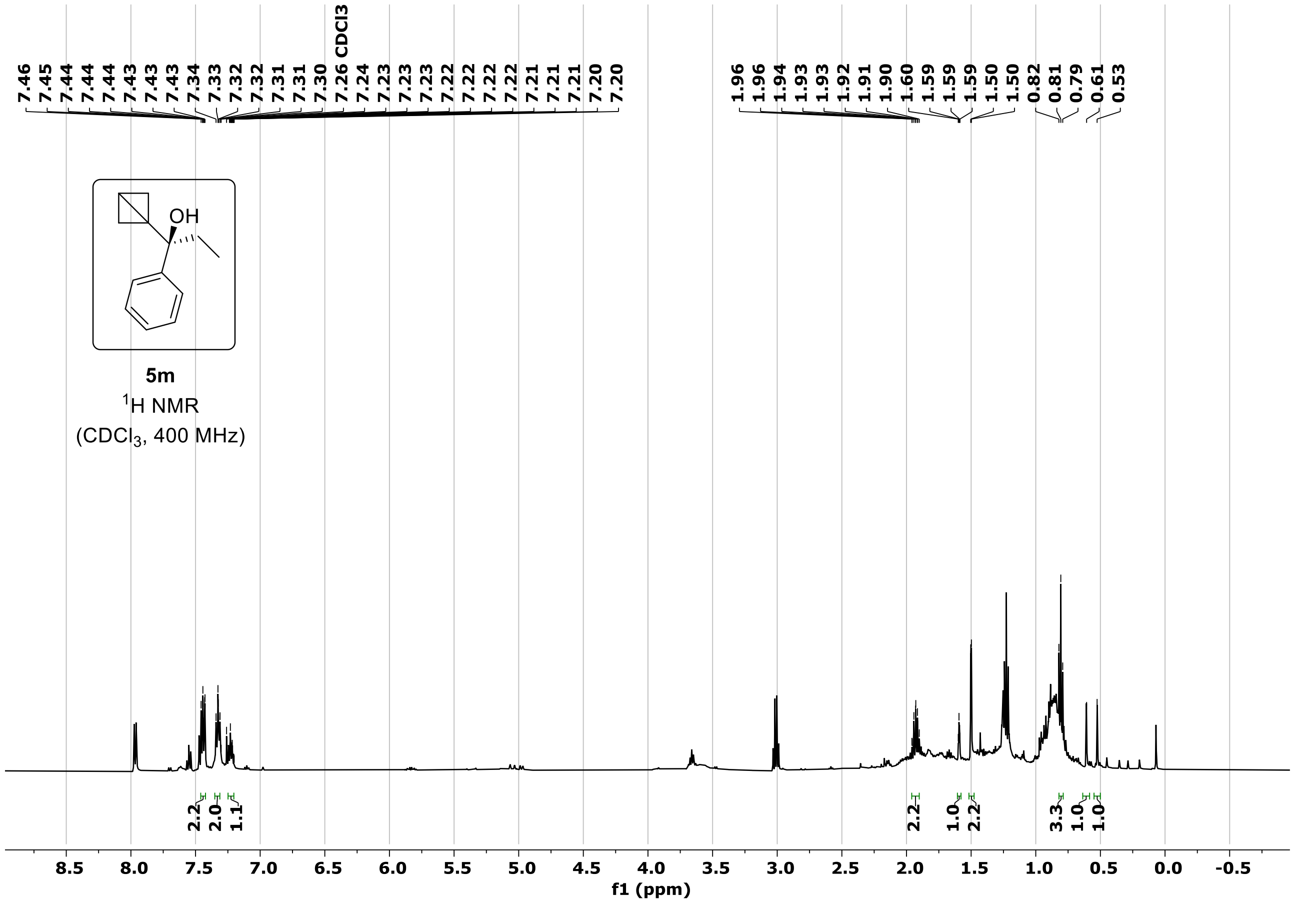
7.46
7.45
7.44
7.44
7.44
7.43
7.43
7.43
7.34
7.33
7.32
7.32
7.31
7.31
7.30
7.26 CDCl3
7.24
7.23
7.23
7.23
7.22
7.22
7.22
7.22
7.21
7.21
7.21
7.20
7.20
1.96
1.96
1.94
1.93
1.93
1.92
1.91
1.90
1.60
1.59
1.59
1.59
1.50
1.50
0.82
0.81
0.79
0.61
0.53
OH
5m
1H NMR
(CDCl3, 400 MHz)
2.2
2.0
1.1
2.2
1.0
2.2
3.3
1.0
1.0
8.5 8.0 7.5 7.0 6.5 6.0 5.5 5.0 4.5 4.0 3.5 3.0 2.5 2.0 1.5 1.0 0.5 0.0 -0.5
f1 (ppm)

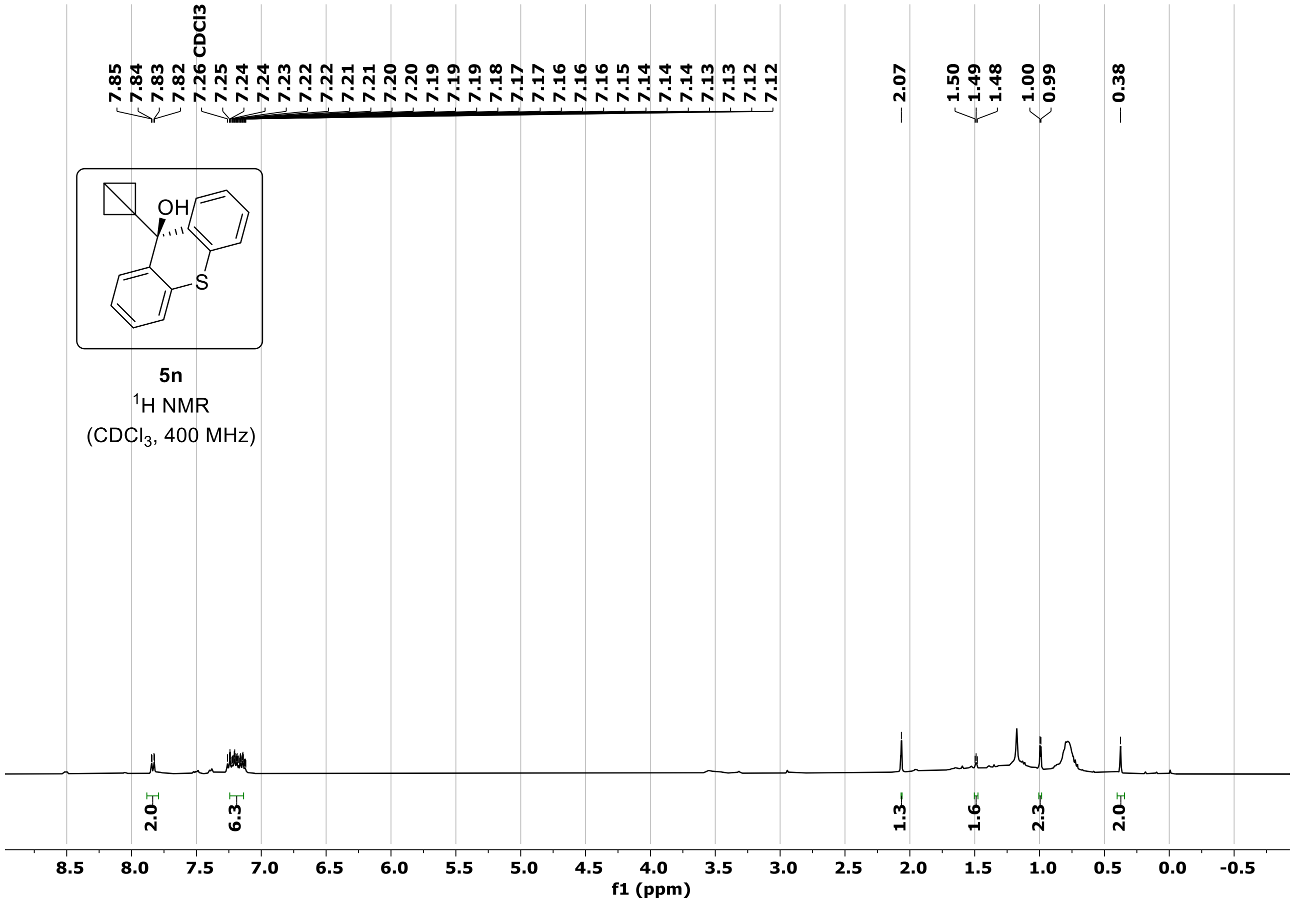
7.85
7.84
7.83
7.82
7.26 CDCl3
7.25
7.24
7.24
7.23
7.22
7.22
7.21
7.21
7.20
7.20
7.19
7.19
7.18
7.17
7.17
7.16
7.16
7.15
7.14
7.14
7.14
7.13
7.13
7.12
7.12
2.07
1.50
1.49
1.48
1.00
0.99
0.38
OH
S
5n
^{1}H NMR
($CDCl_3$, 400 MHz)
2.0
6.3
1.3
1.6
2.3
2.0
8.5 8.0 7.5 7.0 6.5 6.0 5.5 5.0 4.5 4.0 3.5 3.0 2.5 2.0 1.5 1.0 0.5 0.0 -0.5
f1 (ppm)

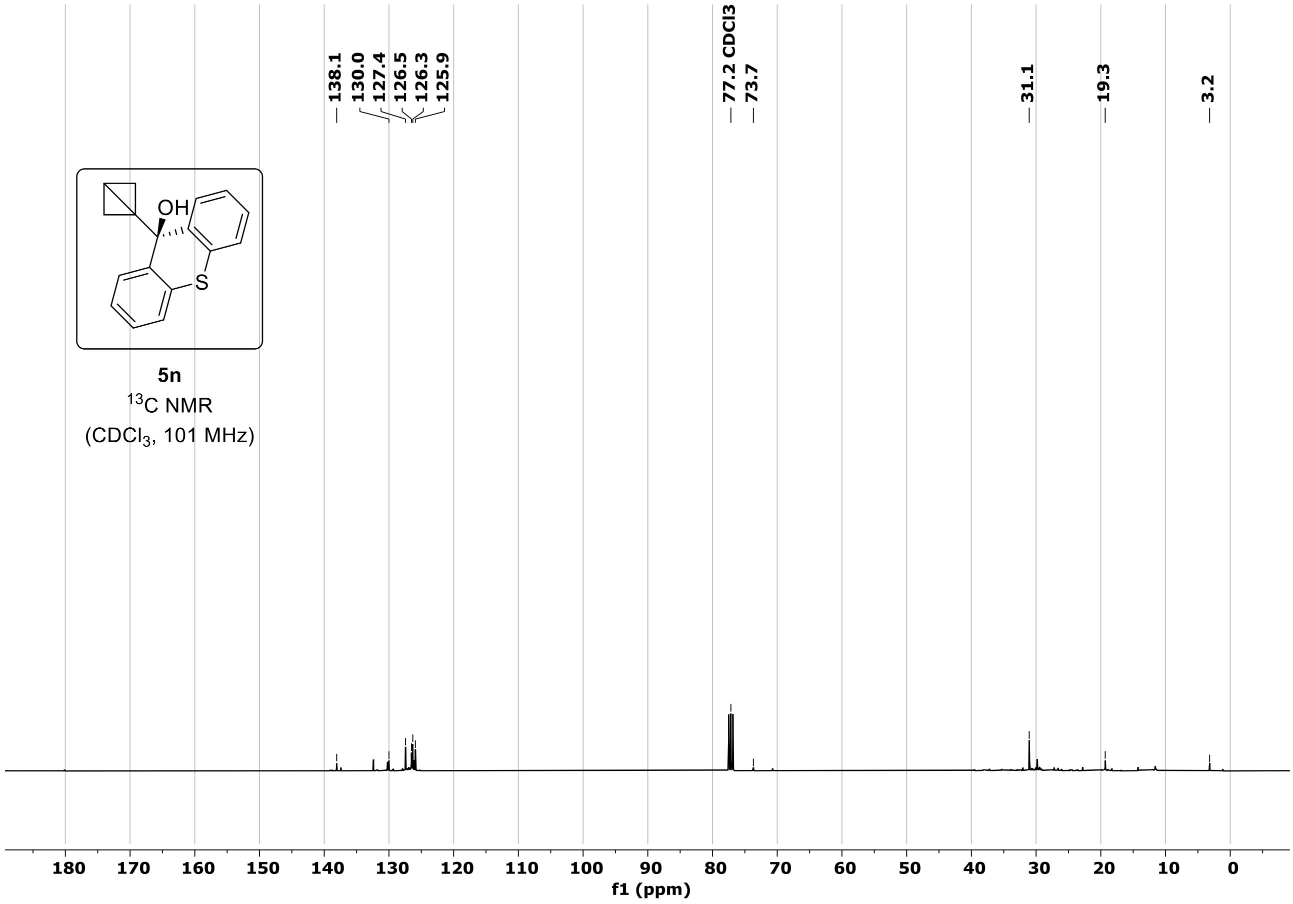
138.1
130.0
127.4
126.5
126.3
125.9
77.2 CDCl3
73.7
31.1
19.3
3.2
OH
S
5n
^{13}C NMR
($CDCl_3$, 101 MHz)
180 170 160 150 140 130 120 110 100 90 80 70 60 50 40 30 20 10 0
f1 (ppm)

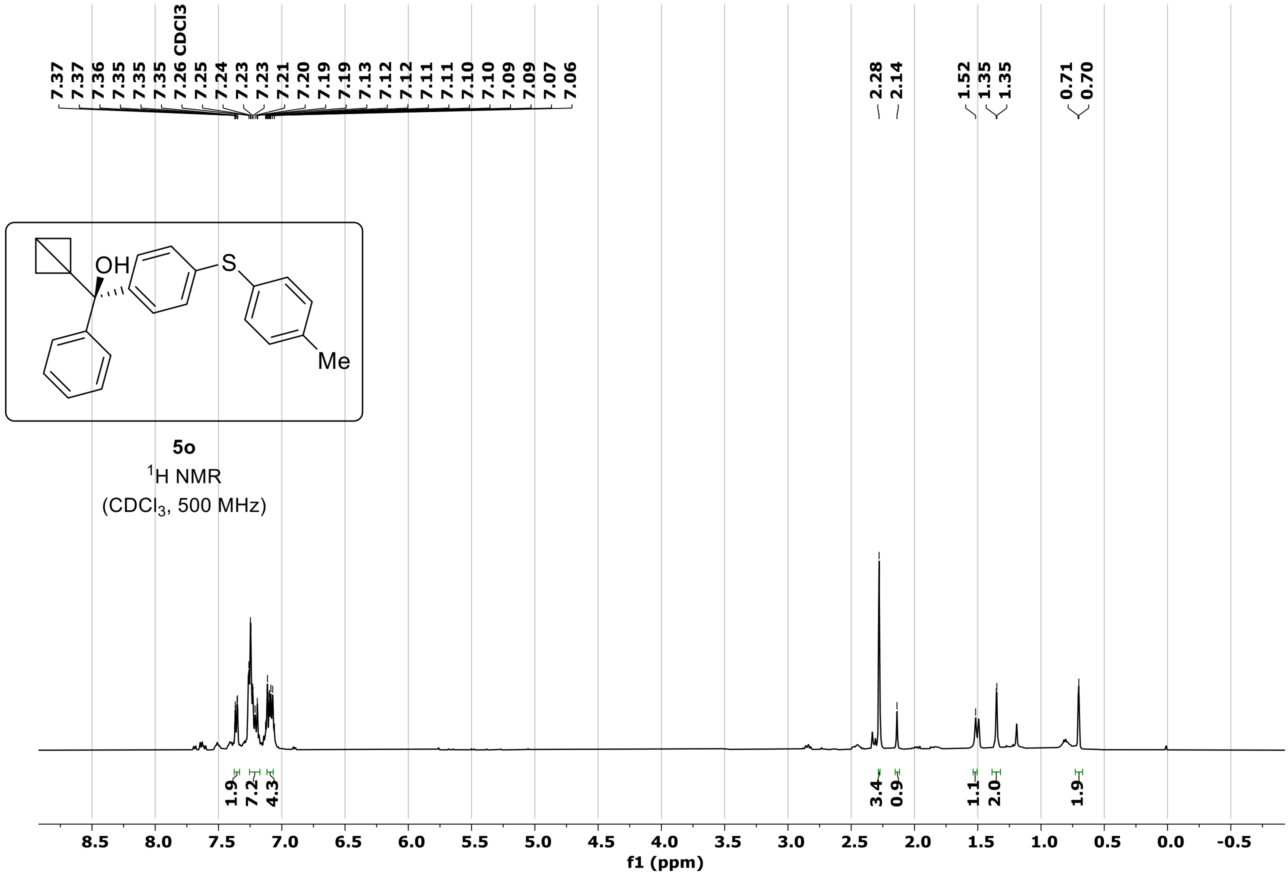

5o
^{1}H NMR
($CDCl_3$, 500 MHz)

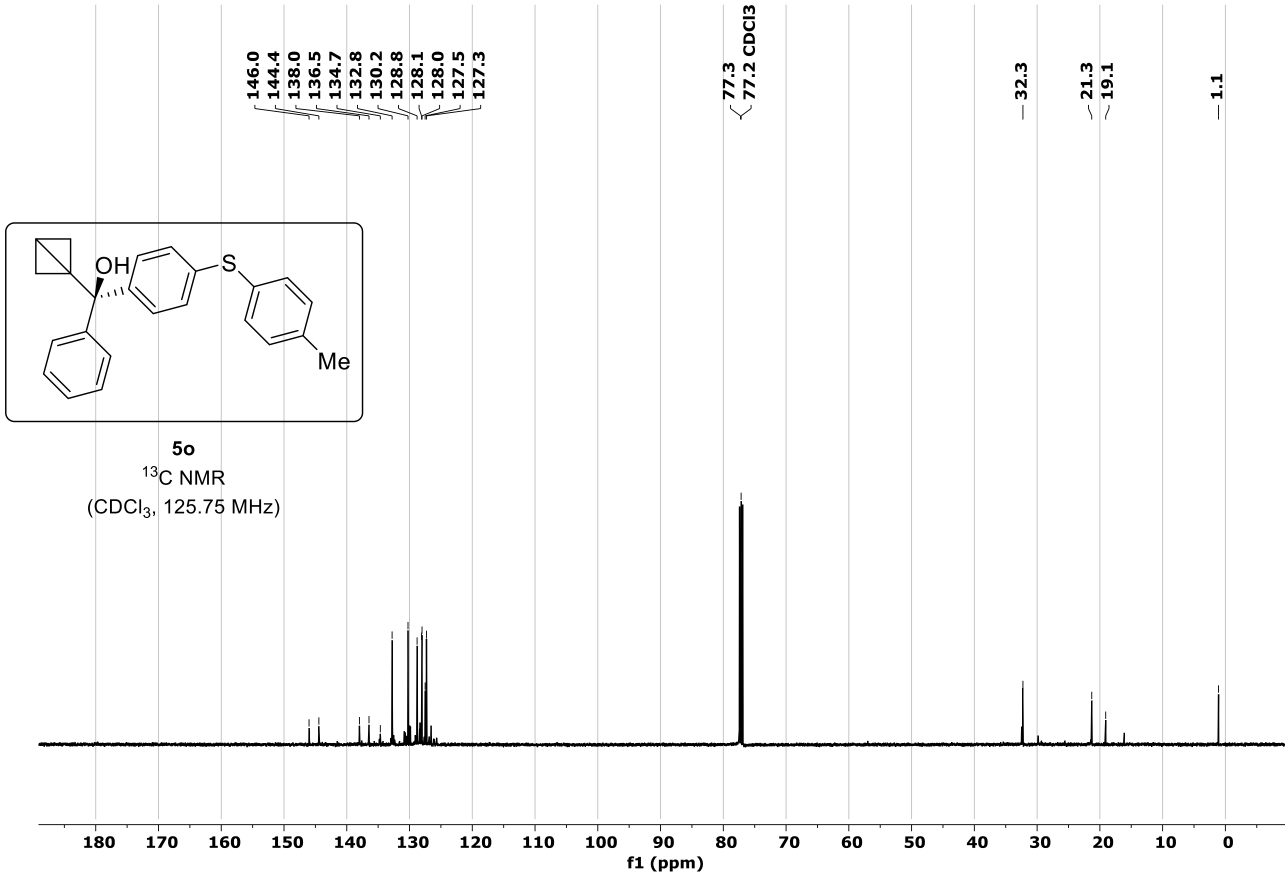

5o
^{13}C NMR
($CDCl_3$, 125.75 MHz)

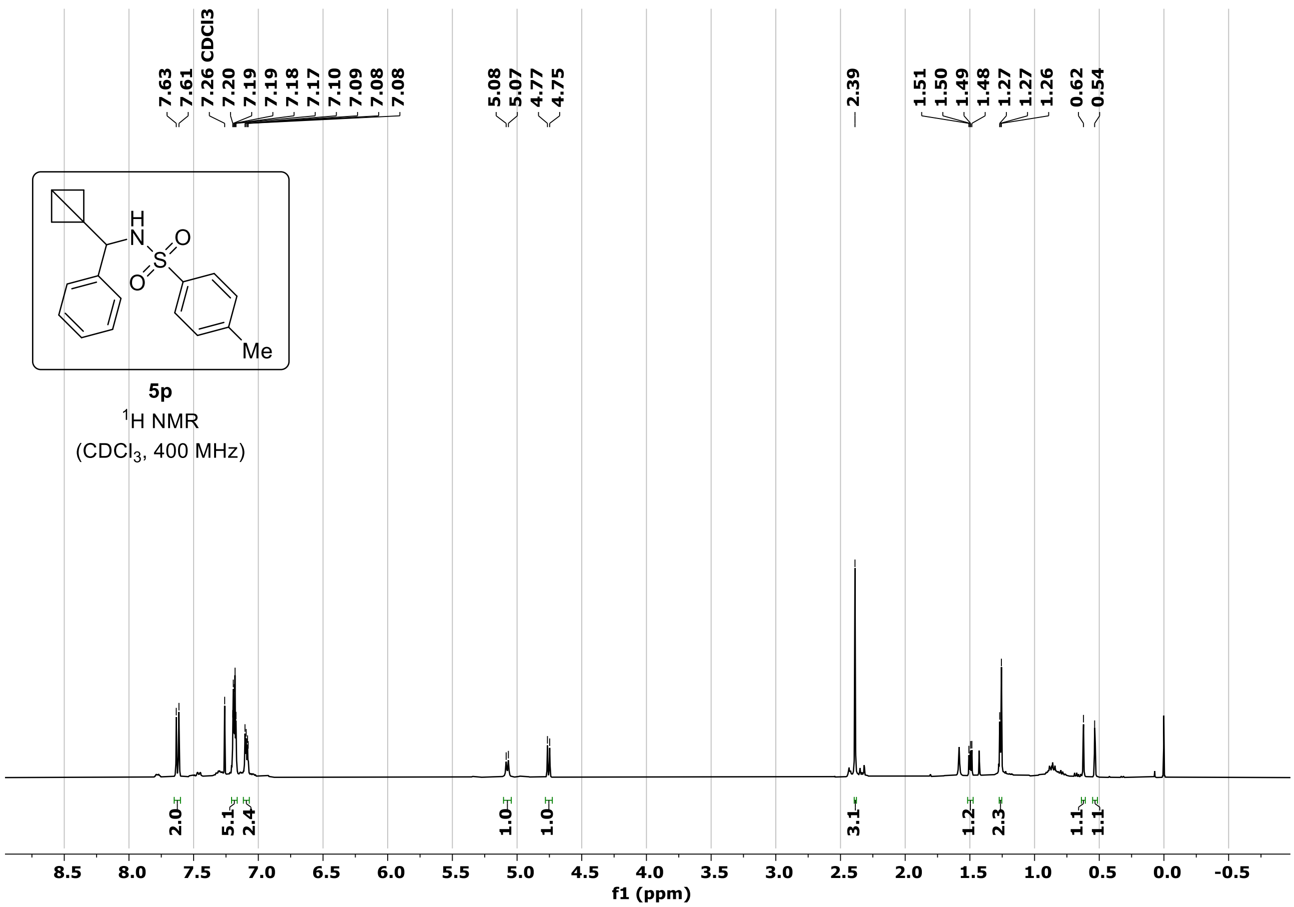
7.63
7.61
7.26 CDCl3
7.20
7.19
7.19
7.18
7.17
7.10
7.09
7.08
7.08
5.08
5.07
4.77
4.75
2.39
1.51
1.50
1.49
1.48
1.27
1.27
1.26
0.62
0.54
H
N
S
O
O
Me
5p
1H NMR
(CDCl3, 400 MHz)
2.0
5.1
2.4
1.0
1.0
3.1
1.2
2.3
1.1
1.1
8.5 8.0 7.5 7.0 6.5 6.0 5.5 5.0 4.5 4.0 3.5 3.0 2.5 2.0 1.5 1.0 0.5 0.0 -0.5
f1 (ppm)

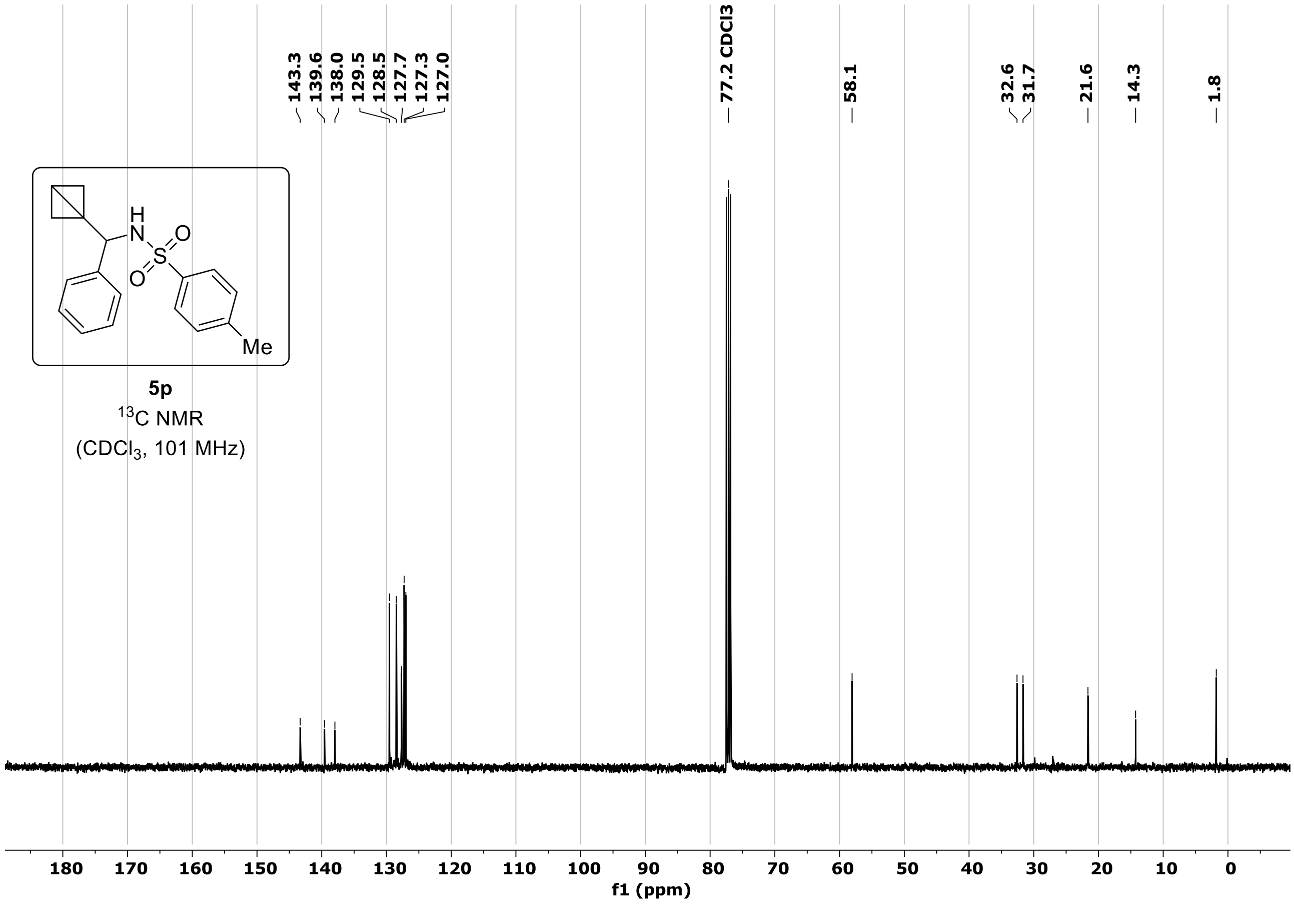
143.3
139.6
138.0
129.5
128.5
127.7
127.3
127.0
77.2 CDCl3
58.1
32.6
31.7
21.6
14.3
1.8
H
N
S
O
O
Me
5p
13C NMR
(CDCl3, 101 MHz)
180 170 160 150 140 130 120 110 100 90 80 70 60 50 40 30 20 10 0
f1 (ppm)

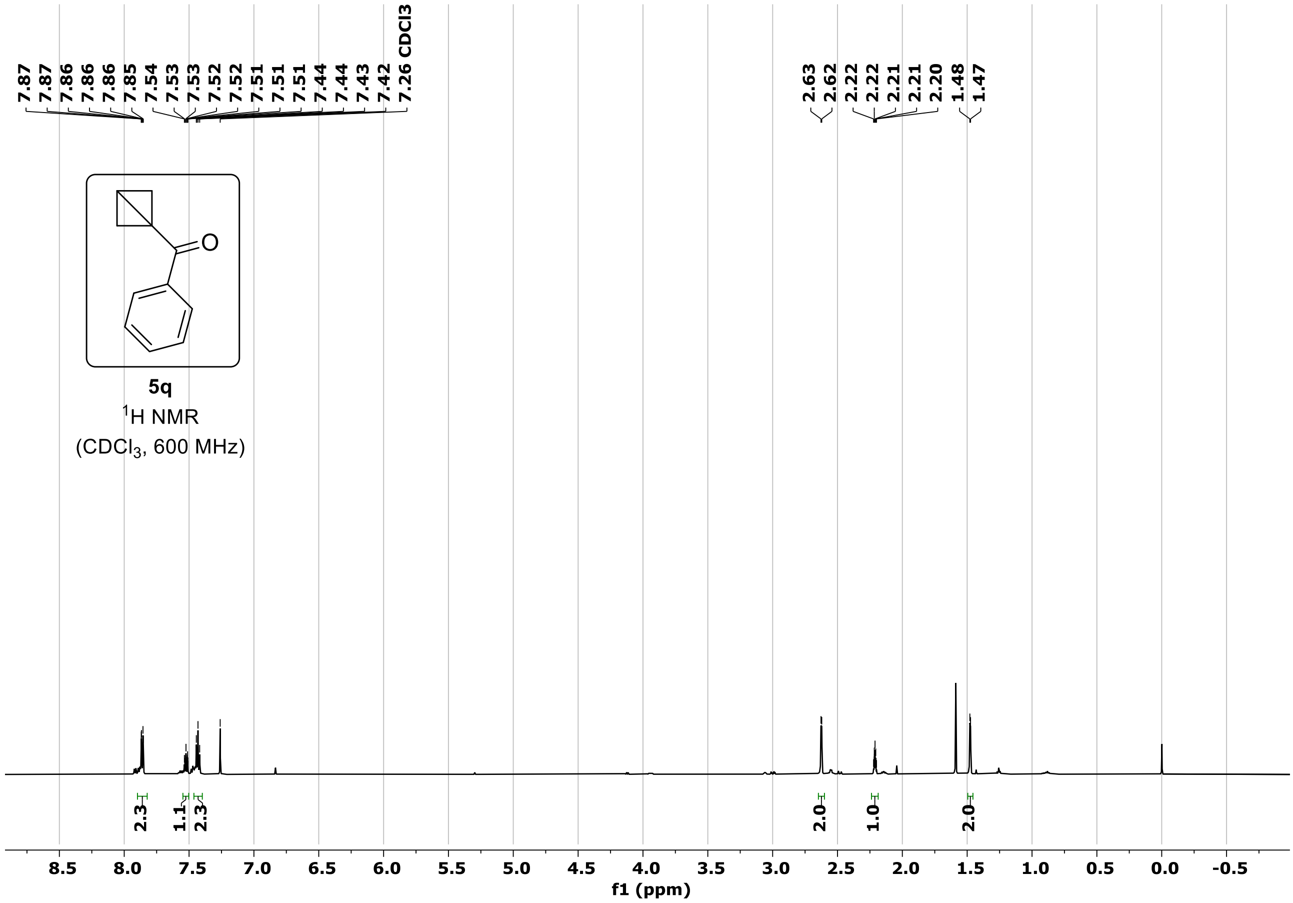
7.87
7.87
7.86
7.86
7.85
7.54
7.53
7.53
7.52
7.52
7.51
7.51
7.51
7.44
7.44
7.43
7.42
7.26 CDCl3
2.63
2.62
2.22
2.22
2.21
2.21
2.20
1.48
1.47
O
5q
1H NMR
(CDCl3, 600 MHz)
2.3
1.1
2.3
2.0
1.0
2.0
8.5 8.0 7.5 7.0 6.5 6.0 5.5 5.0 4.5 4.0 3.5 3.0 2.5 2.0 1.5 1.0 0.5 0.0 -0.5
f1 (ppm)

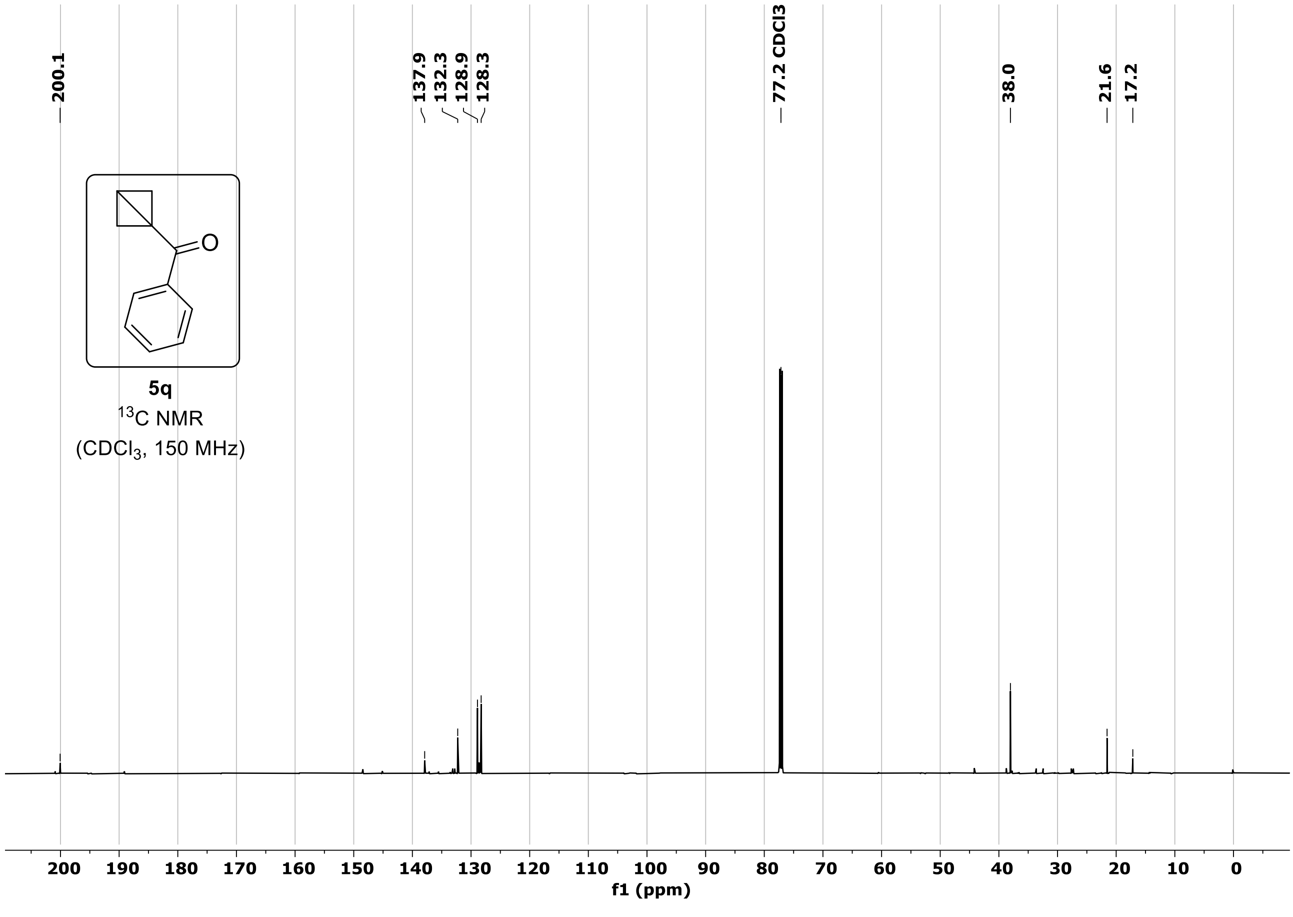
200.1
137.9
132.3
128.9
128.3
77.2 CDCl3
38.0
21.6
17.2
O
5q
13C NMR
(CDCl3, 150 MHz)
200 190 180 170 160 150 140 130 120 110 100 90 80 70 60 50 40 30 20 10 0
f1 (ppm)

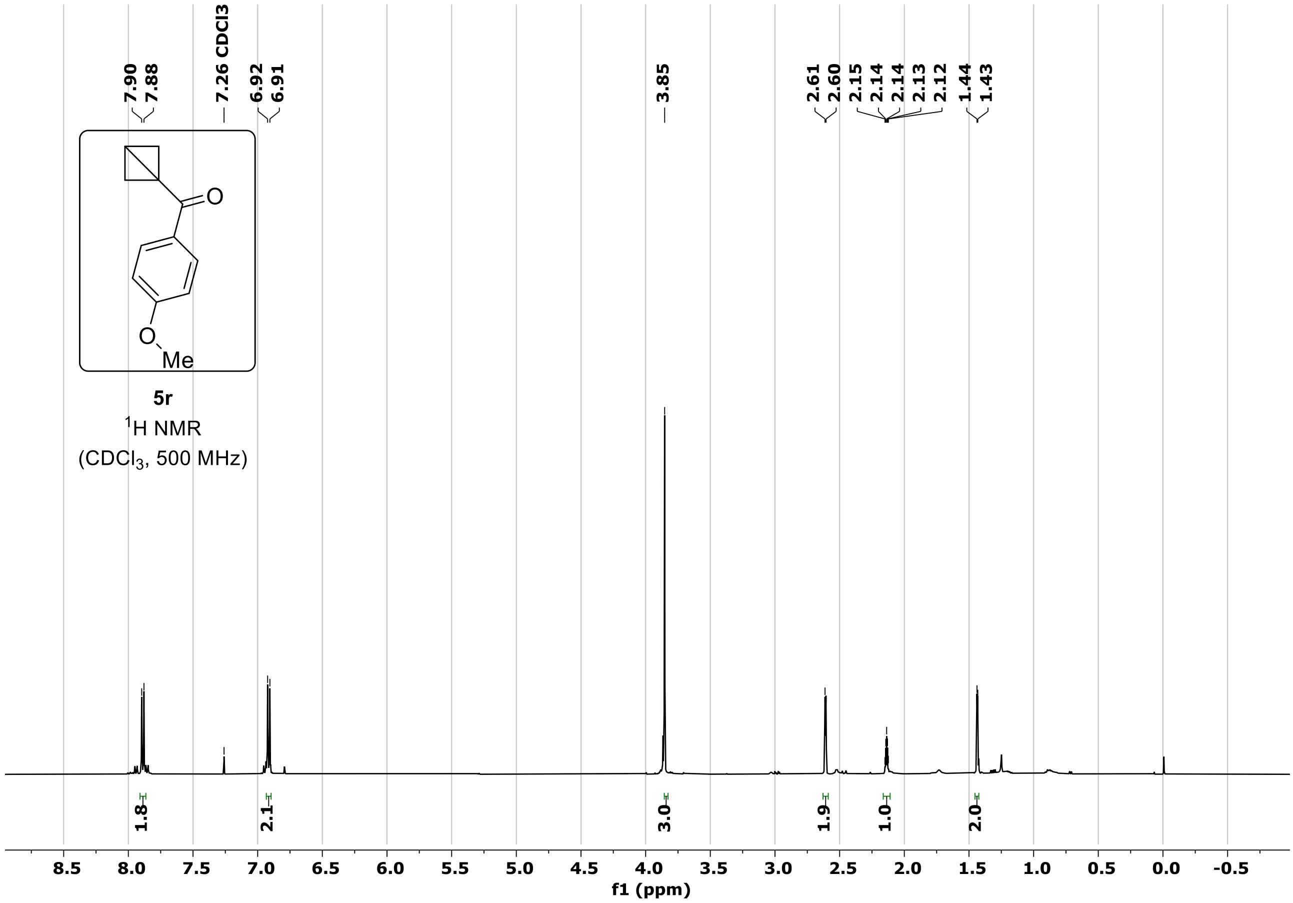
7.90
7.88
7.26 CDCl3
6.92
6.91
3.85
2.61
2.60
2.15
2.14
2.14
2.13
2.12
1.44
1.43
O
Me
5r
1H NMR
(CDCl3, 500 MHz)
1.8
2.1
3.0
1.9
1.0
2.0
8.5 8.0 7.5 7.0 6.5 6.0 5.5 5.0 4.5 4.0 3.5 3.0 2.5 2.0 1.5 1.0 0.5 0.0 -0.5
f1 (ppm)

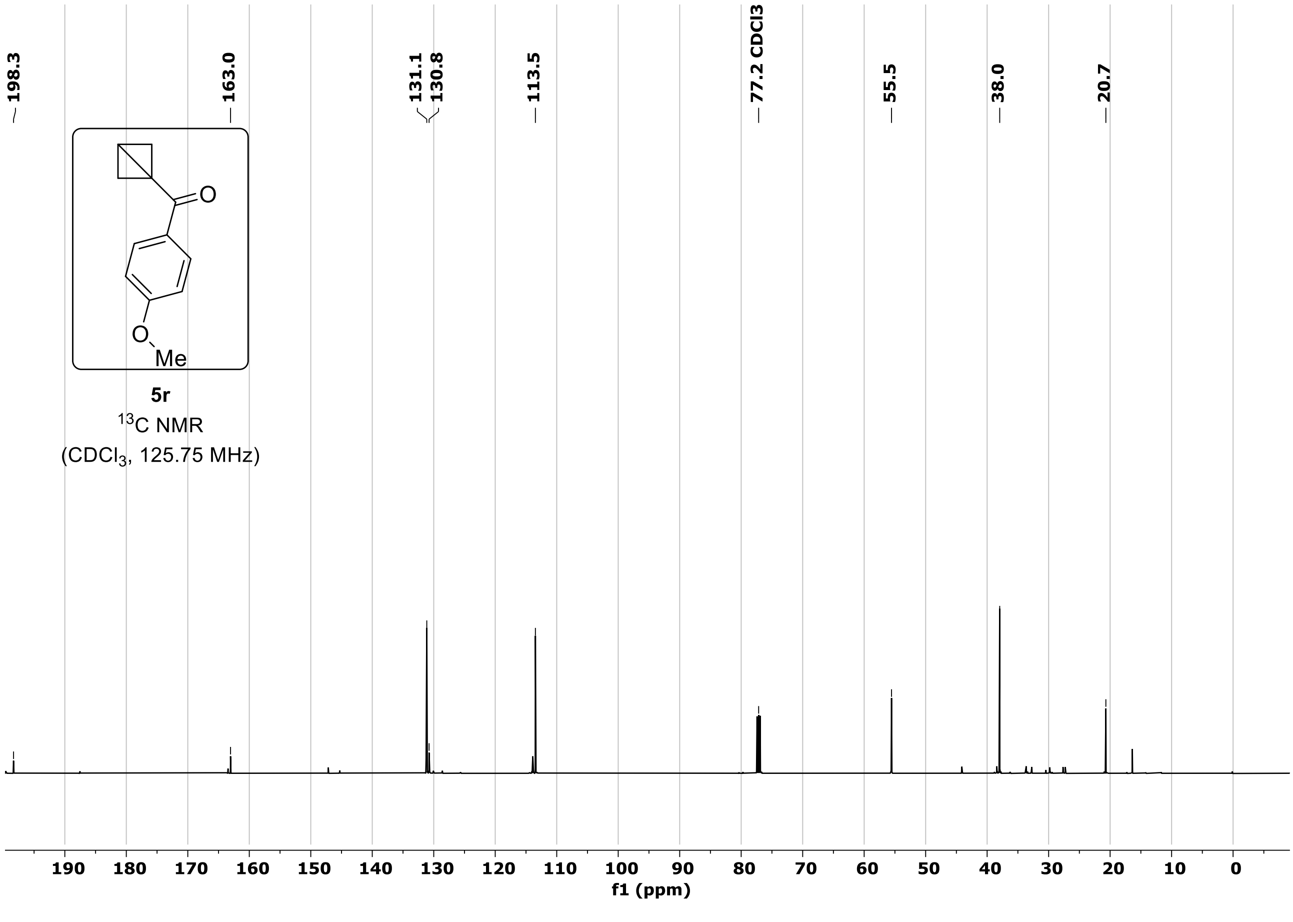
198.3
163.0
131.1
130.8
113.5
77.2 CDCl3
55.5
38.0
20.7
O
Me
5r
13C NMR
(CDCl3, 125.75 MHz)
190 180 170 160 150 140 130 120 110 100 90 80 70 60 50 40 30 20 10 0
f1 (ppm)

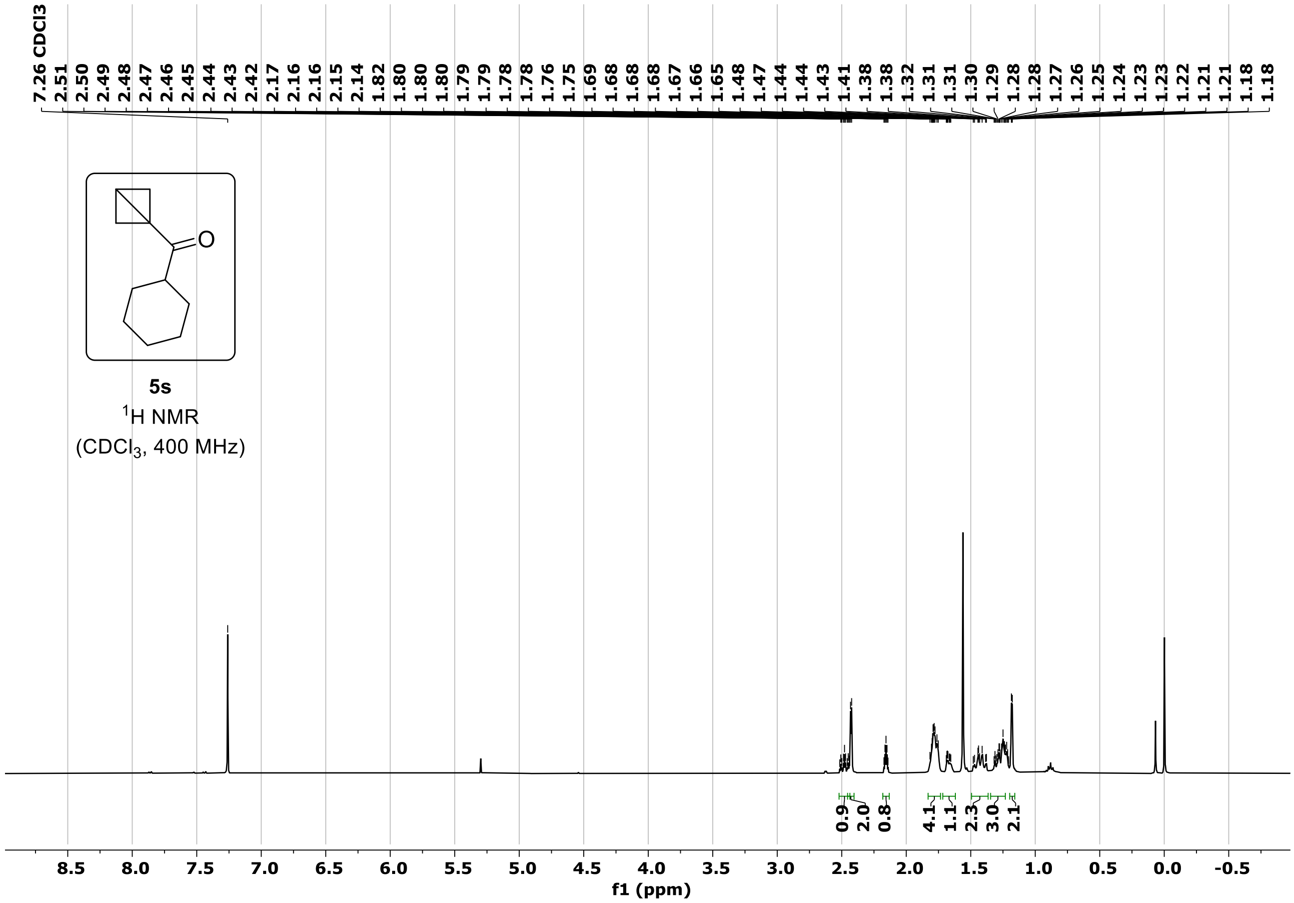
7.26 CDCl3
2.51
2.50
2.49
2.48
2.47
2.46
2.45
2.44
2.43
2.42
2.17
2.16
2.16
2.15
2.14
1.82
1.80
1.80
1.80
1.79
1.79
1.78
1.78
1.76
1.75
1.69
1.68
1.68
1.68
1.67
1.66
1.65
1.48
1.47
1.44
1.44
1.43
1.41
1.38
1.38
1.32
1.31
1.31
1.30
1.29
1.28
1.28
1.27
1.26
1.25
1.24
1.23
1.23
1.22
1.21
1.21
1.18
1.18
O
5s
^{1}H NMR
(CDCl$_3$, 400 MHz)
0.9
2.0
0.8
4.1
1.1
2.3
3.0
2.1
8.5 8.0 7.5 7.0 6.5 6.0 5.5 5.0 4.5 4.0 3.5 3.0 2.5 2.0 1.5 1.0 0.5 0.0 -0.5
f1 (ppm)

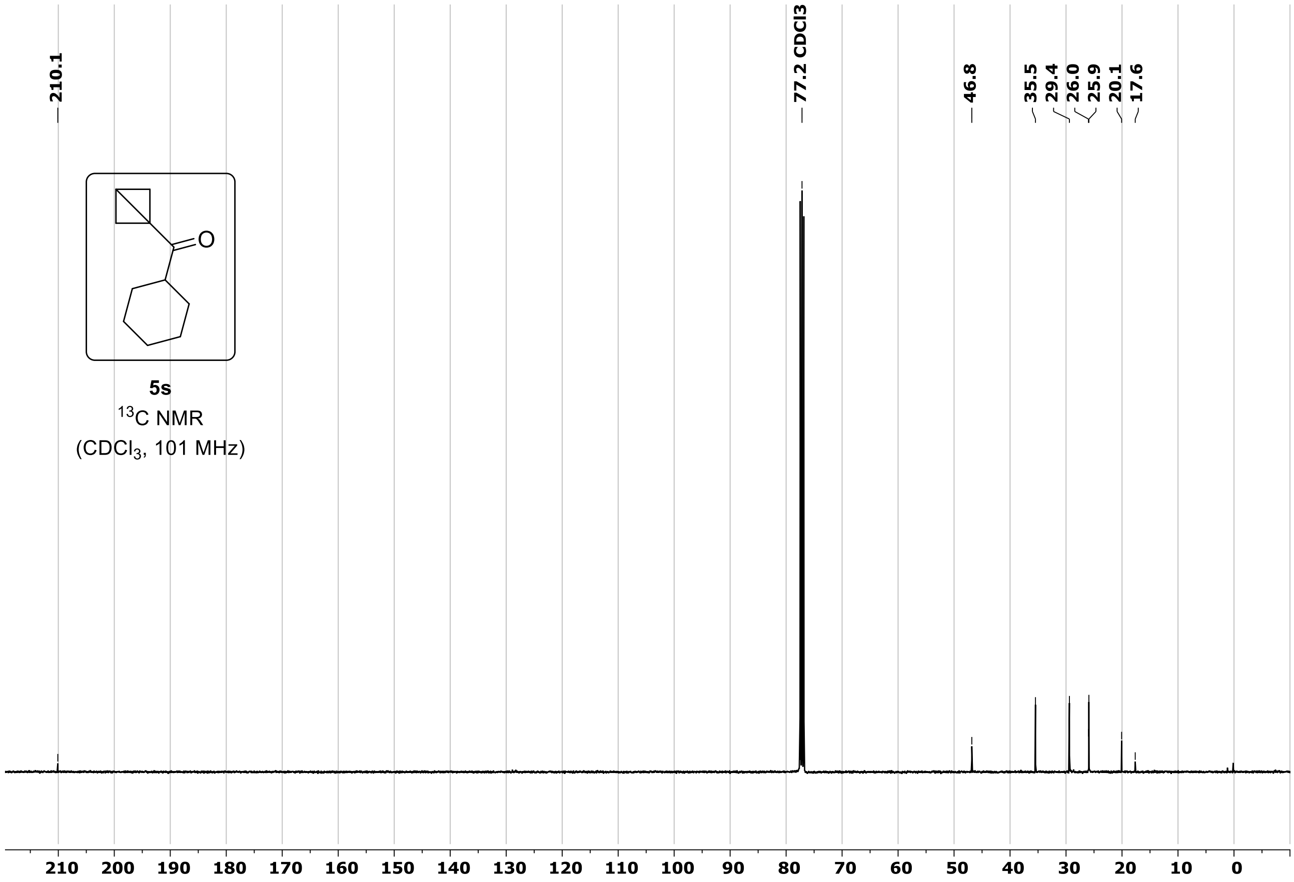
210.1
77.2 CDCl3
46.8
35.5
29.4
26.0
25.9
20.1
17.6
O
5s
^{13}C NMR
(CDCl$_3$, 101 MHz)
210 200 190 180 170 160 150 140 130 120 110 100 90 80 70 60 50 40 30 20 10 0
f1 (ppm)

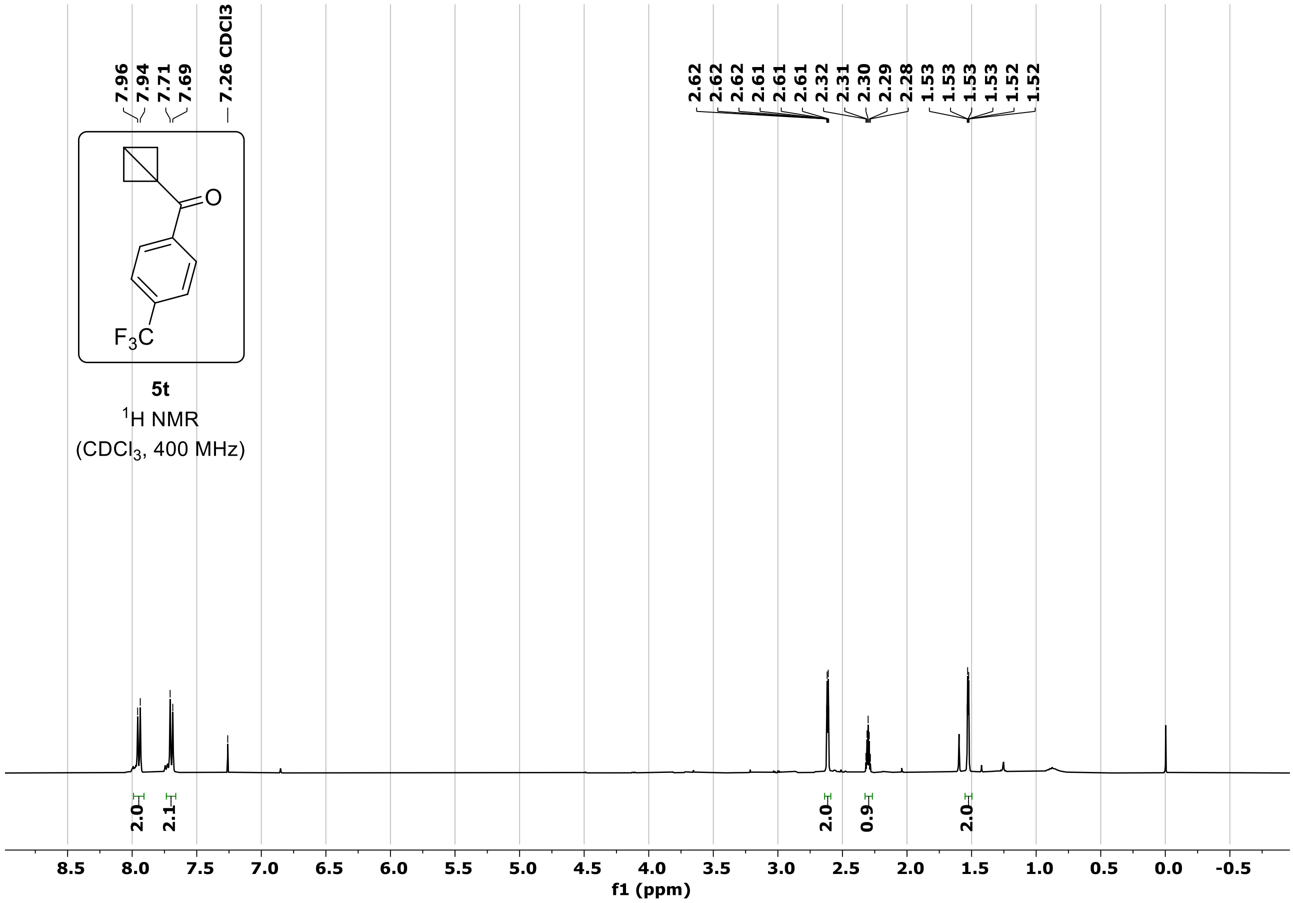

7.96
7.94
7.71
7.69
7.26 CDCl3
2.62
2.62
2.62
2.61
2.61
2.61
2.32
2.31
2.30
2.29
2.28
1.53
1.53
1.53
1.53
1.52
1.52
O
F3C
5t
1H NMR
(CDCl3, 400 MHz)
2.0
2.1
2.0
0.9
2.0
8.5 8.0 7.5 7.0 6.5 6.0 5.5 5.0 4.5 4.0 3.5 3.0 2.5 2.0 1.5 1.0 0.5 0.0 -0.5
f1 (ppm)

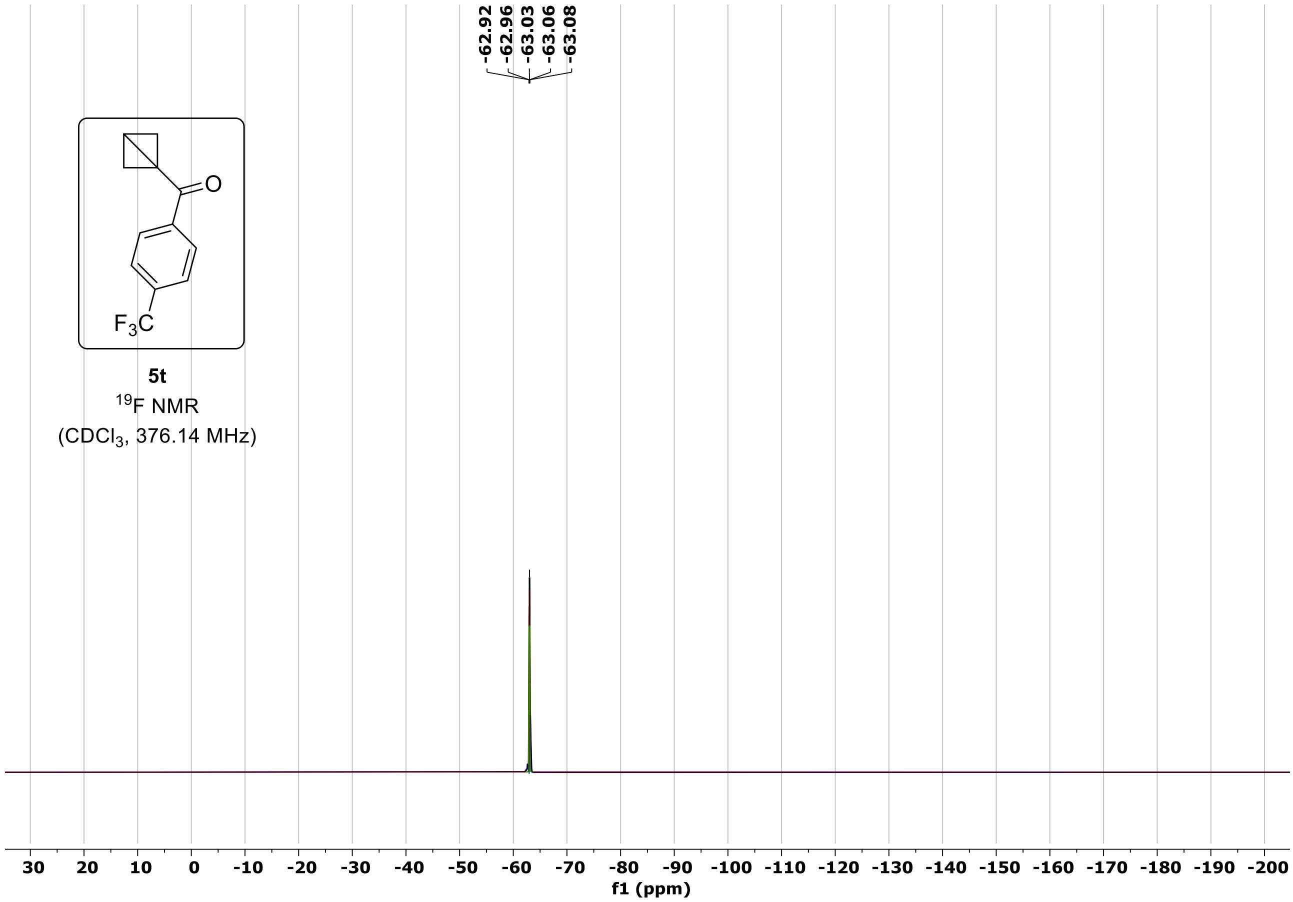

-62.92
-62.96
-63.03
-63.06
-63.08
O
F3C
5t
19F NMR
(CDCl3, 376.14 MHz)
30 20 10 0 -10 -20 -30 -40 -50 -60 -70 -80 -90 -100 -110 -120 -130 -140 -150 -160 -170 -180 -190 -200
f1 (ppm)

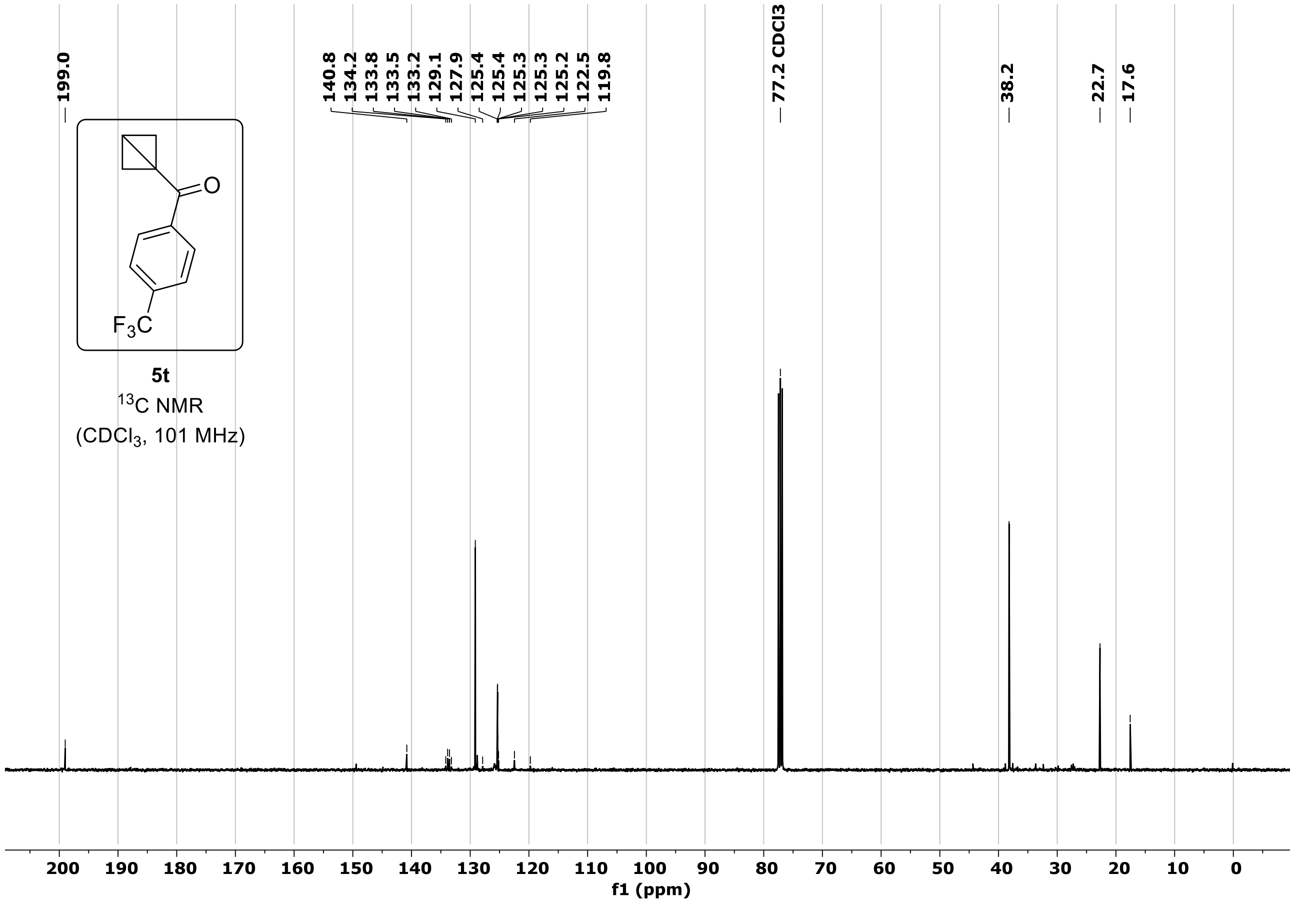
199.0
140.8 134.2 133.8 133.5 133.2 129.1 127.9 125.4 125.4 125.3 125.3 125.2 122.5 119.8
77.2 CDCl3
38.2
22.7
17.6
O
F_3C
5t
^{13}C NMR
($CDCl_3$, 101 MHz)
200 190 180 170 160 150 140 130 120 110 100 90 80 70 60 50 40 30 20 10 0
f1 (ppm)

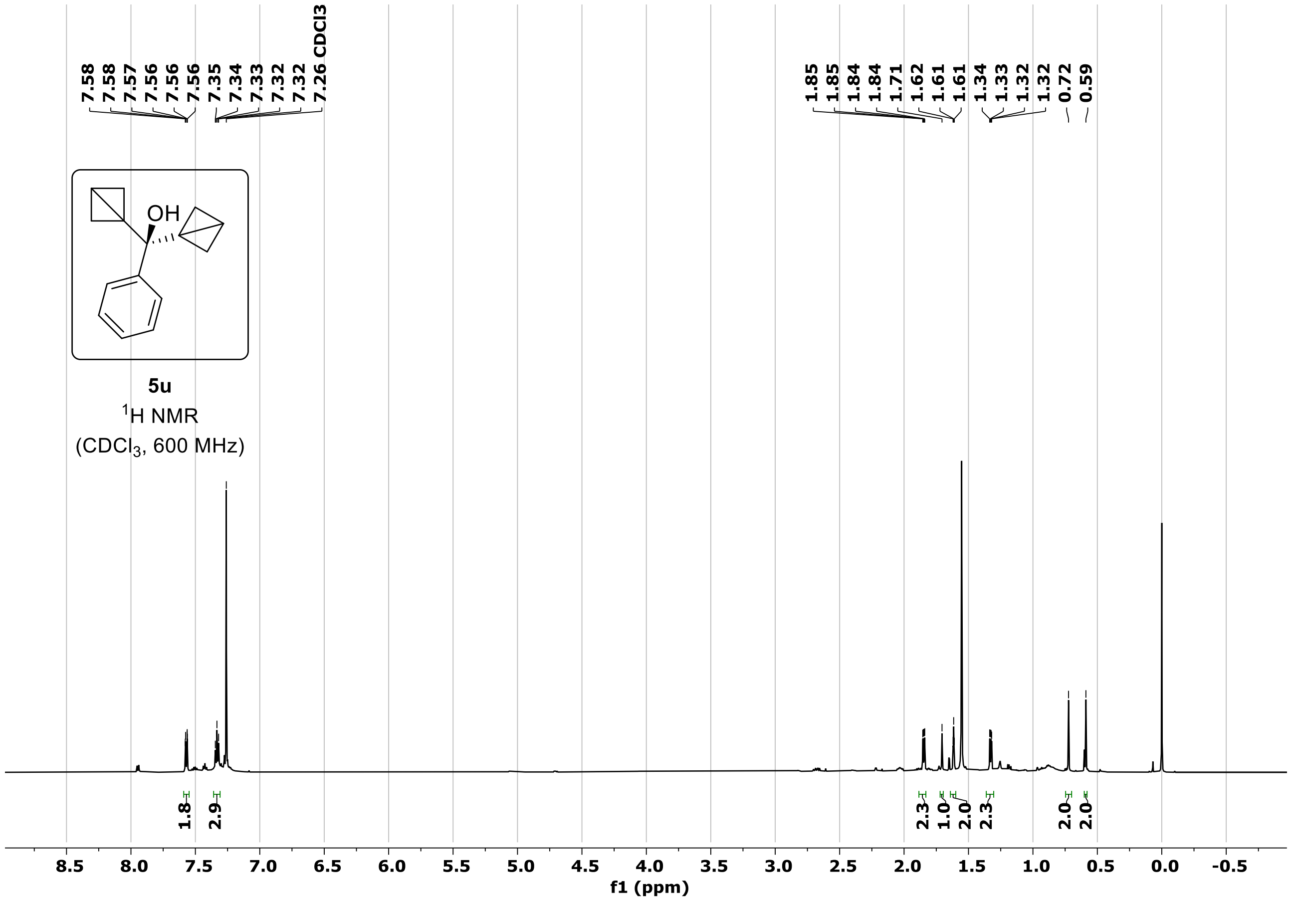
7.58 7.58 7.57 7.56 7.56 7.56 7.35 7.34 7.33 7.32 7.32 7.26 CDCl3
1.85 1.85 1.84 1.84 1.71 1.62 1.61 1.61 1.34 1.33 1.32 1.32 0.72 0.59
OH
5u
^1H NMR
($CDCl_3$, 600 MHz)
1.8 2.9 2.3 1.0 2.0 2.3 2.0 2.0
8.5 8.0 7.5 7.0 6.5 6.0 5.5 5.0 4.5 4.0 3.5 3.0 2.5 2.0 1.5 1.0 0.5 0.0 -0.5
f1 (ppm)

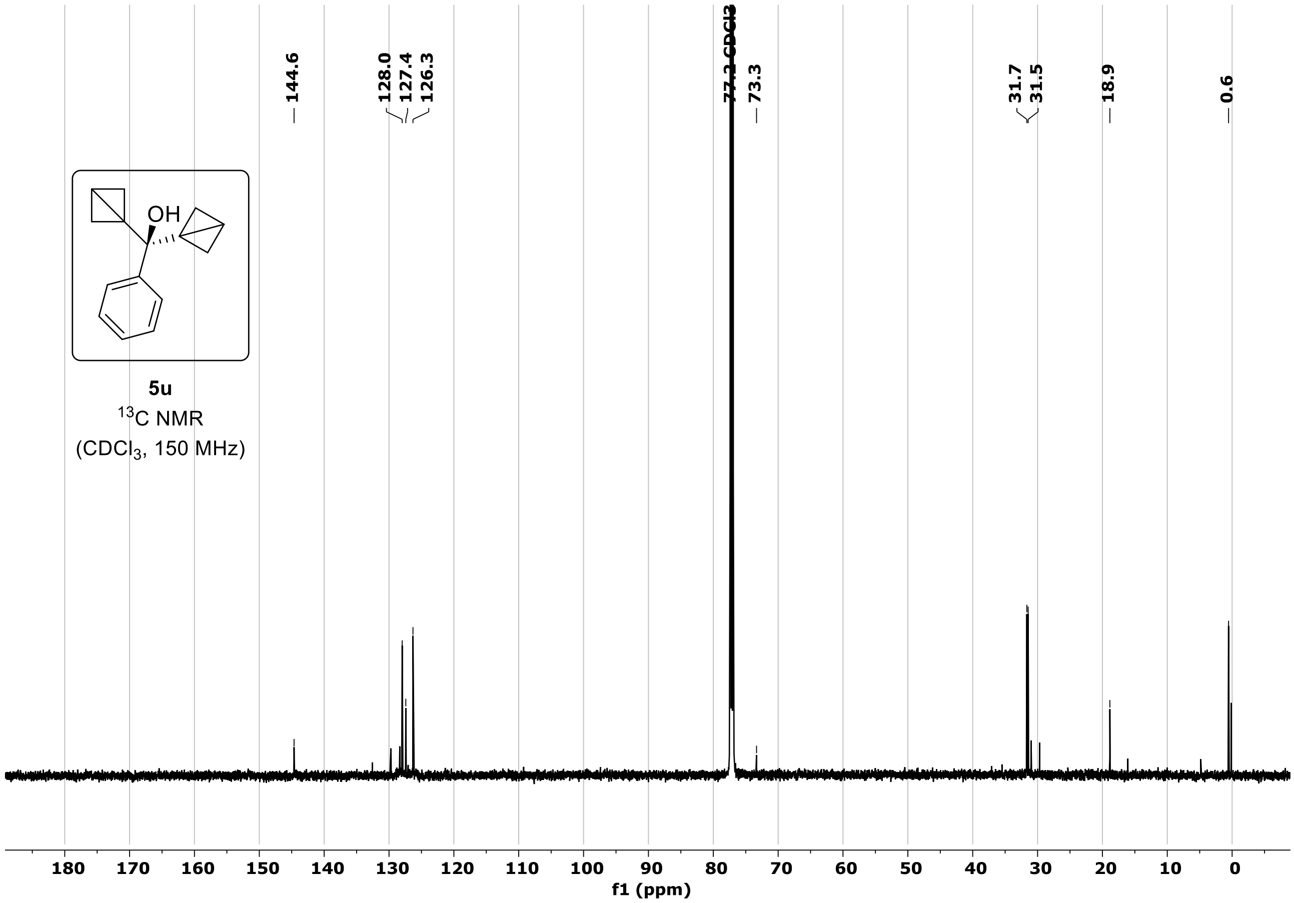
144.6
128.0
127.4
126.3
77.2 CDCl3
73.3
31.7
31.5
18.9
0.6
OH
5u
13C NMR
(CDCl3, 150 MHz)
180 170 160 150 140 130 120 110 100 90 80 70 60 50 40 30 20 10 0
f1 (ppm)

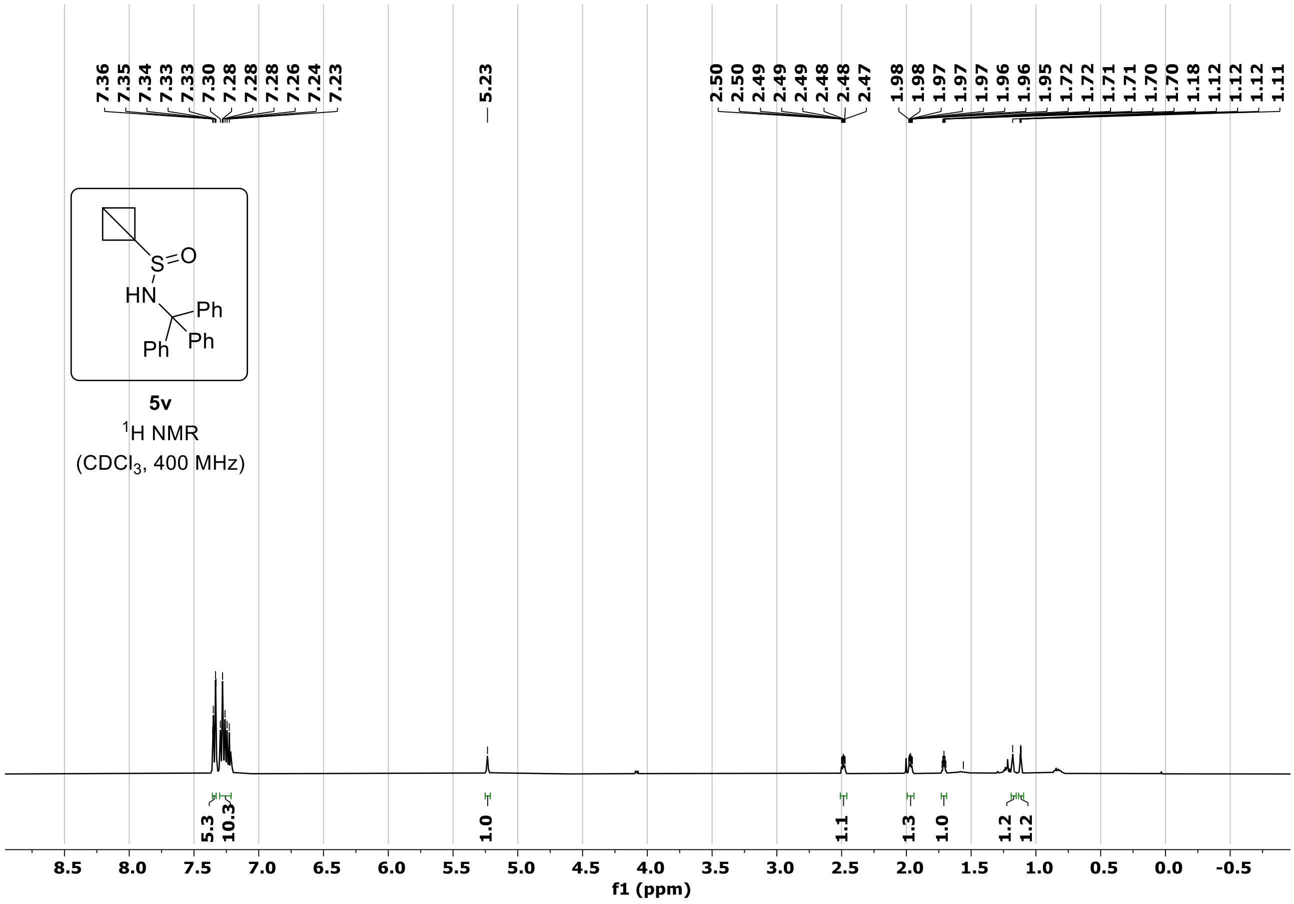
7.36 7.35 7.34 7.33 7.33 7.30 7.28 7.28 7.28 7.26 7.24 7.23
5.23
2.50 2.50 2.49 2.49 2.49 2.48 2.48 2.47
1.98 1.98 1.97 1.97 1.97 1.96 1.96 1.95 1.72 1.72 1.71 1.71 1.70 1.70 1.18 1.12 1.12 1.12 1.11
S=O
HN
Ph
Ph
Ph
5v
1H NMR
(CDCl3, 400 MHz)
5.3
10.3
1.0
1.1
1.3
1.0
1.2
1.2
8.5 8.0 7.5 7.0 6.5 6.0 5.5 5.0 4.5 4.0 3.5 3.0 2.5 2.0 1.5 1.0 0.5 0.0 -0.5
f1 (ppm)

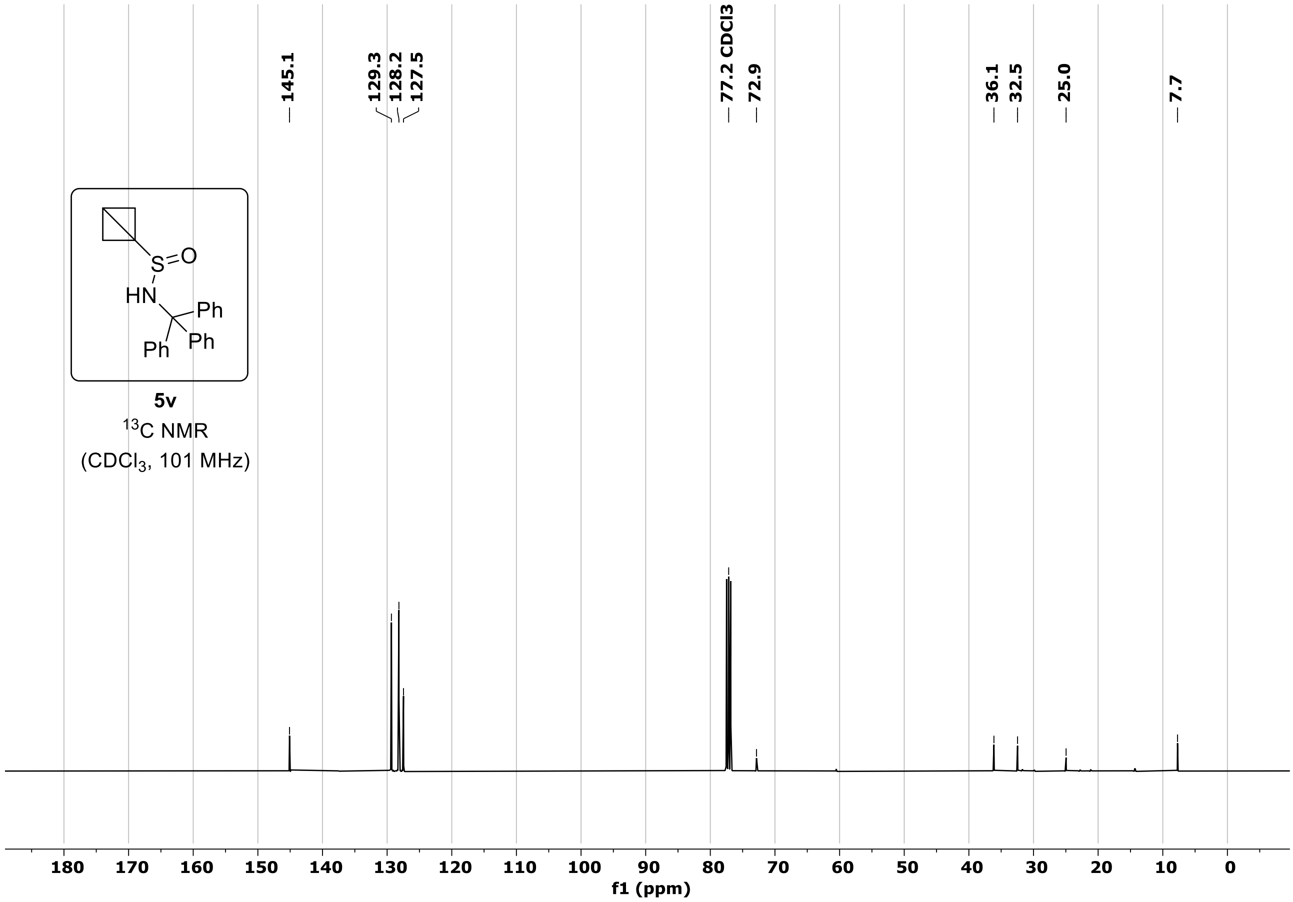
5v
^{13}C NMR
(CDCl$_3$, 101 MHz)
145.1
129.3
128.2
127.5
77.2 CDCl3
72.9
36.1
32.5
25.0
7.7
f1 (ppm)

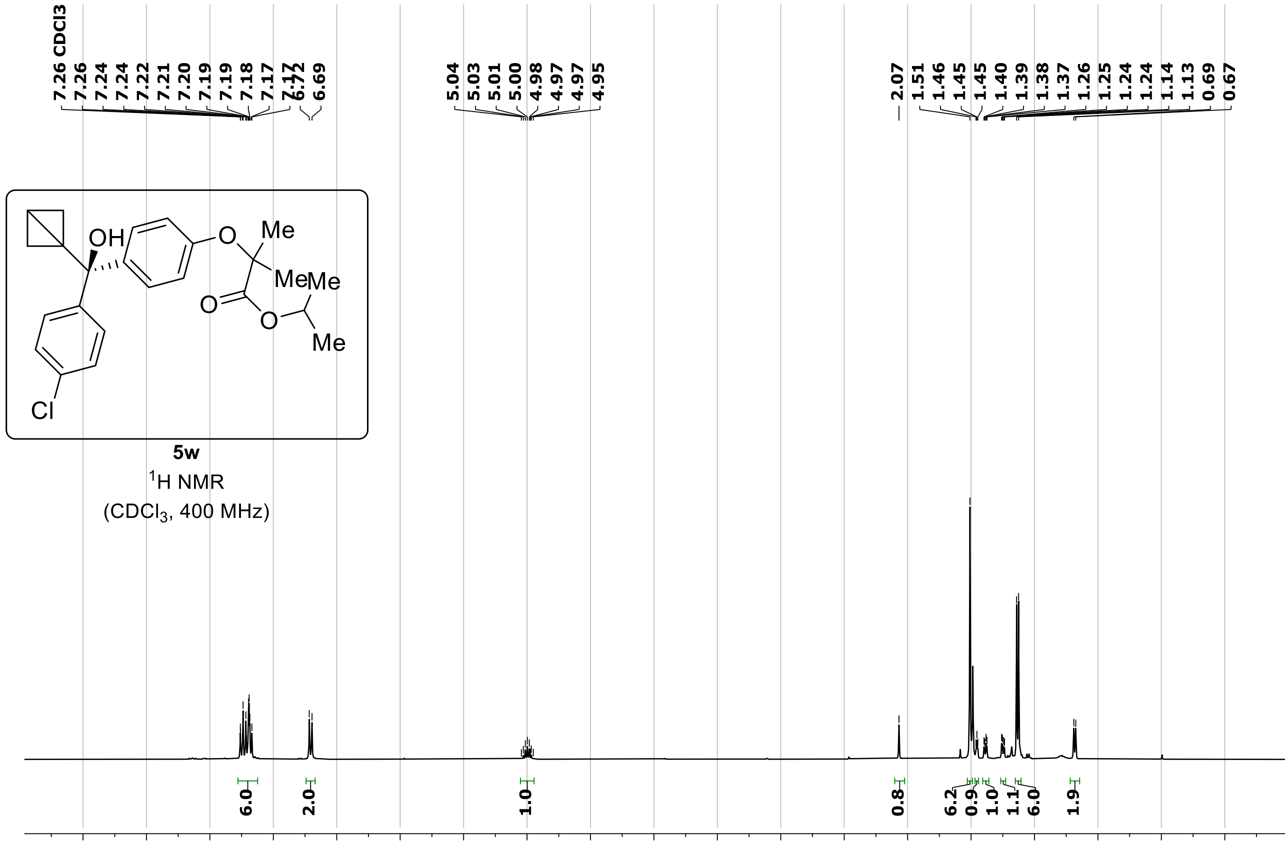
5w
^{1}H NMR
(CDCl$_3$, 400 MHz)
7.26 CDCl3
7.26
7.24
7.24
7.22
7.21
7.20
7.19
7.18
7.17
7.17
6.72
6.69
5.04
5.03
5.01
5.00
4.98
4.97
4.97
4.95
2.07
1.51
1.46
1.45
1.45
1.40
1.39
1.38
1.37
1.26
1.25
1.24
1.24
1.14
1.13
0.69
0.67
6.0
2.0
1.0
0.8
6.2
0.9
1.0
1.1
6.0
1.9
f1 (ppm)

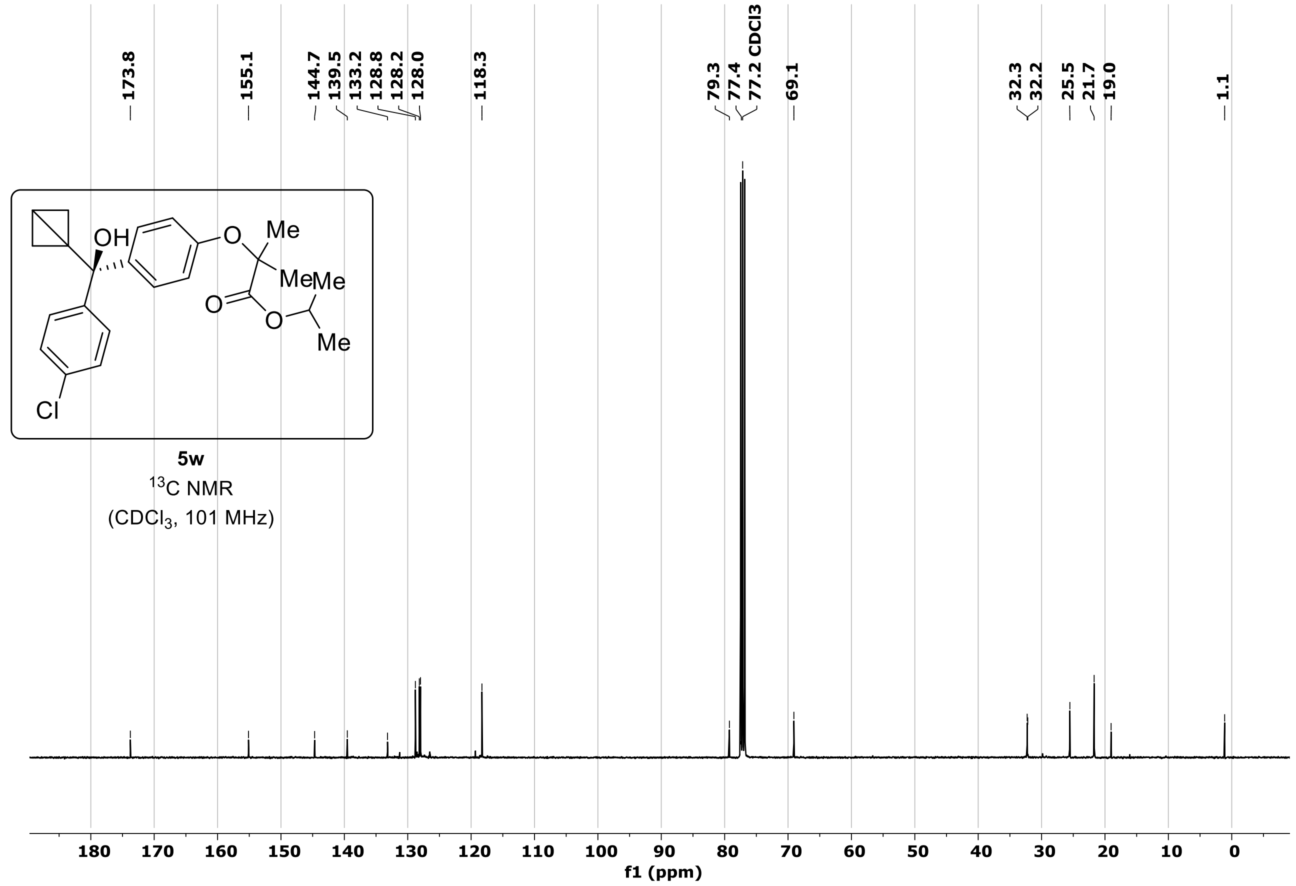

5w

^{13}C NMR

($CDCl_3$, 101 MHz)

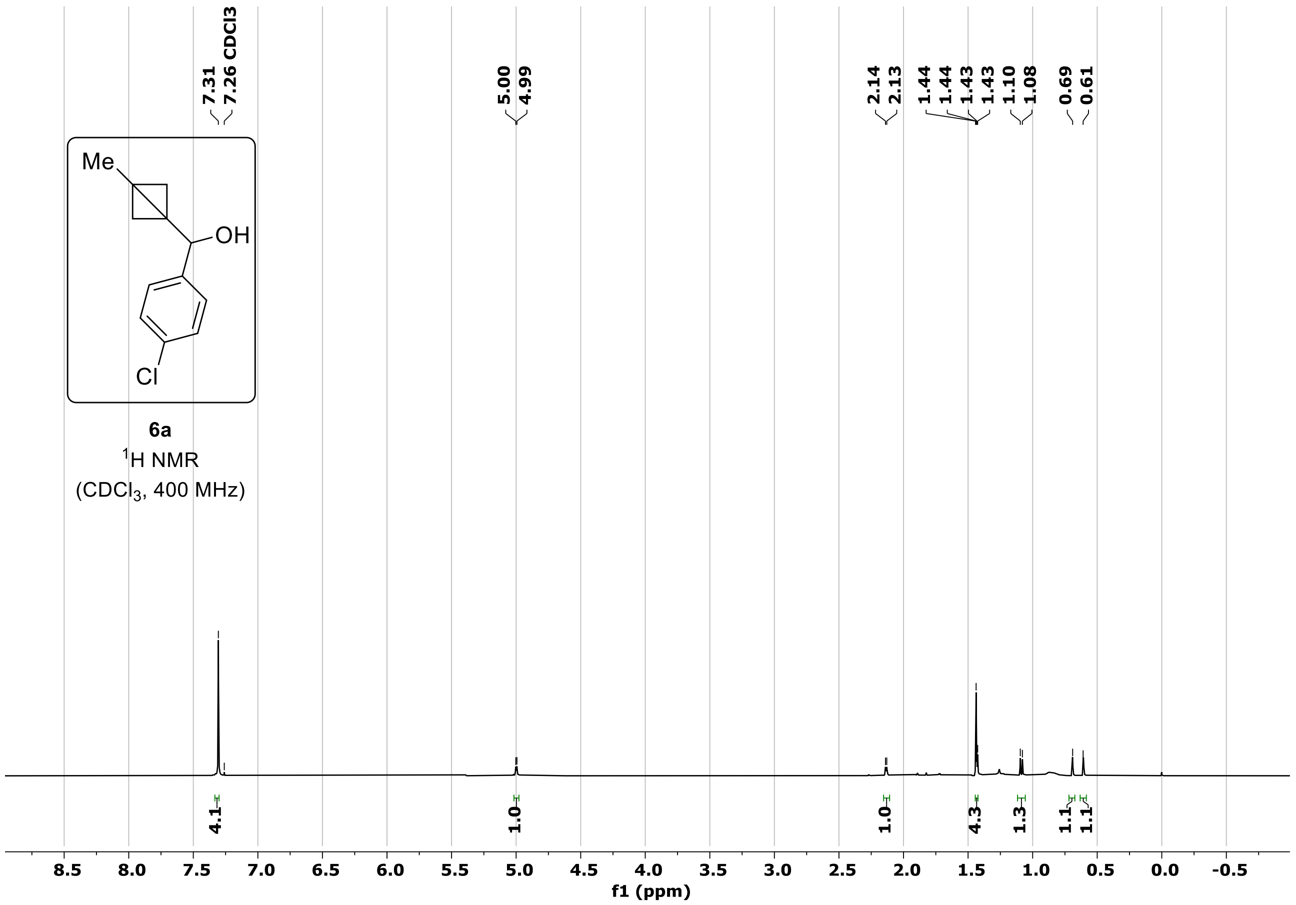

6a

^{1}H NMR

($CDCl_3$, 400 MHz)

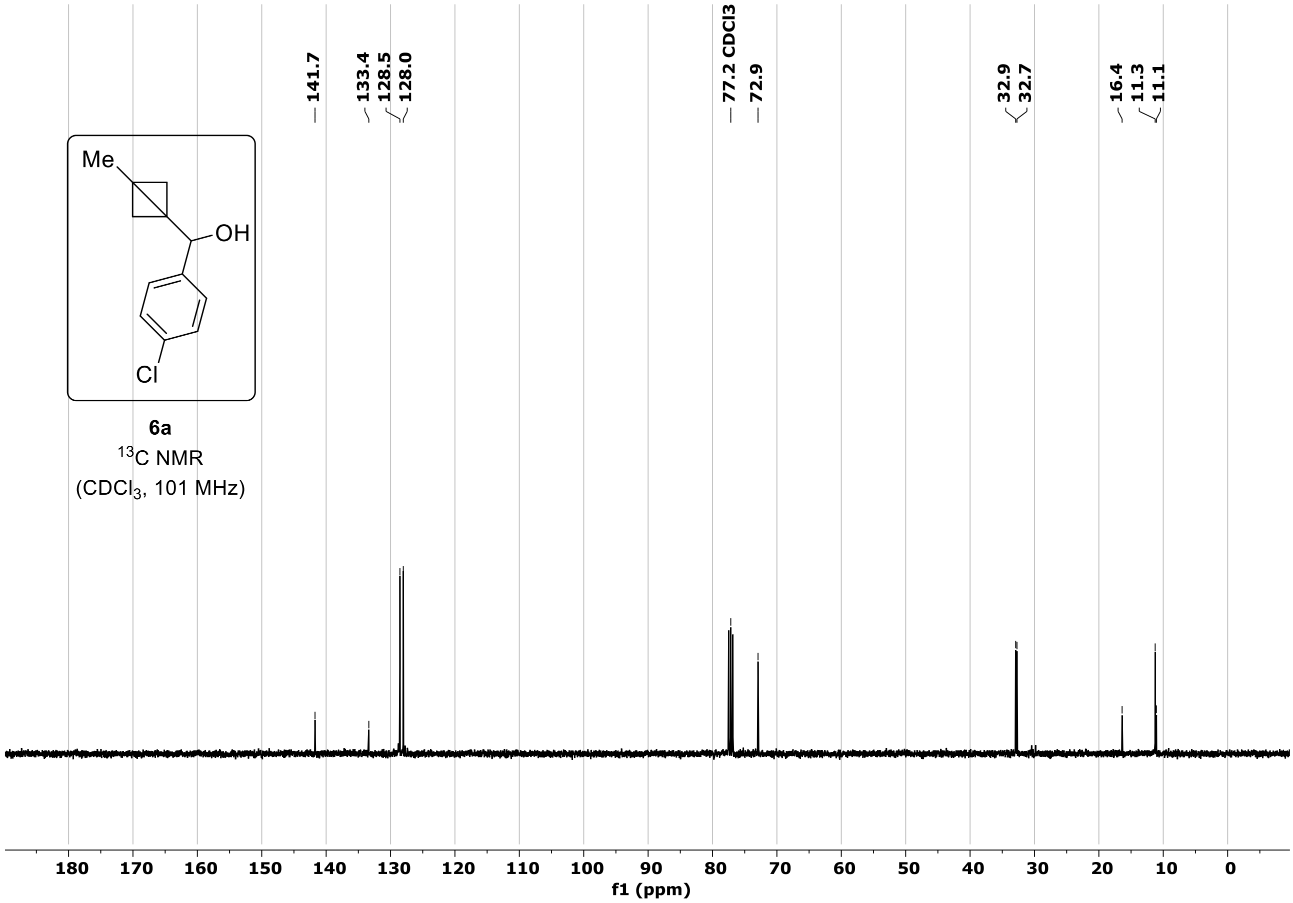
141.7
133.4
128.5
128.0
77.2 CDCl3
72.9
32.9
32.7
16.4
11.3
11.1
Me
OH
Cl
6a
13C NMR
(CDCl3, 101 MHz)
180 170 160 150 140 130 120 110 100 90 80 70 60 50 40 30 20 10 0
f1 (ppm)

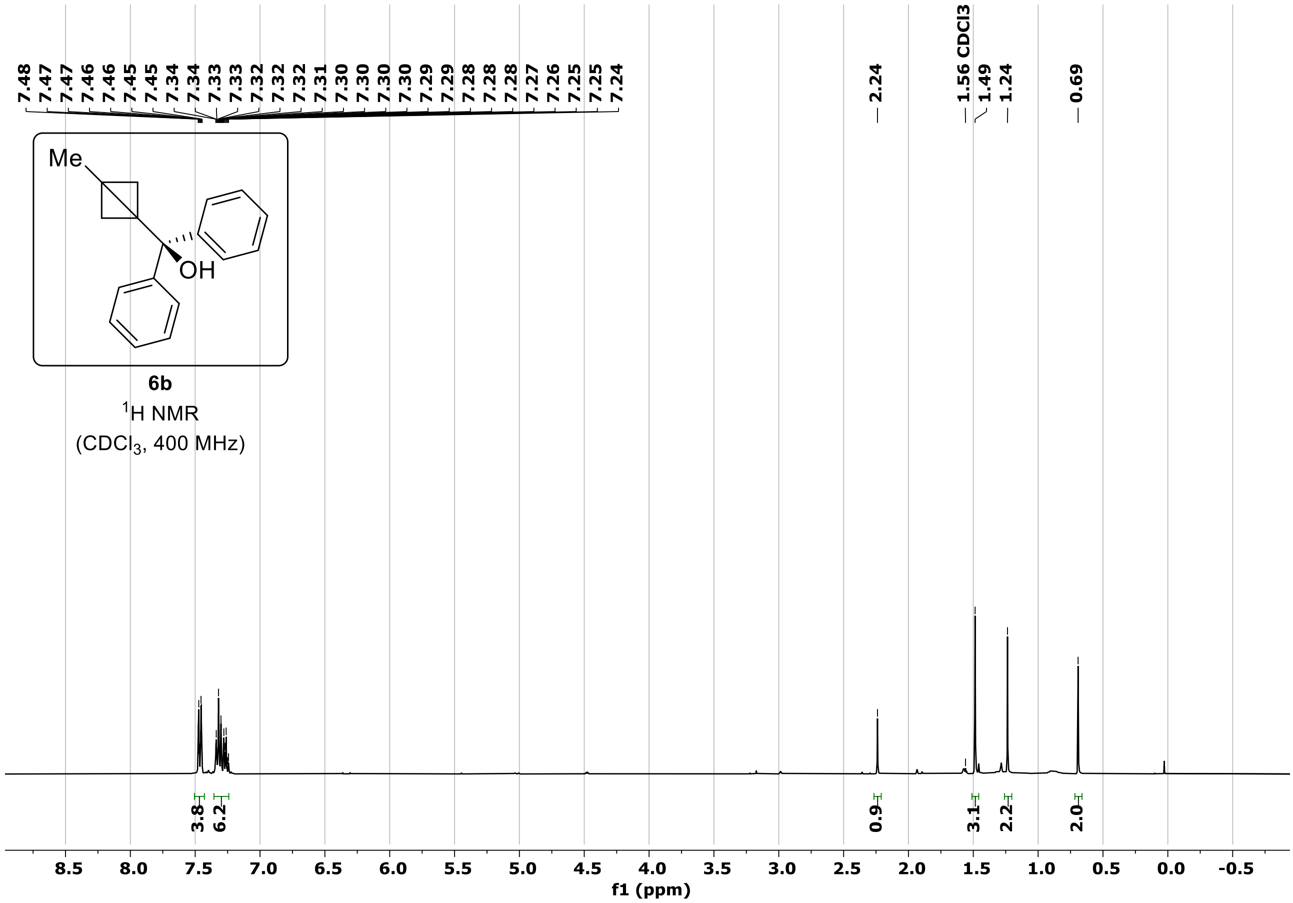
7.48 7.47 7.47 7.46 7.46 7.45 7.45 7.34 7.34 7.33 7.33 7.32 7.32 7.32 7.31 7.30 7.30 7.30 7.30 7.29 7.29 7.28 7.28 7.28 7.27 7.26 7.25 7.25 7.24
2.24
1.56 CDCl3
1.49
1.24
0.69
Me
OH
6b
1H NMR
(CDCl3, 400 MHz)
3.8
6.2
0.9
3.1
2.2
2.0
8.5 8.0 7.5 7.0 6.5 6.0 5.5 5.0 4.5 4.0 3.5 3.0 2.5 2.0 1.5 1.0 0.5 0.0 -0.5
f1 (ppm)

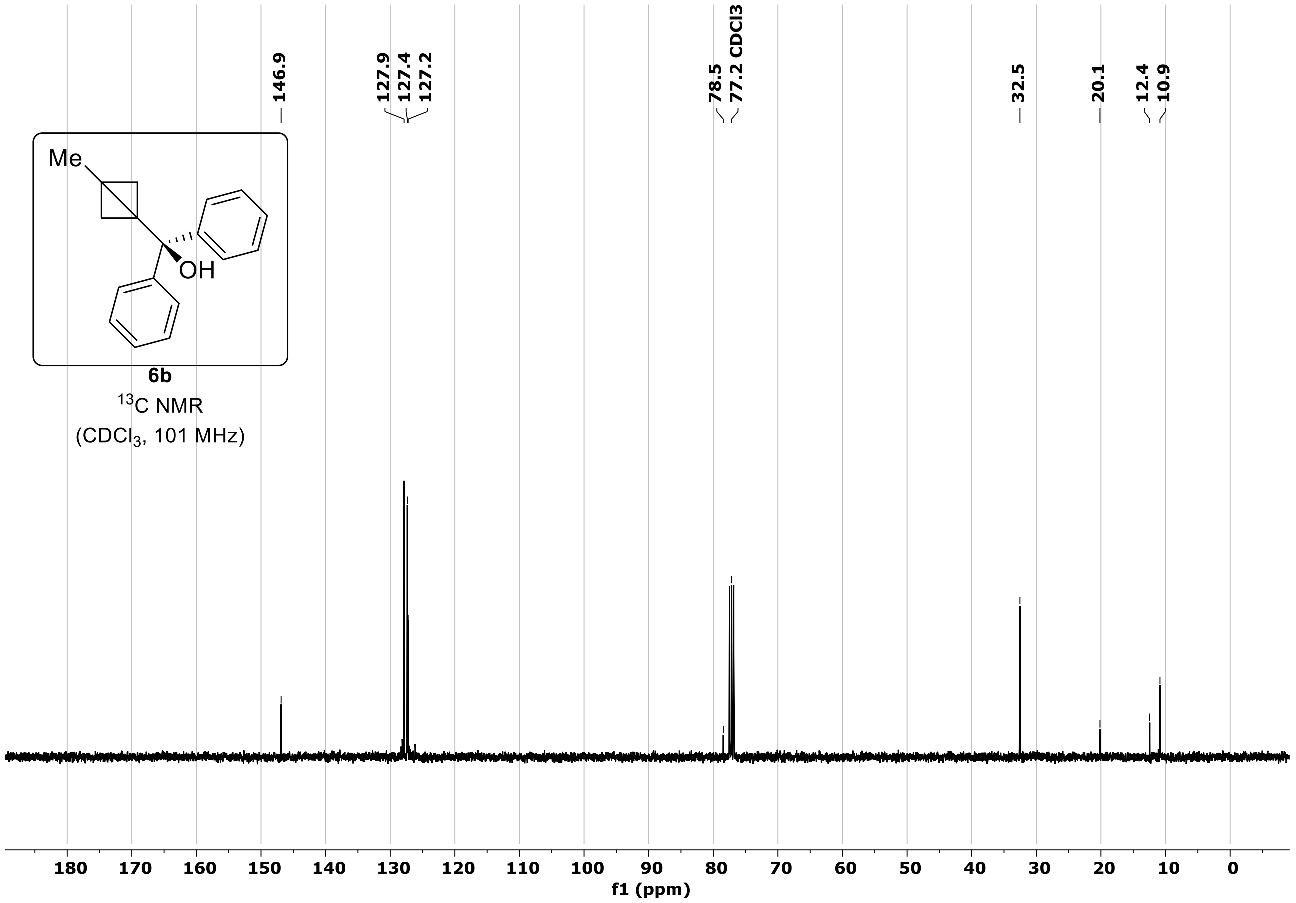
146.9
127.9
127.4
127.2
78.5
77.2 CDCl3
32.5
20.1
12.4
10.9
Me
OH
6b
13C NMR
(CDCl3, 101 MHz)
180 170 160 150 140 130 120 110 100 90 80 70 60 50 40 30 20 10 0
f1 (ppm)

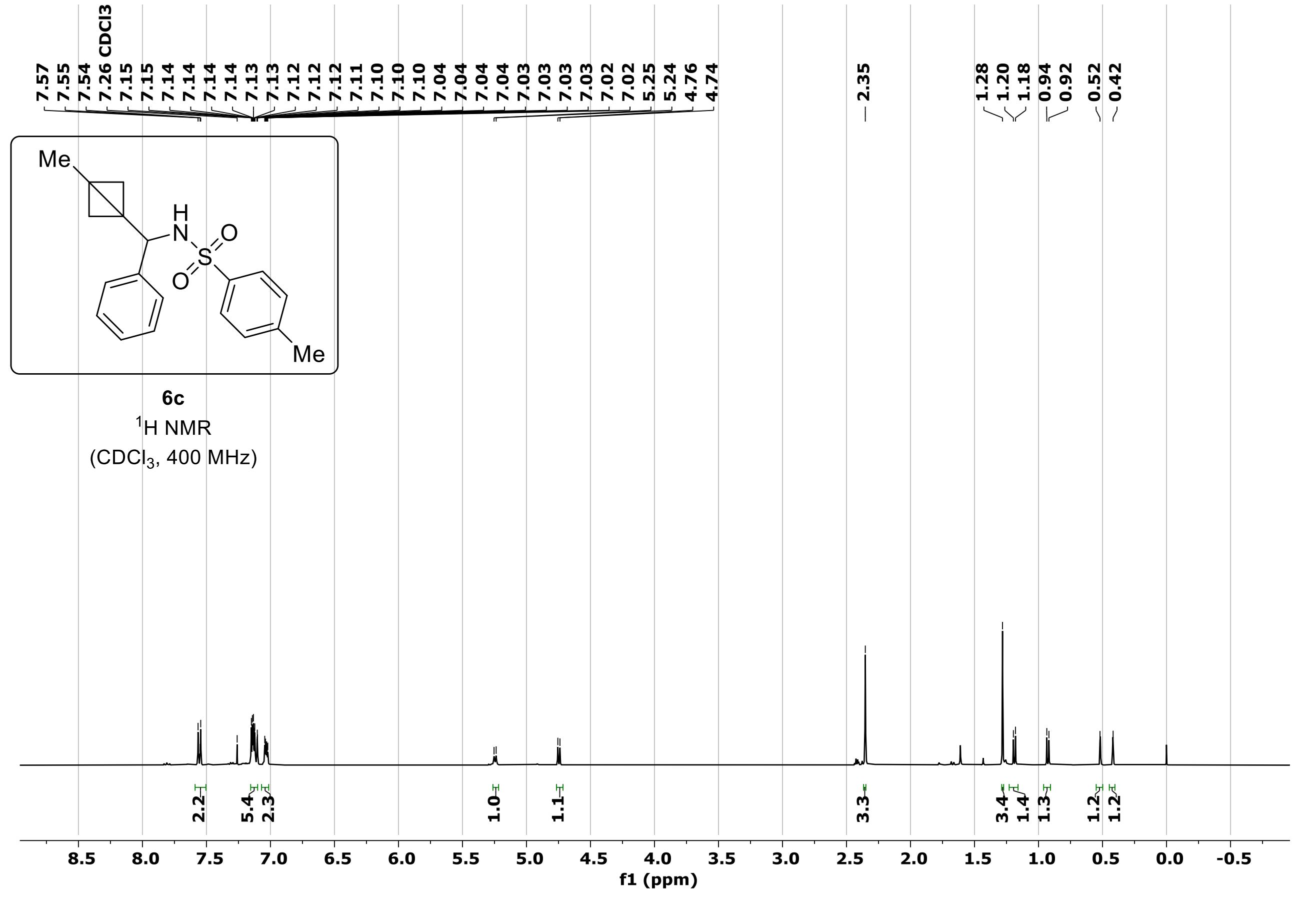
7.57
7.55
7.54
7.26 CDCl3
7.15
7.15
7.14
7.14
7.14
7.13
7.13
7.12
7.12
7.11
7.10
7.10
7.04
7.04
7.04
7.03
7.03
7.03
7.02
7.02
5.25
5.24
4.76
4.74
2.35
1.28
1.20
1.18
0.94
0.92
0.52
0.42
Me
H
N
S
O
O
Me
6c
1H NMR
(CDCl3, 400 MHz)
2.2
5.4
2.3
1.0
1.1
3.3
3.4
1.4
1.3
1.2
1.2
8.5 8.0 7.5 7.0 6.5 6.0 5.5 5.0 4.5 4.0 3.5 3.0 2.5 2.0 1.5 1.0 0.5 0.0 -0.5
f1 (ppm)

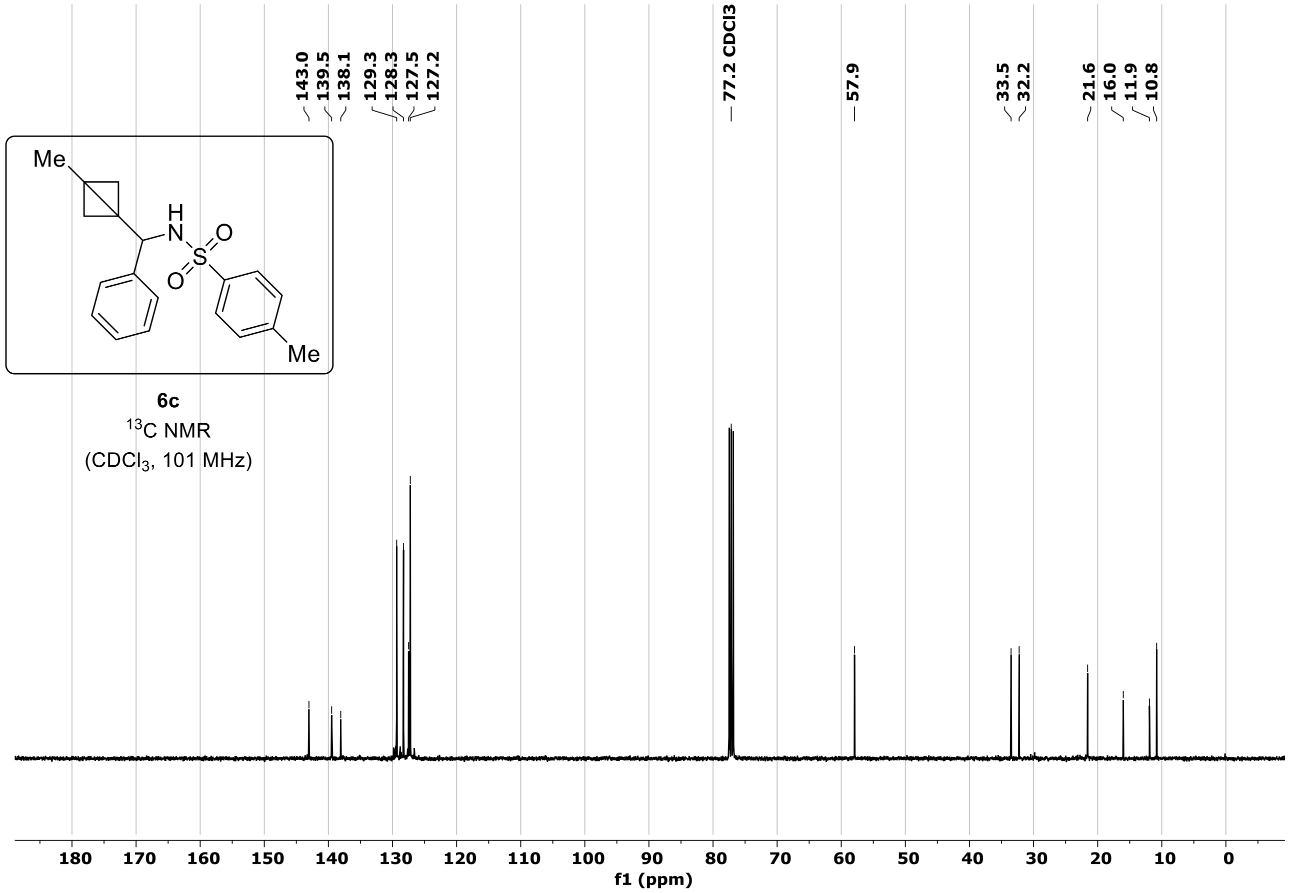
143.0
139.5
138.1
129.3
128.3
127.5
127.2
77.2 CDCl3
57.9
33.5
32.2
21.6
16.0
11.9
10.8
Me
H
N
O
S
O
Me
6c
13C NMR
(CDCl3, 101 MHz)
180 170 160 150 140 130 120 110 100 90 80 70 60 50 40 30 20 10 0
f1 (ppm)

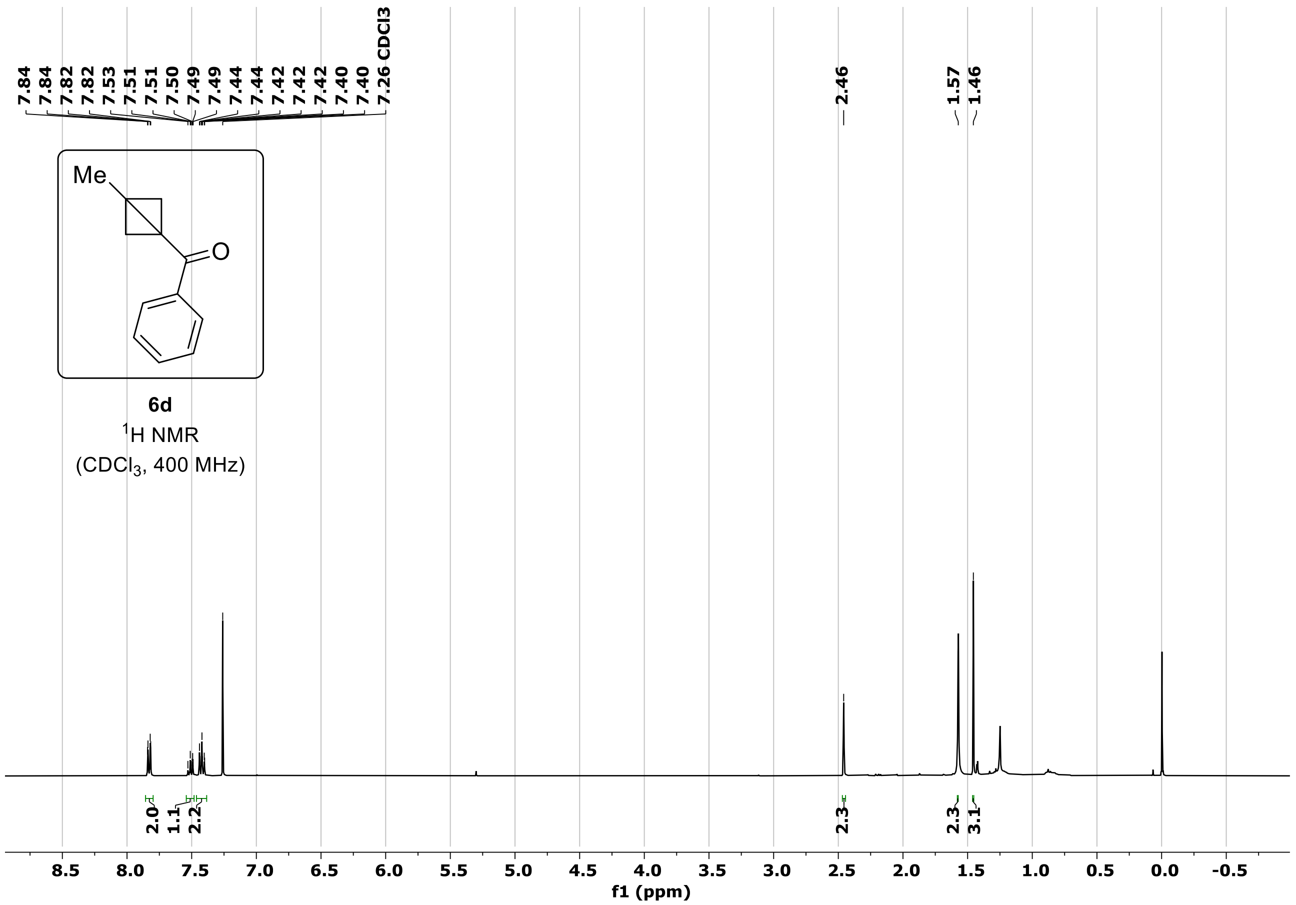
7.84
7.84
7.82
7.82
7.53
7.51
7.51
7.50
7.49
7.49
7.44
7.44
7.42
7.42
7.42
7.40
7.40
7.26 CDCl3
2.46
1.57
1.46
Me
O
6d
1H NMR
(CDCl3, 400 MHz)
2.0
1.1
2.2
2.3
2.3
3.1
8.5 8.0 7.5 7.0 6.5 6.0 5.5 5.0 4.5 4.0 3.5 3.0 2.5 2.0 1.5 1.0 0.5 0.0 -0.5
f1 (ppm)

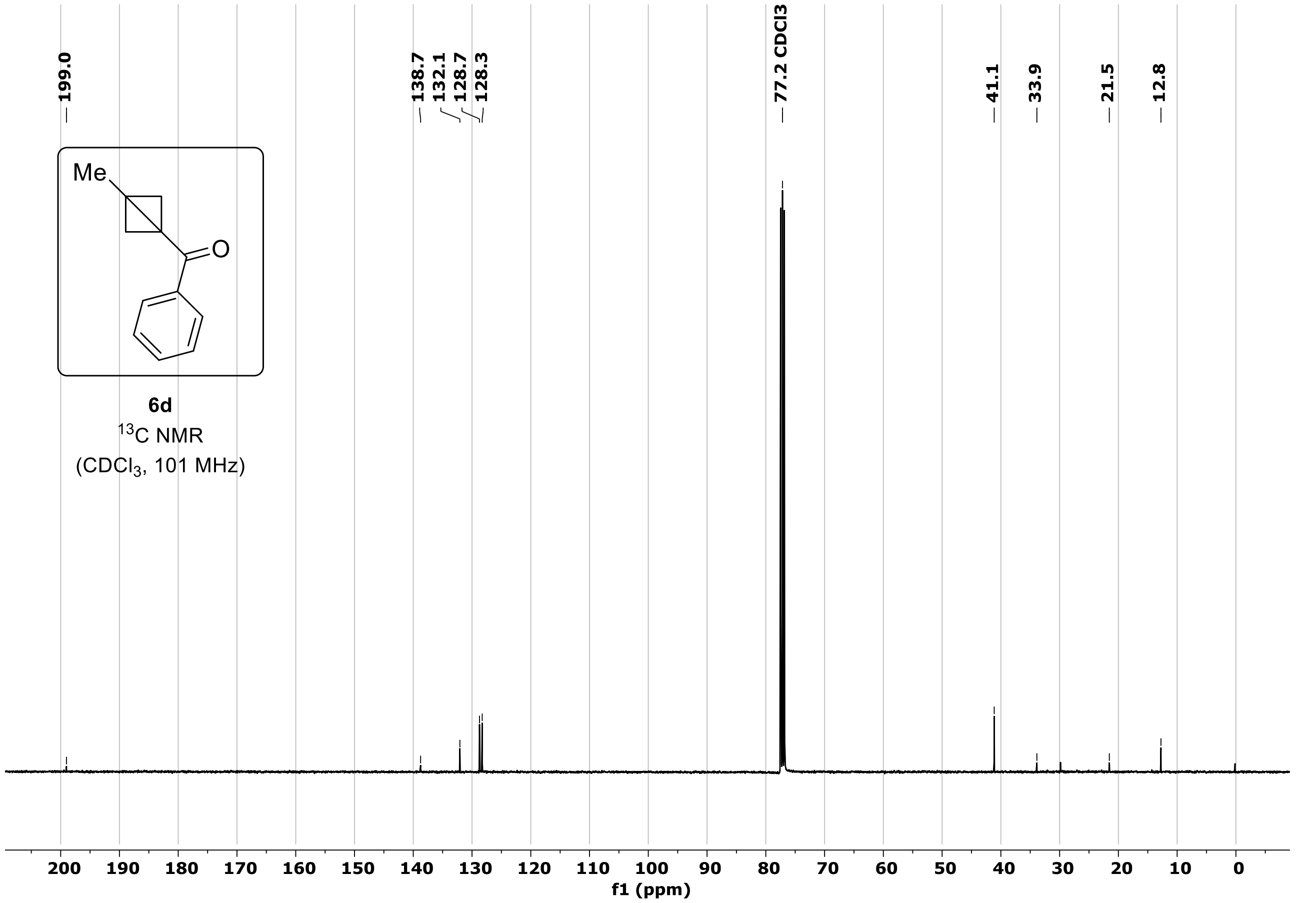
199.0
138.7
132.1
128.7
128.3
77.2 CDCl3
41.1
33.9
21.5
12.8
Me
O
6d
13C NMR
(CDCl3, 101 MHz)
f1 (ppm)

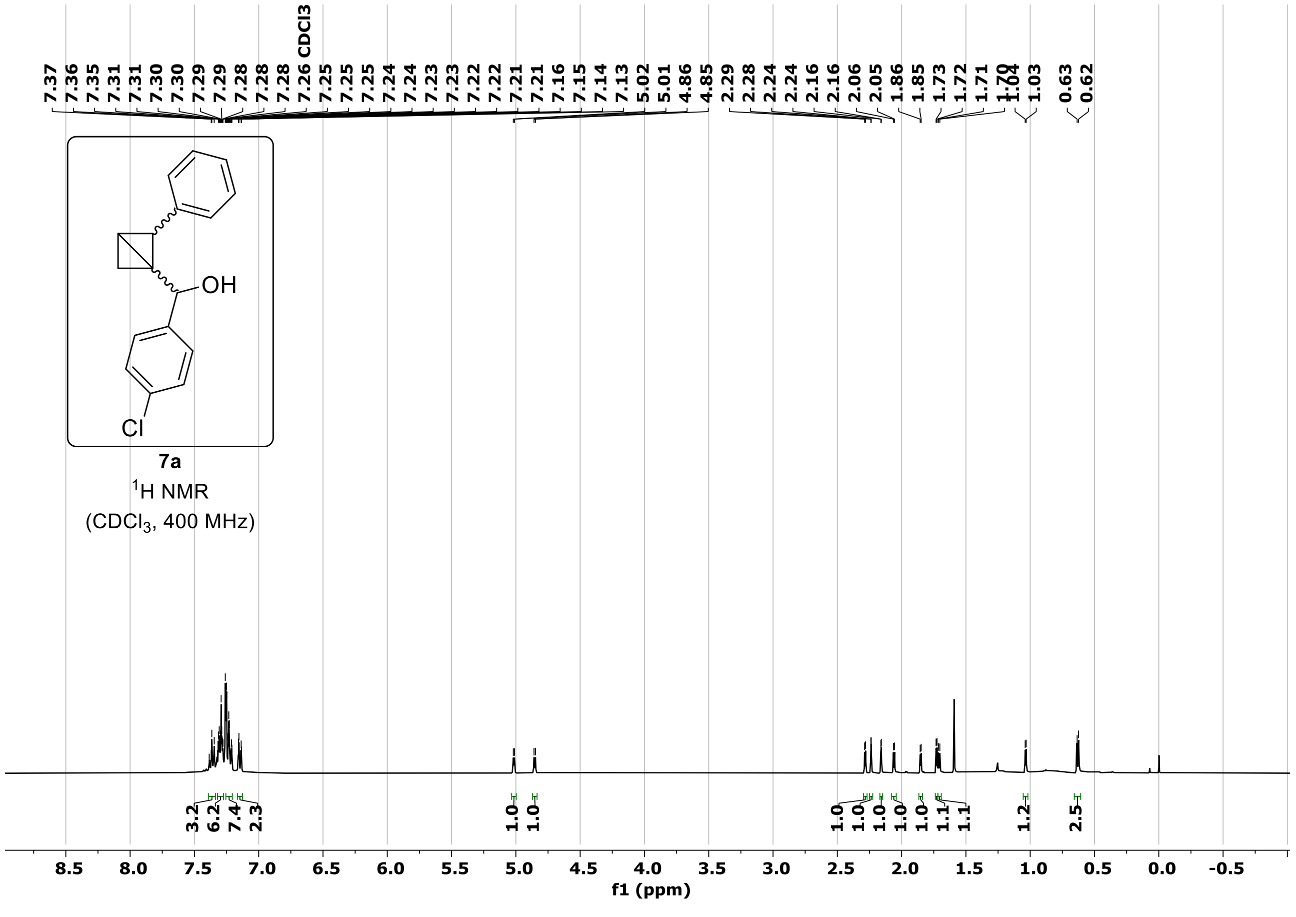
7.37
7.36
7.35
7.31
7.31
7.30
7.30
7.29
7.29
7.28
7.28
7.28
7.26 CDCl3
7.25
7.25
7.25
7.24
7.24
7.23
7.23
7.22
7.22
7.22
7.21
7.21
7.16
7.15
7.14
7.13
5.02
5.01
4.86
4.85
2.29
2.28
2.24
2.24
2.16
2.16
2.06
2.05
1.86
1.85
1.73
1.72
1.71
1.70
1.04
1.03
0.63
0.62
OH
Cl
7a
1H NMR
(CDCl3, 400 MHz)
3.2
6.2
7.4
2.3
1.0
1.0
1.0
1.0
1.0
1.0
1.1
1.1
1.2
2.5
f1 (ppm)

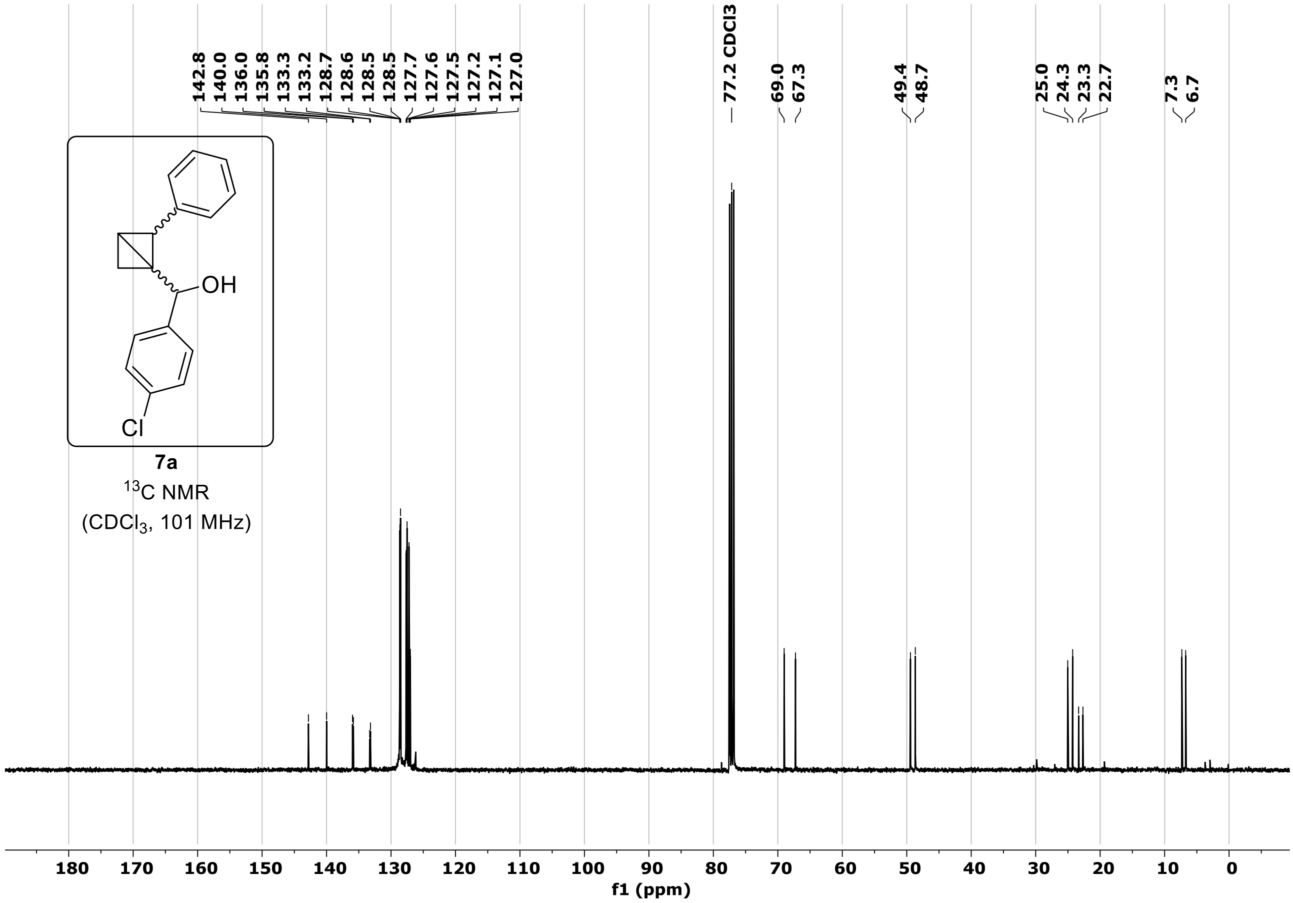
142.8
140.0
136.0
135.8
133.3
133.2
128.7
128.6
128.5
128.5
127.7
127.6
127.5
127.2
127.1
127.0
77.2 CDCl3
69.0
67.3
49.4
48.7
25.0
24.3
23.3
22.7
7.3
6.7
OH
Cl
7a
13C NMR
(CDCl3, 101 MHz)
180 170 160 150 140 130 120 110 100 90 80 70 60 50 40 30 20 10 0
f1 (ppm)

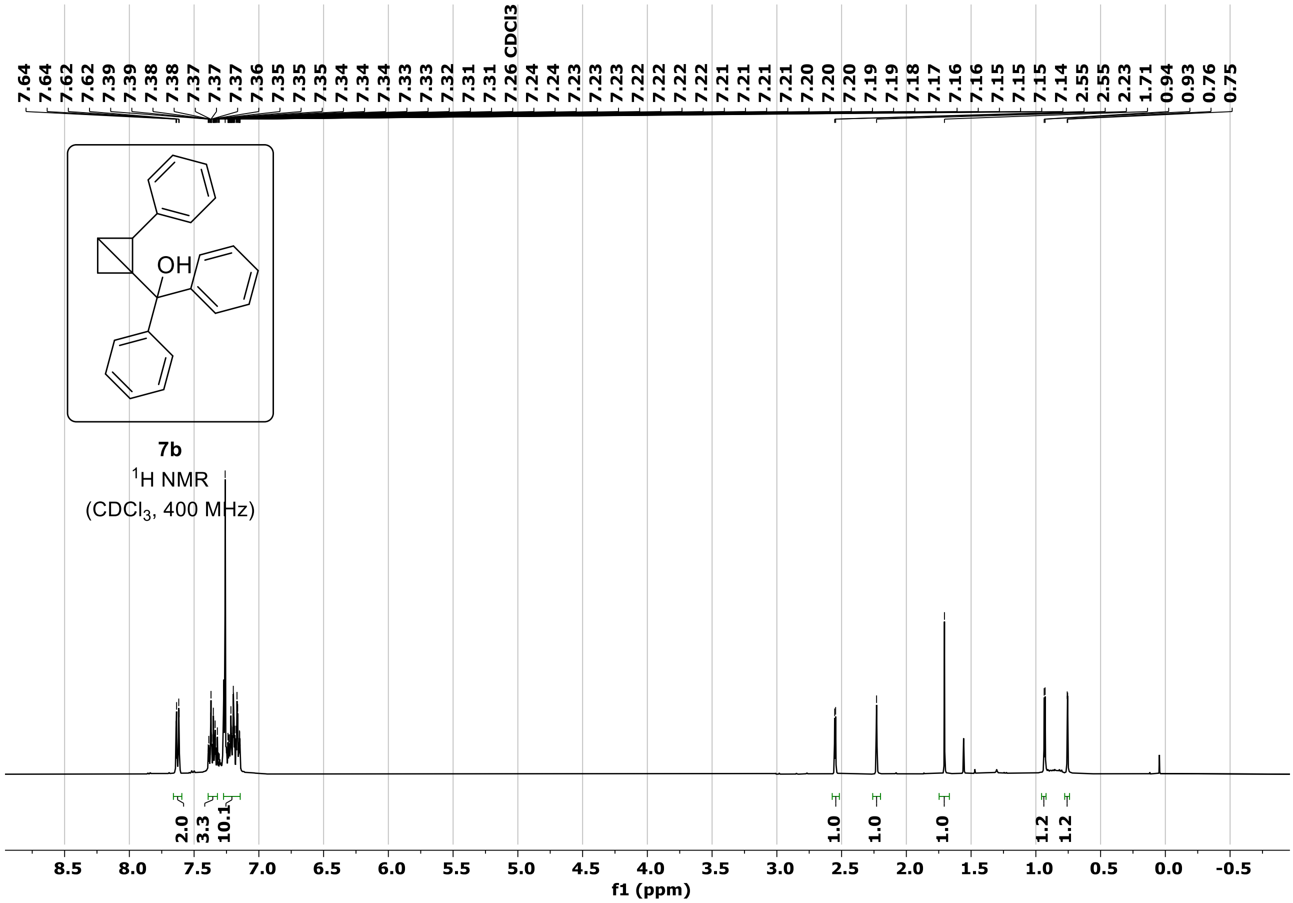
7.64
7.64
7.62
7.39
7.39
7.38
7.38
7.37
7.37
7.37
7.36
7.35
7.35
7.35
7.34
7.34
7.34
7.33
7.33
7.32
7.31
7.31
7.26 CDCl3
7.24
7.24
7.23
7.23
7.23
7.22
7.22
7.22
7.22
7.21
7.21
7.21
7.21
7.20
7.20
7.19
7.19
7.18
7.17
7.16
7.16
7.15
7.15
7.15
7.14
2.55
2.55
2.23
1.71
0.94
0.93
0.76
0.75
OH
7b
1H NMR
(CDCl3, 400 MHz)
2.0
3.3
10.1
1.0
1.0
1.0
1.2
1.2
8.5 8.0 7.5 7.0 6.5 6.0 5.5 5.0 4.5 4.0 3.5 3.0 2.5 2.0 1.5 1.0 0.5 0.0 -0.5
f1 (ppm)

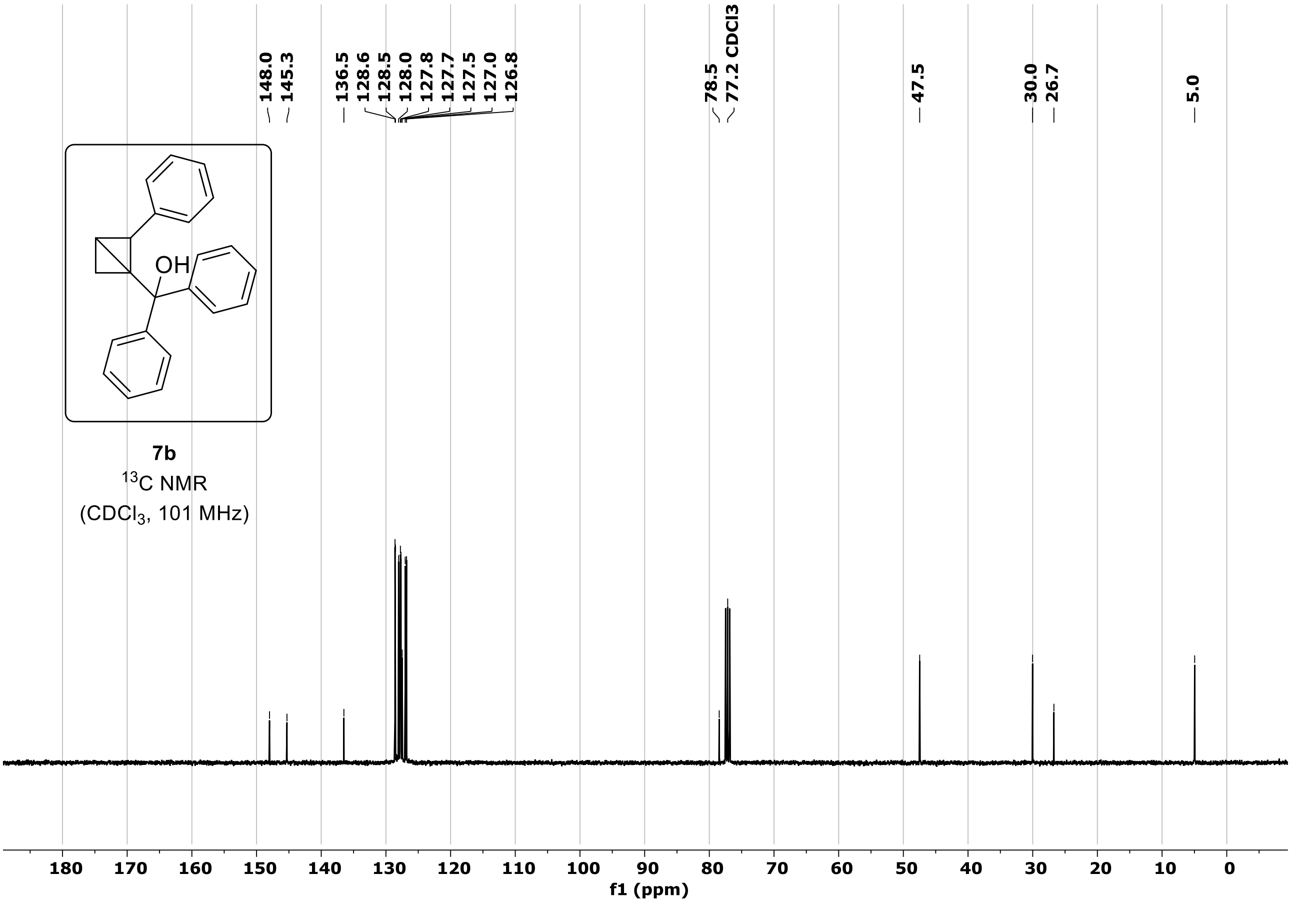
148.0
145.3
136.5
128.6
128.5
128.0
127.8
127.7
127.5
127.0
126.8
78.5
77.2 CDCl3
47.5
30.0
26.7
5.0
OH
7b
13C NMR
(CDCl3, 101 MHz)
180 170 160 150 140 130 120 110 100 90 80 70 60 50 40 30 20 10 0
f1 (ppm)

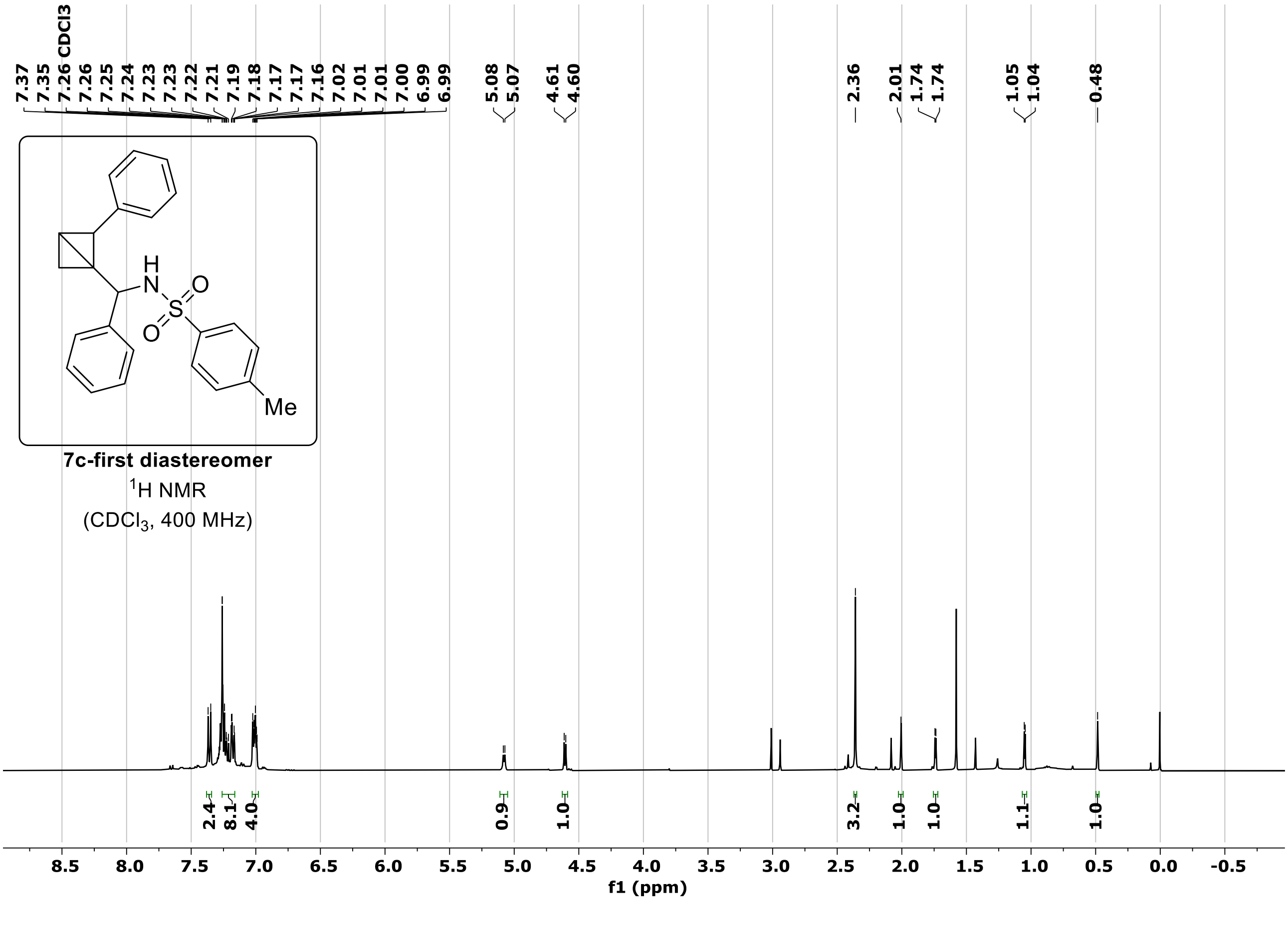
7.37
7.35
7.26 CDCl3
7.26
7.25
7.24
7.23
7.23
7.22
7.21
7.19
7.18
7.17
7.17
7.16
7.02
7.01
7.01
7.00
6.99
6.99
5.08
5.07
4.61
4.60
2.36
2.01
1.74
1.74
1.05
1.04
0.48
H
N
S
O
O
Me
7c-first diastereomer
1H NMR
(CDCl3, 400 MHz)
2.4
8.1
4.0
0.9
1.0
3.2
1.0
1.0
1.1
1.0
8.5 8.0 7.5 7.0 6.5 6.0 5.5 5.0 4.5 4.0 3.5 3.0 2.5 2.0 1.5 1.0 0.5 0.0 -0.5
f1 (ppm)

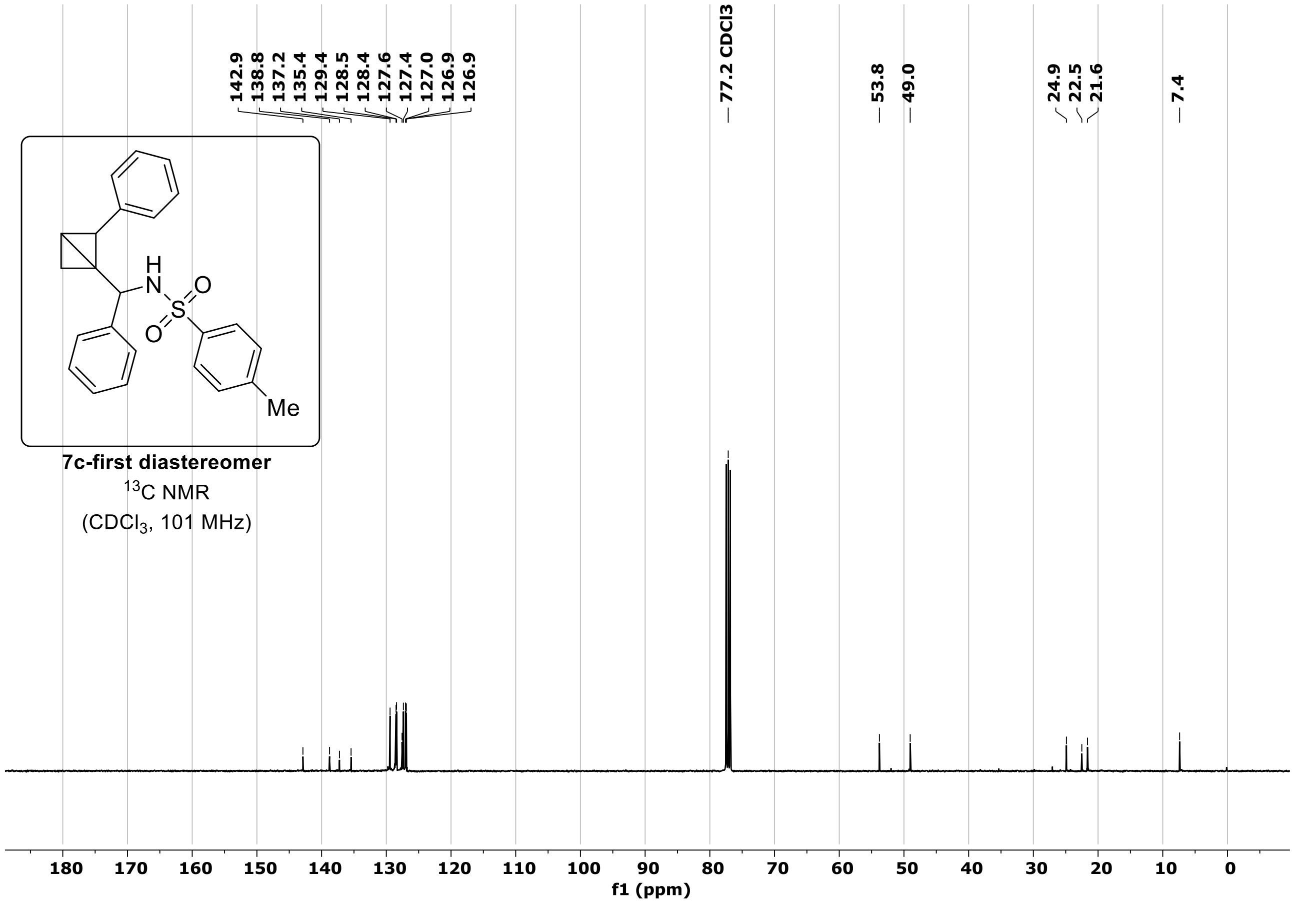
142.9
138.8
137.2
135.4
129.4
128.5
128.4
127.6
127.4
127.0
126.9
126.9
77.2 CDCl3
53.8
49.0
24.9
22.5
21.6
7.4
H
N
S
O
O
Me
7c-first diastereomer
13C NMR
(CDCl3, 101 MHz)
180 170 160 150 140 130 120 110 100 90 80 70 60 50 40 30 20 10 0
f1 (ppm)

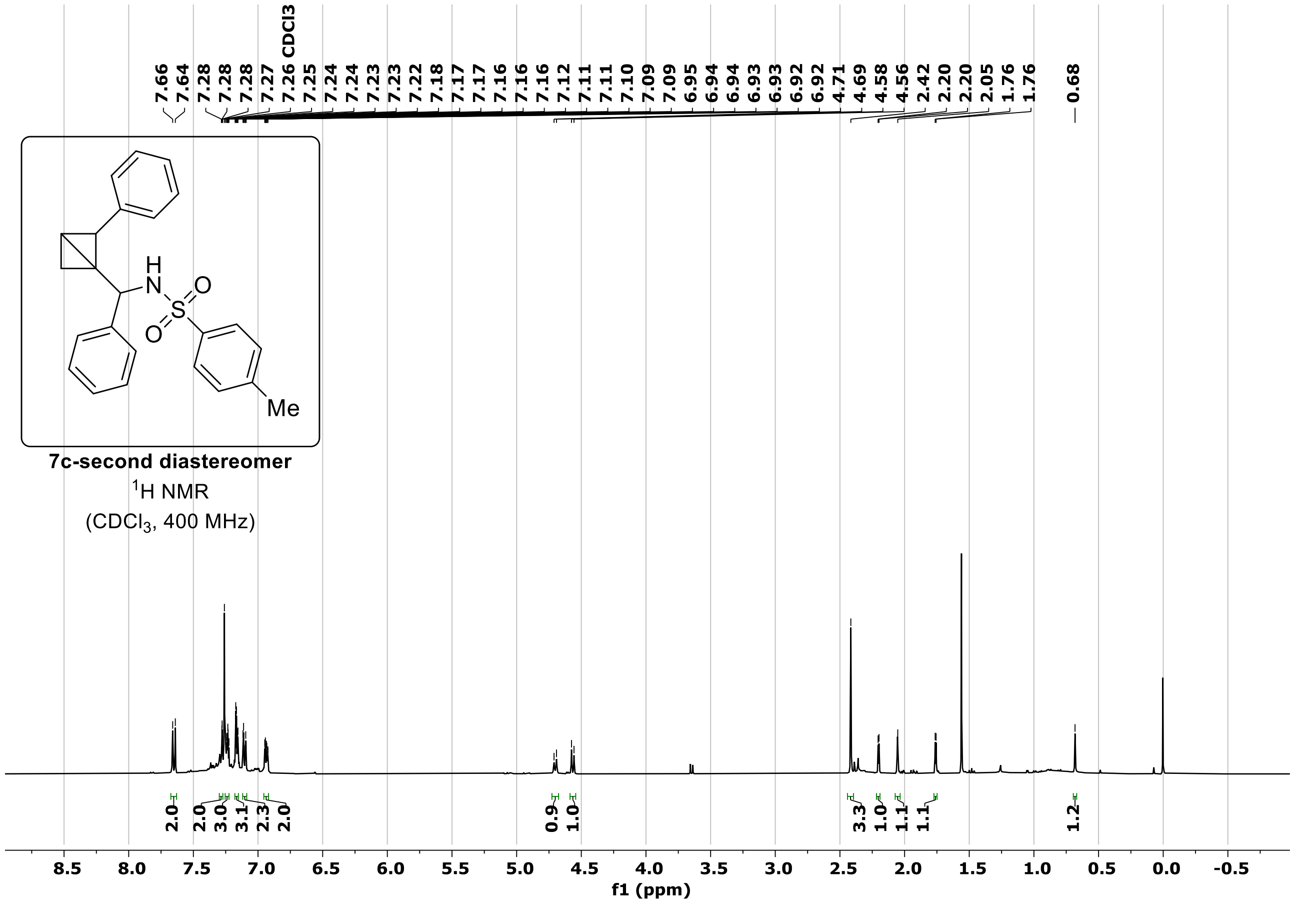
7.66
7.64
7.28
7.28
7.27
7.26 CDCl3
7.25
7.24
7.24
7.23
7.23
7.22
7.18
7.17
7.16
7.16
7.16
7.12
7.11
7.11
7.10
7.09
6.95
6.94
6.94
6.93
6.92
6.92
4.71
4.69
4.58
4.56
2.42
2.20
2.20
2.05
1.76
1.76
0.68
H
N
S
O
O
Me
7c-second diastereomer
1H NMR
(CDCl3, 400 MHz)
2.0
2.0
3.0
3.1
2.3
2.0
0.9
1.0
3.3
1.0
1.1
1.1
1.2
8.5 8.0 7.5 7.0 6.5 6.0 5.5 5.0 4.5 4.0 3.5 3.0 2.5 2.0 1.5 1.0 0.5 0.0 -0.5
f1 (ppm)

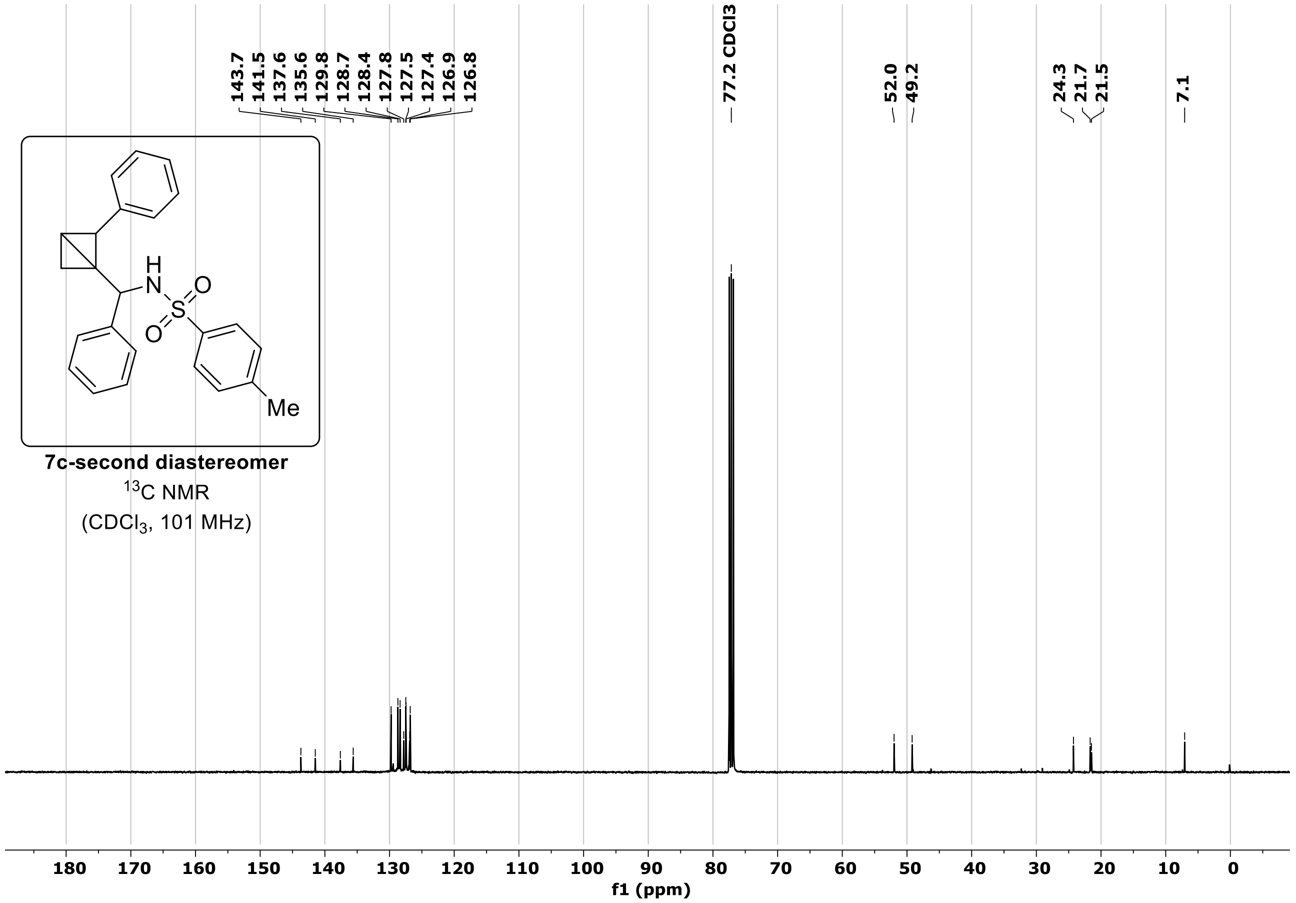
143.7 141.5 137.6 135.6 129.8 128.7 128.4 127.8 127.5 127.4 126.9 126.8
77.2 CDCl3
52.0 49.2
24.3 21.7 21.5
7.1
H
N
S
O
O
Me
7c-second diastereomer
^{13}C NMR
(CDCl$_3$, 101 MHz)
180 170 160 150 140 130 120 110 100 90 80 70 60 50 40 30 20 10 0
f1 (ppm)

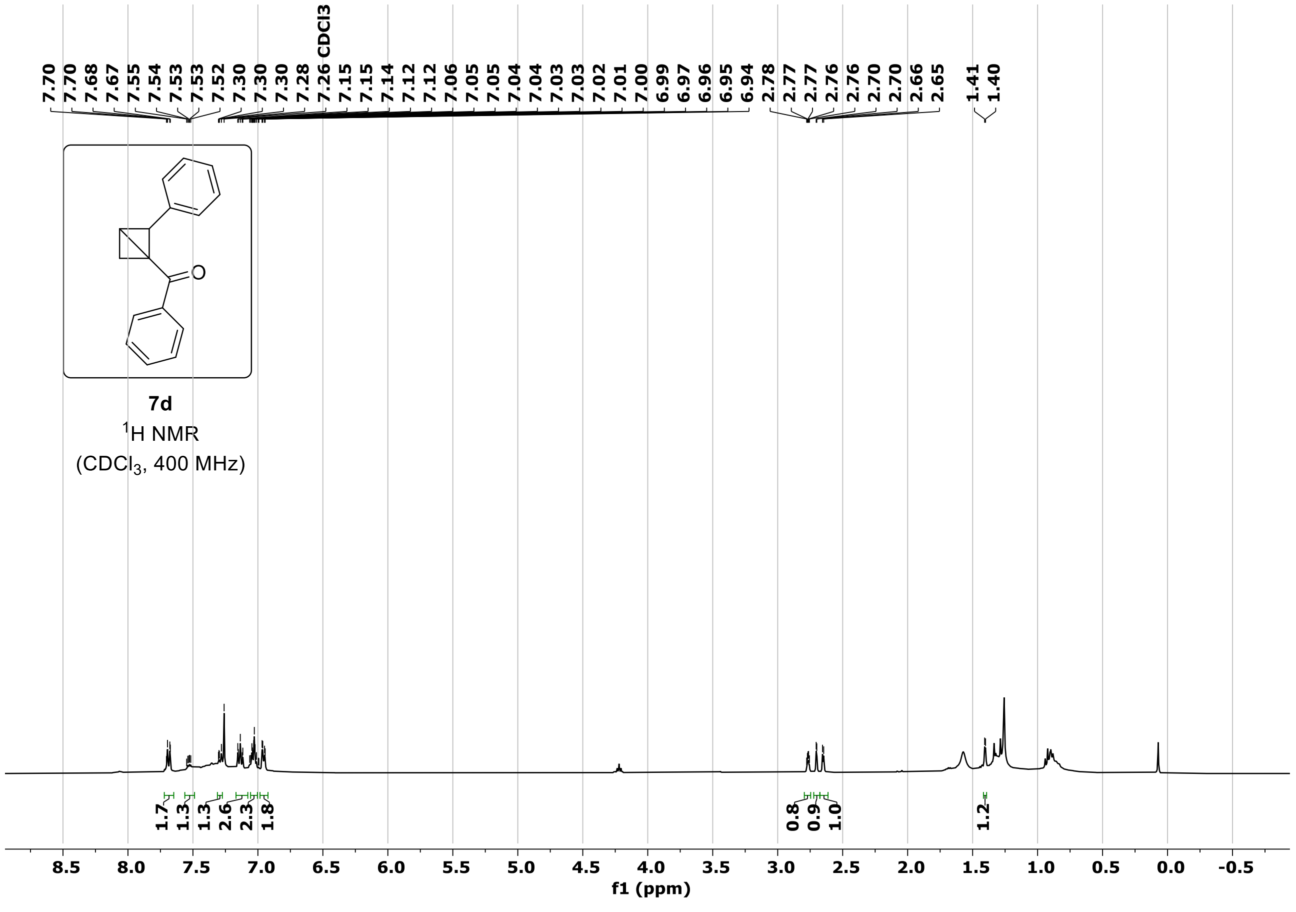
7.70 7.70 7.68 7.67 7.55 7.54 7.53 7.53 7.52 7.30 7.30 7.30 7.28 7.26 CDCl3 7.15 7.15 7.14 7.12 7.12 7.06 7.05 7.05 7.04 7.04 7.03 7.03 7.02 7.01 7.00 6.99 6.97 6.96 6.95 6.94
2.78 2.77 2.77 2.76 2.76 2.70 2.70 2.66 2.65
1.41 1.40
O
7d
^{1}H NMR
(CDCl$_3$, 400 MHz)
1.7 1.3 1.3 2.6 2.3 1.8
0.8 0.9 1.0
1.2
8.5 8.0 7.5 7.0 6.5 6.0 5.5 5.0 4.5 4.0 3.5 3.0 2.5 2.0 1.5 1.0 0.5 0.0 -0.5
f1 (ppm)

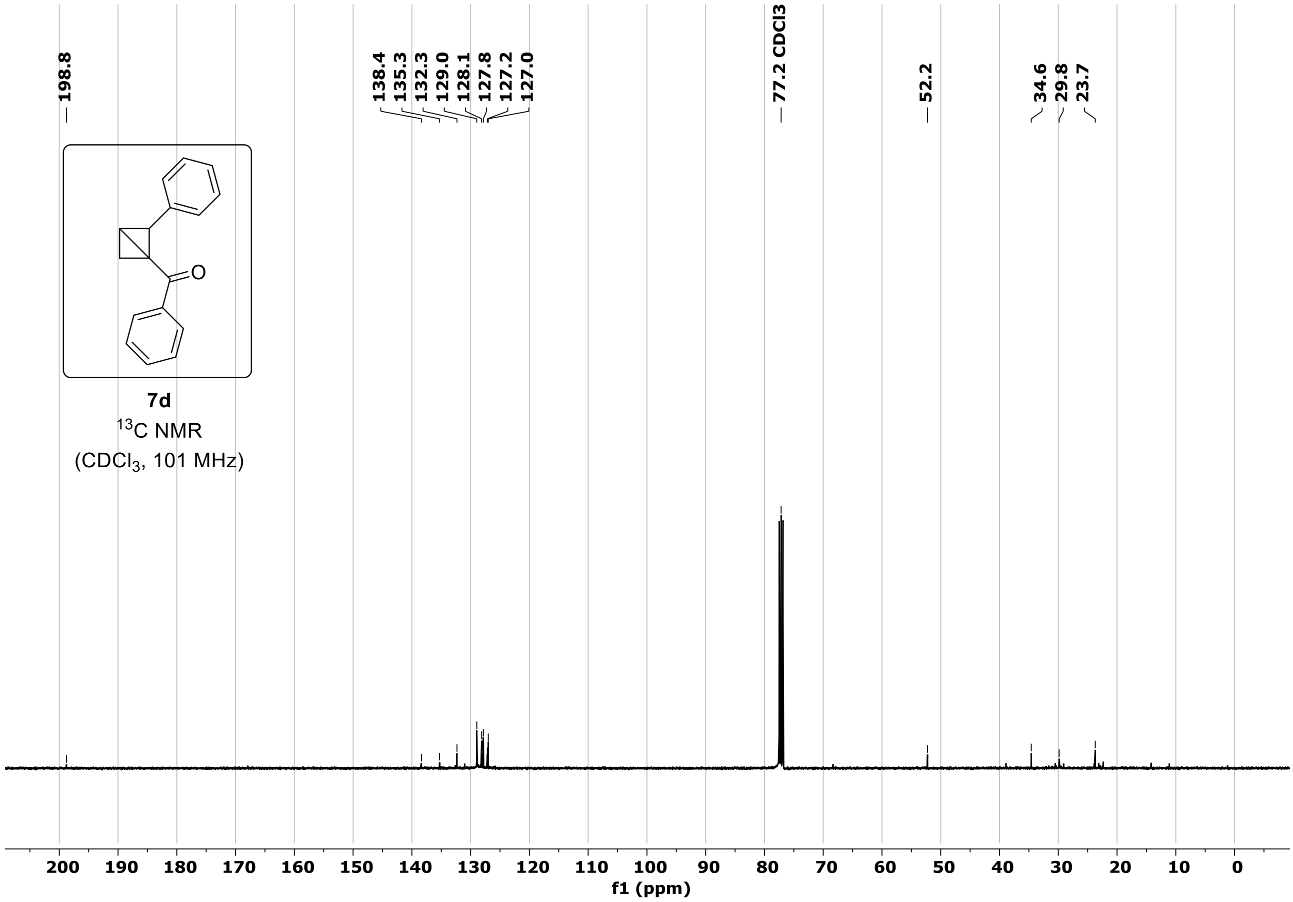
198.8
138.4
135.3
132.3
129.0
128.1
127.8
127.2
127.0
77.2 CDCl3
52.2
34.6
29.8
23.7
O
7d
^{13}C NMR
(CDCl$_3$, 101 MHz)
f1 (ppm)

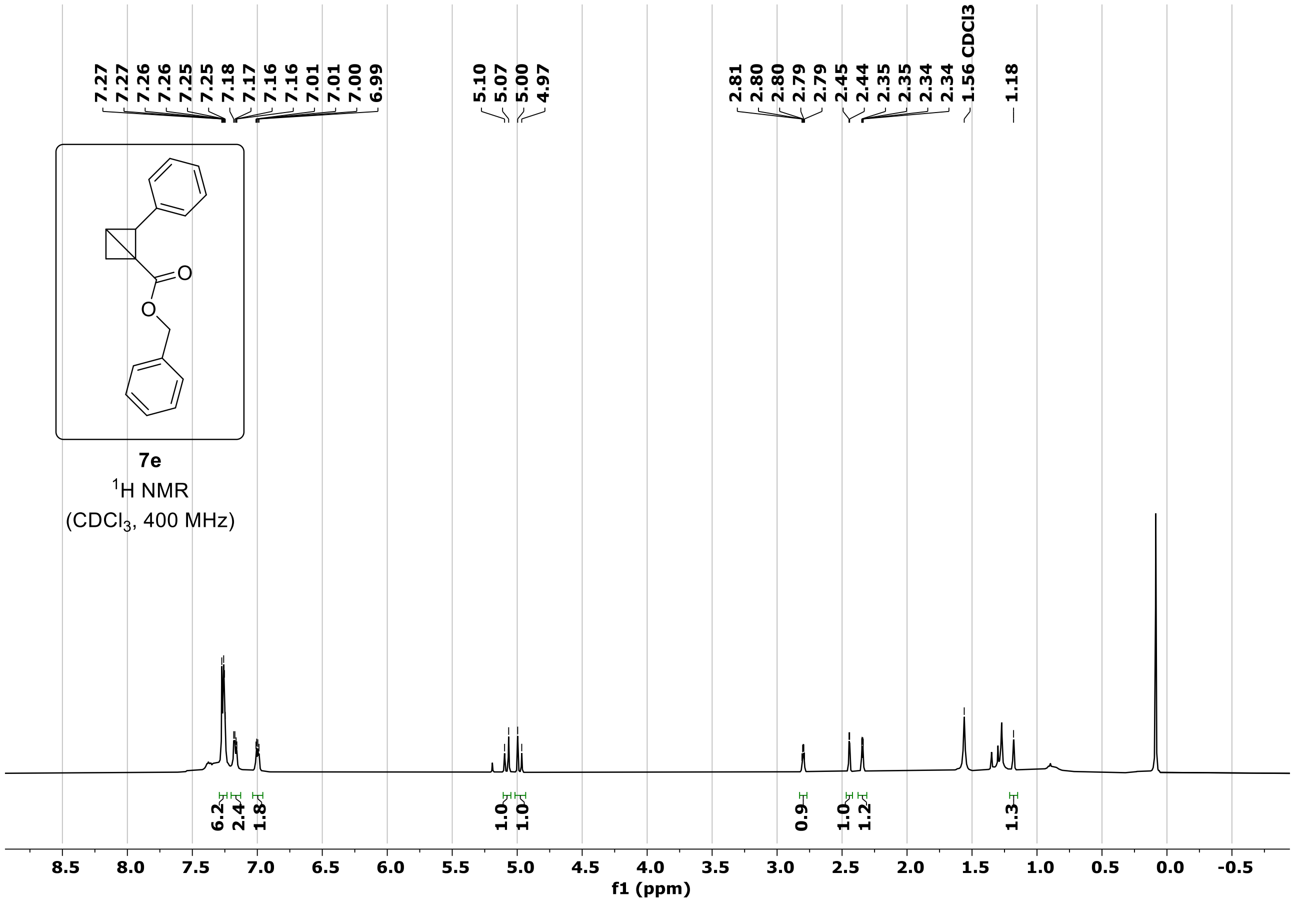
7.27
7.27
7.26
7.26
7.25
7.25
7.18
7.17
7.16
7.16
7.01
7.00
6.99
5.10
5.07
5.00
4.97
2.81
2.80
2.80
2.79
2.79
2.45
2.44
2.35
2.35
2.34
2.34
1.56 CDCl3
1.18
O
O
7e
^{1}H NMR
(CDCl$_3$, 400 MHz)
6.2
2.4
1.8
1.0
1.0
0.9
1.0
1.2
1.3
f1 (ppm)

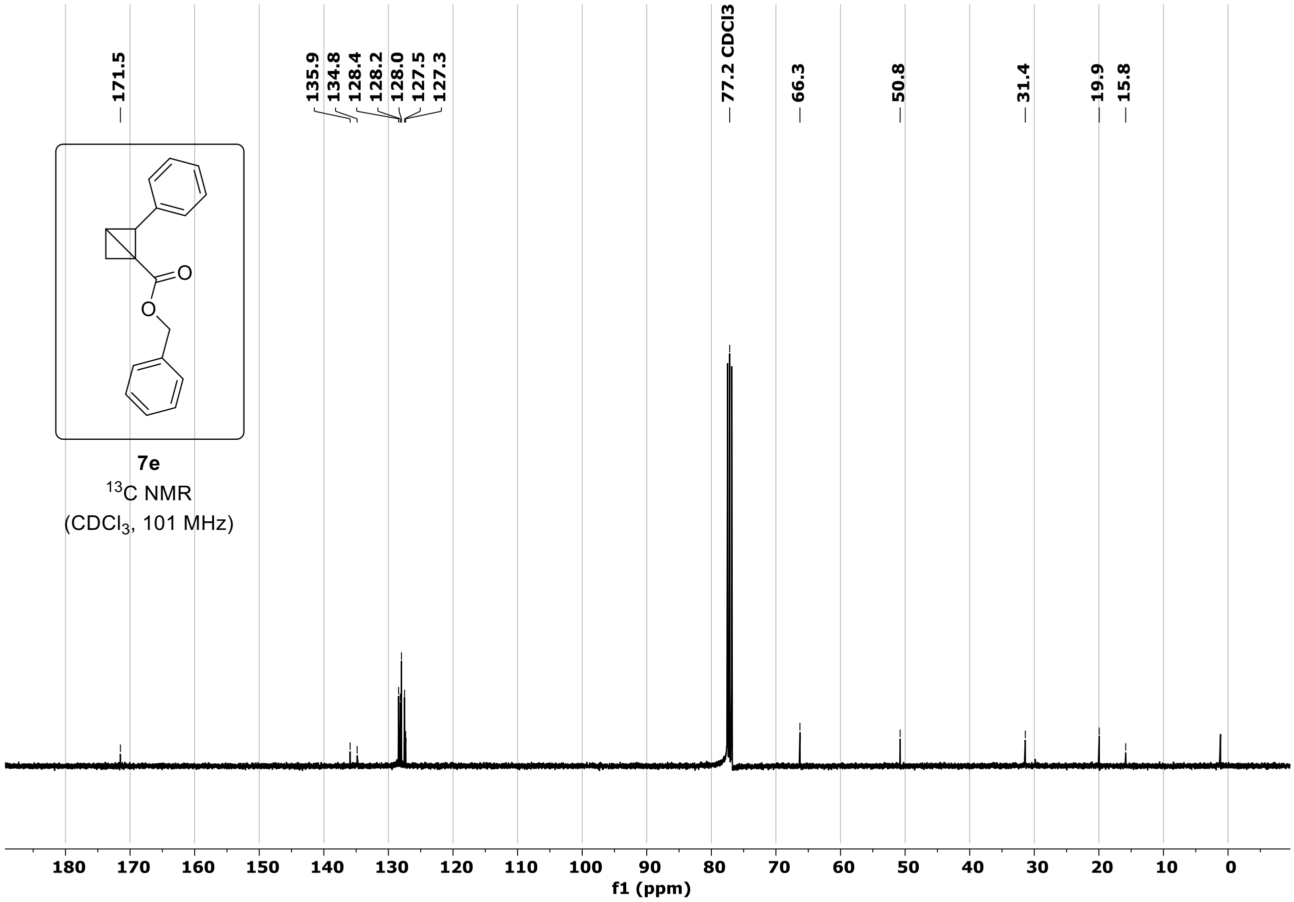

171.5
135.9
134.8
128.4
128.2
128.0
127.5
127.3
77.2 CDCl3
66.3
50.8
31.4
19.9
15.8
7e
^{13}C NMR
($CDCl_3$, 101 MHz)
180 170 160 150 140 130 120 110 100 90 80 70 60 50 40 30 20 10 0
f1 (ppm)

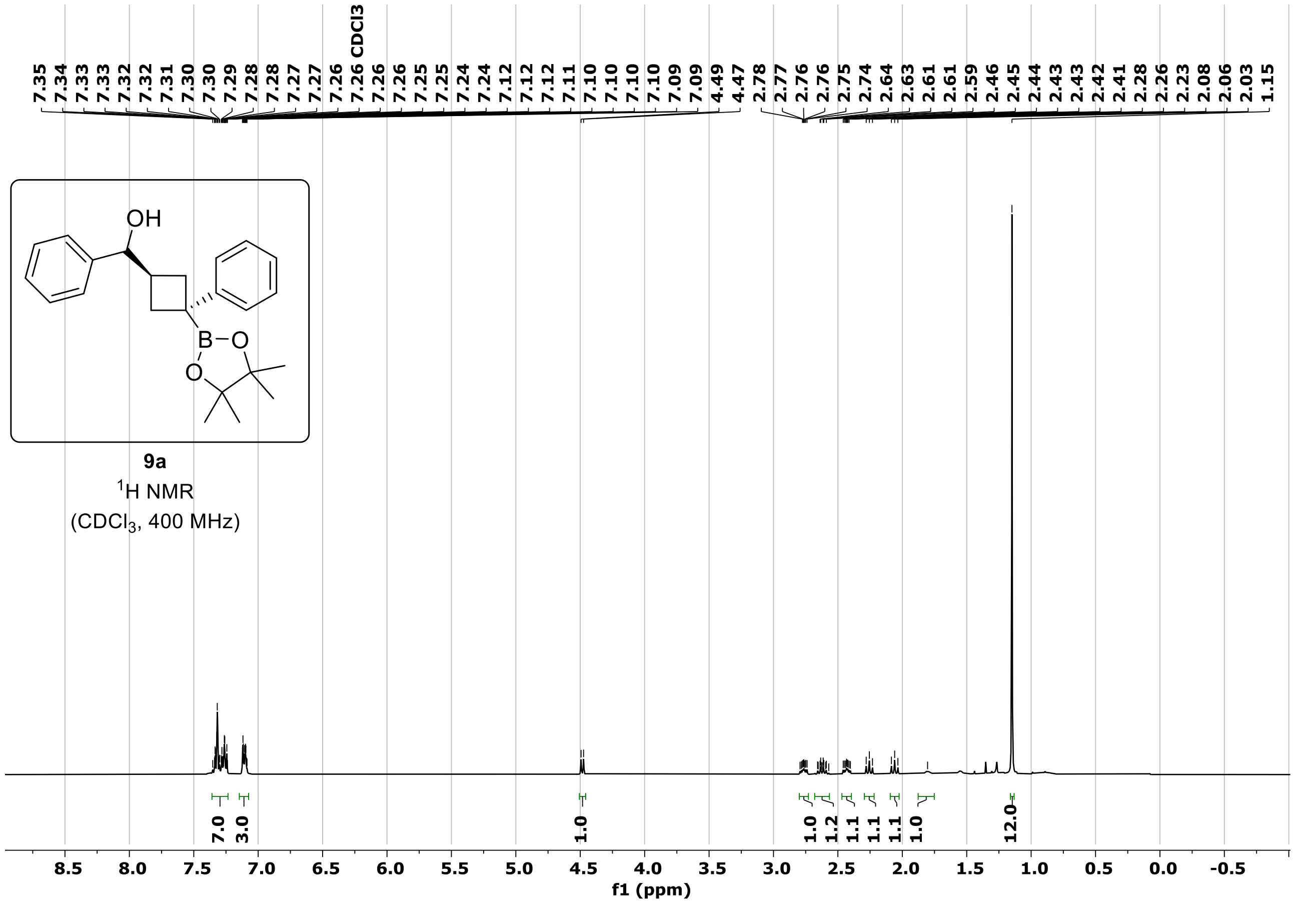

7.35 7.34 7.33 7.33 7.32 7.32 7.31 7.30 7.30 7.29 7.28 7.28 7.27 7.26 7.26 CDCl3 7.26 7.26 7.25 7.25 7.24 7.24 7.12 7.12 7.11 7.10 7.10 7.10 7.10 7.09 4.49 4.47 2.78 2.77 2.76 2.76 2.75 2.74 2.64 2.63 2.61 2.61 2.59 2.46 2.45 2.44 2.43 2.43 2.42 2.41 2.28 2.26 2.23 2.08 2.06 2.03 1.15
OH
B
O
O
9a
^{1}H NMR
($CDCl_3$, 400 MHz)
7.0
3.0
1.0
1.0
1.2
1.1
1.1
1.1
1.0
12.0
8.5 8.0 7.5 7.0 6.5 6.0 5.5 5.0 4.5 4.0 3.5 3.0 2.5 2.0 1.5 1.0 0.5 0.0 -0.5
f1 (ppm)

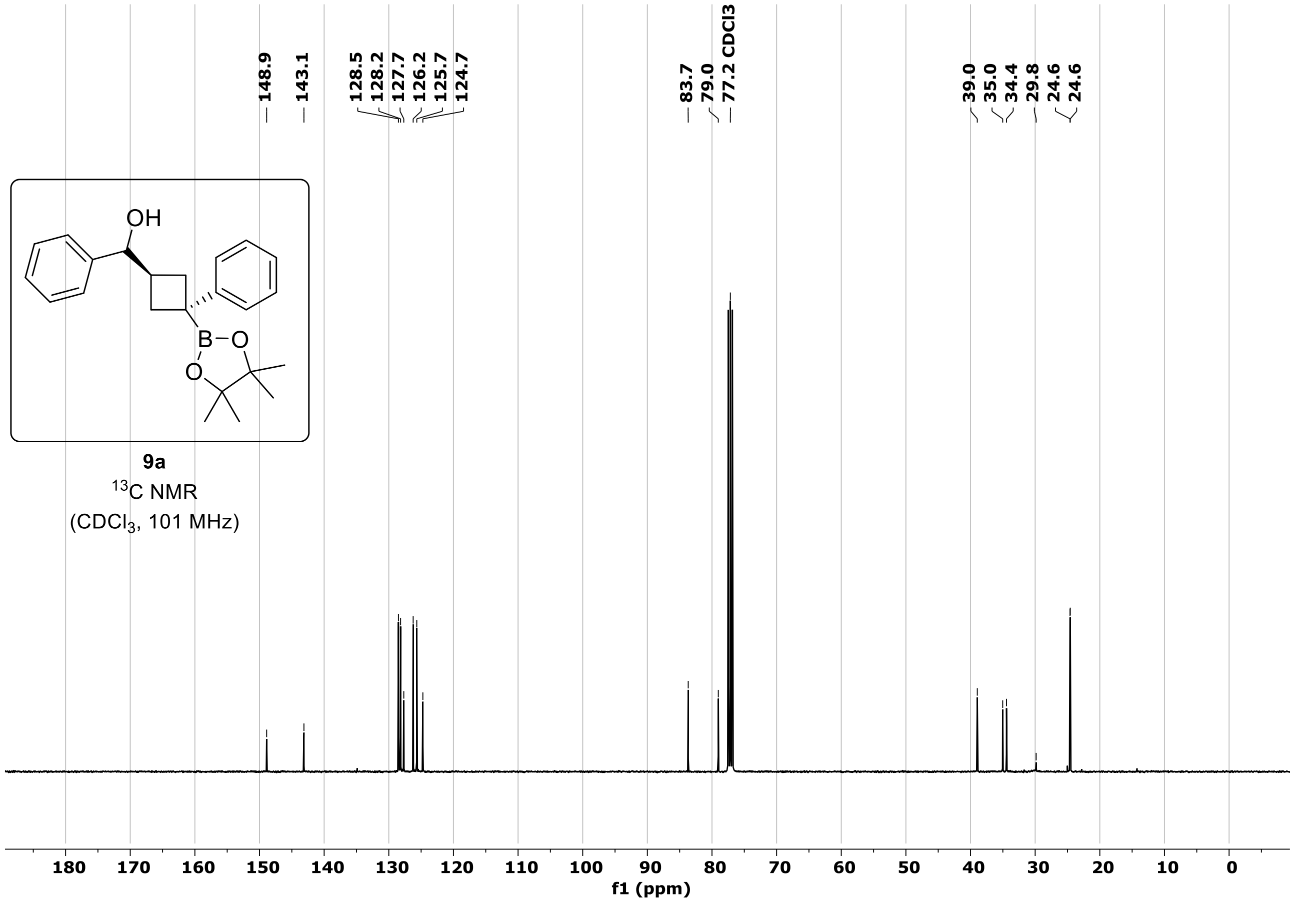
148.9
143.1
128.5
128.2
127.7
126.2
125.7
124.7
83.7
79.0
77.2 CDCl3
39.0
35.0
34.4
29.8
24.6
24.6
OH
B–O
O
9a
^{13}C NMR
(CDCl$_3$, 101 MHz)
180 170 160 150 140 130 120 110 100 90 80 70 60 50 40 30 20 10 0
f1 (ppm)

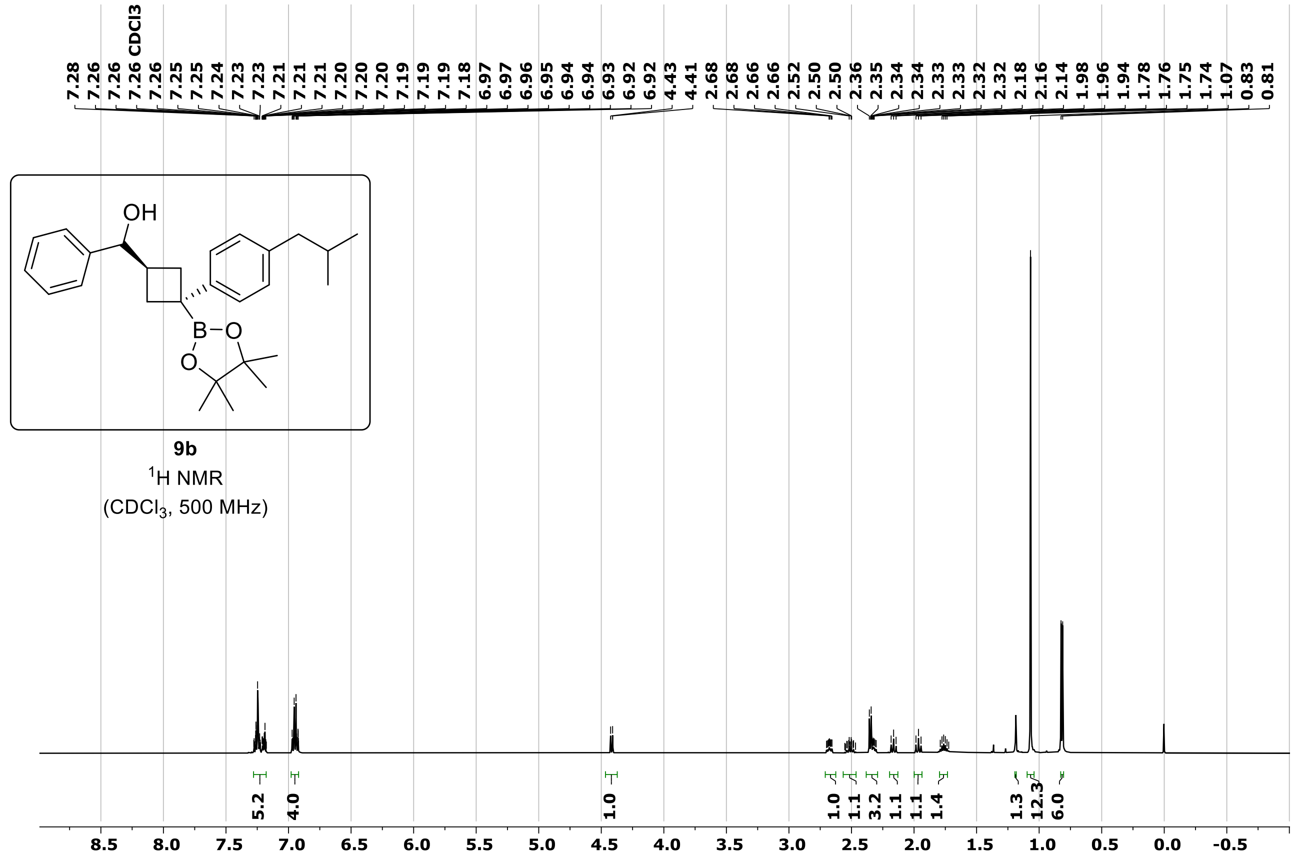
7.28 7.26 7.26 CDCl3 7.26 7.25 7.25 7.24 7.23 7.23 7.21 7.21 7.21 7.20 7.20 7.19 7.19 7.18 6.97 6.96 6.95 6.94 6.93 6.92 6.92 4.43 4.41 2.68 2.68 2.66 2.66 2.52 2.50 2.50 2.36 2.35 2.34 2.34 2.33 2.33 2.32 2.32 2.18 2.16 2.14 1.98 1.96 1.94 1.78 1.76 1.75 1.74 1.07 0.83 0.81
OH
B–O
O
9b
^{1}H NMR
(CDCl$_3$, 500 MHz)
5.2 4.0 1.0 1.0 1.1 3.2 1.1 1.1 1.4 1.3 12.3 6.0
8.5 8.0 7.5 7.0 6.5 6.0 5.5 5.0 4.5 4.0 3.5 3.0 2.5 2.0 1.5 1.0 0.5 0.0 -0.5
f1 (ppm)

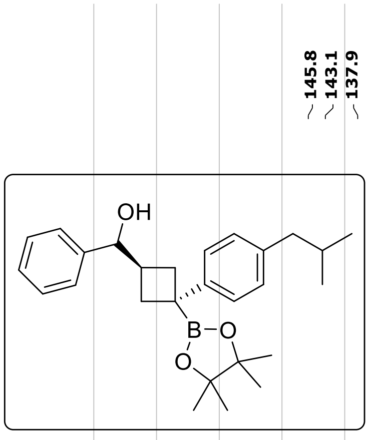

9b
^{13}C NMR
($CDCl_3$, 125.75 MHz)

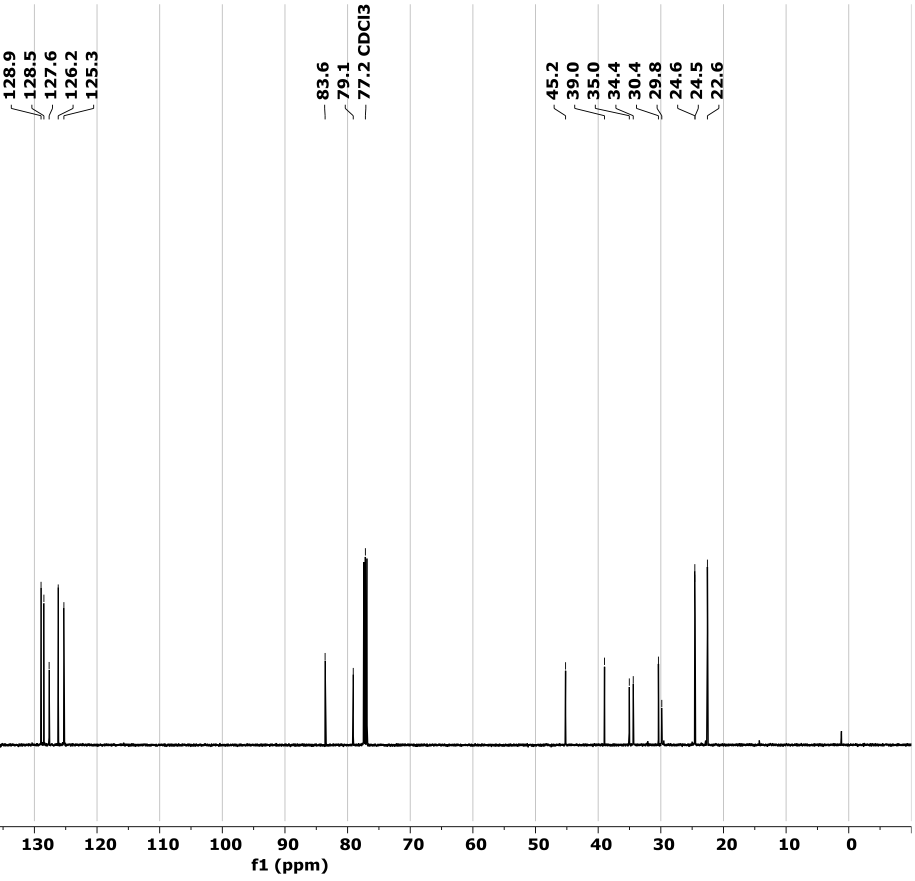

9. X-ray structure of compound 6c

Sample preparation

Crystal of compound **6c** is prepared through slow evaporation of ethyl acetate.

X-ray Diffraction Experiment for Compound 6c – CCDC2423754

The structure of compound **6c** was measured at 102(4) K using a SuperNova, dual four-circle diffractometer with an Atlas detector and a low-temperature device. Equipped with a micro-focus sealed X-ray tube, the setup utilized Cu Kα radiation (λ = 1.54184 Å) and a mirror as the monochromator. Data processing was carried out using Crysalispro, followed by Gaussian absorption correction with SCALE3 ABSPACK.[1]

To solve the structure, we used dual methods with SHELXT and refined it through full-matrix least squares methods against F2, implemented in SHELXL within the Olex2 environment.[2-4] With the exception of H1-N1 (which was found in the Fourier difference map and then freely refined) non-hydrogen atoms were refined anisotropically, while hydrogen atoms were refined isotropically using calculated positions in a riding model. Uiso values for terminal sp^3 carbons were constrained to 1.5 times, and for all other carbons to 1.2 times, the Ueq of their pivot atoms.

[1] Crysalispro, 1.171.42.92, 2023, Rigaku OD.
[2] G. M. Sheldrick, Acta Cryst. 2015, A71, 3–8, doi:10.1107/S2053273314026370.
[3] G. M. Sheldrick, Acta Cryst. 2015, C71, 3–8, doi:10.1107/S2053229614024218.
[4] O. V. Dolomanov, L. J. Bourhis, R. J. Gildea, J. A. K. Howard, H. Puschmann, J. Appl. Cryst. 2009, 42, 339-341, doi:10.1107/S0021889808042726

<table>
<tr><th>Image of crystal structure (at 50% probability level)</th><th>Key crystal data</th></tr>
<tr><td>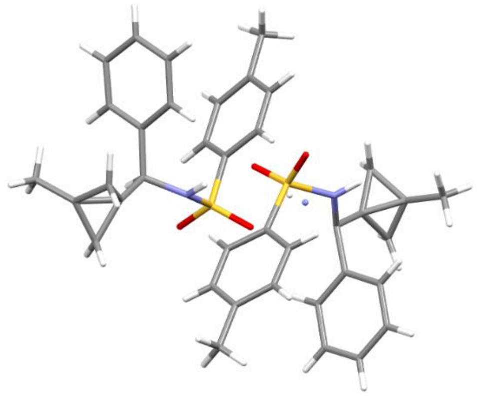</td><td>
<table>
<tr><td>Bond precision:</td><td>C-C = 0.0055 A</td><td></td><td>Wavelength=1.54184</td></tr>
<tr><td>Cell:</td><td>a=9.8498(1)
alpha=90</td><td>b=22.7648(6)
beta=91.346(2)</td><td>c=15.2911(5)
gamma=90</td></tr>
<tr><td>Temperature:</td><td>103 K</td><td></td><td></td></tr>
</table>
<table>
<tr><td></td><td>Calculated</td><td>Reported</td></tr>
<tr><td>Volume</td><td>3427.76(15)</td><td>3427.76(15)</td></tr>
<tr><td>Space group</td><td>P 21/n</td><td>P 1 21/n 1</td></tr>
<tr><td>Hall group</td><td>-P 2yn</td><td>-P 2yn</td></tr>
<tr><td>Moiety formula</td><td>C19 H20 N O2 S, C19 H21 N O2 S</td><td>C19 H20 N O2 S, C19 H21 N O2 S</td></tr>
<tr><td>Sum formula</td><td>C38 H41 N2 O4 S2</td><td>C38 H41 N2 O4 S2</td></tr>
<tr><td>Mr</td><td>653.85</td><td>653.85</td></tr>
<tr><td>Dx,g cm-3</td><td>1.267</td><td>1.267</td></tr>
<tr><td>Z</td><td>4</td><td>4</td></tr>
<tr><td>Mu (mm-1)</td><td>1.744</td><td>1.744</td></tr>
<tr><td>F000</td><td>1388.0</td><td>1388.0</td></tr>
<tr><td>F000’</td><td>1394.28</td><td></td></tr>
<tr><td>h,k,lmax</td><td>12,28,19</td><td>12,28,19</td></tr>
<tr><td>Nref</td><td>7250</td><td>7214</td></tr>
<tr><td>Tmin,Tmax</td><td>0.591,0.797</td><td>0.210,1.000</td></tr>
<tr><td>Tmin’</td><td>0.536</td><td></td></tr>
</table>
</td></tr>
</table>

10. References

[1] Agasti, S.; Beltran, F.; Pye, E.; Kaltsoyannis, N.; Crisenza, G. E.; Procter, D. J. A catalytic alkene insertion approach to bicyclo [2.1. 1] hexane bioisosteres. *Nat. Chem.* **2023**, *15*(4), 535-541.
[2] Colella, M.; Tota, A.; Takahashi, Y.; Higuma, R.; Ishikawa, S.; Degennaro, L.; Luisi, R.; Nagaki, A. Fluoro-substituted methyllithium chemistry: external quenching method using flow microreactors. *Angew. Chem., Int. Ed.* **2020**,*132*(27), 11016-11020.
[3] Alfano, A. I.; Smyth, M.; Wharry, S.; Moody, T. S.; Baumann, M. Modular Synthesis of Benzoylpyridines Exploiting a Reductive Arylation Strategy. *Org. Lett.* **2023**, *26*(14), 2847-2851.
[4] Knupe-Wolfgang, P.; Mahn, B.; Hilt, G. The Application of Flow Chemistry for the Synthesis of Alkyl Sodium Compounds and Their Transformations with Weinreb Amides and Carboxylic Acids. *Org. Lett.* **2024**, *26*(33), 6972-6976.
[5] Wölfl, B.; Winter, N.; Li, J.; Noble, A.; Aggarwal, V. K. Strain-Release Driven Epoxidation and Aziridination of Bicyclo [1.1. 0] butanes via Palladium Catalyzed σ-Bond Nucleopalladation. *Angew. Chem., Int. Ed.* ***2023,*** *62*(7), e202217064.
[6] Bennett, S. H.; Fawcett, A.; Denton, E. H.; Biberger, T.; Fasano, V.; Winter, N.; Aggarwal, V. K. Difunctionalization of C–C σ-bonds enabled by the reaction of bicyclo [1.1. 0] butyl boronate complexes with electrophiles: reaction development, scope, and stereochemical origins. *J. Am. Chem. Soc.* **2020**, *142*(39), 16766-16775.
[7] Walczak, M. A.; Wipf, P. Rhodium (I)-catalyzed cycloisomerizations of bicyclobutanes. *J. Am. Chem. Soc.* **2008**, *130*(22), 6924-6925.

[8] Schwartz, B. D.; Zhang, M. Y.; Attard, R. H.; Gardiner, M. G.; Malins, L. R. Structurally diverse acyl bicyclobutanes: valuable strained electrophiles. *Chemistry–A European Journal* **2020**, *26*(13), 2808-2812.
